# Publisher Correction: Redox-neutral photocatalytic hydrodealkenylation of aryl olefins

**DOI:** 10.1038/s41467-025-61950-4

**Published:** 2025-07-16

**Authors:** Ke Liao, Chunming Gui, Ziming Cao, Yong Huang, Jiean Chen

**Affiliations:** 1 Pingshan Translational Medicine Center, Shenzhen Bay Laboratory, Shenzhen, China; 2 College of Pharmacy, Shenzhen Technology University, Shenzhen, China; 3 Department of Chemistry, The Hong Kong University of Science and Technology, Clear Water Bay, Kowloon, Hong Kong SAR, China

**Keywords:** Photocatalysis, Photocatalysis

Correction to: *Nature Communications*; 10.1038/s41467-025-60229-y,published online 01 July 2025

In this article Fig. 2 did not display correctly; the figure should have appeared as shown below.


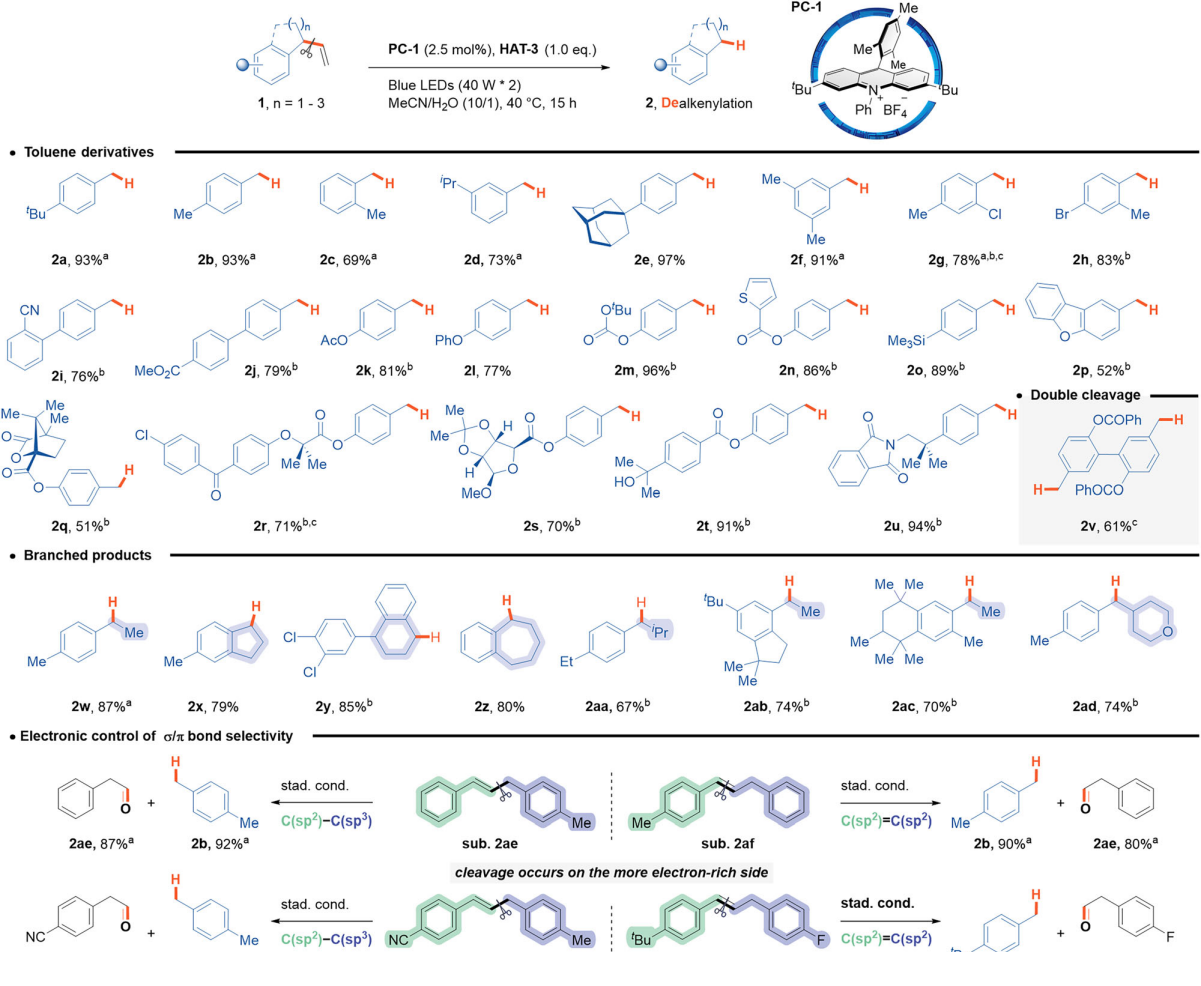


The original article has been corrected.

