# Supplementary material for: Redox-neutral photocatalytic hydrodealkenylation of aryl olefins
Source: Nat Commun. 2025 Jul 1;16:5553. doi: 10.1038/s41467-025-60229-y (PMC12216682; doi:10.1038/s41467-025-60229-y)
Supplement: Supplementary file 1 — Supplementary Information [file 41467_2025_60229_MOESM1_ESM.pdf]

Supplementary Information

Redox-neutral photocatalytic hydrodealkenylation of aryl olefins

Table of Contents

Supplementary Information 1
1. General Information 2
2. Preparation of Substrates 3
3. General procedure 18
3.1 Reaction conditions 18
3.2 Homologation of aldhydes 18
3.3 Reaction setup 19
4. Mechanistic study 31
4.1 Evidence of migration 31
4.2 ^{18}O labelling experiment 32
4.3 Light on-off/intensity experiment 32
4.4 Kinetic study 33
4.5 Stern-Volmer quenching experiment 34
4.6 Domino C−C bond cleavage 35
4.7 Plausible mechanism 36
5. ^{1}H NMR and ^{13}C NMR spectra 37
6. References 134

1. **General Information**

All solvents were distilled according to general practice before use. Solvents for flash column chromatography were technical grade and distilled before use. ^1H NMR and ^{13}C NMR data were recorded on Bruker, 400 MHz (101 MHz for ^{13}C) nuclear resonance spectrometers unless otherwise specified. ^1H and ^{13}C NMR chemical shifts are given in ppm relative to $SiMe_4$, with the solvent resonance used as the internal reference. Chemical shifts (δ) are given in parts per million and referenced to the residual solvent signal; all coupling constants are reported in Hz. The following abbreviations were used to explain the multiplicits: s (singlet), d (doublet), t (triplet), q (quartet), m (multiplet). Thin-layer chromatography (TLC) was conducted with 0.25 mm Yantai silica gel plates (60F-254) and visualized by exposure to UV light (254 nm) or stained with phosphomolybdic acid in EtOH. Flash column chromatography was performed using Tsingdao silica gel (60, particle size 0.040–0.063 mm). HRMS (ESI) analysis was performed by The Analytical Instrumentation Center at Peking University, Shenzhen Graduate School and (HRMS) data were reported with ion mass/charge (m/z) ratios as values in atomic mass units.

2. Preparation of Substrates

General procedure A for **sub. 2k**

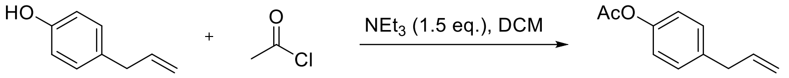

A solution of allylarene in DCM (0.25 M) and triethylamine (1.5 eq.) was added to a round-bottom flask. The mixture was stirred at 0 °C, and acetyl chloride (1.5 eq.) was added slowly. The resulting solution was stirred at room temperature for 24 h, quenched with H_2O, and extracted with DCM. The combined organic layers were washed with brine, dried over Na_2SO_4, and concentrated. The residue was purified by flash chromatography using petroleum ether and ethyl acetate on silica gel.

General procedure B for **sub. 2m**

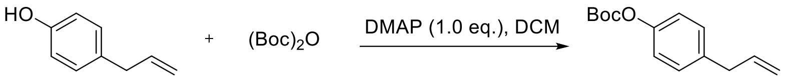

A solution of allylarene in DCM (0.25 M) and DMAP (1.0 eq.) was added to a round-bottom flask. The mixture was stirred at 0 °C, and $(Boc)_2O$ (2.2 eq.) was added. The resulting solution was stirred at room temperature for 20 min. The solution was diluted with DCM, washed with H_2O, dried over Na_2SO_4, and concentrated. The residue was purified by flash chromatography using petroleum ether and ethyl acetate on silica gel.

General procedure C for **sub. 2i**, **sub. 2p**, **sub. 2x-sub. 2z**, **sub. 2aa-sub. 2ac**

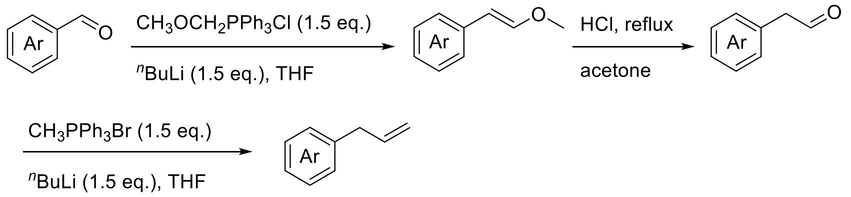

Step 1: Following a typical Wittig reaction procedure, in a round-bottom flask with a magnetic stir bar, phosphonium salt (1.5 eq.) was suspended in dry THF (0.2 M) under Ar and then cooled to 0 °C. ^{n}BuLi (1.5 eq.) was added dropwise under stirring, and the red mixture was stirred for 1 h. The corresponding aldehyde (1 eq.) in THF was added dropwise while keeping the internal temperature below 0 °C. After the addition, the mixture was warmed to room temperature and stirred overnight. The reaction was quenched by the addition of water. The layers were separated, and the aqueous layer was extracted with ethyl acetate. The combined organic layers were washed with brine, dried over anhydrous Na_2SO_4, filtered, and concentrated in vacuo. The crude residue was purified by chromatography on silica gel eluting with a petroleum ether/ethyl acetate gradient.

Step 2: The intermediate was dissolved in acetone (0.5 M), and HCl (2 M, 1.2 eq.) was added dropwise. The mixture was then heated to reflux for 2 h. After completion, the mixture was adjusted to PH=8 with saturated $NaHCO_3$, extracted with DCM, dried over anhydrous Na_2SO_4, filtered, and concentrated in vacuo. The crude residue was purified by chromatography on silica gel eluting with a petroleum ether/ethyl acetate gradient.

Step 3: The procedure for step 3 was performed according to step 1 using another Wittig reagent of CH_3PPh_3Br. **Sub. 4c-sub. 4h** were synthesized using $iPrPPh_3Br$ as a Wittig reagent.

General procedure D for **sub. 5a**, **sub. 2ad**

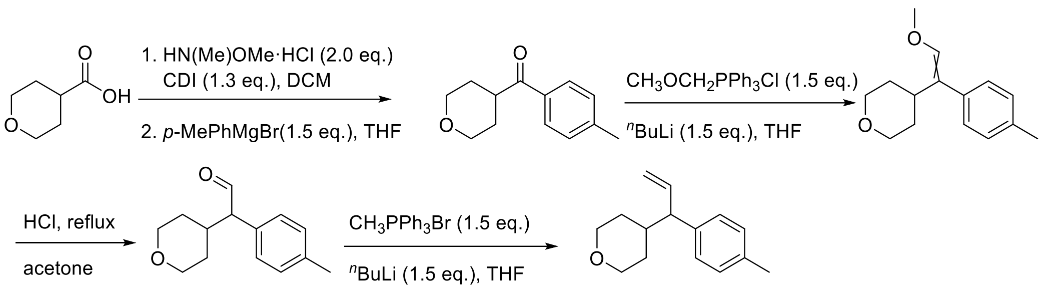

Step 1: In a round-bottom flask charged with a magnetic stir bar, a carboxylic acid (1.0 eq.) was dissolved in DCM (0.4 M), CDI (1.3 eq.) was added portionwise, and the reaction was stirred for 1 h at room temperature. N, O-Dimethylhydroxylamine hydrochloride (2.0 eq.) was added, and the reaction was stirred under Ar overnight. The crude mixture was quenched with aqueous HCl (2 M, 20 mL), washed with saturated aqueous $NaHCO_3$, dried over anhydrous Na_2SO_4 and concentrated without further purification. Then, the crude product was dissolved in THF (0.2 M) and cooled to 0°C. Grignard reagent (1.5 eq.) was added dropwise at the same temperature. After stirring overnight, the reaction was quenched with aqueous NH_4Cl, extracted with ethyl acetate, concentrated and purified by column chromatography to afford the desired product.

The follow-up steps are consistent with general procedure C.

General procedure E for **sub. 2n, sub. 2q- sub. 2s, sub. 2v**

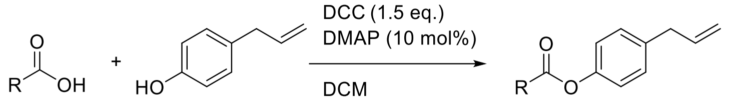

Following a typical esterification reaction procedure, in a round-bottom flask charged with a magnetic stir bar, alcohol (1.0 eq.), acid (1.2 eq.), DCC (1.5 eq.) and DMAP (10 mol%) in DCM (0.2 M) were added and stirred at room temperature for 24 h. The mixture was filtered, and the filtrate was washed with water, dried over anhydrous Na_2SO_4 and concentrated in vacuo. The crude residue was purified by chromatography on silica gel eluting with a petroleum ether/ethyl acetate gradient to afford the corresponding ester compound.

General procedure F for phosphonium salt

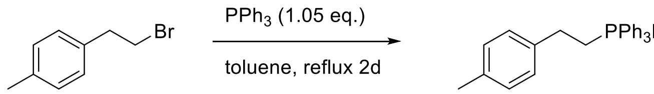

In a round-bottom flask charged with a magnetic stir bar, PPh_3 (1.05 eq.) was added to a solution of 4-methylphenethyl bromide (1.0 eq.) in toluene (1.0 M). The well-mixed reaction mixture was placed in an oil bath at 110 °C (pre-heated) for 2 days. The mixture was filtered, and the obtained white precipitate was washed with petroleum ether and dried in the vacuum to give the corresponding phosphonium salt. The salt was then used without further purification.

The synthesis for **sub. 3a- sub. 3t, sub. 5e- sub. 5g** was performed according to general procedure C, step 1, using another Wittig reagent of $^{p}MePhCH_2CH_2PPh_3Br$. For **sub. 3q**, **sub. 3r, sub. 3t**, as described in General Procedure E, an extra esterification step was needed. For **sub. 3n**, a reduction using Pd/H_2 and THF was also needed.

Substrates **[2a-2b, 2l]**(1), **2c**(4), **[2e, 2j, 2u]**(3), **2f**(5), **2g**(6), **2h**(7), **2o**(2), **2w**(8), **2x**(9), **2z**(10) , **5b**(11), **[2ae-2ag]**(12) were consistent with the reported literature.

New Compounds are characterized as shown below:

1-allyl-3-isopropylbenzene **sub. 2d**

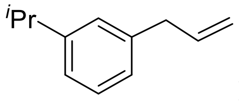

R_f= 0.9, PE. **^{1}H NMR** (400 MHz, Chloroform-*d*) δ 7.30-7.23 (m, 1H), 7.14-7.01 (m, 3H), 6.02 (ddt, *J* = 16.9, 10.0, 6.8 Hz, 1H), 5.20-5.01 (m, 2H), 3.42 (dd, *J* = 6.7, 1.6 Hz, 2H), 2.92 (hept, *J* = 6.9 Hz, 1H), 1.28 (d, *J* = 7.0 Hz, 6H). **^{13}C NMR** (101 MHz, Chloroform-*d*) δ 149.06, 140.00, 137.63, 128.38, 126.80, 126.01, 124.11, 115.68, 40.37, 34.11, 24.06. **HRMS** (ESI-TOF) calculated for: $C_{16}H_{21}$ $[M+H]^+$: 161.1325, found: 161.1326.

1-(4-allylphenyl)adamantane **sub. 2e**

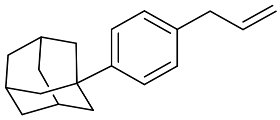

R_f= 0.3, PE. **^{1}H NMR** (400 MHz, Chloroform-*d*) δ 7.36-7.30 (m, 2H), 7.18 (m, 2H), 6.01 (ddt, *J* = 16.9, 10.0, 6.8 Hz, 1H), 5.20-5.03 (m, 2H), 3.40 (d, *J* = 6.7 Hz, 2H), 2.13-2.11 (m, 3H), 1.96-1.94 (m, 6H), 1.85-1.75 (m, 6H). **^{13}C NMR** (101 MHz, Chloroform-*d*) δ 149.21, 137.66, 137.12, 128.24, 124.92, 115.62, 43.26, 39.80, 36.85, 35.92, 29.00. **HRMS** (ESI-TOF) calculated for: $C_{19}H_{24}$ $[M+H]^+$: 253.1951, found: 253.1954.

4'-allyl-[1,1'-biphenyl]-2-carbonitrile **sub. 2i**

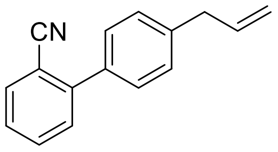

R_f= 0.5, PE/EA = 20:1 (v/v). **^{1}H NMR** (400 MHz, Chloroform-*d*) δ 7.78 (dd, *J* = 7.8, 1.4 Hz, 1H), 7.66 (td, *J* = 7.7, 1.4 Hz, 1H), 7.55-7.52 (m, 3H), 7.45 (td, *J* = 7.6, 1.3 Hz, 1H), 7.36-7.34 (m, 2H), 6.04 (ddt, *J* = 16.9, 10.1, 6.7 Hz, 1H), 5.20-5.14 (m, 2H), 3.49 (dd, *J* = 6.7, 1.6 Hz, 2H). **^{13}C NMR** (101 MHz, Chloroform-*d*) δ 145.43, 140.83, 136.94, 135.99, 133.77, 132.81, 130.04, 128.99, 128.83, 127.39, 118.86, 116.32, 111.23, 39.96. **HRMS** (ESI-TOF) calculated for: $C_{16}H_{13}N$ $[M+H]^+$: 220.1121, found: 220.1122.

methyl 4'-allyl-[1,1'-biphenyl]-4-carboxylate **sub. 2j**

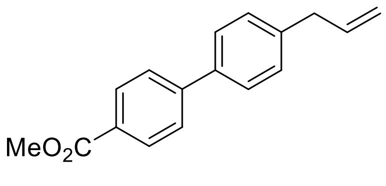

R_f= 0.3, PE/EA = 50:1 (v/v). **^{1}H NMR** (400 MHz, Chloroform-*d*) δ 8.12 (d, *J* = 8.5 Hz, 2H), 7.69-7.66 (m, 2H), 7.60-7.58 (m, 2H), 7.33-7.31 (m, 2H), 6.03 (ddt, *J* = 16.9, 10.1, 6.7 Hz, 1H), 5.18-5.12 (m, 2H), 3.97 (s, 3H), 3.47 (dt, *J* = 6.7, 1.5 Hz, 2H). **^{13}C NMR** (101 MHz, Chloroform-*d*) δ 167.05, 145.48, 140.26, 137.83, 137.11, 130.11, 129.21, 128.71, 127.32, 126.88, 116.16, 52.13, 39.89. **HRMS** (ESI-TOF) calculated for: $C_{17}H_{16}O_2$ $[M+H]^+$: 253.1223, found: 253.1223.

4-allylphenyl acetate **sub. 2k**

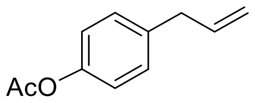

R_f = 0.3, PE/EA = 50:1 (v/v). **^{1}H NMR** (400 MHz, Chloroform-*d*) δ 7.23-7.21 (m, 2H), 7.04-7.02 (m, 2H), 5.98 (ddt, *J* = 15.7, 10.4, 6.7 Hz, 1H), 5.14-5.09 (m, 2H), 3.41 (dt, *J* = 6.7, 1.6 Hz, 2H), 2.32 (s, 3H). ^{13}C NMR (101 MHz, Chloroform-*d*) δ 169.65, 148.99, 137.63, 137.15, 129.53, 121.44, 116.08, 39.60, 21.14. **HRMS** (ESI-TOF) calculated for: $C_{11}H_{12}O_2$ $[M+H]^+$: 177.0910, found: 177.0911.

4-allylphenyl tert-butyl carbonate **sub. 2m**

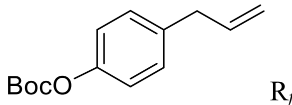

R_f = 0.5, PE/EA = 50:1 (v/v). **^{1}H NMR** (400 MHz, Chloroform-*d*) δ 7.22-7.19 (m, 2H), 7.13-7.10 (m, 2H), 5.97 (ddt, *J* = 16.1, 10.7, 6.7 Hz, 1H), 5.13-5.08 (m, 2H), 3.40 (dt, *J* = 6.7, 1.6 Hz, 2H), 1.58 (s, 9H). **^{13}C NMR** (101 MHz, Chloroform-*d*) δ 152.09, 149.39, 137.54, 137.17, 129.47, 121.17, 116.03, 83.42, 39.57, 27.72. **HRMS** (ESI-TOF) calculated for: $C_{14}H_{18}O_3$ $[M+Na]^+$: 257.1148, found: 257.1148.

4-allylphenyl thiophene-2-carboxylate **sub. 2n**

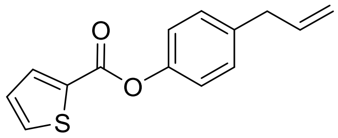

R_f = 0.4, PE/EA = 50:1 (v/v). **^{1}H NMR** (400 MHz, Chloroform-*d*) δ 8.01 (dd, *J* = 3.8, 1.3 Hz, 1H), 7.68 (dd, *J* = 5.0, 1.3 Hz, 1H), 7.27 (d, *J* = 8.5 Hz, 2H), 7.21-7.16 (m, 3H), 6.01 (ddt, *J* = 15.8, 10.4, 6.7 Hz, 1H), 5.16-5.11 (m, 2H), 3.45-3.43 (m, 2H). **^{13}C NMR** (101 MHz, Chloroform-*d*) δ 160.74, 148.89, 137.81, 137.17, 134.64, 133.44, 133.02, 129.60, 128.03, 121.54, 116.12, 39.63. **HRMS** (ESI-TOF) calculated for: $C_{14}H_{12}O_2S$ $[M+H]^+$: 245.0631, found: 245.0631.

3-allyldibenzo[b,d]furan **sub. 2p**

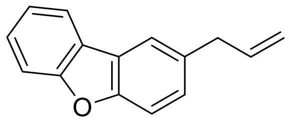

R_f = 0.8, PE/EA = 50:1 (v/v). **^{1}H NMR** (400 MHz, Chloroform-*d*) δ 7.96 (dt, *J* = 7.7, 1.0 Hz, 1H), 7.80-7.79 (m, 1H), 7.59-7.57 (m, 1H), 7.53-7.45 (m, 2H), 7.37-7.30 (m, 2H), 6.08 (ddt, *J* = 16.7, 10.1, 6.6 Hz, 1H), 5.18-5.13 (m, 2H), 3.58 (dt, *J* = 6.6, 1.5 Hz, 2H). **^{13}C NMR** (101 MHz, Chloroform-*d*) δ 156.53, 154.93, 137.88, 134.52, 127.87, 127.04, 124.36, 124.25, 122.60, 120.63, 120.38, 115.85, 111.68, 111.40, 40.13. **HRMS** (ESI-TOF) calculated for: $C_{15}H_{12}O$ $[M+H]^+$: 209.0961, found: 209.0961.

4-allylphenyl (1S,4R)-4,7,7-trimethyl-3-oxo-2-oxabicyclo[2.2.1]heptane-1-carboxylate **sub. 2q**

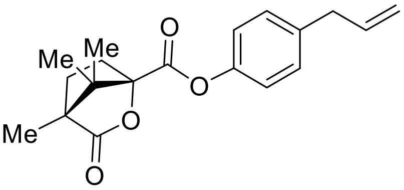

R_f= 0.4, PE/EA = 5:1 (v/v). [1]**H NMR** (400 MHz, Chloroform-*d*) δ 7.24 (d, *J* = 8.5 Hz, 2H), 7.07 (d, *J* = 8.6 Hz, 2H), 6.02-5.92 (m, 1H), 5.13-5.09 (m, 2H), 3.42 (dd, *J* = 6.6, 1.6 Hz, 2H), 2.59 (ddd, *J* = 13.5, 10.8, 4.3 Hz, 1H), 2.22 (ddd, *J* = 13.6, 9.4, 4.6 Hz, 1H), 2.02 (ddd, *J* = 13.2, 10.8, 4.6 Hz, 1H), 1.79 (ddd, *J* = 13.4, 9.3, 4.2 Hz, 1H), 1.18 (d, *J* = 8.2 Hz, 6H), 1.13 (s, 3H). [13]**C NMR** (101 MHz, Chloroform-*d*) δ 177.91, 166.24, 148.27, 138.30, 136.96, 129.70, 121.17, 116.24, 90.89, 54.91, 54.69, 39.55, 30.76, 29.00, 16.89, 9.76. **HRMS** (ESI-TOF) calculated for: $C_{19}H_{22}O_4$ $[M+H]^+$: 315.1591, found: 315.1591.

4-allylphenyl 2-(4-(4-chlorobenzoyl)phenoxy)-2-methylpropanoate **sub. 2r**

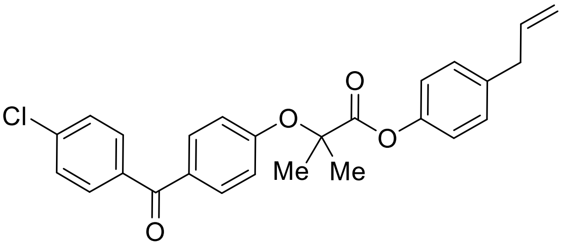

R_f = 0.6, PE/EA = 5:1 (v/v). [1]**H NMR** (400 MHz, Chloroform-*d*) δ 7.82-7.80 (m, 2H), 7.75-7.73 (m, 2H), 7.49-7.47 (m, 2H), 7.20 (d, *J* = 8.5 Hz, 2H), 7.03-7.01 (m, 2H), 6.93 (d, *J* = 8.5 Hz, 2H), 6.01-5.90 (m, 1H), 5.12-5.07 (m, 2H), 3.40 (dt, *J* = 6.6, 1.6 Hz, 2H), 1.85 (s, 6H). [13]**C NMR** (101 MHz, Chloroform-*d*) δ 194.26, 172.55, 159.59, 148.68, 138.49, 138.15, 136.97, 136.33, 132.16, 131.22, 130.66, 129.65, 128.60, 120.98, 117.34, 116.21, 79.48, 39.54, 25.47. **HRMS** (ESI-TOF) calculated for: $C_{26}H_{23}ClO_4$ $[M+H]^+$: 435.1358, found: 435.1357.

4-allylphenyl (3aS,4S,6R,6aR)-6-methoxy-2,2-dimethyltetrahydrofuro [3,4-d] [1,3] dioxole-4-carboxylate **sub. 2s**

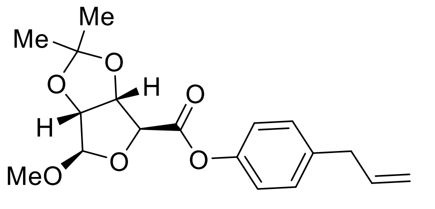

R_f = 0.6, PE/EA = 5:1 (v/v). [1]**H NMR** (400 MHz, Chloroform-*d*) δ 7.23-7.21 (m, 2H), 7.06-7.03 (m, 2H), 5.97 (ddt, *J* = 18.3, 9.4, 6.7 Hz, 1H), 5.38 (dd, *J* = 5.9, 1.0 Hz, 1H), 5.13-5.09 (m, 3H), 4.87-4.86 (m, 1H), 4.66 (d, *J* = 5.8 Hz, 1H), 3.47 (s, 3H), 3.42-3.39 (m, 2H), 1.55 (s, 3H), 1.38 (s, 3H). [13]**C NMR** (101 MHz, Chloroform-*d*) δ 168.94, 148.72, 137.94, 137.04, 129.62, 121.05, 116.16, 112.88, 109.61, 84.34, 83.62, 82.23, 55.78, 39.57, 26.40, 25.05. **HRMS** (ESI-TOF) calculated for: $C_{18}H_{22}O_6$ $[M+H]^+$: 335.1489, found: 335.1487.

4-allylphenyl 4-(2-hydroxypropan-2-yl)benzoate **sub. 2t**

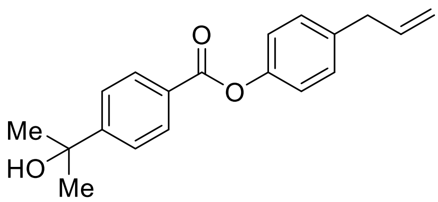

R_f = 0.5 PE/EA = 4:1 (v/v). **^{1}H NMR** (400 MHz, Chloroform-*d*) δ 8.20-8.19 (m, 2H), 7.66-7.64 (m, 2H), 7.27 (d, *J* = 8.4 Hz, 2H), 7.16 (d, *J* = 8.5 Hz, 2H), 6.01 (ddt, *J* = 16.9, 10.3, 6.7 Hz, 1H), 5.15-5.10 (m, 2H), 3.44 (dt, *J* = 6.6, 1.6 Hz, 2H), 1.83 (d, *J* = 3.9 Hz, 1H), 1.65 (s, 6H). **^{13}C NMR** (101 MHz, Chloroform-*d*) δ 165.15, 154.94, 149.26, 137.64, 137.20, 130.23, 129.58, 128.05, 124.67, 121.57, 116.06, 72.65, 39.62, 31.77. **HRMS** (ESI-TOF) calculated for: $C_{19}H_{20}O_3$ $[M+H]^+$: 297.1485, found: 297.1485.

2-(2-(4-allylphenyl)-2-methylpropyl)isoindoline-1,3-dione **sub. 2u**

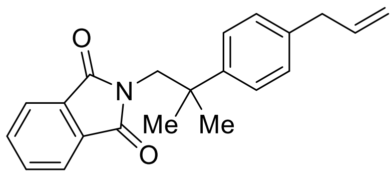

R_f= 0.2, PE/EA = 50:1 (v/v). **^{1}H NMR** (400 MHz, Chloroform-*d*) δ 7.86 (dd, *J* = 5.5, 3.1 Hz, 2H), 7.74 (dd, *J* = 5.4, 3.1 Hz, 2H), 7.44 (d, *J* = 8.2 Hz, 2H), 7.19 (d, *J* = 8.1 Hz, 2H), 6.00 (ddt, *J* = 16.9, 10.2, 6.7 Hz, 1H), 5.13-5.08 (m, 2H), 3.82 (s, 2H), 3.41 (d, *J* = 6.8 Hz, 2H), 1.41 (s, 6H). **^{13}C NMR** (101 MHz, Chloroform-*d*) δ 168.78, 144.52, 138.06, 137.49, 133.92, 132.04, 128.44, 126.20, 123.27, 115.77, 49.66, 40.18, 39.72, 26.88. **HRMS** (ESI-TOF) calculated for: $C_{21}H_{21}NO_2$ $[M+H]^+$: 320.1645, found: 320.1645.

5,5'-diallyl-[1,1'-biphenyl]-2,2'-diyl dibenzoate **sub. 2v**

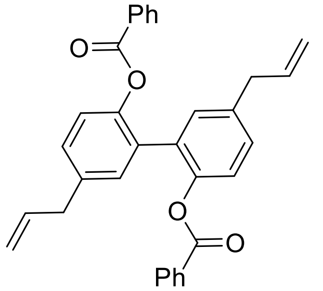

R_f= 0.4, PE/EA = 20:1 (v/v).**^{1}H NMR** (400 MHz, Chloroform-*d*) δ 8.04-8.01 (m, 4H), 7.61-7.57 (m, 2H), 7.44 (t, *J* = 7.8 Hz, 4H), 7.28-7.19 (m, 6H), 5.85 (ddt, *J* = 16.8, 10.1, 6.7 Hz, 2H), 5.04-4.98 (m, 4H), 3.37-3.35 (m, 4H). **^{13}C NMR** (101 MHz, Chloroform-*d*) δ 165.10, 146.60, 137.55, 136.89, 133.33, 131.27, 130.60, 130.26, 130.10, 129.52, 129.07, 128.91, 128.38, 122.55, 116.09, 39.50. **HRMS** (ESI-TOF) calculated for: $C_{32}H_{26}O_4$ $[M+H]^+$: 475.1904, found: 475.1904.

1-(3,4-dichlorophenyl)-4-vinyl-1,2,3,4-tetrahydronaphthalene **sub. 2y**

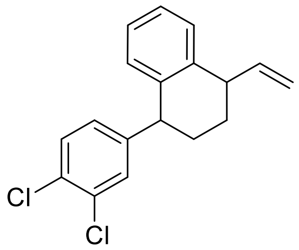

R_f= 0.8, PE/EA = 50:1 (v/v). **^{1}H NMR** (400 MHz, Chloroform-*d*) δ 7.37 (dd, *J* = 8.3, 3.3 Hz, 1H), 7.26-7.18 (m, 3H), 7.11 (qd, *J* = 7.0, 6.4, 1.7 Hz, 1H), 6.94 (ddd, *J* = 10.1, 8.2, 2.1 Hz, 1H), 6.84 (dd, *J* = 12.6, 7.7 Hz, 1H), 6.06-5.88 (m, 1H), 5.21-5.02 (m, 2H), 4.11 (q, *J* = 7.3, 6.8 Hz, 1H), 3.60 (dq, *J* = 13.0, 7.1 Hz, 1H), 2.25-2.06 (m, 1H), 1.96-1.72 (m, 3H). **^{13}C NMR** (101 MHz, Chloroform-*d*) δ 147.74, 143.01, 142.64, 138.70, 138.45, 138.04, 138.00, 132.27, 132.22, 130.71, 130.69, 130.26, 130.23, 130.10, 130.01, 129.97, 129.44, 128.25, 126.48, 126.45, 126.40, 115.77, 115.61, 45.19, 44.94, 43.95, 43.14, 31.01, 29.72, 27.93, 26.76. **HRMS** (ESI-TOF) calculated for: $C_{18}H_{16}Cl_2$ $[M+H]^+$: 303.0702, found: 303.0702.

1-ethyl-4-(4-methylpent-1-en-3-yl)benzene **sub. 2aa**

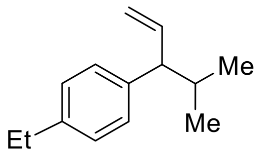

R_f= 0.9, PE. **^{1}H NMR** (400 MHz, Chloroform-*d*) δ 7.17-7.10 (m, 4H), 6.03 (ddd, *J* = 16.9, 10.3, 9.1 Hz, 1H), 5.08-5.03 (m, 2H), 2.90 (t, *J* = 8.9 Hz, 1H), 2.66 (q, *J* = 7.6 Hz, 2H), 1.97 (dp, *J* = 8.7, 6.6 Hz, 1H), 1.27 (t, *J* = 7.6 Hz, 3H), 1.00 (d, *J* = 6.7 Hz, 3H), 0.81 (d, *J* = 6.7 Hz, 3H). **^{13}C NMR** (101 MHz, Chloroform-*d*) δ 141.78, 141.54, 141.42, 127.82, 127.77, 114.73, 58.18, 32.62, 28.43, 21.09, 20.78, 15.54. **HRMS** (ESI-TOF) calculated for: $C_{14}H_{20}$ $[M+H]^+$: 189.1638, found: 189.1640.

4-(but-3-en-2-yl)-6-(tert-butyl)-1,1-dimethyl-2,3-dihydro-1H-indene **sub. 2ab**

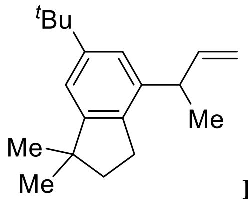

R_f= 0.9, PE. **^{1}H NMR** (400 MHz, Chloroform-*d*) δ 7.10-7.07 (m, 2H), 6.05 (ddd, *J* = 16.9, 10.3, 6.3 Hz, 1H), 5.12-5.05 (m, 2H), 3.62-3.56 (m, 1H), 2.87 (t, *J* = 7.2 Hz, 2H), 1.95 (t, *J* = 7.2 Hz, 2H), 1.40 (d, *J* = 7.0 Hz, 3H), 1.36 (s, 9H), 1.30 (d, *J* = 5.7 Hz, 6H). **^{13}C NMR** (101 MHz, Chloroform-*d*) δ 152.38, 149.94, 142.75, 140.26, 137.96, 121.02, 116.72, 112.86, 44.07, 41.37, 40.60, 34.85, 31.72, 28.85, 27.91, 19.69. **HRMS** (ESI-TOF) calculated for: $C_{19}H_{28}$ $[M+H]^+$: 257.2264, found: 257.2264.

6-(but-3-en-2-yl)-1,1,2,4,4,7-hexamethyl-1,2,3,4-tetrahydronaphthalene **sub. 2ac**

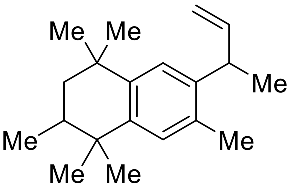

R_f = 0.9, PE. ^{1}H NMR (400 MHz, Chloroform-*d*) δ 7.19-7.08 (m, 2H), 6.09-5.97 (m, 1H), 5.07 (ddd, *J* = 15.6, 3.8, 1.7 Hz, 2H), 3.66 (p, *J* = 7.0 Hz, 1H), 2.33 (s, 3H), 1.94 -1.86 (m, 1H), 1.66 (t, *J* = 13.2 Hz, 1H), 1.40-1.27 (m, 13H), 1.09 (d, *J* = 3.2 Hz, 3H), 1.01 (d, *J* = 6.7 Hz, 3H). **^{13}C NMR** (101 MHz, Chloroform-*d*) δ 143.44, 143.21, 143.11, 142.36, 142.34, 140.36, 140.32, 132.62, 128.58, 124.13, 124.09, 112.80, 43.89, 38.62, 38.60, 37.31, 34.66, 34.64, 34.19, 32.49, 32.46, 32.14, 28.61, 25.06, 25.03, 20.01, 19.94, 19.16, 16.87. **HRMS** (ESI-TOF) calculated for: $C_{20}H_{30}$ [M+H]$^+$: 271.2420, found: 271.2421.

4-(1-(p-tolyl)allyl)tetrahydro-2H-pyran **sub. 2ad**

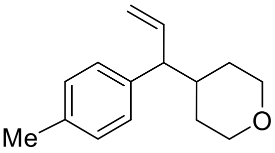

R_f = 0.4, PE/EA = 50:1 (v/v). **^{1}H NMR** (400 MHz, Chloroform-*d*) δ 7.15 (d, *J* = 7.8 Hz, 2H), 7.09-7.07 (m, 2H), 5.98 (ddd, *J* = 17.3, 10.1, 8.7 Hz, 1H), 5.10-5.05 (m, 2H), 4.05-4.01 (m, 1H), 3.93-3.88 (m, 1H), 3.35 (dtd, *J* = 36.6, 11.7, 2.2 Hz, 2H), 2.94 (t, *J* = 9.2 Hz, 1H), 2.35 (s, 3H), 1.85-1.77 (m, 2H), 1.39-1.19 (m, 3H). **^{13}C NMR** (101 MHz, Chloroform-*d*) δ 140.34, 139.95, 135.80, 129.26, 127.73, 115.45, 68.13, 68.11, 56.90, 39.54, 31.65, 31.45, 21.03. **HRMS** (ESI-TOF) calculated for: $C_{15}H_{20}O$ [M+H]$^+$: 217.1587, found: 217.1587.

(E)-1-(tert-butyl)-4-(3-(4-fluorophenyl)prop-1-en-1-yl)benzene **sub. 2ah**

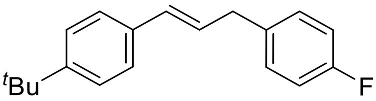

R_f = 0.7, PE. **^{1}H NMR** (400 MHz, Chloroform-*d*) δ 7.38-7.32 (m, 4H), 7.22 (dd, *J* = 8.5, 5.6 Hz, 2H), 7.04-7.00 (m, 2H), 6.49-6.48 (m, 1H), 6.32 (dt, *J* = 15.8, 6.8 Hz, 1H), 3.54 (d, *J* = 6.8 Hz, 2H), 1.35 (s, 9H). **^{13}C NMR** (101 MHz, Chloroform-*d*) δ 161.50 (d, *J* = 243.8 Hz), 150.32, 135.94 (d, *J* = 3.3 Hz), 134.58, 131.02, 130.02 (d, *J* = 7.9 Hz), 128.18, 125.67 (d, *J* = 38.6 Hz), 115.30, 115.08, 38.51, 34.55, 31.32. **^{19}F NMR** (376 MHz, Chloroform-*d*) δ -117.35. **HRMS** (ESI-TOF) calculated for: $C_{19}H_{21}F$ [M+H]$^+$: 269.1700, found: 269.1700.

1-methyl-4-(5-methylhex-2-en-1-yl)benzene **sub. 3a**

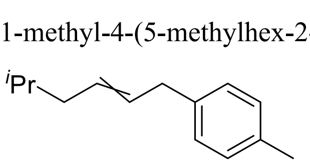

R_f = 0.9, PE. **^{1}H NMR** (400 MHz, Chloroform-*d*) δ 7.15-7.14 (m, 8H), 5.68-5.52 (m, 4H), 3.41 (t, *J* = 6.0 Hz, 2H), 3.37-3.34 (m, 2H), 2.38-2.36 (m, 6H), 2.10 (q, *J* = 6.5 Hz, 2H), 1.96 (q, *J* = 6.3 Hz, 2H), 1.77-1.67 (m, 2H), 1.01-0.93 (m, 12H). **^{13}C NMR** (101 MHz, Chloroform-*d*) δ 138.23, 138.09, 135.30, 135.27, 130.56, 130.19, 129.47, 129.11, 129.04, 129.02, 128.39, 128.26, 41.95, 38.74, 36.40, 33.17, 28.78, 28.50, 22.47, 22.36, 21.02. **HRMS** (ESI-TOF) calculated for: $C_{14}H_{20}$ [M+H]$^+$: 189.1638, found: 189.1638.

ethyl (E)-6-(p-tolyl)hex-4-enoate **sub. 3b**

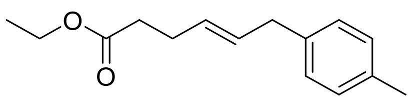

R_f = 0.5, PE/EA = 30:1 (v/v). **^{1}H NMR** (400 MHz, Chloroform-*d*) δ 7.14-7.09 (m, 4H), 5.67-5.59 (m, 1H), 5.53-5.47 (m, 1H), 4.17 (q, *J* = 7.1 Hz, 2H), 3.41 (d, *J* = 7.3 Hz, 2H), 2.51 (p, *J* = 6.5, 5.6 Hz, 2H), 2.44-2.37 (m, 2H), 2.34 (s, 3H), 1.29 (t, *J* = 7.2 Hz, 3H). **^{13}C NMR** (101 MHz, Chloroform-*d*) δ 173.12, 137.72, 135.39, 129.90, 129.14, 128.27, 128.20, 60.37, 34.34, 33.02, 22.85, 20.98, 14.26. **HRMS** (ESI-TOF) calculated for: $C_{15}H_{20}O_2$ $[M+H]^+$: 233.1536, found: 233.1536.

1-(7-chlorohept-2-en-1-yl)-4-methylbenzene **sub. 3c**

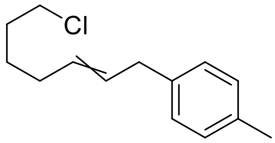

R_f= 0.6, PE. **^{1}H NMR** (400 MHz, Chloroform-*d*) δ 7.17-7.09 (m, 8H), 5.66 -5.48 (m, 4H), 3.58 (q, *J* = 6.7 Hz, 4H), 3.40 (d, *J* = 7.3 Hz, 2H), 3.33 (d, *J* = 6.5 Hz, 2H), 2.37-2.36 (m, 6H), 2.25-2.05 (m, 4H), 1.89-1.80 (m, 4H), 1.63-1.54 (m, 4H). **^{13}C NMR** (101 MHz, Chloroform-*d*) δ 137.92, 137.82, 135.41, 130.84, 129.90, 129.83, 129.17, 129.10, 129.07, 128.39, 128.21, 125.49, 45.04, 38.64, 33.08, 32.20, 32.10, 31.73, 26.91, 26.67, 26.46, 21.04. **HRMS** (ESI-TOF) calculated for: $C_{14}H_{19}Cl$ $[M+H]^+$: 223.1248, found: 223.1248.

2-(5-(p-tolyl)pent-3-en-1-yl)isoindoline-1,3-dione **sub. 3d**

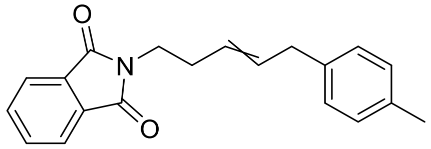

R_f = 0.2, PE/EA = 30:1 (v/v). **^{1}H NMR** (400 MHz, Chloroform-*d*) δ 7.87-7.82 (m, 4H), 7.75-7.71 (m, 4H), 7.01-6.97 (m, 8H), 5.72-5.49 (m, 4H), 3.82-3.75 (m, 4H), 3.35-3.24 (m, 4H), 2.60 (qd, *J* = 7.1, 1.4 Hz, 2H), 2.47-2.41 (m, 2H), 2.30-2.28 (m, 6H). **^{13}C NMR** (101 MHz, Chloroform-*d*) δ 168.42, 168.38, 137.46, 137.29, 135.31, 135.26, 133.88, 133.83, 132.64, 132.09, 131.62, 129.06, 129.00, 128.28, 128.08, 126.98, 125.78, 123.23, 38.58, 37.67, 37.57, 32.89, 31.65, 26.54, 21.00. **HRMS** (ESI-TOF) calculated for: $C_{20}H_{19}NO_2$ $[M+H]^+$: 306.1489, found: 306.1489.

1-bromo-4-(5-(p-tolyl)pent-3-en-1-yl)benzene **sub. 3e**

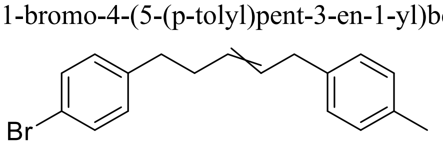

R_f= 0.7, PE. **^{1}H NMR** (400 MHz, Chloroform-*d*) δ 7.47-7.43 (m, 4H), 7.17-7.00 (m, 12H), 5.66-5.51 (m, 4H), 3.35-3.32 (m, 4H), 2.75-2.70 (m, 4H), 2.54-2.37 (m, 10H). **^{13}C NMR** (101 MHz, Chloroform-*d*) δ 140.93, 140.87, 137.75, 137.70, 135.45, 135.40, 131.40, 131.34, 130.37, 130.25, 129.53, 129.16, 129.10, 129.03, 128.94, 128.38, 128.21, 119.67, 119.52, 38.59, 35.32, 34.15, 33.08, 29.02, 21.06. **HRMS** (ESI-TOF) calculated for: $C_{18}H_{19}Br$ $[M+H]^+$: 315.0743, found: 315.0743.

1-(tert-butyl)-4-(2-methyl-5-(p-tolyl)pent-3-en-1-yl)benzene **sub. 3f**

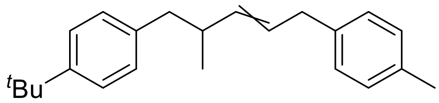

R_f = 0.9, PE. **¹H NMR** (400 MHz, Chloroform-*d*) δ 7.37-7.35 (m, 4H), 7.19-6.96 (m, 12H), 5.55-5.37 (m, 4H), 3.35-3.17 (m, 4H), 2.96-2.90 (m, 2H), 2.66-2.64 (m, 4H), 2.39-2.36 (m, 6H), 1.40-1.39 (m, 18H), 1.12-1.09 (m, 6H). **¹³C NMR** (101 MHz, Chloroform-*d*) δ 148.58, 148.46, 138.08, 137.99, 137.83, 137.78, 137.15, 136.07, 135.30, 135.23, 129.07, 129.03, 128.98, 128.44, 128.27, 127.51, 127.42, 125.03, 124.97, 43.37, 43.19, 38.66, 38.39, 34.41, 34.05, 33.22, 31.53, 21.10, 21.04, 20.12. **HRMS** (ESI-TOF) calculated for: $C_{23}H_{30}$ $[M+H]^+$: 307.2420, found: 307.2422.

1-(2-cyclohexylideneethyl)-4-methylbenzene **sub. 3g**

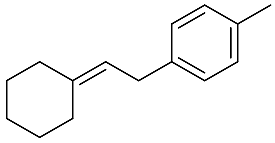
R_f = 0.9, PE. **¹H NMR** (400 MHz, Chloroform-*d*) δ 7.14-7.10 (m, 4H), 5.29 (tt, *J* = 7.5, 1.3 Hz, 1H), 3.35 (d, *J* = 7.5 Hz, 2H), 2.35 (s, 3H), 2.28 (t, *J* = 5.2 Hz, 2H), 2.15 (t, *J* = 5.3 Hz, 2H), 1.61-1.57 (m, 6H). **¹³C NMR** (101 MHz, Chloroform-*d*) δ 140.36, 138.93, 135.11, 129.05, 128.24, 120.07, 37.22, 32.99, 28.77, 28.65, 27.91, 26.98, 21.01. **HRMS** (ESI-TOF) calculated for: $C_{15}H_{20}$ $[M+H]^+$: 201.1638, found: 201.1639.

(2-(p-tolyl)ethylidene)cyclododecane **sub. 3h**

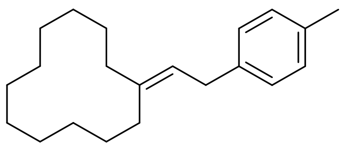
R_f = 0.9, PE. **¹H NMR** (400 MHz, Chloroform-*d*) δ 7.14-7.10 (m, 4H), 5.41 (t, *J* = 7.3 Hz, 1H), 3.39 (d, *J* = 7.2 Hz, 2H), 2.35 (s, 3H), 2.19 (t, *J* = 6.8 Hz, 2H), 2.10 (dt, *J* = 7.2, 4.1 Hz, 2H), 1.59-1.51 (m, 4H), 1.40-1.35 (m, 14H). **¹³C NMR** (101 MHz, Chloroform-*d*) δ 139.01, 138.58, 135.11, 129.05, 128.28, 123.90, 33.77, 31.80, 28.57, 24.97, 24.75, 24.39, 24.24, 24.12, 24.08, 23.54, 23.15, 22.29, 21.03. **HRMS** (ESI-TOF) calculated for: $C_{21}H_{32}$ $[M+H]^+$: 285.2577, found: 285.2578.

(E)-4-(4-(p-tolyl)but-2-en-2-yl)benzonitrile **sub. 3i**

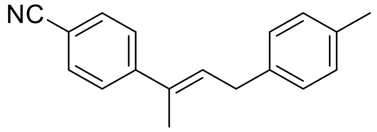

R_f = 0.6, PE/EA = 30:1 (v/v). **¹H NMR** (400 MHz, Chloroform-*d*) δ 7.68-7.65 (m, 2H), 7.37-7.35 (m, 2H), 7.14-7.12 (d, *J* = 7.8 Hz, 2H), 7.05-7.03 (m, 2H), 5.78 (td, *J* = 7.7, 1.6 Hz, 1H), 3.27 (d, *J* = 7.6 Hz, 2H), 2.35 (s, 3H), 2.11-2.10 (m, 3H). **¹³C NMR** (101 MHz, Chloroform-*d*) δ 146.78, 137.52, 135.66, 135.60, 132.13, 129.25, 128.82, 128.05, 127.97, 118.99, 110.51, 34.80, 25.11, 21.01. **HRMS** (ESI-TOF) calculated for: $C_{18}H_{17}N$ $[M+H]^+$: 248.1434, found: 248.1435.

4-(2-(p-tolyl)ethylidene)cyclohexan-1-one **sub. 3j**

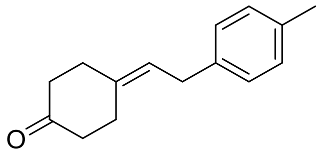

R_f = 0.2, PE/EA = 5:1 (v/v). **^{1}H NMR** (400 MHz, Chloroform-*d*) δ 7.15-7.10 (m, 4H), 5.58 (tt, *J* = 7.5, 1.5 Hz, 1H), 3.40 (d, *J* = 7.5 Hz, 2H), 2.67-2.63 (m, 2H), 2.56-2.44 (m, 6H), 2.35 (s, 3H). **^{13}C NMR** (101 MHz, Chloroform-*d*) δ 211.67, 137.80, 135.58, 134.69, 129.25, 128.16, 124.29, 41.78, 40.86, 34.23, 33.59, 26.25, 21.03. **HRMS** (ESI-TOF) calculated for: $C_{15}H_{18}O$ $[M+H]^+$: 215.1430, found: 215.1431.

5-methyl-7-(p-tolyl)hept-5-enenitrile **sub. 3k**

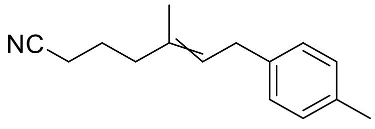

R_f= 0.2, PE/EA = 5:1 (v/v). **^{1}H NMR** (400 MHz, Chloroform-*d*) δ 7.14-7.08 (m, 8H), 5.50-5.41 (m, 2H), 3.37-3.35 (m, 4H), 2.37-2.30 (m, 12H), 2.23-2.19 (m, 2H), 1.86-1.72 (m, 10H). **^{13}C NMR** (101 MHz, Chloroform-*d*) δ 138.19, 138.11, 135.43, 135.37, 133.43, 133.34, 129.18, 129.17, 128.15, 126.22, 125.59, 119.78, 119.76, 38.28, 33.82, 33.62, 30.52, 23.74, 23.50, 23.15, 21.02, 16.74, 16.42, 15.86. **HRMS** (ESI-TOF) calculated for: $C_{15}H_{19}N$ $[M+H]^+$:214.1590, found: 214.1590.

4-(2-(p-tolyl)ethylidene)tetrahydro-2H-pyran **sub. 3l**

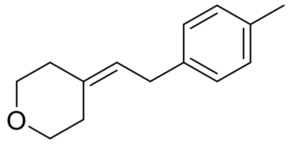

R_f= 0.4, PE/EA = 100:1 (v/v). **^{1}H NMR** (400 MHz, Chloroform-*d*) δ 7.16-7.10 (m, 4H), 5.44-5.40 (m, 1H), 3.75 (t, *J* = 5.5 Hz, 4H), 3.38 (d, *J* = 7.5 Hz, 2H), 2.44-2.41 (m, 2H), 2.37 (s, 3H), 2.29 (t, *J* = 5.4 Hz, 2H). **^{13}C NMR** (101 MHz, Chloroform-*d*) δ 138.23, 135.40, 134.98, 129.18, 128.20, 122.12, 69.67, 68.83, 37.01, 32.78, 29.80, 21.02. **HRMS** (ESI-TOF) calculated for: $C_{14}H_{18}O$ $[M+H]^+$: 203.1430, found: 203.1430.

1-methyl-4-(3-methyldodec-2-en-1-yl)benzene **sub. 3m**

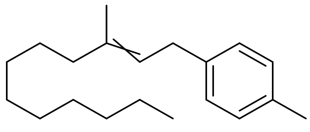

R_f= 0.9, PE. **^{1}H NMR** (400 MHz, Chloroform-*d*) δ 7.13 (m, 8H), 5.38 -5.33 (m, 2H), 3.37-3.35 (m, 4H), 2.36 (m, 6H), 2.16 (dd, *J* = 8.7, 6.6 Hz, 2H), 2.06 (t, *J* = 7.6 Hz, 2H), 1.77-1.74 (m, 6H), 1.48-1.44 (m, 4H), 1.38-1.31 (m, 24H), 0.96-0.92 (m, 6H). **^{13}C NMR** (101 MHz, Chloroform-*d*) δ 138.86, 136.50, 136.39, 135.13, 135.10, 129.06, 129.05, 128.24, 128.23, 123.75, 123.04, 39.75, 33.80, 33.69, 31.97, 31.88, 29.74, 29.68, 29.62, 29.40, 29.38, 28.16, 28.00, 23.53, 22.74, 21.02, 16.09, 14.18. **HRMS** (ESI-TOF) calculated for: $C_{20}H_{32}$ $[M+H]^+$:273.2577, found: 273.2574.

(S)-1-(5,9-dimethyldec-2-en-1-yl)-4-methylbenzene **sub. 3n**

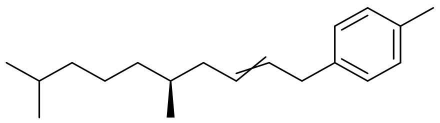

R_f = 0.9, PE. **^{1}H NMR** (400 MHz, Chloroform-*d*) δ 7.17-7.13 (m, 8H), 5.68-5.53 (m, 4H), 3.42 (d, *J* = 7.0 Hz, 2H), 3.36 (d, *J* = 6.1 Hz, 2H), 2.34 (m, 6H), 2.24-2.01 (m, 4H), 1.62-1.53 (m, 4H), 1.41-1.28 (m, 6H), 1.24-1.15 (m, 6H), 0.98-0.92 (m, 18H). **^{13}C NMR** (101 MHz, Chloroform-*d*) δ 138.25, 138.11, 135.30, 135.27, 130.46, 130.23, 129.45, 129.11, 129.06, 128.40, 128.27, 40.08, 39.34, 38.78, 37.01, 36.86, 34.59, 33.53, 33.20, 28.03, 28.00, 24.96, 24.85, 22.77, 22.68, 21.04, 19.73, 19.63. **HRMS** (ESI-TOF) calculated for: $C_{19}H_{30}$ $[M+H]^+$: 259.2420, found: 259.2423.

1-(2-(4,4-difluorocyclohexylidene)ethyl)-4-methylbenzene **sub. 3o**

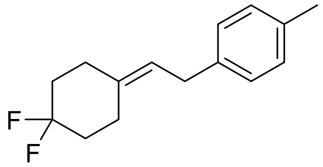

R_f = 0.7, PE. **^{1}H NMR** (400 MHz, Chloroform-*d*) δ 7.14 (d, *J* = 7.8 Hz, 2H), 7.09 (d, *J* = 8.1 Hz, 2H), 5.48-5.44 (m, 1H), 3.37 (d, *J* = 7.5 Hz, 2H), 2.46 (t, *J* = 6.7 Hz, 2H), 2.36-2.32 (m, 5H), 2.04-1.94 (m, 4H). **^{13}C NMR** (101 MHz, Chloroform-*d*) δ 137.93, 135.51, 135.04, 129.22, 128.14, 123.61, 123.56 (t, *J* = 240.9 Hz), 35.13 (t, *J* = 23.4 Hz), 34.45 (t, *J* = 23.8 Hz), 33.37, 32.21 (t, *J* = 5.3 Hz), 23.88 (t, *J* = 5.4 Hz), 21.00. **^{19}F NMR** (376 MHz, Chloroform-*d*) δ -97.23. **HRMS** (ESI-TOF) calculated for: $C_{15}H_{18}F_2$ $[M+H]^+$: 237.1449, found: 237.1454.

(3aS,4R,7R,7aS)-5-(2-(p-tolyl)ethylidene)octahydro-1H-4,7-methanoindene **sub. 3p**

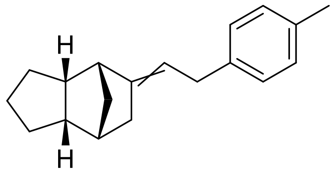

R_f = 0.9, PE. **^{1}H NMR** (400 MHz, Chloroform-*d*) δ 7.13-7.11 (m, 8H), 5.43-5.17 (m, 2H), 3.40 (d, *J* = 7.3 Hz, 2H), 3.26 (d, *J* = 7.3 Hz, 2H), 2.84 (s, 1H), 2.45 (s, 1H), 2.36-2.35 (m, 6H), 2.23-2.10 (m, 4H), 2.00-1.81 (m, 10H), 1.74-1.68 (m, 2H), 1.55-1.47 (m, 2H), 1.28-1.15 (m, 4H), 1.07-0.97 (m, 4H). **^{13}C NMR** (101 MHz, Chloroform-*d*) δ 146.60, 145.70, 139.09, 138.84, 135.09, 129.06, 129.03, 128.22, 128.14, 116.70, 116.01, 53.45, 49.49, 47.99, 47.68, 47.56, 47.26, 44.36, 40.95, 40.85, 37.85, 35.37, 35.10, 34.60, 32.81, 32.76, 32.48, 32.46, 31.84, 31.76, 27.70, 27.66, 21.03. **HRMS** (ESI-TOF) calculated for: $C_{19}H_{24}$ $[M+H]^+$: 253.1951, found: 253.1951.

7-(p-tolyl)hept-5-en-1-yl (S)-3,3,3-trifluoro-2-methoxy-2-phenylpropanoate **sub. 3q**

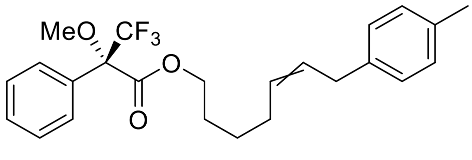

R_f = 0.7, PE/EA = 30:1 (v/v). **^{1}H NMR (**400 MHz, Chloroform-*d*) δ 7.57-7.54 (m, 4H), 7.44-7.41 (m, 6H), 7.26-7.24 (m, 4H), 7.14-7.12 (m, 4H), 6.38-6.34 (m, 2H), 6.19-6.11 (m, 2H), 4.41-4.30 (m, 4H), 3.58 (m, 6H), 2.36 (m, 6H), 2.23-2.18 (m, 4H),

1.79-1.72 (m, 4H), 1.54-1.37 (m, 8H). **^{13}C NMR** (101 MHz, Chloroform-*d*) δ 166.63, 136.60, 134.96, 132.40, 129.97, 129.61, 129.38, 129.20, 128.42, 127.32, 125.83, 123.35 (q, *J* = 284.0 Hz), 84.61 (q, *J* = 27.8 Hz), 66.46, 55.47, 55.45, 32.77, 28.84, 28.24, 25.31, 21.15. **^{19}F NMR** (376 MHz, Chloroform-*d*) δ -71.58. **HRMS** (ESI-TOF) calculated for: $C_{24}H_{27}F_3O_3$ $[M+H]^+$:421.1985, found: 421.1985.

(E)-7-(p-tolyl)hept-5-en-1-yl 4-(N,N-dipropylsulfamoyl)benzoate **sub. 3r**

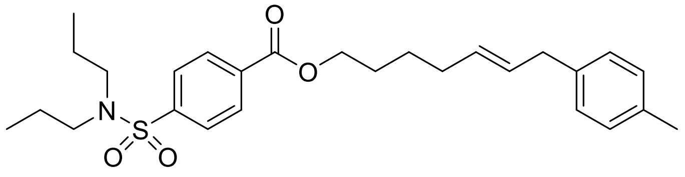

R_f = 0.3, PE/EA = 30:1 (v/v). **^{1}H NMR** (400 MHz, Chloroform-*d*) δ 8.17-8.15 (m, 2H), 7.88-7.86 (m, 2H), 7.25 (d, *J* = 8.1 Hz, 2H), 7.12 (d, *J* = 7.9 Hz, 2H), 6.39 (dt, *J* = 15.7, 1.5 Hz, 1H), 6.18 (dt, *J* = 15.8, 6.9 Hz, 1H), 4.38 (t, *J* = 6.6 Hz, 2H), 3.13-3.10 (m, 4H), 2.35 (s, 3H), 2.25 (dd, *J* = 7.0, 1.4 Hz, 2H), 1.84 (p, *J* = 6.7 Hz, 2H), 1.57 (m, 9H), 0.89 (t, *J* = 7.4 Hz, 6H). **^{13}C NMR** (101 MHz, Chloroform-*d*) δ 165.33, 144.16, 136.66, 134.95, 133.76, 130.17, 130.02, 129.43, 129.22, 127.00, 125.81, 65.67, 49.94, 32.76, 28.93, 28.49, 25.48, 21.95, 21.14, 11.17. **HRMS** (ESI-TOF) calculated for: $C_{27}H_{37}NO_4S$ $[M+H]^+$: 472.2516, found: 472.2516.

1-methyl-4-(trideca-2,12-dien-1-yl)benzene **sub. 3s**

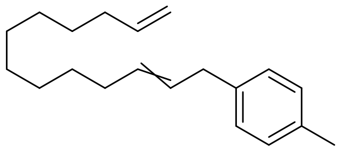

R_f= 0.8, PE. **^{1}H NMR** (400 MHz, Chloroform-*d*) δ 7.15-7.10 (m, 8H), 5.91-5.81 (m, 2H), 5.61-5.49 (m, 4H), 5.06-4.95 (m, 4H), 3.40 (d, *J* = 6.3 Hz, 2H), 3.32 (d, *J* = 5.7 Hz, 2H), 2.36 (m, 6H), 2.20-2.02 (m, 8H), 1.44-1.30 (m, 24H). **^{13}C NMR** (101 MHz, Chloroform-*d*) δ 139.29, 138.21, 138.09, 135.32, 135.28, 131.90, 130.83, 129.11, 129.05, 129.00, 128.38, 128.27, 128.24, 114.15, 38.68, 33.87, 33.08, 32.56, 29.74, 29.54, 29.52, 29.48, 29.36, 29.22, 29.18, 28.98, 27.26, 21.03. **HRMS** (ESI-TOF) calculated for: $C_{20}H_{30}$ $[M+H]^+$: 271.2420, found: 271.2422.

(E)-7-(p-tolyl)hept-5-en-1-yl 2-(11-oxo-6,11-dihydrodibenzo[b,e]oxepin-9-yl)acetate **sub. 3t**

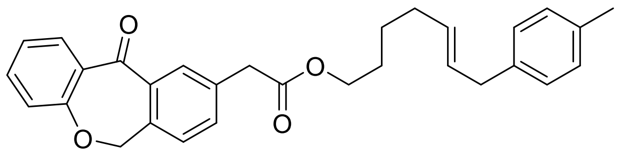

R_f= 0.2, PE/EA = 30:1 (v/v). **^{1}H NMR** (400 MHz, Chloroform-*d*) δ 8.15 (d, *J* = 2.4 Hz, 1H), 7.92 (dd, *J* = 7.7, 1.4 Hz, 1H), 7.58 (td, *J* = 7.4, 1.4 Hz, 1H), 7.51-7.43 (m, 2H), 7.38 (dd, *J* = 7.5, 1.3 Hz, 1H), 7.26-7.24 (m, 2H), 7.12 (d, *J* = 7.9 Hz, 2H), 7.05 (d, *J* = 8.5 Hz, 1H), 6.39-6.34 (m, 1H), 6.16 (dt, *J* = 15.8, 6.9 Hz, 1H), 5.20 (s, 2H), 4.13 (t, *J* = 6.7 Hz, 2H), 3.66 (s, 2H), 2.34 (s, 3H), 2.21 (qd, *J* = 7.1, 1.4 Hz, 2H), 1.68 (p, *J* = 6.8 Hz, 2H), 1.52-1.38 (m, 4H). **^{13}C NMR** (101 MHz, Chloroform-*d*) δ 190.85, 171.51, 160.46, 140.49, 136.54, 136.37, 135.58, 135.03, 132.77, 132.47, 129.86, 129.60, 129.51, 129.28, 129.19, 128.00, 127.82, 125.83, 125.15, 121.03, 73.65, 65.06, 40.31, 32.85, 28.98, 28.46, 25.47, 21.15.

HRMS (ESI-TOF) calculated for: $C_{30}H_{30}O_4$ $[M+H]^+$: 455.2217, found: 455.2217.

1-(tert-butyl)-4-(2,4-dimethylpent-3-en-2-yl)benzene **sub. 4c**

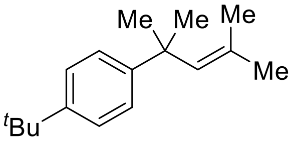

R_f= 0.9, PE. **^{1}H NMR** (400 MHz, Chloroform-*d*) δ 7.31-7.28 (m, 4H), 5.52 (p, *J* = 1.5 Hz, 1H), 1.73 (d, *J* = 1.5 Hz, 3H), 1.43 (s, 6H), 1.35 (s, 9H), 1.23 (d, *J* = 1.4 Hz, 3H). **^{13}C NMR** (101 MHz, Chloroform-*d*) δ 147.83, 147.68, 135.02, 132.29, 125.79, 124.72, 39.05, 34.23, 31.59, 31.45, 27.10, 18.96. **HRMS** (ESI-TOF) calculated for: $C_{17}H_{26}$ $[M+Na]^+$: 253.1927, found: 253.1927.

(2,4-dimethylpent-3-en-2-yl)benzene **sub. 4d**

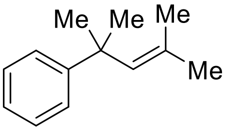

R_f= 0.9, PE. **^{1}H NMR** (400 MHz, Chloroform-*d*) δ 7.41-7.39 (m, 2H), 7.32-7.28 (m, 2H), 7.20-7.15 (m, 1H), 5.54 (p, *J* = 1.4 Hz, 1H), 1.73 (d, *J* = 1.5 Hz, 3H), 1.44 (s, 6H), 1.20 (d, *J* = 1.3 Hz, 3H). **^{13}C NMR** (101 MHz, Chloroform-*d*) δ 151.08, 134.94, 132.56, 127.95, 126.23, 125.08, 39.45, 31.61, 26.98, 18.98. **HRMS** (ESI-TOF) calculated for: $C_{13}H_{18}$ $[M+H]^+$: 175.1481, found: 175.1483.

1-bromo-4-(2,4-dimethylpent-3-en-2-yl)benzene **sub. 4e**

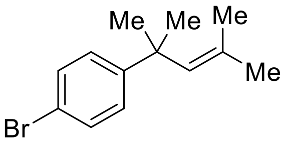

R_f= 0.7, PE. **^{1}H NMR** (400 MHz, Chloroform-*d*) δ 7.40 (d, *J* = 8.6 Hz, 2H), 7.28-7.25 (m, 2H), 5.50 (p, *J* = 1.5 Hz, 1H), 1.71 (d, *J* = 1.5 Hz, 3H), 1.39 (s, 6H), 1.18 (d, *J* = 1.4 Hz, 3H). **^{13}C NMR** (101 MHz, Chloroform-*d*) δ 150.22, 134.37, 133.09, 130.99, 128.15, 118.88, 39.23, 31.52, 26.90, 19.13. **HRMS** (ESI-TOF) calculated for: $C_{13}H_{17}Br$ $[M+NH_4]^+$: 270.0852, found: 270.0852.

1-(tert-butyl)-4-(4-methylpent-3-en-1-yl)benzene **sub. 4f**

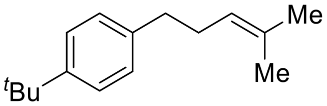

R_f= 0.9, PE. **^{1}H NMR (**400 MHz, Chloroform-*d*) δ 7.35-7.33 (m, 2H), 7.18-7.16 (m, 2H), 5.23 (ddq, *J* = 8.4, 5.7, 1.3 Hz, 1H), 2.64 (dd, *J* = 9.4, 6.7 Hz, 2H), 2.35-2.29 (m, 2H), 1.73 (t, *J* = 1.3 Hz, 3H), 1.62 (s, 3H), 1.35 (s, 9H). **^{13}C NMR** (101 MHz, Chloroform-*d*) δ 148.46, 139.38, 131.99, 128.05, 125.13, 124.00, 35.61, 34.35, 31.44, 30.06, 25.70, 17.68. **HRMS** (ESI-TOF) calculated for: $C_{16}H_{24}$ $[M+Na]^+$: 239.1770, found: 239.1770.

1-(2,4-dimethylpent-3-en-1-yl)-4-isopropylbenzene **sub. 4g**

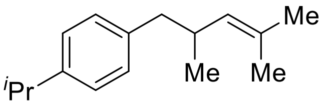

R$_f$= 0.9, PE. **^{1}H NMR** (400 MHz, Chloroform-*d*) δ 7.15 (d, *J* = 8.2 Hz, 2H), 7.09 (d, *J* = 8.1 Hz, 2H), 5.01 (dt, *J* = 8.9, 1.4 Hz, 1H), 2.90 (p, *J* = 6.9 Hz, 1H), 2.66-2.43 (m, 3H), 1.69 (d, *J* = 1.4 Hz, 3H), 1.49 (d, *J* = 1.4 Hz, 3H), 1.27 (d, *J* = 7.0 Hz, 6H), 0.95 (d, *J* = 6.4 Hz, 3H). **^{13}C NMR** (101 MHz, Chloroform-*d*) δ 146.08, 138.49, 130.71, 130.23, 129.15, 125.97, 43.57, 34.56, 33.70, 25.72, 24.10, 20.61, 17.74. **HRMS** (ESI-TOF) calculated for: $C_{16}H_{24}$ [M+H]$^+$: 217.1951, found: 217.1952.

1-methyl-3-(2,2,4-trimethylpent-3-en-1-yl)benzene **sub. 4h**

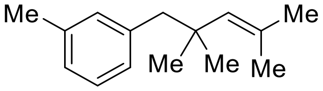

R$_f$= 0.9, PE. **^{1}H NMR** (400 MHz, Chloroform-*d*) δ 7.16 (td, *J* = 7.3, 1.1 Hz, 1H), 7.05-7.03 (m, 1H), 6.96 (m, 2H), 5.14-5.13 (m, 1H), 2.65 (s, 2H), 2.35 (s, 3H), 1.71 (dd, *J* = 15.7, 1.4 Hz, 6H), 1.11 (s, 6H). **^{13}C NMR (**101 MHz, Chloroform-*d*) δ 139.50, 136.87, 133.53, 131.54, 130.93, 127.68, 127.35, 126.41, 49.12, 36.43, 28.96, 28.20, 21.46, 19.03. **HRMS** (ESI-TOF) calculated for: $C_{15}H_{22}$ [M+H]$^+$: 203.1794, found: 203.1795

1-methyl-4-(1-(1-methylcyclohexyl)allyl)benzene **sub. 5e**

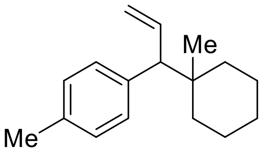

R$_f$= 0.9, PE. **^{1}H NMR** (400 MHz, Chloroform-*d*) δ 7.12-7.08 (m, 4H), 6.28 (dt, *J* = 16.8, 10.0 Hz, 1H), 5.10-5.03 (m, 2H), 3.13 (d, *J* = 9.9 Hz, 1H), 2.34 (s, 3H), 1.57-1.52 (m, 3H), 1.44-1.25 (m, 6H), 1.14-1.10 (m, 1H), 0.91 (s, 3H). **^{13}C NMR** (101 MHz, Chloroform-*d*) δ 139.32, 138.62, 135.38, 129.35, 128.43, 116.04, 36.19, 36.14, 36.06, 26.32, 21.96, 21.90, 20.99, 20.73. **HRMS** (ESI-TOF) calculated for: $C_{17}H_{24}$ [M+Na]$^+$: 251.1770, found:251.1771.

1-methyl-4-(non-2-en-1-yl)benzene **sub. 5e**

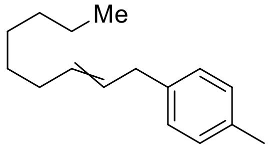

R$_f$= 0.9, PE. **^{1}H NMR** (400 MHz, Chloroform-*d*) δ 7.18-7.13 (m, 8H), 5.64-5.53 (m, 4H), 3.43 (d, *J* = 6.2 Hz, 2H), 3.35 (d, *J* = 5.7 Hz, 2H), 2.38 (m, 6H), 2.24-2.06 (m, 4H), 1.47-1.34 (m, 16H), 0.98-0.95 (m, 6H). **^{13}C NMR** (101 MHz, Chloroform-*d*) δ 138.24, 138.12, 135.33, 135.29, 131.95, 130.86, 129.13, 129.07, 129.01, 128.41, 128.29, 128.27, 38.71, 33.11, 32.60, 31.85, 31.81, 29.76, 29.53, 29.10, 28.96, 27.31, 22.74, 22.72, 21.04, 14.18. **HRMS** (ESI-TOF) calculated for: $C_{16}H_{24}$ [M+H]$^+$: 217.1951, found: 217.1953.

1-methyl-4-(undec-2-en-1-yl)benzene **sub. 5f**

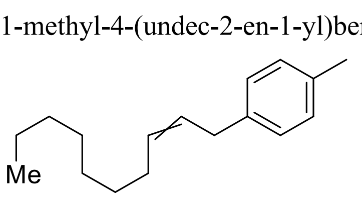

R$_f$= 0.9, PE. **^{1}H NMR** (400 MHz, Chloroform-*d*) δ 7.15-7.10 (m, 8H),

5.61-5.51 (m, 4H), 3.41-3.39 (m, 2H), 3.33-3.32 (m, 2H), 2.35 (m, 6H), 2.21-2.02 (m, 4H), 1.47-1.31 (m, 20H), 0.94-0.91 (m, 6H). **^{13}C NMR** (101 MHz, Chloroform-*d*) δ 138.24, 138.12, 135.26, 131.93, 130.85, 129.11, 129.05, 129.01, 128.39, 128.29, 128.25, 38.70, 33.11, 32.57, 31.93, 29.78, 29.37, 29.28, 29.22, 27.29, 22.73, 21.02, 14.16. **HRMS** (ESI-TOF) calculated for: $C_{17}H_{26}$ $[M+H]^+$: 231.2107, found: 231.2108.

1-methyl-4-(undec-2-en-1-yl)benzene **sub. 5g**

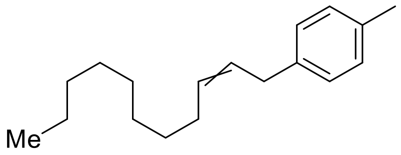

R_f = 0.9, PE. **^{1}H NMR** (400 MHz, Chloroform-*d*) δ 7.15-7.11 (m, 8H), 5.62-5.50 (m, 4H), 3.40 (d, *J* = 6.3 Hz, 2H), 3.33 (d, *J* = 5.8 Hz, 2H), 2.36 (m, 6H), 2.23-2.04 (m, 4H), 1.45-1.31 (m, 24H), 0.95-0.92 (m, 6H). **^{13}C NMR** (101 MHz, Chloroform-*d*) δ 138.22, 135.25, 131.92, 130.83, 129.09, 129.03, 128.83, 128.37, 128.26, 128.23, 125.47, 38.67, 33.08, 32.54, 31.92, 29.74, 29.54, 29.50, 29.38, 29.34, 29.23, 27.27, 22.71, 20.99, 14.12. **HRMS** (ESI-TOF) calculated for: $C_{18}H_{28}$ $[M+H]^+$: 245.2264, found: 245.2263.

3. General procedure

3.1 Reaction conditions

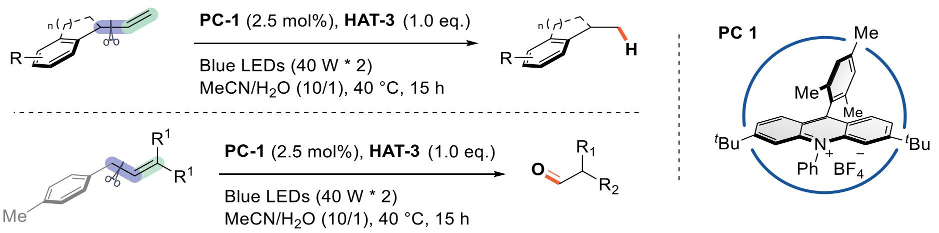

PC-1 (0.005 mmol, 2.9 mg) was weighed in an oven-dried 8 mL vial equipped with a magnetic stirring bar. H_2O (0.1 mL) and MeCN (1.0 mL) were added, followed by **HAT-3** (0.2 mmol) and the substrate of alkene (0.2 mmol). The reaction vessel was degassed, back-filled with argon, and placed between two kessil lights (40 W * 2). An oil bath was used to heat at 40 °C to ensure stable temperature control. The progress of the reaction was monitored by TLC. Upon completion, the reaction mixture was concentrated and purified by silica gel flash column chromatography.

3.2 Homologation of aldhydes

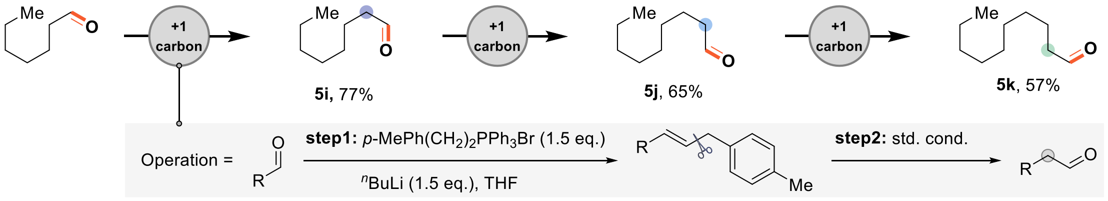

Step 1 was performed according to general procedure C; step 2 was performed under standard conditions.

3.3 Reaction setup

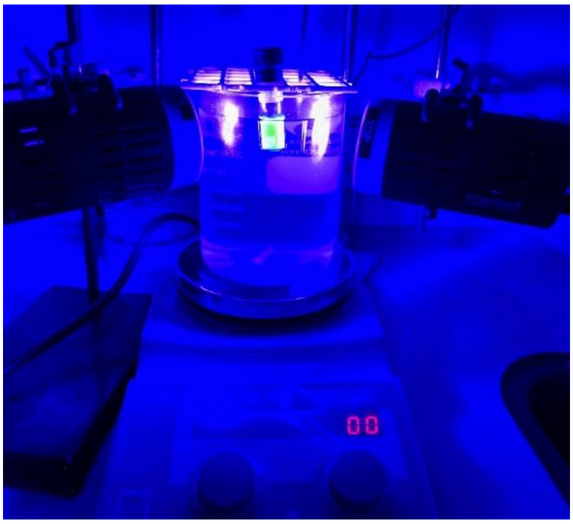

Supplementary Figure 1. Reaction setup

All products were characterized as shown below:

(3r,5r,7r)-1-(p-tolyl)adamantane **2e**

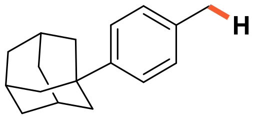

R_f= 0.9, PE. **^{1}H NMR** (400 MHz, Chloroform-*d*) δ 7.32-7.30 (m, 2H), 7.20-7.19 (m, 2H), 2.40-2.37 (m, 3H), 2.15-2.13 (m, 3H), 2.01-1.95 (m, 6H), 1.86-1.78 (m, 6H). **^{13}C NMR** (101 MHz, Chloroform-*d*) δ 148.50, 134.94, 128.84, 124.76, 43.30, 36.87, 35.86, 29.03, 20.92. **HRMS** (ESI-TOF) calculated for: $C_{17}H_{22}$ $[M+H]^+$: 227.1794, found: 227.1797.

4-bromo-1,2-dimethylbenzene **2h**

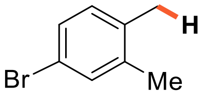

R_f= 0.8, PE. **^{1}H NMR** (400 MHz, Chloroform-*d*) δ 7.39 (d, *J* = 1.8 Hz, 1H), 7.14 (d, *J* = 7.7 Hz, 1H), 7.03 (dd, *J* = 7.7, 1.8 Hz, 1H), 2.38 (s, 3H), 2.32 (s, 3H). **^{13}C NMR** (101 MHz, Chloroform-*d*) δ 137.22, 134.60, 132.77, 130.51, 128.03, 124.66, 22.37, 20.56. **HRMS** (ESI-TOF) calculated for: C_8H_9Br $[M+H]^+$: 184.9960, found: 184.9961.

4'-methyl-[1,1'-biphenyl]-2-carbonitrile **2i**

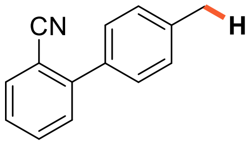

R_f= 0.5, PE/EA = 20:1 (v/v). **^{1}H NMR** (400 MHz, Chloroform-*d*) δ 7.78 (dd, *J* = 7.7, 1.4 Hz, 1H), 7.66 (td, *J* = 7.7, 1.4 Hz, 1H), 7.55-7.42 (m, 4H), 7.34-7.30 (m, 2H), 2.45 (s, 3H). **^{13}C NMR** (101 MHz, Chloroform-*d*) δ 145.55, 138.73, 135.30, 133.75, 132.81, 130.01, 129.48, 128.65, 127.31, 118.93, 111.21, 21.30. **HRMS** (ESI-TOF) calculated for: $C_{14}H_{11}N$ $[M+H]^+$: 194.0964, found: 194.0965.

methyl 4'-methyl-[1,1'-biphenyl]-4-carboxylate **2j**

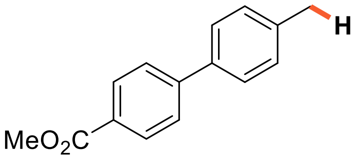

R_f= 0.3, PE/EA = 50:1 (v/v). **^{1}H NMR** (400 MHz, Chloroform-*d*) δ 8.12 (d, *J* = 8.4 Hz, 2H), 7.68 (d, *J* = 8.5 Hz, 2H), 7.56 (d, *J* = 8.2 Hz, 2H), 7.31-7.29 (m, 2H), 3.97 (s, 3H), 2.44 (s, 3H). **^{13}C NMR** (101 MHz, Chloroform-*d*) δ 167.07, 145.59, 138.13, 137.10, 130.10, 129.68, 128.61, 127.13, 126.81, 52.12, 21.19. **HRMS** (ESI-TOF) calculated for: $C_{15}H_{14}N_2$ $[M+H]^+$: 227.1067, found: 227.1066.

p-tolyl acetate **2k**

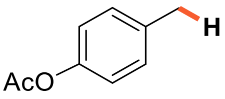

R_f= 0.3, PE/EA = 50:1 (v/v)., PE.**^{1}H NMR** (400 MHz, Chloroform-*d*) δ 7.21-7.18 (m, 2H), 7.01-6.97 (m, 2H), 2.37 (s, 3H), 2.31 (s, 3H). **^{13}C NMR** (101 MHz, Chloroform-*d*) δ

169.74, 148.47, 135.48, 129.96, 121.25, 21.13, 20.87. **HRMS** (ESI-TOF) calculated for: $C_9H_{10}O_2$ $[M+H]^+$: 151.0754, found: 151.0755.

trimethyl(p-tolyl)silane **2l**

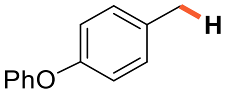

R_f= 0.7, PE. **^{1}H NMR** (400 MHz, Chloroform-*d*) δ 7.37-7.33 (m, 2H), 7.19-7.17 (m, 2H), 7.13-7.09 (m, 1H), 7.04-7.01 (m, 2H), 6.97-6.95 (m, 2H), 2.37 (s, 3H). **^{13}C NMR** (101 MHz, Chloroform-*d*) δ 157.84, 154.73, 132.93, 130.26, 129.67, 122.82, 119.16, 118.36, 20.74. **HRMS** (ESI-TOF) calculated for: $C_{13}H_{12}O$ $[M+H]^+$: 185.0961, found: 185.0962.

tert-butyl p-tolyl carbonate **2m**

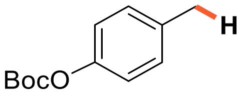

R_f= 0.5, PE/EA = 50:1 (v/v). **^{1}H NMR** (400 MHz, Chloroform-*d*) δ 7.20-7.18 (m, 2H), 7.08-7.06 (m, 2H), 2.36 (s, 3H), 1.58 (s, 9H). **^{13}C NMR** (101 MHz, Chloroform-*d*) δ 152.15, 148.91, 135.38, 129.87, 120.98, 83.33, 27.73, 20.85. **HRMS** (ESI-TOF) calculated for: $C_{12}H_{16}O_3$ $[M+H]^+$: 209.1172, found: 209.1173.

p-tolyl thiophene-2-carboxylate **2n**

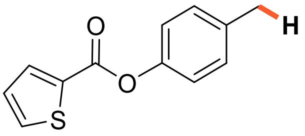

R_f= 0.4, PE/EA = 50:1 (v/v). **^{1}H NMR** (400 MHz, Chloroform-*d*) δ 8.01 (dd, *J* = 3.8, 1.3 Hz, 1H), 7.68 (dd, *J* = 5.1, 1.3 Hz, 1H), 7.26-7.24 (m, 2H), 7.20 (dd, *J* = 5.0, 3.8 Hz, 1H), 7.15-7.12 (m, 2H), 2.40 (s, 3H). **^{13}C NMR** (101 MHz, Chloroform-*d*) δ 160.83, 148.38, 135.67, 134.60, 133.40, 133.07, 130.02, 128.02, 121.36, 20.94. **HRMS** (ESI-TOF) calculated for: $C_{12}H_{10}O_2S$ $[M+H]^+$: 219.0474, found: 219.0475.

trimethyl(p-tolyl)silane **2o**

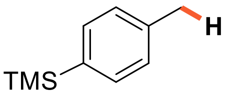

R_f= 0.8, PE. **^{1}H NMR** (400 MHz, Chloroform-*d*) δ 7.48-7.46 (m, 2H), 7.23-7.21 (m, 2H), 2.39 (s, 3H), 0.29 (m, 9H). **^{13}C NMR** (101 MHz, Chloroform-*d*) δ 138.62, 136.84, 133.38, 128.59, 21.45, -1.04. **HRMS** (ESI-TOF) calculated for $C_{18}H_{16}N_2$ (M - H^+): 259.1241, found: 259.1241. **HRMS** (ESI-TOF) calculated for: $C_{10}H_{16}Si$ $[M+H]^+$: 165.1094, found: 165.1104.

3-methyldibenzo[b,d]furan **2p**

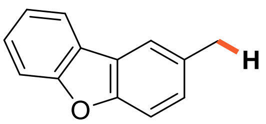

R_f= 0.8, PE/EA = 50:1 (v/v). **^{1}H NMR** (400 MHz, Chloroform-*d*) δ 7.95 (dd, *J* = 7.7, 1.4 Hz, 1H), 7.78 (m, 1H), 7.60-7.57 (m, 1H), 7.50-7.45 (m, 2H), 7.36 (td, *J* = 7.5, 1.0 Hz, 1H), 7.31-7.28 (m, 1H), 2.55 (s, 3H). **^{13}C NMR** (101 MHz, Chloroform-*d*) δ 156.47, 154.54,

132.21, 128.23, 126.95, 124.26, 124.20, 122.54, 120.66, 120.56, 111.65, 111.17, 21.39. **HRMS** (ESI-TOF) calculated for: $C_{13}H_{10}O$ [M+H]$^+$: 183.0804, found: 183.0804.

p-tolyl (1S,4R)-4,7,7-trimethyl-3-oxo-2-oxabicyclo[2.2.1]heptane-1-carboxylate **2q**

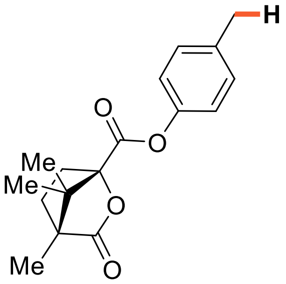

R_f= 0.4, PE/EA = 5:1 (v/v). **^{1}H NMR** (400 MHz, Chloroform-*d*) δ 7.23-7.21 (m, 2H), 7.04-7.01 (m, 2H), 2.59 (ddd, *J* = 13.5, 10.8, 4.3 Hz, 1H), 2.38 (s, 3H), 2.21 (ddd, *J* = 13.7, 9.4, 4.6 Hz, 1H), 2.01 (ddd, *J* = 13.2, 10.8, 4.6 Hz, 1H), 1.78 (ddd, *J* = 13.4, 9.4, 4.2 Hz, 1H), 1.18 (d, *J* = 8.1 Hz, 6H), 1.12 (s, 3H). **^{13}C NMR** (101 MHz, Chloroform-*d*) δ 177.91, 166.29, 147.77, 136.15, 130.09, 120.97, 90.91, 54.91, 54.67, 30.76, 29.00, 20.89, 16.88, 9.75. **HRMS** (ESI-TOF) calculated for: $C_{17}H_{20}O_4$ [M+H]$^+$: 289.1434, found: 289.1434.

p-tolyl 2-(4-(4-chlorobenzoyl)phenoxy)-2-methylpropanoate **2r**

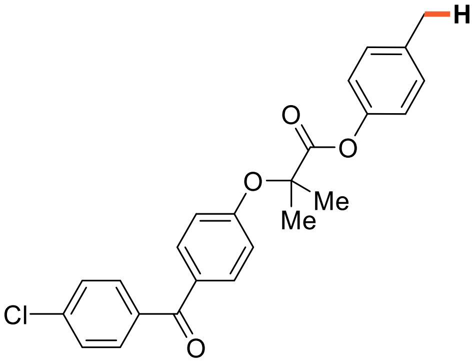

R_f = 0.6, PE/EA = 5:1 (v/v). **^{1}H NMR** (400 MHz, Chloroform-*d*) δ 7.82 (d, *J* = 8.8 Hz, 2H), 7.75 (d, *J* = 8.5 Hz, 2H), 7.48 (d, *J* = 8.5 Hz, 2H), 7.18 (d, *J* = 8.2 Hz, 2H), 7.03 (d, *J* = 8.8 Hz, 2H), 6.89 (d, *J* = 8.5 Hz, 2H), 2.35 (s, 3H), 1.85 (s, 6H). **^{13}C NMR** (101 MHz, Chloroform-*d*) δ 194.23, 172.60, 159.61, 148.19, 138.47, 136.35, 135.99, 132.16, 131.23, 130.65, 130.06, 128.60, 120.80, 117.36, 79.49, 25.47, 20.89. **HRMS** (ESI-TOF) calculated for: $C_{24}H_{21}ClO_4$ [M+H]$^+$: 409.1201, found: 409.1201.

p-tolyl (3aS,4S,6R,6aR)-6-methoxy-2,2-dimethyltetrahydrofuro[3,4-d][1,3]dioxole-4-carboxylate **2s**

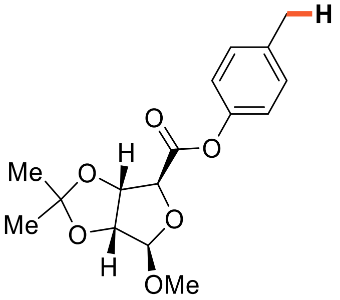

R_f= 0.6, PE/EA = 5:1 (v/v). **^{1}H NMR** (400 MHz, Chloroform-*d*) δ

7.20 (d, *J* = 8.4 Hz, 2H), 7.00 (d, *J* = 8.5 Hz, 2H), 5.38 (dd, *J* = 5.9, 1.0 Hz, 1H), 5.13 (s, 1H), 4.86 (t, *J* = 0.8 Hz, 1H), 4.65 (d, *J* = 5.9 Hz, 1H), 3.47 (s, 3H), 2.37 (s, 3H), 1.55 (s, 3H), 1.38 (s, 3H). **^{13}C NMR** (101 MHz, Chloroform-*d*) δ 169.01, 148.20, 135.80, 130.05, 120.86, 112.87, 109.60, 84.34, 83.62, 82.23, 55.78, 26.40, 25.04, 20.88. **HRMS** (ESI-TOF) calculated for: $C_{16}H_{20}O_6$ $[M+H]^+$: 309.1333, found: 309.1333.

p-tolyl 4-(2-hydroxypropan-2-yl)benzoate **2t**

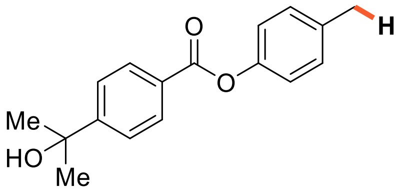

R_f = 0.7, PE/EA = 2:1 (v/v). ^{1}H NMR (400 MHz, Chloroform-*d*) δ 8.21-8.17 (m, 2H), 7.66-7.64 (m, 2H), 7.26-7.23 (m, 2H), 7.12-7.10 (m, 2H), 2.40 (s, 3H), 2.00 (s, 1H), 1.64 (s, 6H). **^{13}C NMR** (101 MHz, Chloroform-*d*) δ 165.26, 154.95, 148.74, 135.49, 130.22, 130.00, 128.06, 124.68, 121.39, 72.64, 31.75, 20.92. **HRMS** (ESI-TOF) calculated for: $C_{17}H_{18}O_3$ $[M+H]^+$: 271.1329, found: 271.1329.

2-(2-methyl-2-(p-tolyl)propyl)isoindoline-1,3-dione **2u**

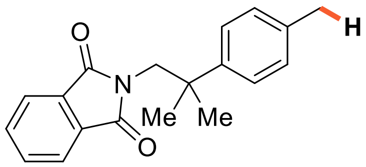

R_f = 0.2, PE/EA = 50:1 (v/v). **^{1}H NMR** (400 MHz, Chloroform-*d*) δ 7.86 (dd, *J* = 5.5, 3.0 Hz, 2H), 7.74 (dd, *J* = 5.4, 3.1 Hz, 2H), 7.40 (d, *J* = 8.2 Hz, 2H), 7.18 (d, *J* = 8.0 Hz, 2H), 3.82 (s, 2H), 2.37 (s, 3H), 1.41 (s, 6H). **^{13}C NMR** (101 MHz, Chloroform-*d*) δ 168.79, 143.77, 135.88, 133.93, 132.04, 128.96, 126.04, 123.28, 49.69, 40.11, 26.92, 20.97. **HRMS** (ESI-TOF) calculated for: $C_{19}H_{19}NO_2$ $[M+H]^+$: 294.1489, found: 294.1487.

5,5'-dimethyl-[1,1'-biphenyl]-2,2'-diyl dibenzoate **2v**

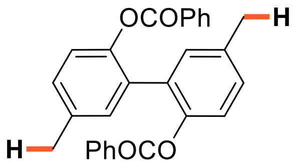

R_f = 0.4, PE/EA = 20:1 (v/v). **^{1}H NMR** (400 MHz, Chloroform-*d*) δ 8.03 (dd, *J* = 8.3, 1.4 Hz, 4H), 7.60-7.56 (m, 2H), 7.44 (t, *J* = 7.8 Hz, 4H), 7.24 (m, 2H), 7.18 (m, 4H), 2.34 (s, 6H). **^{13}C NMR** (101 MHz, Chloroform-*d*) δ 165.14, 146.11, 135.52, 133.28, 131.76, 130.27, 130.04, 129.65, 129.49, 128.40, 122.15, 20.85. **HRMS** (ESI-TOF) calculated for: $C_{28}H_{22}O_4$ $[M+H]^+$: 423.1591, found: 423.1591.

5-methyl-2,3-dihydro-1H-indene **2x**

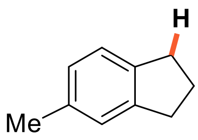

R_f = 0.9, PE. **^{1}H NMR** (400 MHz, Chloroform-*d*) δ 7.21 (d, *J* = 7.6 Hz, 1H), 7.14

(m, 1H), 7.04 (d, *J* = 7.5 Hz, 1H), 2.96 (ddd, *J* = 9.2, 5.3, 1.8 Hz, 4H), 2.41 (s, 3H), 2.18-2.11 (m, 2H). **^{13}C NMR** (101 MHz, Chloroform-*d*) δ 144.43, 141.17, 135.60, 126.82, 125.18, 124.14, 32.86, 32.51, 25.65, 21.29. **HRMS** (ESI-TOF) calculated for: $C_{10}H_{12}$ $[M+H]^+$: 133.1012, found: 133.1014.

1-(3,4-dichlorophenyl)-1,2,3,4-tetrahydronaphthalene **2y**

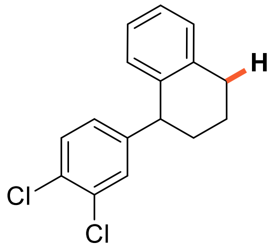

R_f = 0.8, PE/EA = 50:1 (v/v). **^{1}H NMR** (400 MHz, Chloroform-*d*) δ 7.37 (d, *J* = 8.3 Hz, 1H), 7.23 (d, *J* = 2.2 Hz, 1H), 7.19 (d, *J* = 4.3 Hz, 2H), 7.10 (dt, *J* = 8.4, 4.2 Hz, 1H), 6.96 (dd, *J* = 8.3, 2.2 Hz, 1H), 6.84 (d, *J* = 7.7 Hz, 1H), 4.13 (t, *J* = 6.5 Hz, 1H), 2.92 (dtd, *J* = 16.9, 10.9, 10.1, 5.6 Hz, 2H), 2.19 (ddd, *J* = 12.4, 8.3, 4.5 Hz, 1H), 1.92-1.75 (m, 3H). **^{13}C NMR** (101 MHz, Chloroform-*d*) δ 147.90, 138.02, 137.58, 132.23, 130.73, 130.20, 130.03, 129.93, 129.26, 128.30, 126.41, 125.93, 44.89, 33.13, 29.62, 20.75. **HRMS** (ESI-TOF) calculated for: $C_{16}H_{14}Cl_2$ $[M-H]^-$: 275.0400, found: 275.0400.

6,7,8,9-tetrahydro-5H-benzo[7]annulene **2z**

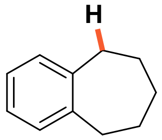

R_f = 0.9, PE. **^{1}H NMR** (400 MHz, Chloroform-*d*) δ 7.13 (m, 4H), 2.85-2.82 (m, 4H), 1.88 (p, *J* = 5.9 Hz, 2H), 1.69 (p, *J* = 5.8 Hz, 4H). **^{13}C NMR** (101 MHz, Chloroform-*d*) δ 143.50, 129.01, 125.94, 36.73, 32.80, 28.34. **HRMS** (ESI-TOF) calculated for: $C_{11}H_{14}$ $[M+H]^+$: 147.1168, found: 147.1169.

1-ethyl-4-isobutylbenzene **2aa**

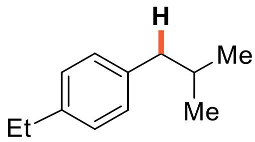

R_f = 0.9, PE. **^{1}H NMR** (400 MHz, Chloroform-*d*) δ 7.16-7.14 (m, 2H), 7.11 -7.09 (m, 2H), 2.66 (q, *J* = 7.6 Hz, 2H), 2.48 (d, *J* = 7.3 Hz, 2H), 1.89 (ddd, *J* = 14.7, 7.4, 6.0 Hz, 1H), 1.27 (td, *J* = 7.6, 1.3 Hz, 3H), 0.94 (dd, *J* = 6.6, 1.5 Hz, 6H). **^{13}C NMR** (101 MHz, Chloroform-*d*) δ 141.44, 138.88, 129.06, 127.55, 45.08, 30.28, 28.46, 22.42, 15.65. **HRMS** (ESI-TOF) calculated for: $C_{12}H_{18}$ $[M+H]^+$: 163.1481, found: 163.1482.

6-(tert-butyl)-4-ethyl-1,1-dimethyl-2,3-dihydro-1H-indene **2ab**

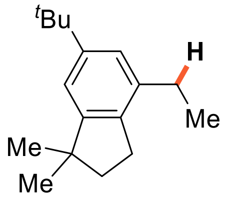

R_f = 0.9, PE. **^{1}H NMR** (400 MHz, Chloroform-*d*) δ 7.10-7.07 (m, 2H), 2.86 (t,

J = 7.2 Hz, 2H), 2.65 (q, J = 7.6 Hz, 2H), 1.97 (t, J = 7.2 Hz, 2H), 1.38 (s, 9H), 1.31-1.26 (m, 9H). **^{13}C NMR** (101 MHz, Chloroform-*d*) δ 152.26, 149.92, 139.05, 138.09, 122.67, 116.34, 44.11, 41.38, 34.73, 31.74, 28.86, 27.81, 26.73, 14.49. **HRMS** (ESI-TOF) calculated for: $C_{17}H_{26}$ $[M+H]^+$: 231.2107, found: 231.2109.

6-ethyl-1,1,2,4,4,7-hexamethyl-1,2,3,4-tetrahydronaphthalene **2ac**

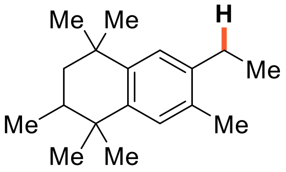

R_f = 0.9, PE. **^{1}H NMR** (400 MHz, Chloroform-*d*) δ 7.19-7.10 (m, 2H), 2.64 (q, J = 7.6 Hz, 2H), 2.31 (s, 3H), 1.92 (dtt, J = 13.5, 6.8, 3.4 Hz, 1H), 1.68 (t, J = 13.2 Hz, 1H), 1.42-1.35 (m, 7H), 1.31-1.25 (m, 6H), 1.11 (s, 3H), 1.03 (d, J = 6.8 Hz, 3H). **^{13}C NMR** (101 MHz, Chloroform-*d*) δ 143.30, 142.25, 139.24, 132.99, 128.38, 125.64, 43.92, 37.32, 34.69, 34.06, 32.47, 32.15, 28.68, 25.94, 25.04, 19.02, 16.89, 14.34. **HRMS** (ESI-TOF) calculated for: $C_{18}H_{28}$ $[M+Na]^+$: 267.2083, found: 267.2083.

4-(4-methylbenzyl)tetrahydro-2H-pyran **2ad**

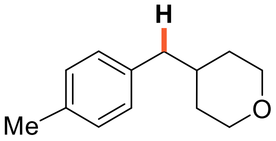

R_f = 0.3, PE/EA = 50:1 (v/v). **^{1}H NMR** (400 MHz, Chloroform-*d*) δ 7.13 (d, J = 7.8 Hz, 2H), 7.07 (d, J = 8.0 Hz, 2H), 3.99-3.94 (m, 2H), 3.35 (td, J = 11.8, 2.2 Hz, 2H), 2.53 (d, J = 7.2 Hz, 2H), 2.35 (s, 3H), 1.75 (tdp, J = 11.1, 7.3, 3.8 Hz, 1H), 1.58 (ddq, J = 13.2, 4.1, 2.2 Hz, 2H), 1.41-1.29 (m, 2H). **^{13}C NMR** (101 MHz, Chloroform-*d*) δ 137.05, 135.38, 129.04, 128.93, 68.10, 43.11, 37.23, 33.00, 21.05. **HRMS** (ESI-TOF) calculated for: $C_{13}H_{18}O$ $[M+H]^+$: 191.1430, found: 191.1431.

ethyl 5-oxopentanoate **3b**

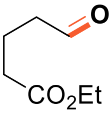

R_f = 0.4, PE/EA = 20:1 (v/v). **^{1}H NMR** (400 MHz, Chloroform-*d*) δ 9.75 (m, 1H), 4.10 (q, J = 7.1 Hz, 2H), 2.51 (td, J = 7.2, 1.4 Hz, 2H), 2.34 (t, J = 7.3 Hz, 2H), 1.93 (p, J = 7.2 Hz, 2H), 1.23 (t, J = 7.2 Hz, 3H).**^{13}C NMR** (101 MHz, Chloroform-*d*) δ 201.54, 172.87, 60.41, 42.89, 33.15, 17.31, 14.17. **HRMS** (ESI-TOF) calculated for: $C_7H_{12}O_3$ $[M+H]^+$: 145.0859, found: 145.0859.

6-chlorohexanal **3c**

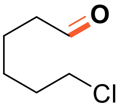

R_f = 0.5, PE/EA = 20:1 (v/v). **^{1}H NMR** (400 MHz, Chloroform-*d*) δ 9.80-9.79 (m, 1H), 3.56 (t, J = 6.6 Hz, 3H), 2.48 (td, J = 7.3, 1.7 Hz, 2H), 1.81 (dq, J = 7.9, 6.7 Hz, 3H), 1.72-1.64 (m, 3H), 1.55-1.46 (m, 3H). **^{13}C NMR** (101 MHz, Chloroform-*d*) δ 202.26, 44.69, 43.68, 32.29, 26.39, 21.30. **HRMS** (ESI-TOF) calculated for: $C_6H_{11}ClO$ $[M+H]^+$: 135.0571, found: 135.0573.

4-(1,3-dioxoisoindolin-2-yl)butanal **3d**

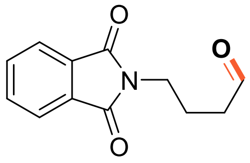

R_f = 0.2, PE/EA = 5:1 (v/v). **^{1}H NMR** (400 MHz, Chloroform-*d*) δ 9.78 (t, *J* = 1.2 Hz, 1H), 7.88-7.83 (m, 2H), 7.76-7.71 (m, 2H), 3.75 (t, *J* = 6.8 Hz, 2H), 2.55 (td, *J* = 7.2, 1.2 Hz, 2H), 2.03 (p, *J* = 7.0 Hz, 2H). **^{13}C NMR** (101 MHz, Chloroform-*d*) δ 200.86, 168.38, 134.04, 132.02, 123.30, 41.10, 37.13, 21.18. **HRMS** (ESI-TOF) calculated for: $C_{12}H_{11}NO_3$ $[M+H]^+$: 218.0812, found: 218.0812.

4-(4-bromophenyl)butanal **3e**

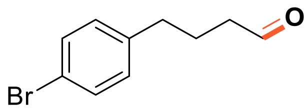

R_f = 0.3, PE/EA = 50:1 (v/v). **^{1}H NMR** (400 MHz, Chloroform-*d*) δ 9.78-9.77 (m, 1H), 7.42 (dd, *J* = 8.4, 1.0 Hz, 2H), 7.08-7.06 (m, 2H), 2.63 (t, *J* = 7.6 Hz, 2H), 2.47 (tt, *J* = 7.2, 1.3 Hz, 2H), 1.99-1.91 (m, 2H). **^{13}C NMR** (101 MHz, Chloroform-*d*) δ 201.98, 140.22, 131.53, 130.22, 119.86, 43.00, 34.39, 23.44. **HRMS** (ESI-TOF) calculated for: $C_{10}H_{11}BrO$ $[M+Na]^+$: 248.9885, found: 248.9885.

4-(4-(tert-butyl)phenyl)-3-methylbutanal **3f**

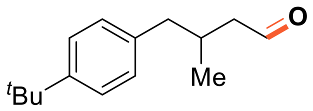

R_f = 0.4, PE/EA = 50:1 (v/v). **^{1}H NMR** (400 MHz, Chloroform-*d*) δ 9.74 (m, 1H), 7.34-7.32 (m, 2H), 7.12-7.10 (m, 2H), 2.58 (d, *J* = 7.0 Hz, 2H), 2.49-2.34 (m, 2H), 2.25 (ddd, *J* = 15.7, 7.8, 2.6 Hz, 1H), 1.34 (s, 9H), 1.02 (d, *J* = 6.5 Hz, 3H). **^{13}C NMR** (101 MHz, Chloroform-*d*) δ 202.78, 149.03, 136.88, 128.86, 125.24, 50.25, 42.70, 34.39, 31.41, 30.25, 20.06. **HRMS** (ESI-TOF) calculated for: $C_{15}H_{22}O$ $[M+H]^+$: 219.1743, found: 219.1743.

cyclododecanecarbaldehyde **3h**

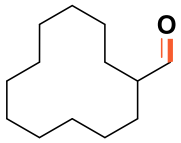

R_f = 0.5, PE/EA = 50:1 (v/v). **^{1}H NMR** (400 MHz, Chloroform-*d*) δ 9.66 (d, *J* = 1.7 Hz, 1H), 2.41 (ddd, *J* = 15.4, 8.5, 6.5 Hz, 1H), 1.66 (dt, *J* = 12.0, 5.9 Hz, 2H), 1.58-1.50 (m, 2H), 1.45-1.26 (m, 18H). **^{13}C NMR** (101 MHz, Chloroform-*d*) δ 205.48, 47.75, 23.75, 23.61, 23.56, 23.49, 23.36, 22.34. **HRMS** (ESI-TOF) calculated for: $C_{13}H_{24}O$ $[M+H]^+$: 197.1900, found: 197.1902.

4-(1-oxopropan-2-yl)benzonitrile **3i**

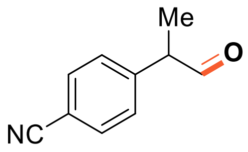

R_f = 0.3, PE/EA = 3:1 (v/v). **^{1}H NMR** (400 MHz, Chloroform-*d*) δ 9.71 (d,

J = 1.3 Hz, 1H), 7.70-7.68 (m, 2H), 7.36 (d, J = 8.3 Hz, 2H), 3.77-3.72 (m, 1H), 1.51 (d, J = 7.2 Hz, 3H). **^{13}C NMR** (101 MHz, Chloroform-*d*) δ 199.54, 143.13, 132.79, 129.13, 118.50, 111.64, 52.90, 14.56. **HRMS** (ESI-TOF) calculated for: $C_{10}H_9NO$ $[M+H]^+$: 160.0757, found: 160.0758.

4-oxocyclohexane-1-carbaldehyde **3j**

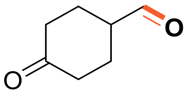

R_f = 0.2, PE/EA = 3:1 (v/v). **^{1}H NMR** (400 MHz, Chloroform-*d*) δ 9.78 (m, 1H), 2.73-2.65 (m, 1H), 2.51-2.35 (m, 4H), 2.27-2.20 (m, 2H), 2.04-1.94 (m, 2H). **^{13}C NMR** (101 MHz, Chloroform-*d*) δ 209.76, 202.41, 47.31, 40.18, 39.61, 39.47, 28.31, 25.47. **HRMS** (ESI-TOF) calculated for: $C_7H_{10}O_2$ $[M+H]^+$: 124.0754, found: 124.0755.

5-methyl-6-oxohexanenitrile **3k**

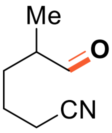

R_f = 0.4, PE/EA = 3:1 (v/v). **^{1}H NMR** (400 MHz, Chloroform-*d*) δ 9.65 (d, J = 1.6 Hz, 1H), 2.45-2.37 (m, 3H), 1.93-1.84 (m, 1H), 1.76-1.69 (m, 2H), 1.62-1.51 (m, 1H), 1.18 (d, J = 7.2 Hz, 3H). **^{13}C NMR** (101 MHz, Chloroform-*d*) δ 203.81, 119.22, 45.59, 29.27, 22.91, 17.31, 13.44. **HRMS** (ESI-TOF) calculated for: $C_7H_{11}NO$ $[M+H]^+$: 126.0913, found: 126.0915.

2-methylundecanal **3m**

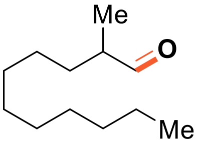

R_f = 0.4, PE/EA = 50:1 (v/v). **^{1}H NMR** (400 MHz, Chloroform-*d*) δ 9.63 (d, J = 2.0 Hz, 1H), 2.35 (qd, J = 6.8, 2.1 Hz, 1H), 1.72 (td, J = 7.6, 4.1 Hz, 1H), 1.41-1.28 (m, 15H), 1.10 (d, J = 7.0 Hz, 3H), 0.90 (t, J = 6.7 Hz, 3H). **^{13}C NMR** (101 MHz, Chloroform-*d*) δ 205.46, 46.34, 31.89, 30.54, 29.64, 29.54, 29.47, 29.31, 26.95, 22.68, 14.12, 13.33. **HRMS** (ESI-TOF) calculated for: $C_{12}H_{24}O$ $[M+H]^+$: 185.1900, found: 185.1900.

(S)-4,8-dimethylnonanal **3n**

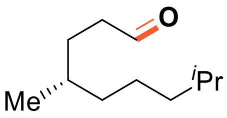

R_f = 0.4, PE/EA = 50:1 (v/v). **^{1}H NMR** (400 MHz, Chloroform-*d*) δ 9.79 (t, J = 2.0 Hz, 1H), 2.44 (dtd, J = 8.4, 6.3, 2.0 Hz, 2H), 1.67 (qd, J = 9.7, 9.1, 6.9 Hz, 1H), 1.54 (dt, J = 13.3, 6.6 Hz, 1H), 1.46 (td, J = 8.4, 5.9 Hz, 2H), 1.28 (ddt, J = 20.1, 16.1, 6.7 Hz, 3H), 1.15 (ddd, J = 11.6, 9.2, 5.4 Hz, 3H), 0.91-0.88 (m, 9H). **^{13}C NMR** (101 MHz, Chloroform-*d*) δ 203.06, 41.74, 39.22, 36.93, 32.41, 28.93, 27.96, 24.68, 22.69, 22.59, 19.36. **HRMS** (ESI-TOF) calculated for: $C_{11}H_{22}O$ $[M+H]^+$: 171.1743, found: 171.1744.

(3aS,4R,7R,7aS)-octahydro-1H-4,7-methanoindene-5-carbaldehyde **3p**

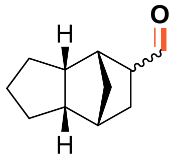

R_f= 0.4, PE/EA = 50:1 (v/v). (d.r.=2.3:1) **1H NMR** (400 MHz, Chloroform-*d*) δ 9.81 (d, *J* = 1.3 Hz, 1H), 9.68 (d, *J* = 1.6 Hz, 1H), 2.72-2.67 (m, 1H), 2.52-2.50 (m, 1H), 2.33-2.32 (m, 1H), 2.29-2.25 (m, 1H), 2.09-2.07 (m, 2H), 1.93-1.81 (m, 7H), 1.68-1.56 (m, 5H), 1.55-1.52 (m, 1H), 1.34-1.10 (m, 6H), 1.01-0.90 (m, 5H). ^{13}**C NMR** (101 MHz, Chloroform-*d*) δ 205.30, 203.59, 54.31, 53.35, 48.12, 48.07, 48.05, 42.92, 42.68, 42.19, 41.62, 40.42, 33.76, 32.54, 32.25, 32.15, 31.88, 30.09, 29.49, 28.64, 27.26, 26.93. **HRMS** (ESI-TOF) calculated for: $C_{11}H_{16}O$ $[M+H]^+$: 165.1274, found: 165.1274.

6-oxohexyl (S)-3,3,3-trifluoro-2-methoxy-2-phenylpropanoate **3q**

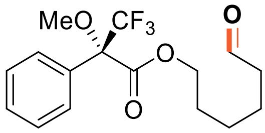

R_f= 0.6, PE/EA = 3:1 (v/v). ^{1}**H NMR** (400 MHz, Chloroform-*d*) δ 9.75 (t, *J* = 1.6 Hz, 1H), 7.54-7.52 (m, 2H), 7.44-7.40 (m, 3H), 4.40-4.27 (m, 2H), 3.56 (q, *J* = 1.3 Hz, 3H), 2.42 (td, *J* = 7.3, 1.6 Hz, 2H), 1.77-1.60 (m, 4H), 1.44-1.33 (m, 2H). ^{13}C NMR (101 MHz, Chloroform-*d*) δ 202.11, 166.57, 132.31, 129.63, 128.43, 127.28, 123.33 (q, *J* = 283.9 Hz), 84.59 (q, *J* = 27.7 Hz), 66.08, 55.43 (d, *J* = 1.5 Hz), 43.58, 28.13, 25.31, 21.44. ^{19}**F NMR** (376 MHz, Chloroform-*d*) δ -71.59. **HRMS** (ESI-TOF) calculated for: $C_{16}H_{19}F_3O_4$ $[M+H]^+$: 333.1308, found: 333.1308.

6-oxohexyl 4-(N,N-dipropylsulfamoyl)benzoate **3r**

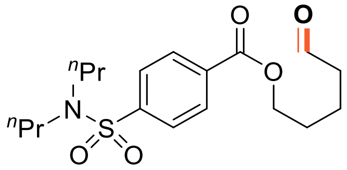

R_f= 0.3, PE/EA = 5:1 (v/v). ^{1}**H NMR** (400 MHz, Chloroform-*d*) δ 9.77 (t, *J* = 1.6 Hz, 1H), 8.14 (d, *J* = 8.5 Hz, 2H), 7.86 (d, *J* = 8.5 Hz, 2H), 4.34 (t, *J* = 6.6 Hz, 2H), 3.11-3.07 (m, 4H), 2.48 (td, *J* = 7.2, 1.6 Hz, 2H), 1.75 (m, 4H), 1.56-1.46 (m, 6H), 0.85 (t, *J* = 7.4 Hz, 6H). ^{13}**C NMR** (101 MHz, Chloroform-*d*) δ 202.22, 165.23, 144.19, 133.62, 130.16, 126.98, 65.30, 49.93, 43.67, 28.46, 25.58, 21.92, 21.63, 11.13. **HRMS** (ESI-TOF) calculated for: $C_{19}H_{29}NO_5S$ $[M+H]^+$: 384.1839, found: 384.1838.

dodec-11-enal **3s**

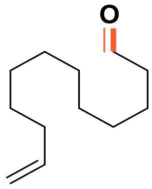

R_f= 0.6, PE/EA = 50:1 (v/v). ^{1}**H NMR** (400 MHz, Chloroform-*d*) δ 9.78 (t, *J* = 2.0 Hz, 1H), 5.83 (ddt, *J* = 16.9, 10.2, 6.7 Hz, 1H), 5.04-4.93 (m, 2H), 2.44 (td, *J* = 7.3, 1.9 Hz, 2H), 2.05 (q, *J* = 7.0 Hz, 2H), 1.63 (q, *J* = 7.2 Hz, 2H), 1.41-1.30 (m, 12H). ^{13}**C NMR** (101 MHz,

Chloroform-*d*) δ 203.00, 139.22, 114.15, 43.94, 33.81, 29.41, 29.37, 29.35, 29.16, 29.10, 28.92, 22.08. **HRMS** (ESI-TOF) calculated for: $C_{12}H_{22}O$ [M+H]$^{+}$: 183.1743, found: 183.1745.

6-oxohexyl 2-(11-oxo-6,11-dihydrodibenzo[b,e]oxepin-9-yl)acetate **3t**

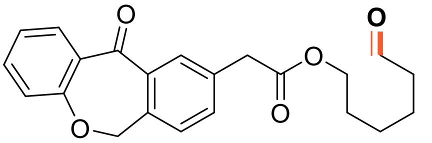

R_f = 0.3, PE/EA = 3:1 (v/v). **^{1}H NMR** (400 MHz, Chloroform-*d*) δ 9.74 (t, *J* = 1.7 Hz, 1H), 8.12 (d, *J* = 2.4 Hz, 1H), 7.89 (dd, *J* = 7.7, 1.4 Hz, 1H), 7.56 (td, *J* = 7.5, 1.5 Hz, 1H), 7.49-7.42 (m, 2H), 7.36 (dd, *J* = 7.3, 1.3 Hz, 1H), 7.03 (d, *J* = 8.4 Hz, 1H), 5.19 (s, 2H), 4.11 (t, *J* = 6.5 Hz, 2H), 3.64 (s, 2H), 2.43 (td, *J* = 7.3, 1.7 Hz, 2H), 1.69-1.60 (m, 4H), 1.41-1.33 (m, 2H). **^{13}C NMR** (101 MHz, Chloroform-*d*) δ 202.34, 190.82, 171.44, 160.46, 140.43, 136.35, 135.57, 132.80, 132.41, 129.45, 129.26, 127.90, 127.84, 125.14, 121.05, 73.62, 64.68, 43.68, 40.28, 28.35, 25.48, 21.62. **HRMS** (ESI-TOF) calculated for: $C_{22}H_{22}O_5$ [M+H]$^{+}$: 367.1540, found: 367.1541.

1-(tert-butyl)-4-ethylbenzene **4f**

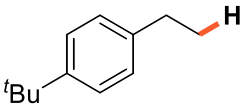

R_f= 0.9, PE. **^{1}H NMR** (400 MHz, Chloroform-*d*) δ 7.36-7.32 (m, 2H), 7.18 -7.16 (m, 2H), 2.65 (q, *J* = 7.6 Hz, 2H), 1.34 (s, 9H), 1.26 (t, *J* = 7.7 Hz, 3H). **^{13}C NMR** (101 MHz, Chloroform-*d*) δ 148.37, 141.16, 127.50, 125.18, 34.33, 31.43, 28.26, 15.48. **HRMS** (ESI-TOF) calculated for: $C_{12}H_{18}$ [M+H]$^{+}$: 163.1481, found: 163.1482.

1-isopropyl-4-propylbenzene **4g**

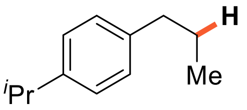

R_f= 0.9, PE. **^{1}H NMR** (400 MHz, Chloroform-*d*) δ 7.19-7.13(m, 4H), 2.92 (hept, *J* = 6.9 Hz, 1H), 2.59 (dd, *J* = 8.7, 6.8 Hz, 2H), 1.72-1.63 (m, 2H), 1.28 (d, *J* = 6.9 Hz, 6H), 0.98 (t, *J* = 7.3 Hz, 3H). **^{13}C NMR** (101 MHz, Chloroform-*d*) δ 146.09, 140.02, 128.35, 126.23, 37.68, 33.70, 24.60, 24.09, 13.94. **HRMS** (ESI-TOF) calculated for: $C_{12}H_{18}$ [M+H]$^{+}$: 163.1481, found: 163.1483.

1-isobutyl-3-methylbenzene **4h**

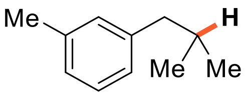

R_f= 0.9, PE. **^{1}H NMR** (400 MHz, Chloroform-*d*) δ 7.21 (td, *J* = 7.5, 1.7 Hz, 1H), 7.05-6.98 (m, 3H), 2.48 (dd, *J* = 7.3, 2.0 Hz, 2H), 2.38 (d, *J* = 2.0 Hz, 3H), 1.90 (dtd, *J* = 13.6, 6.8, 2.0 Hz, 1H), 0.95 (dd, *J* = 7.2, 2.0 Hz, 6H). **^{13}C NMR** (101 MHz, Chloroform-*d*) δ 141.67, 137.56, 129.96, 127.96, 126.36, 126.17, 45.44, 30.24, 22.45, 21.44. **HRMS** (ESI-TOF) calculated for: $C_{11}H_{16}$ [M+H]$^{+}$: 149.1325, found: 149.1326.

2-(2-isopropylphenyl)acetaldehyde **5b**

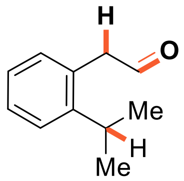

R_f= 0.5, PE/EA = 50:1. **^{1}H NMR** (400 MHz, Chloroform-*d*) δ 9.75 (t, *J* = 2.3 Hz, 1H), 7.39-7.31 (m, 2H), 7.24-7.16 (m, 2H), 3.78 (d, *J* = 2.4 Hz, 2H), 3.04 (p, *J* = 6.8 Hz, 1H), 1.25 (d, *J* = 6.8 Hz, 6H). **^{13}C NMR** (101 MHz, Chloroform-*d*) δ 199.77, 147.78, 130.90, 129.03, 128.16, 126.22, 125.89, 48.23, 29.41, 23.74. **HRMS** (ESI-TOF) calculated for: $C_{11}H_{14}O$ $[M+H]^+$: 163.1117, found: 163.1118.

4. **Mechanistic study**

4.1 Evidence of migration

Deuterium labelling:

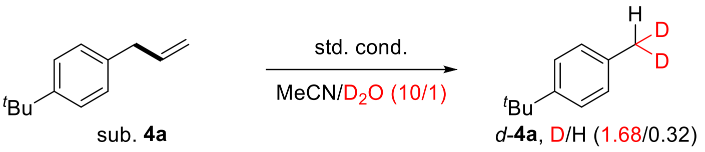

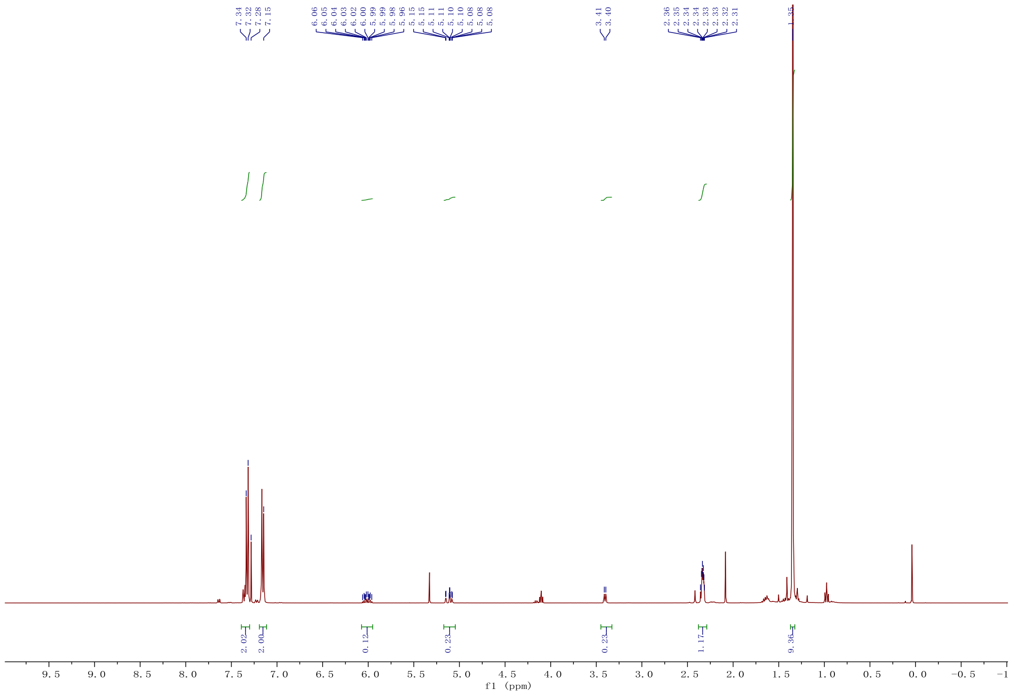

Supplementary Figure 2. NMR of the deuterium labelling experiment

A mixture of **sub. 4a** and *d*-**4a** were separated, and the D/H was calculated to be 1.68/1.32.

Trapping experiments:

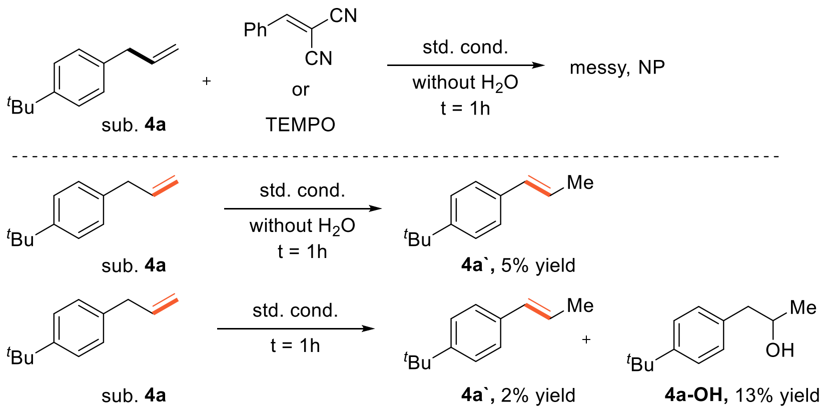

Supplementary Figure 3. Trapping experiments

4.2 ^{18}O labelling experiment

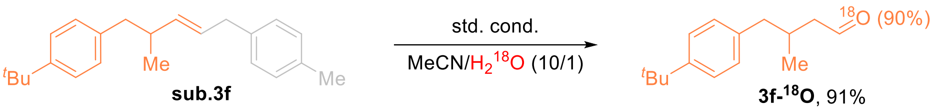

Supplementary Figure 4. The ^{18}O labelling experiment

4.3 Light on-off/intensity experiment

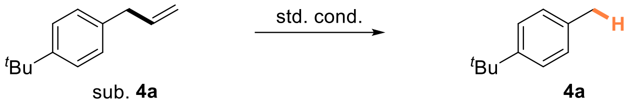

Supplementary Table 1. Light on-off experiment

**t/h**	0	1	2	3	4	5	6
**yield**	0	19	19	48	50	64	64

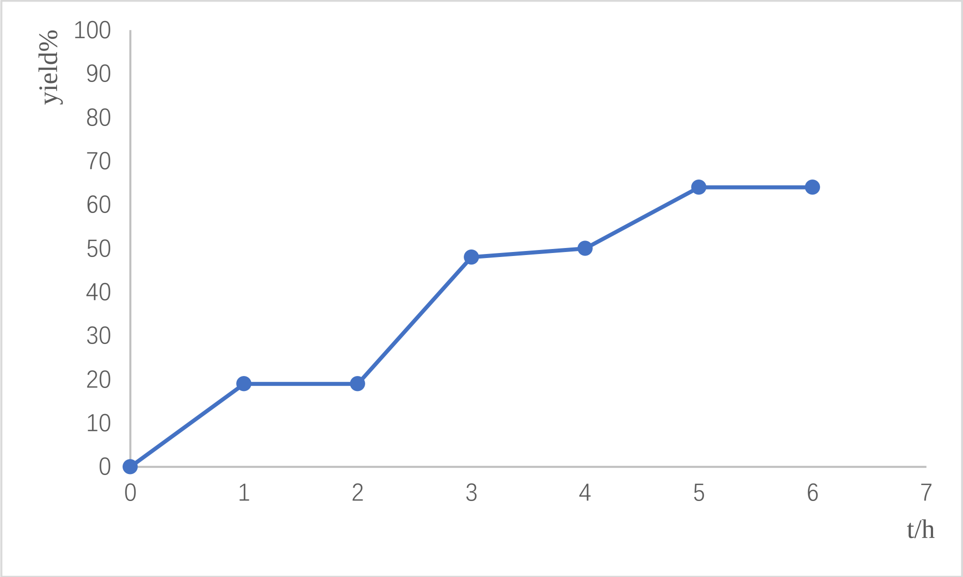

Supplementary Figure 5. Light on-off experiment

Supplementary Table 2. Light intensity study

**t/h**	0	1	2	3	4
**yield% (10W*2)**	0	2	8	18	31
**yield% (20W*2)**	0	9	31	55	67
**yield% (40W*2)**	0	19	48	64	72

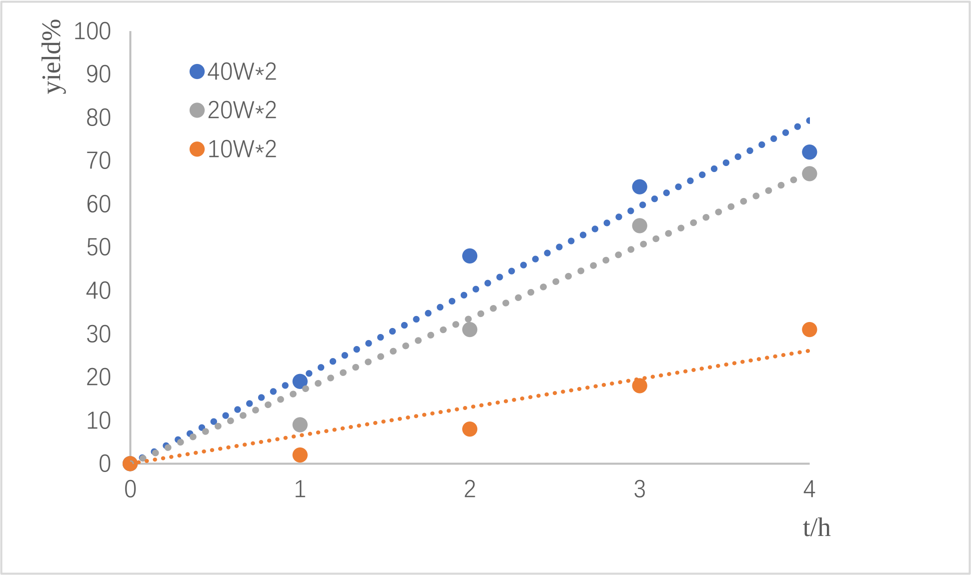

Supplementary Figure 6. Light intensity study

4.4 Kinetic study

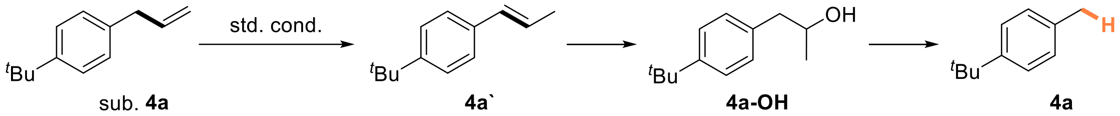

Supplementary Table 3. Kinetic study

**t/h**	0	1	2	3	4	15
**yield% (1)**	0	27	47	68	75	93
**yield% (1a`)**	0	10	5	3	2	0
**remaining% (1a)**	100	48	36	19	10	0

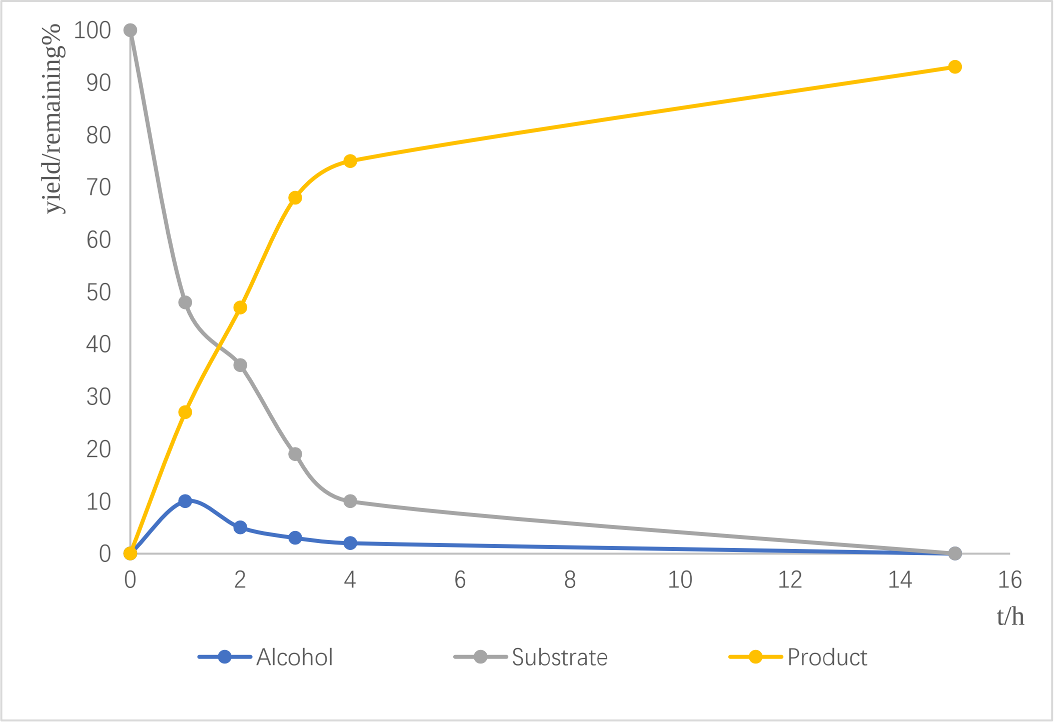

Supplementary Figure 7. Kinetic study

4.5 Stern-Volmer quenching experiment

The concentration of the photocatalyst was maintained constant (0.1 mM), varying only the quencher concentration. The excitation wavelength was fixed at 440 nm, while the emission light was acquired from 420 nm to 750 nm. For quenching data, the emission wavelength was fixed at 511 nm.

Supplementary Table 4. Data at different concentrations of **HAT-3** (5 mL MeCN was used as solvent)

concentration	quantity	Emission intensity(I_0/I-1)
0 mM	0 μL	0
1 mM	0.65 μL	-0.002
3 mM	1.95 μL	0.009
5 mM	3.25 μL	0.006
7 mM	4.55 μL	0.006

Supplementary Table 5. Data at different concentrations of **sub. 2b** (5 mL MeCN was used as solvent)

concentration	quantity	Emission intensity(I_0/I-1)
0 mM	0 μL	0
1 mM	0.80 μL	0.025
3 mM	2.40 μL	0.043
5 mM	4.00 μL	0.071
7 mM	5.60 μL	0.094

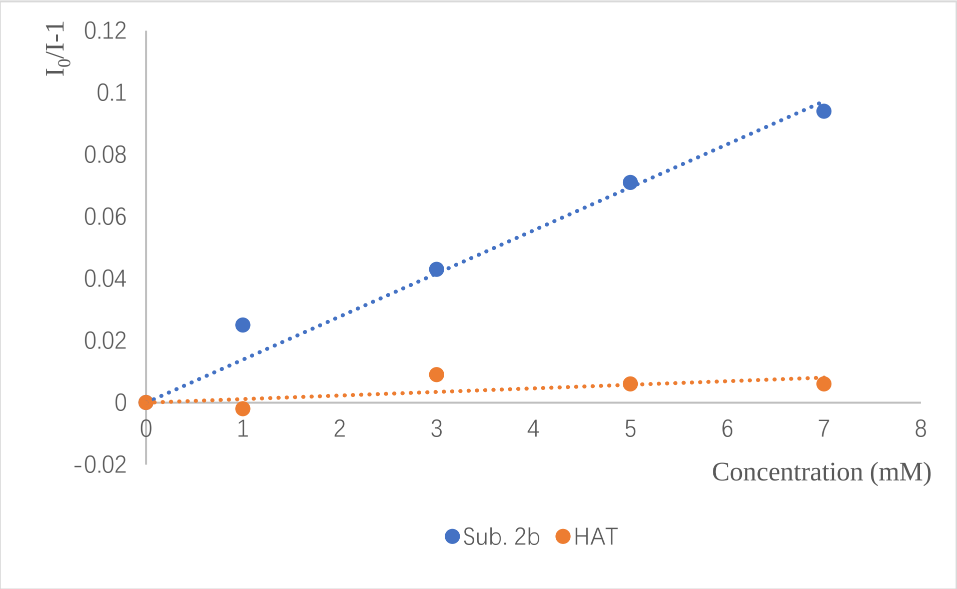

Supplementary Figure 8. Stern-Volmer quenching experiment

4.6 Domino C−C bond cleavage

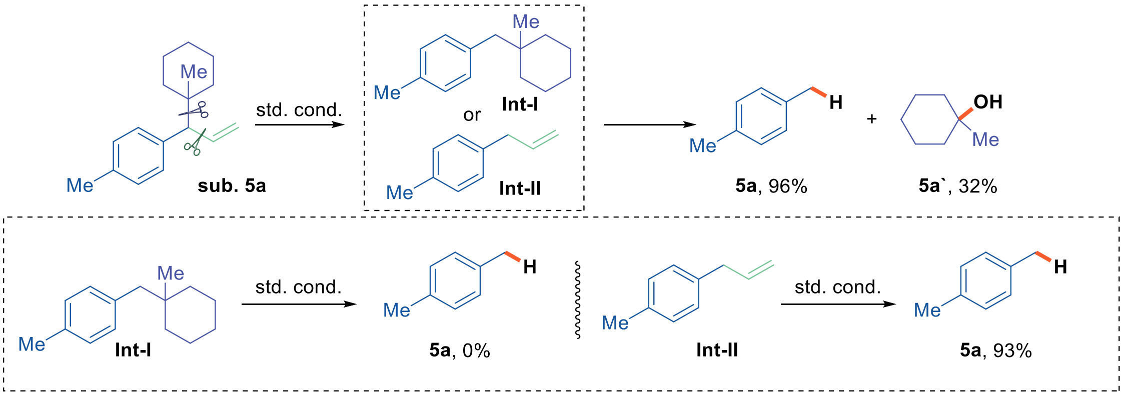

Supplementary Figure 9. Domino C−C bond cleavage experiment

4.7 Plausible mechanism

Allylarenes:

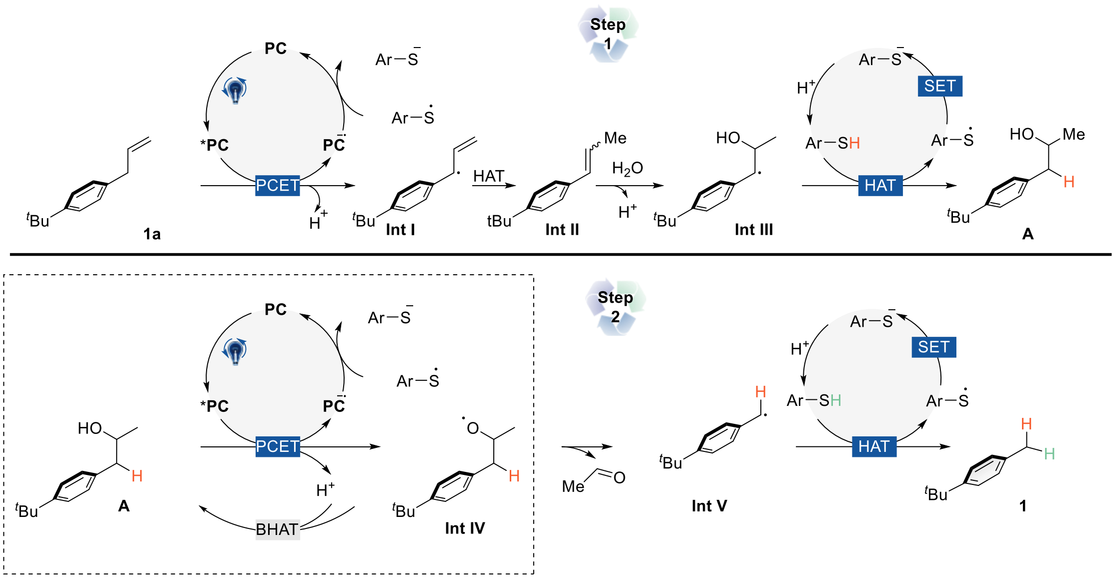

Supplementary Figure 10. A plausible mechanism of allylarenes

Trisubstituted alkenes:

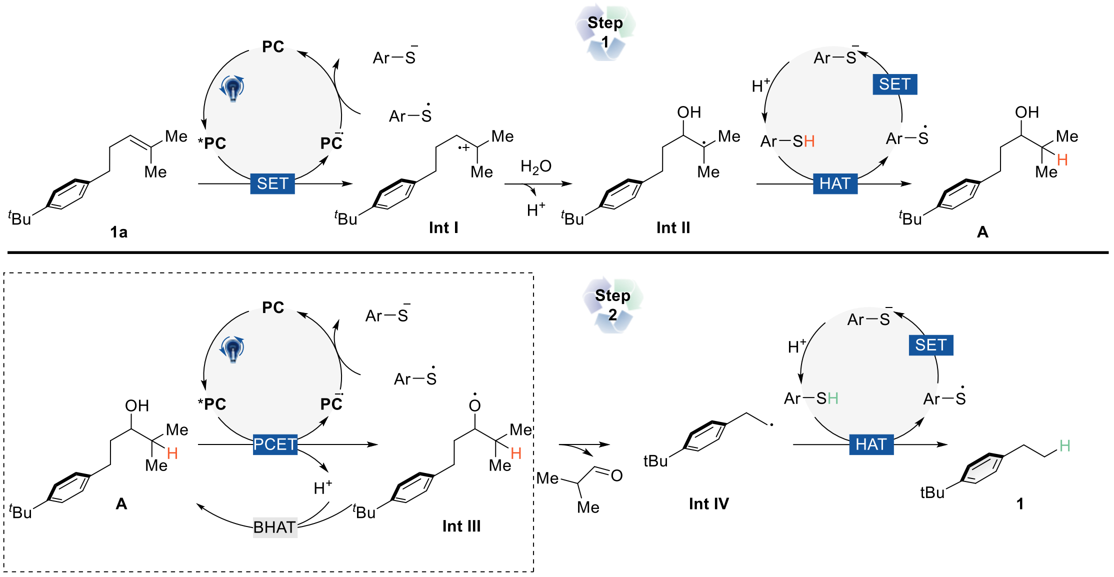

Supplementary Figure 11. Plausible mechanism of trisubstituted alkenes

5. ^{1}H NMR and ^{13}C NMR spectra

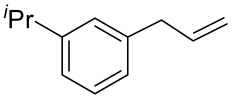

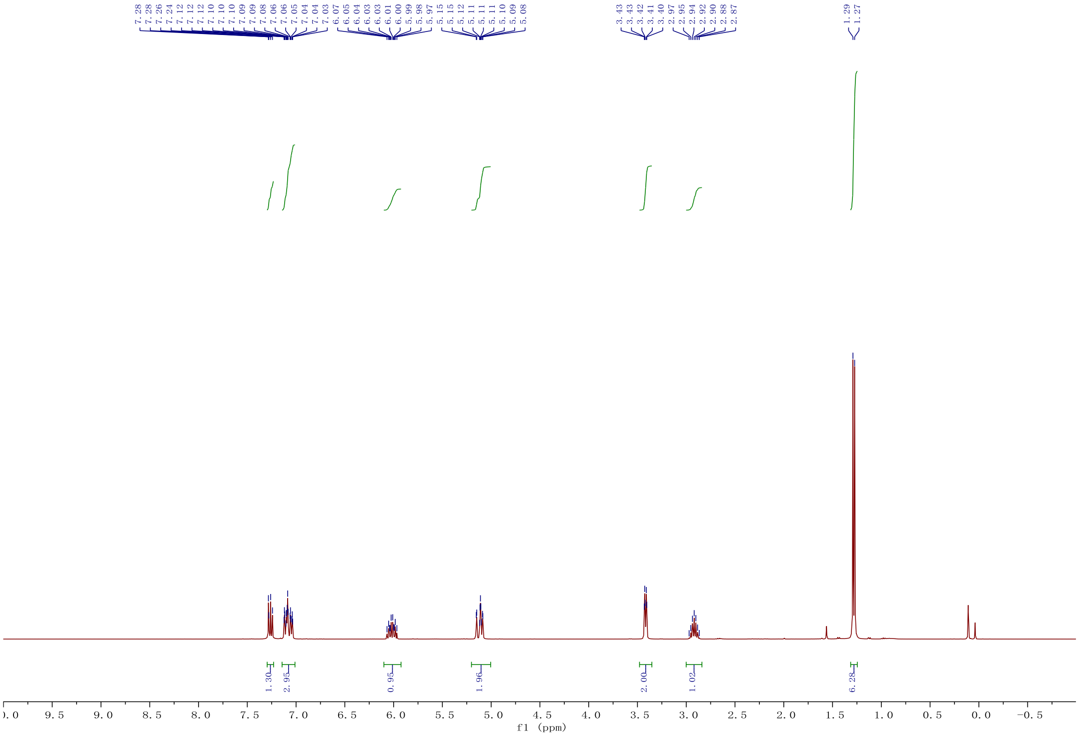

Supplementary Figure 12. ^{1}H NMR (400 MHz, Chloroform-*d*) of **sub. 2d**

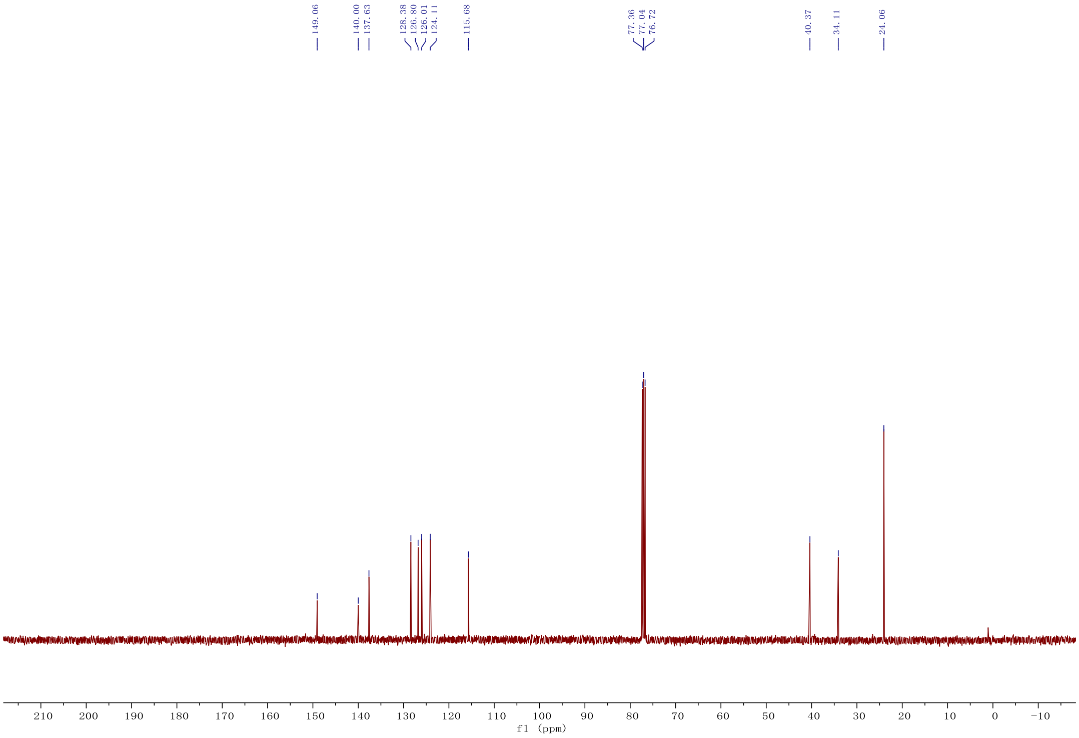

Supplementary Figure 13. ^{13}C NMR (101 MHz, Chloroform-*d*) of **sub. 2d**

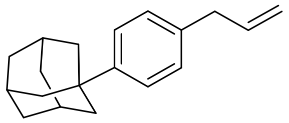

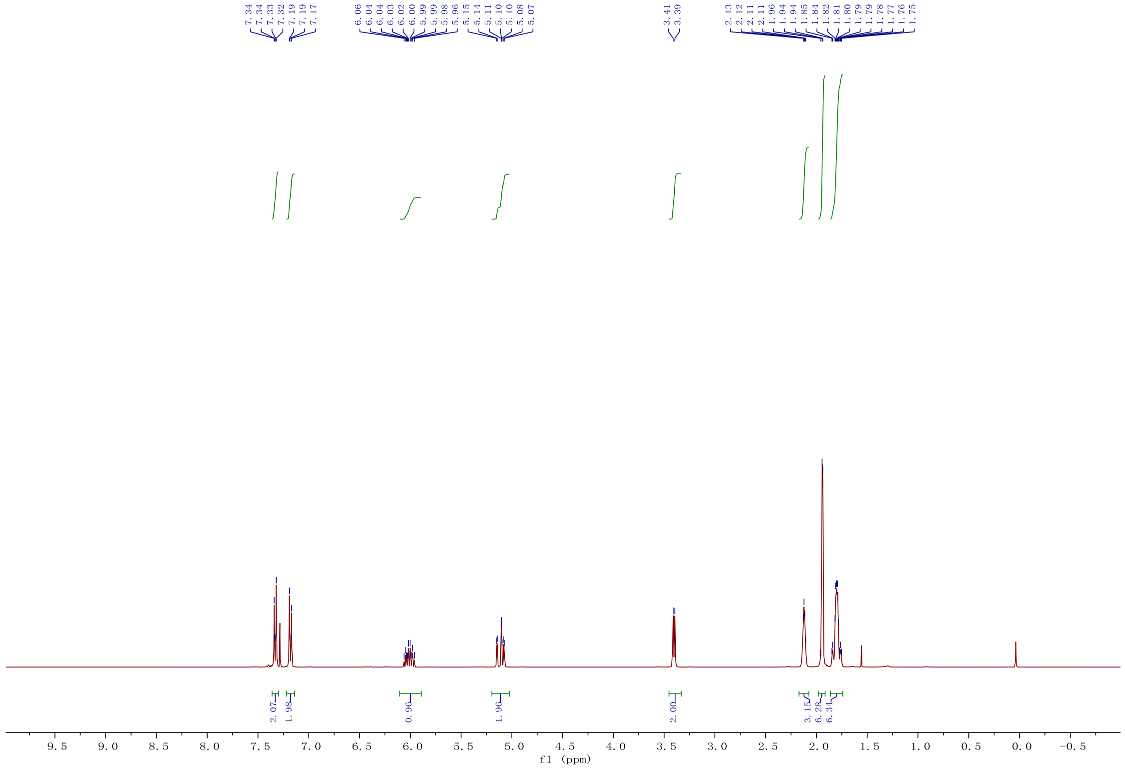

Supplementary Figure 14. ^{1}H NMR (400 MHz, Chloroform-*d*) of **sub. 2e**

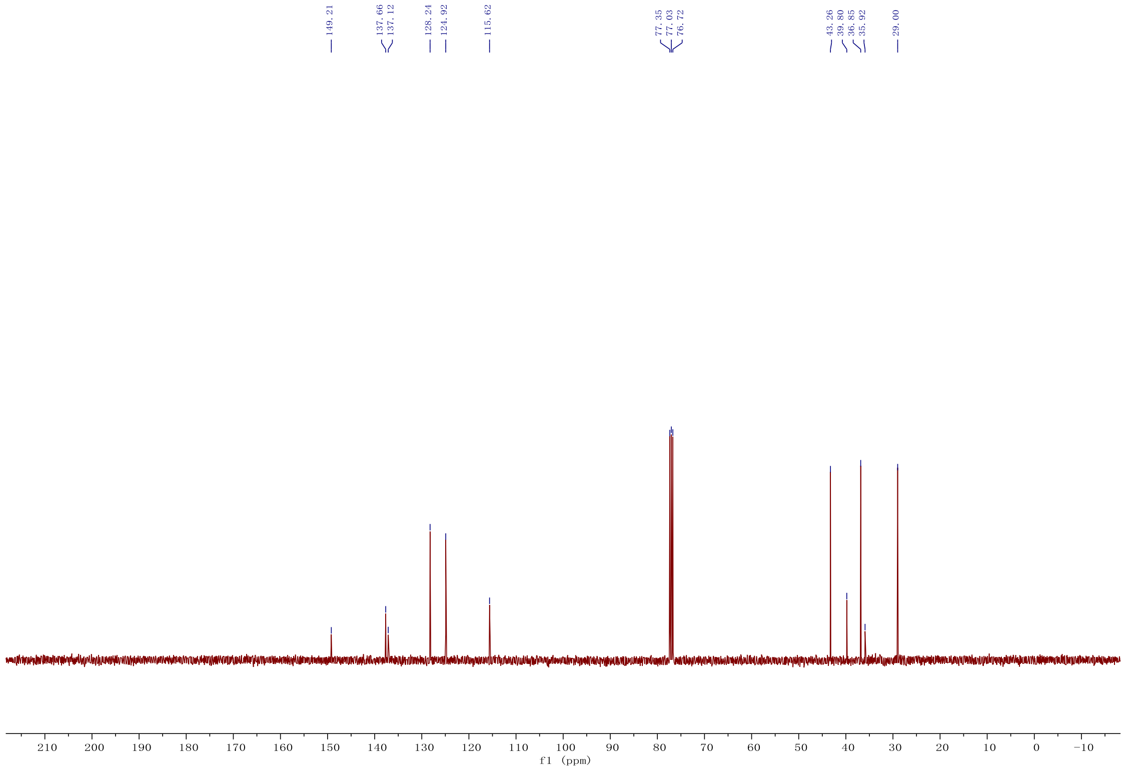

Supplementary Figure 15. ^{13}C NMR (101 MHz, Chloroform-*d*) of **sub. 2e**

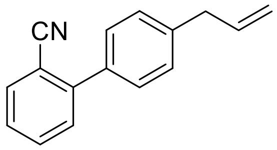

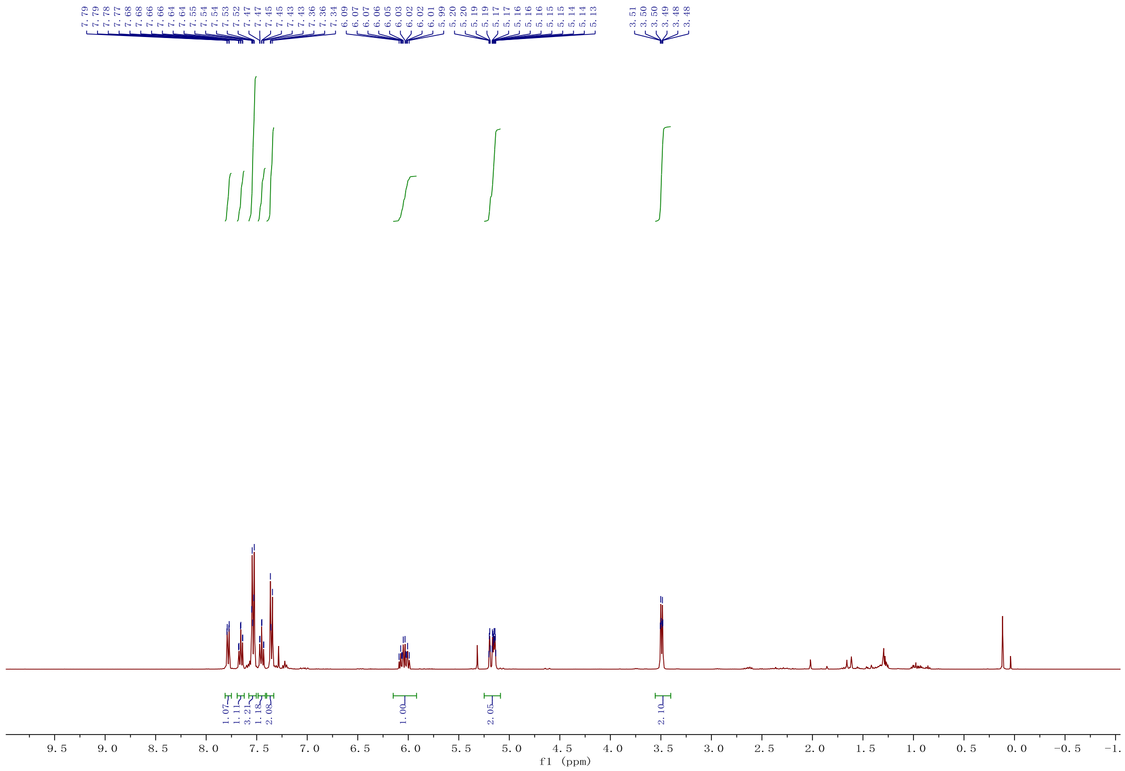

Supplementary Figure 16. ^{1}**H NMR** (400 MHz, Chloroform-*d*) of **sub. 2i**

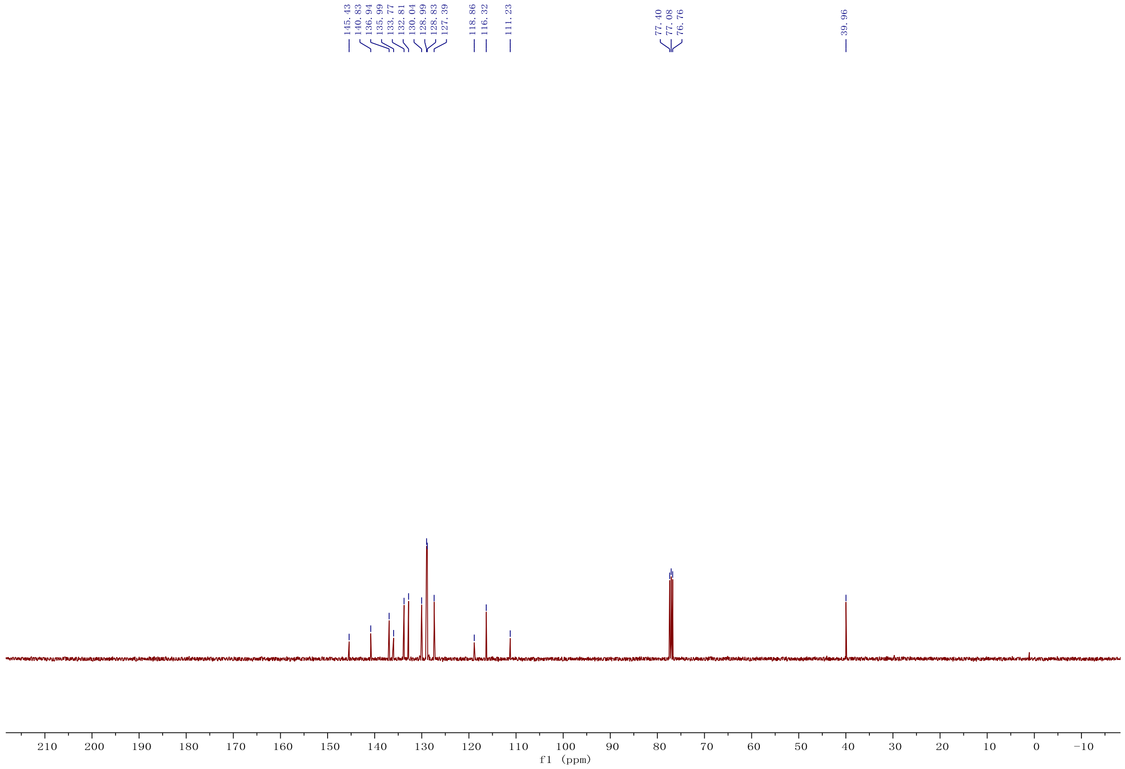

Supplementary Figure 17. ^{13}**C NMR** (101 MHz, Chloroform-*d*) of **sub. 2i**

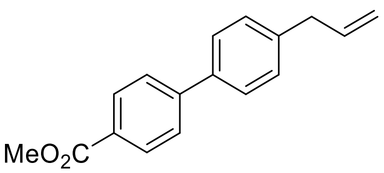

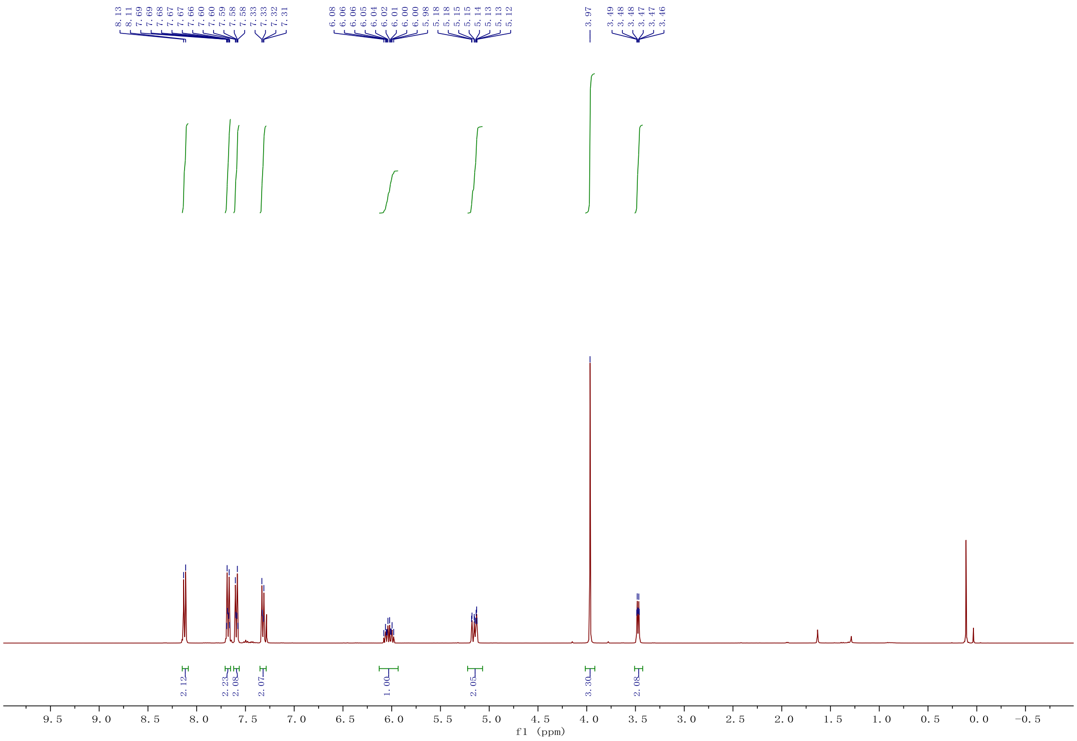

Supplementary Figure 18. ^{1}**H NMR** (400 MHz, Chloroform-*d*) of **sub. 2j**

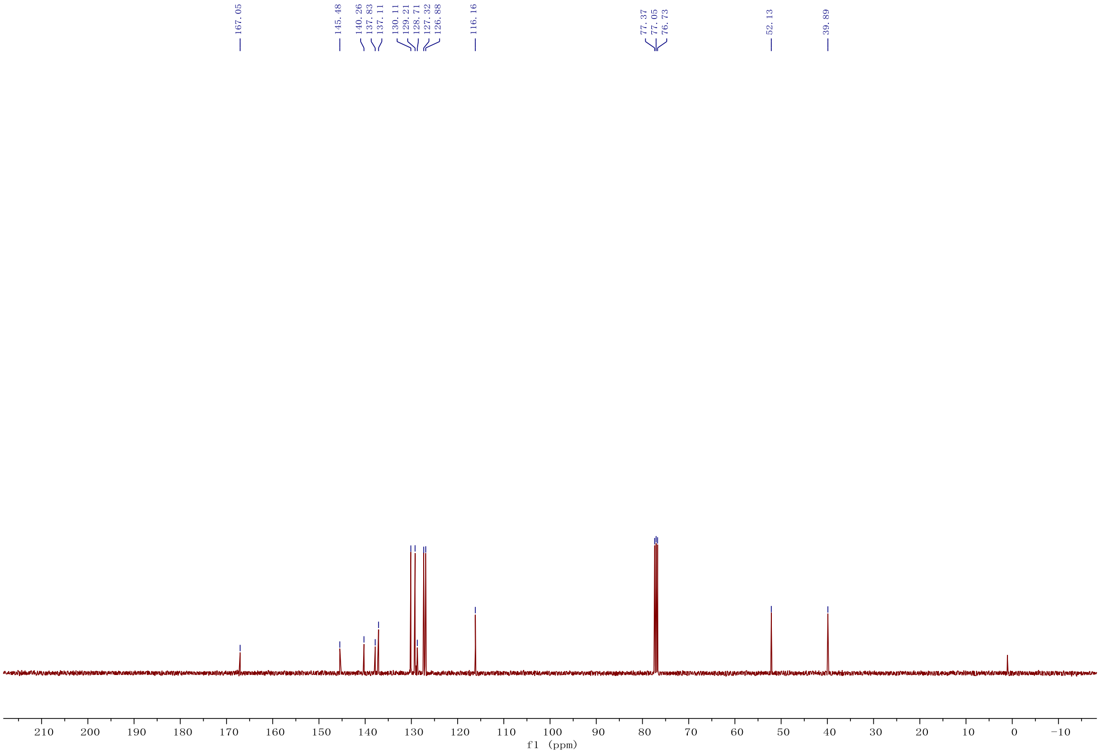

Supplementary Figure 19. ^{13}**C NMR** (101 MHz, Chloroform-*d*) of **sub. 2j**

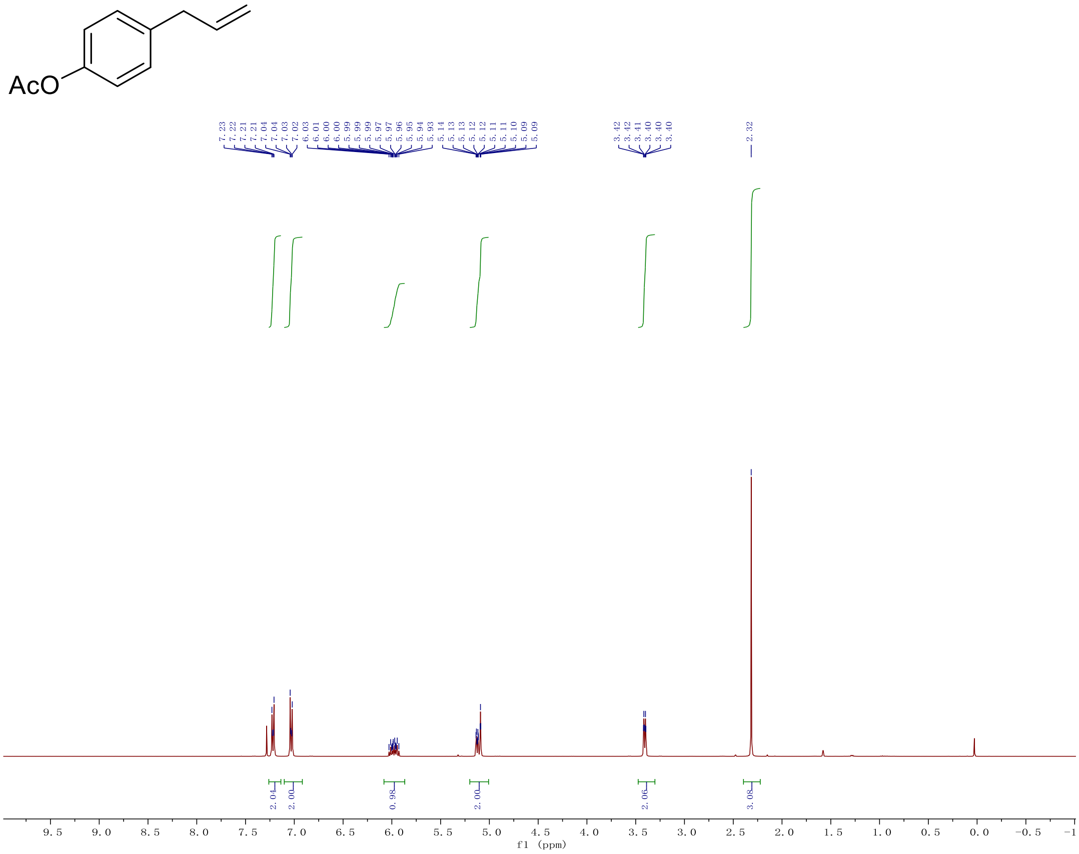

Supplementary Figure 20. ^{1}**H NMR** (400 MHz, Chloroform-*d*) of **sub. 2k**

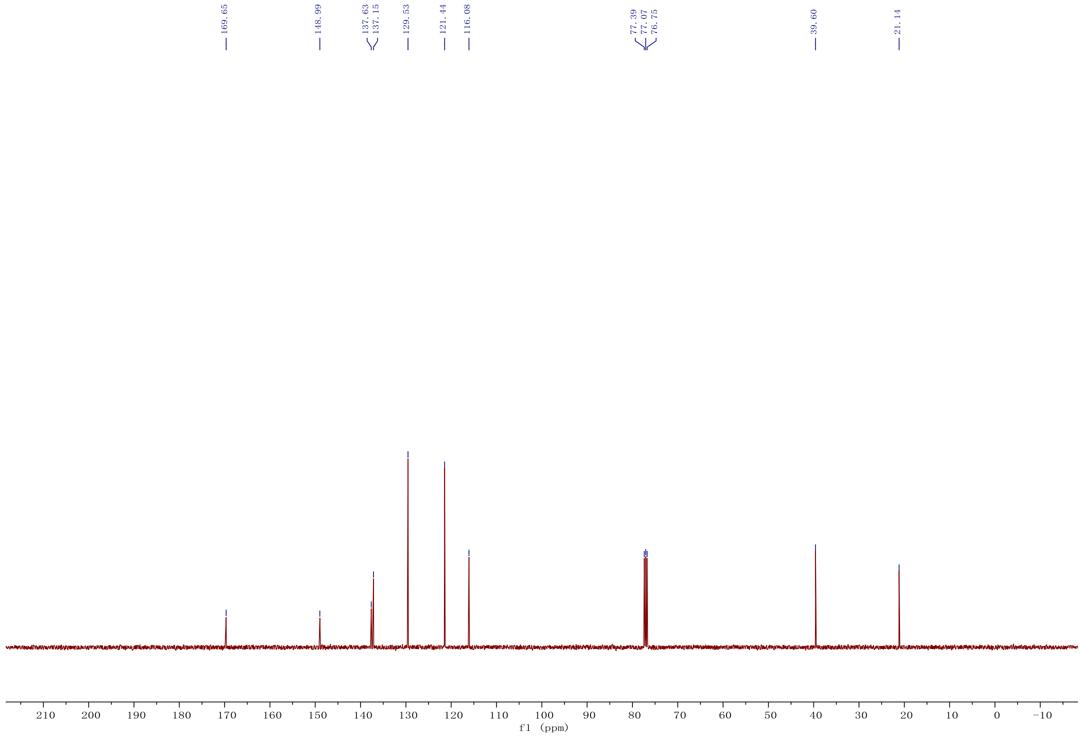

Supplementary Figure 21. ^{13}**C NMR** (101 MHz, Chloroform-*d*) of **sub. 2k**

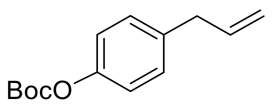

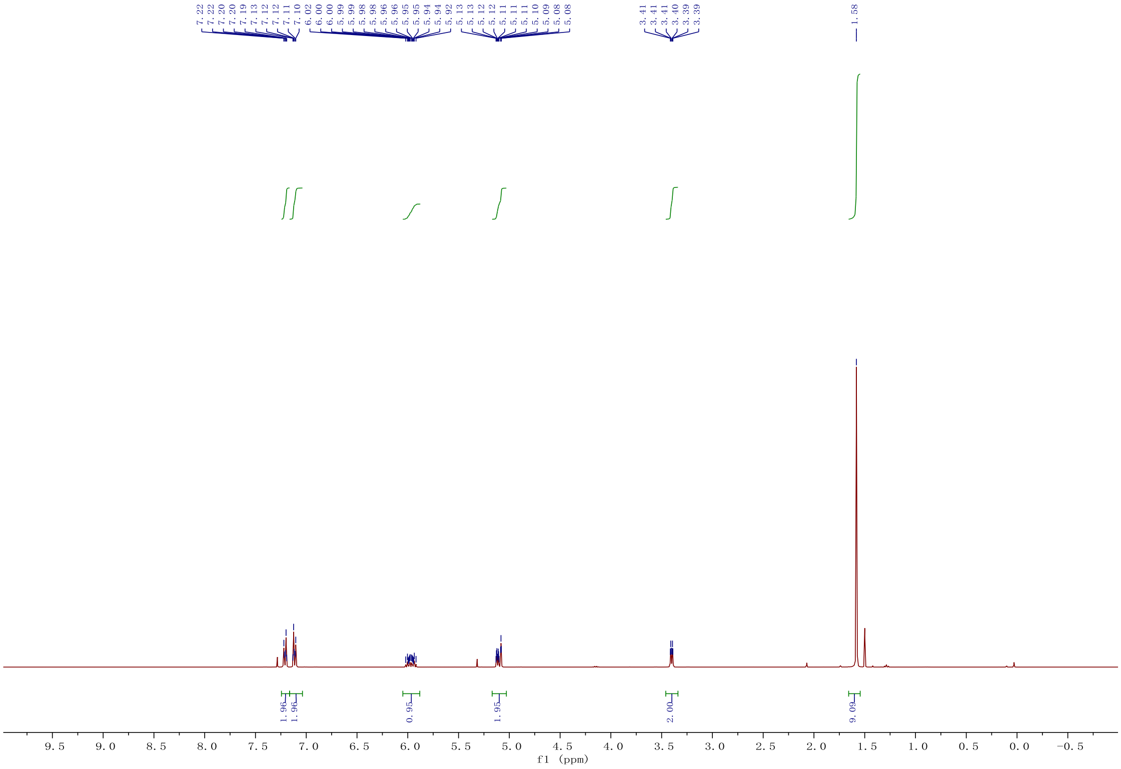

Supplementary Figure 22. ^{1}**H NMR** (400 MHz, Chloroform-*d*) of **sub. 2m**

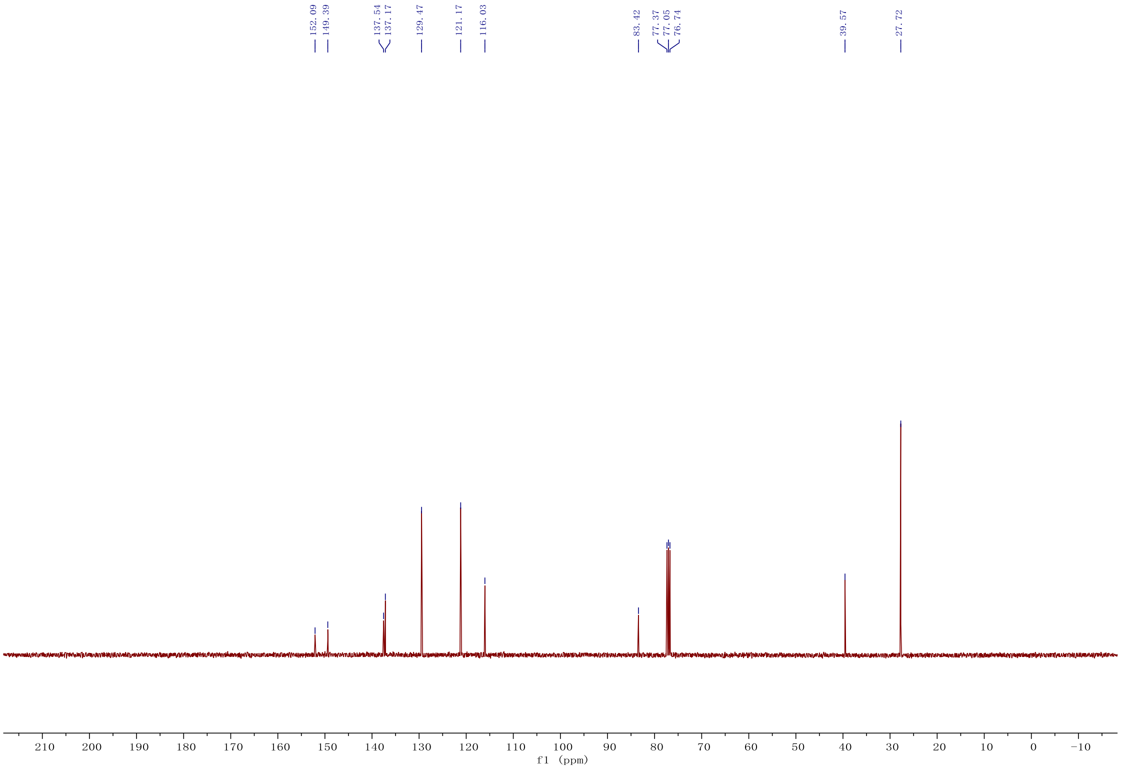

Supplementary Figure 23. ^{13}**C NMR** (101 MHz, Chloroform-*d*) of **sub. 2m**

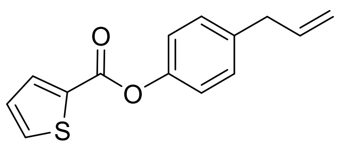

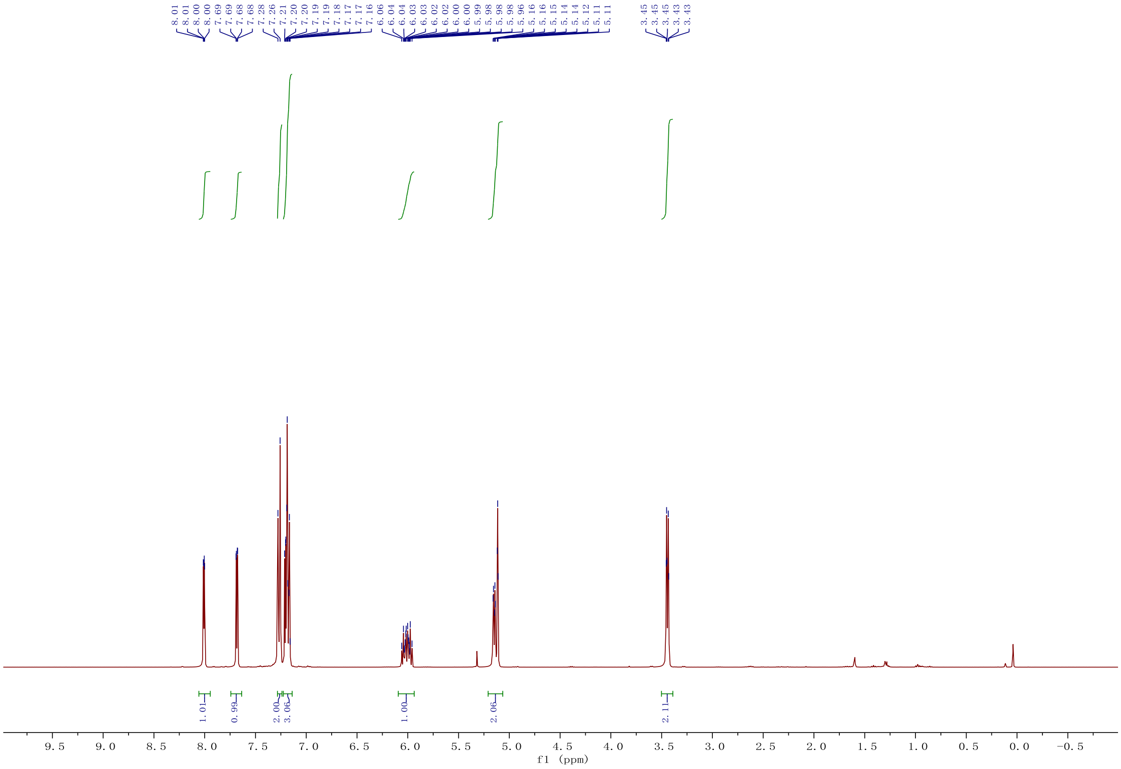

Supplementary Figure 24. ^{1}**H NMR** (400 MHz, Chloroform-*d*) of **sub. 2n**

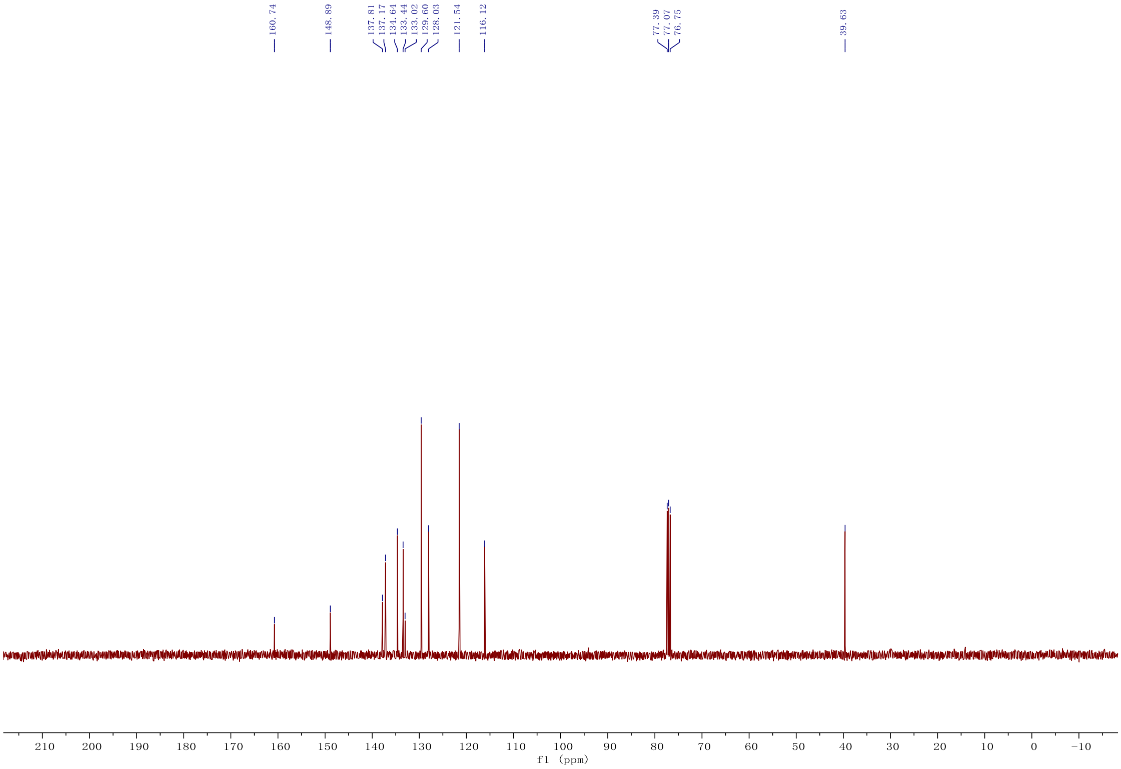

Supplementary Figure 25. ^{13}**C NMR** (101 MHz, Chloroform-*d*) of **sub. 2n**

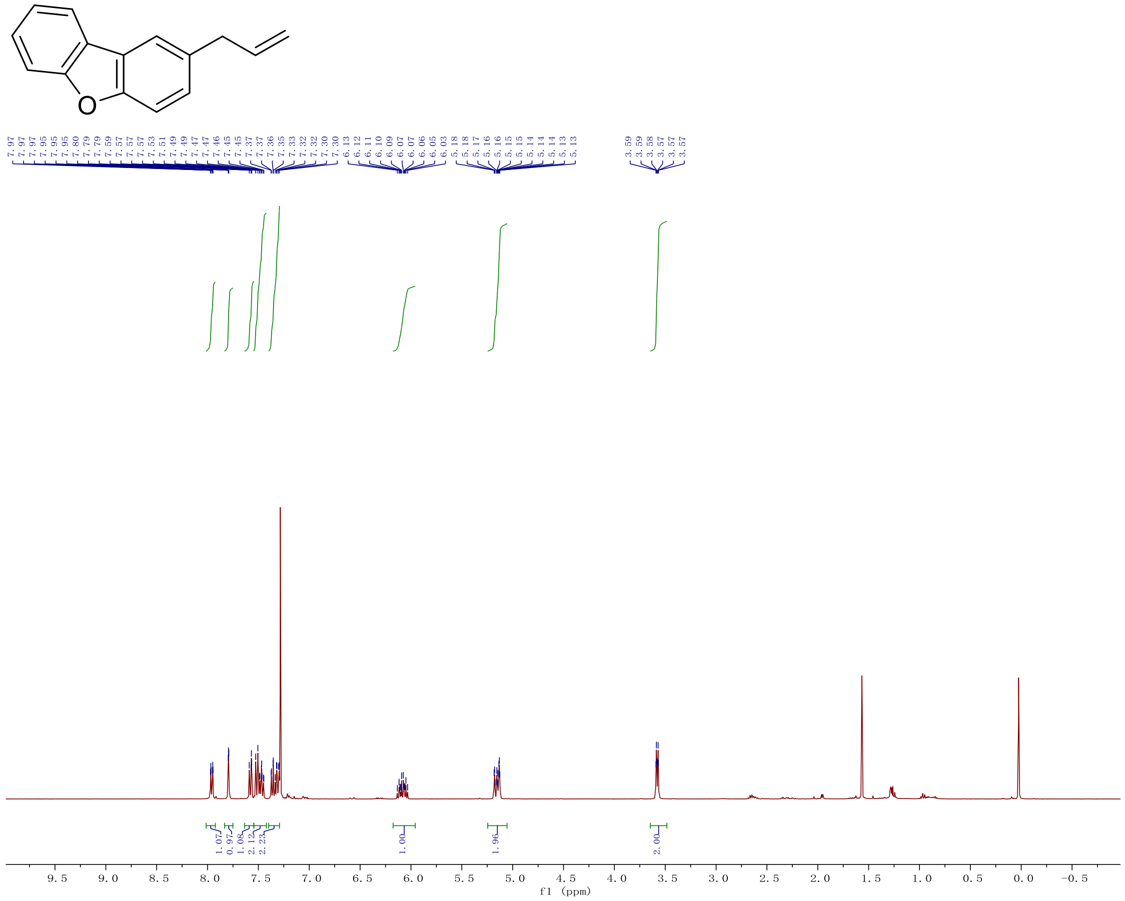

Supplementary Figure 26. **^{1}H NMR** (400 MHz, Chloroform-*d*) of **sub. 2p**

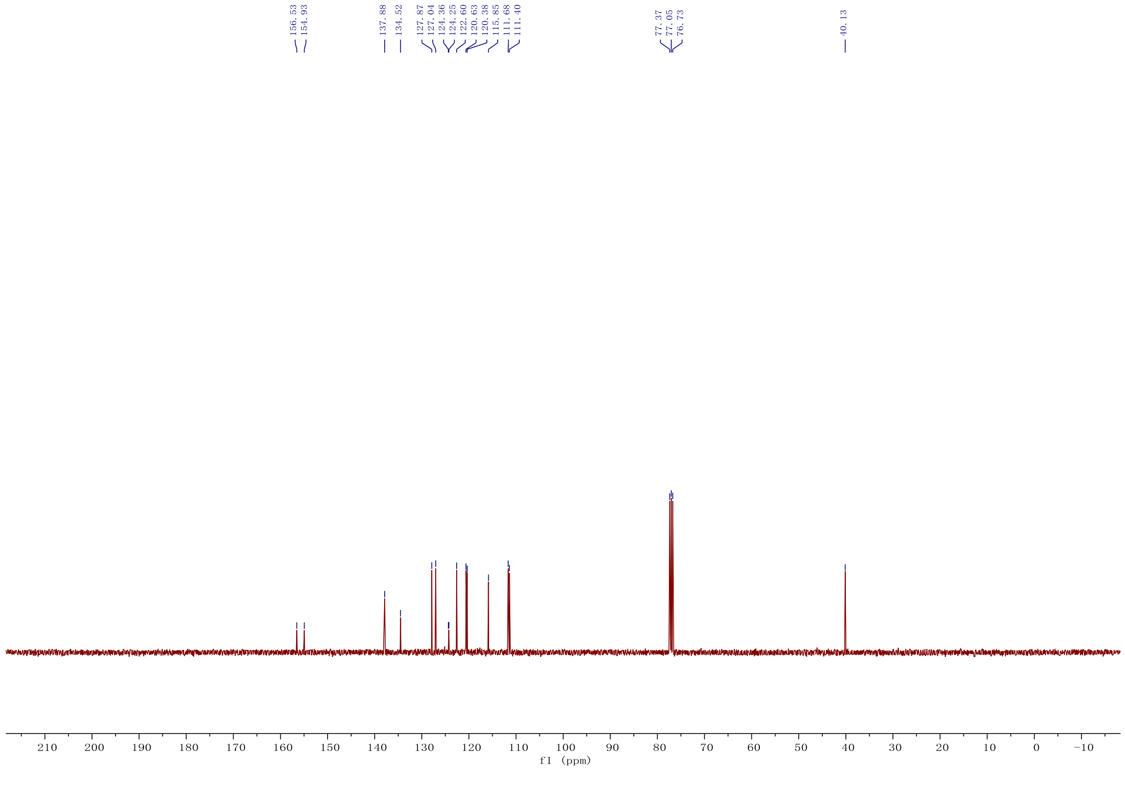

Supplementary Figure 27. **^{13}C NMR** (101 MHz, Chloroform-*d*) of **sub. 2p**

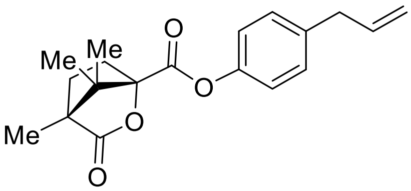

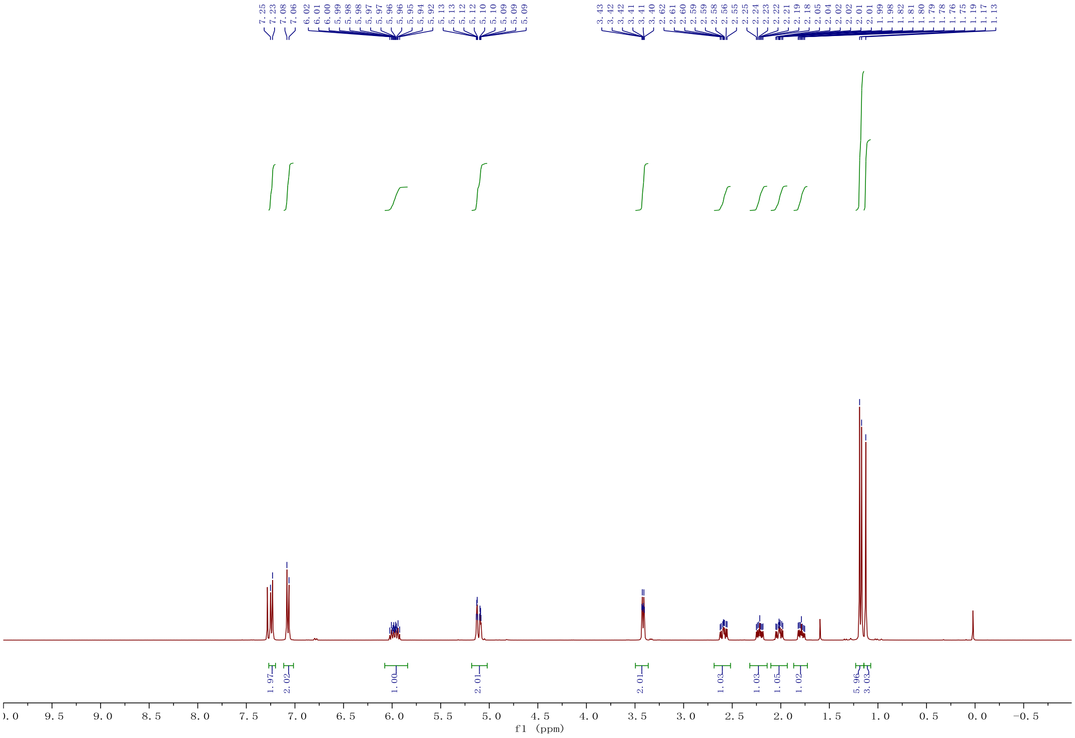

Supplementary Figure 28. ^{1}H NMR (400 MHz, Chloroform-*d*) of **sub. 2q**

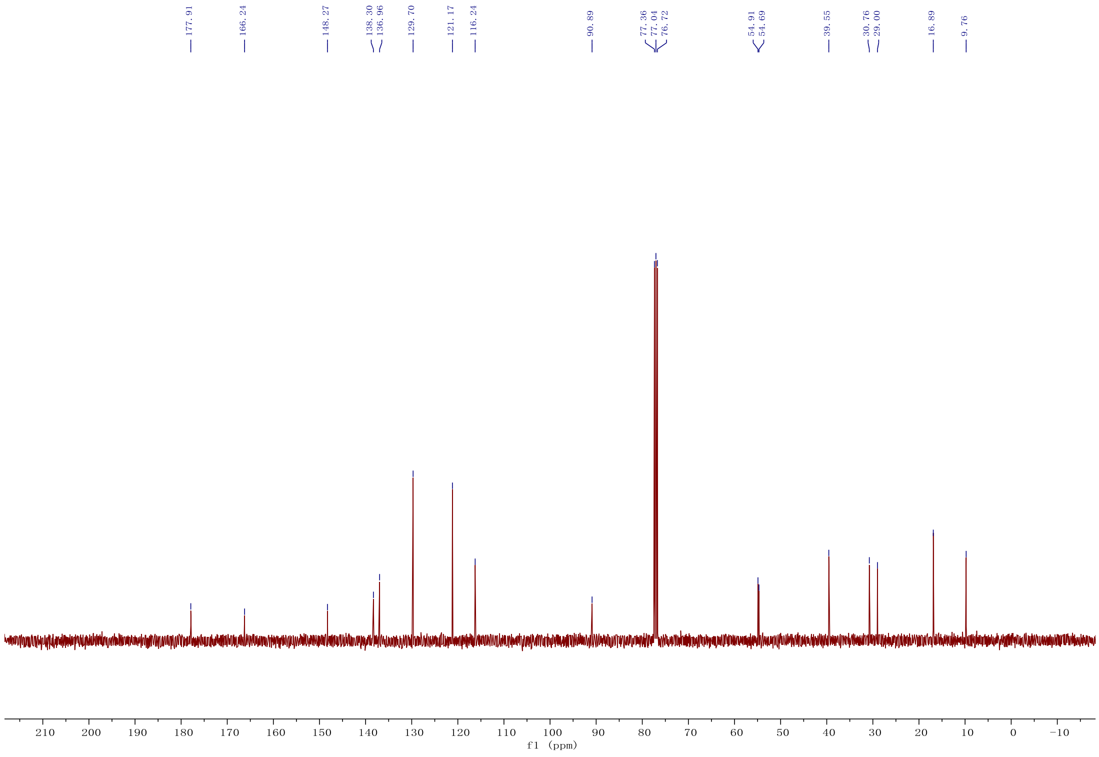

Supplementary Figure 29. ^{13}C NMR (101 MHz, Chloroform-*d*) of **sub. 2q**

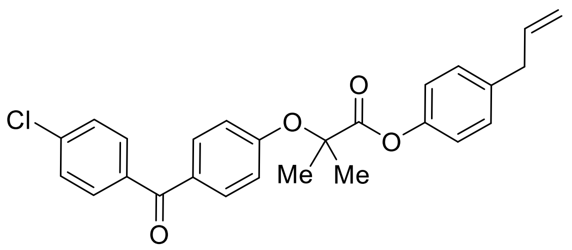

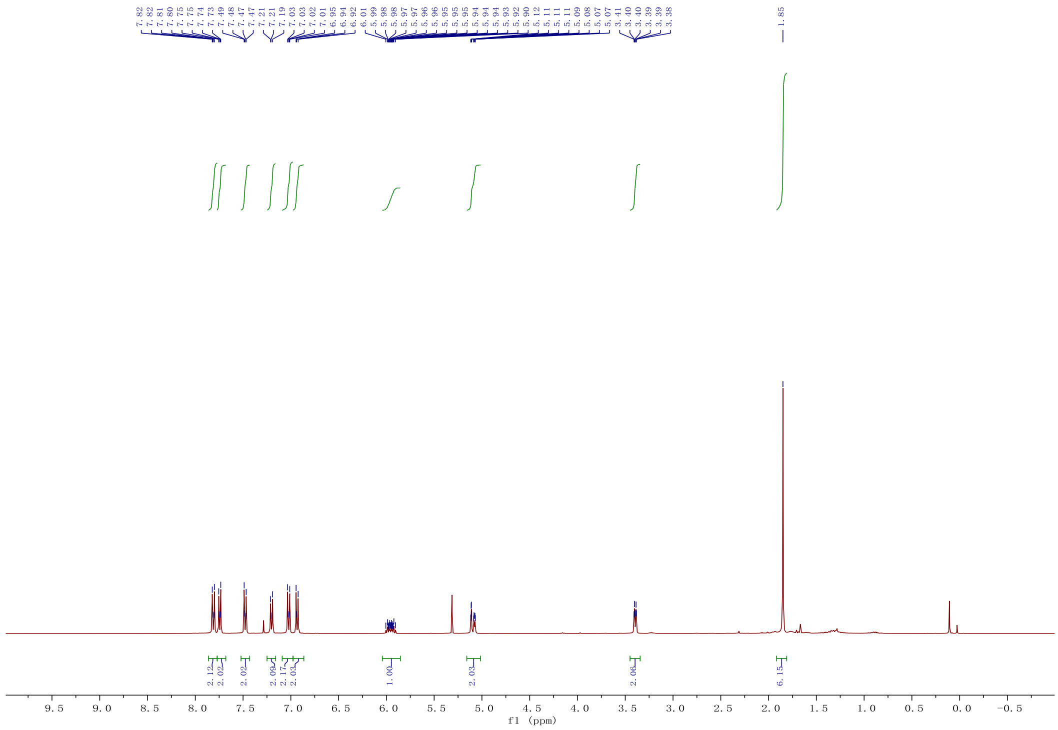

Supplementary Figure 30. **^{1}H NMR** (400 MHz, Chloroform-*d*) of **sub. 2r**

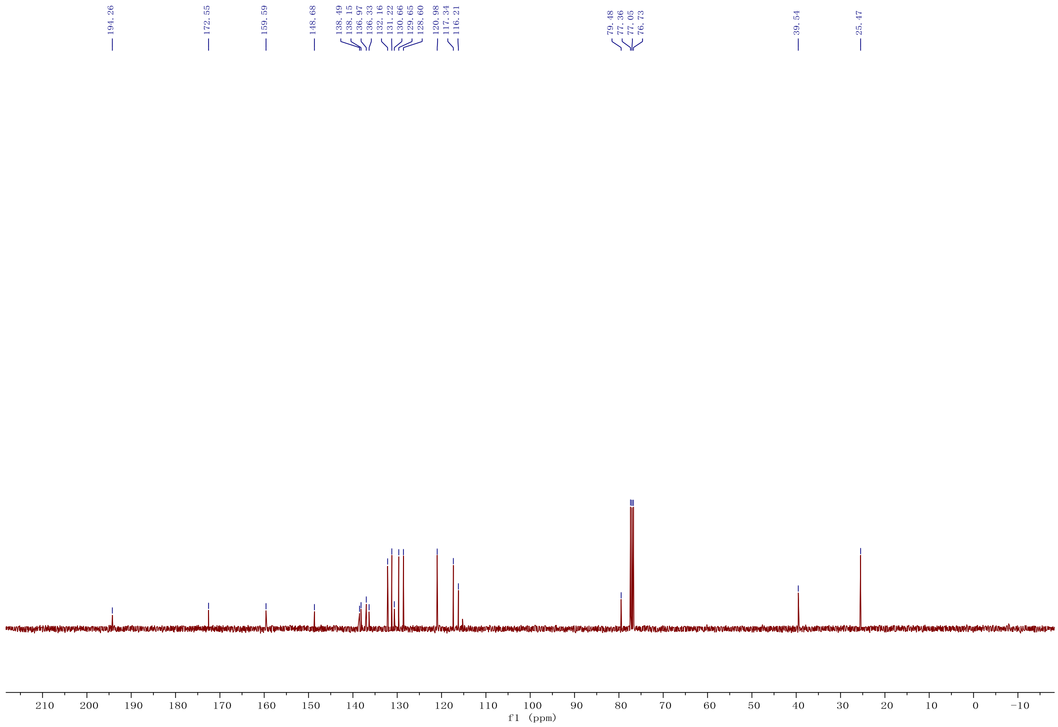

Supplementary Figure 31. **^{13}C NMR** (101 MHz, Chloroform-*d*) of **sub. 2r**

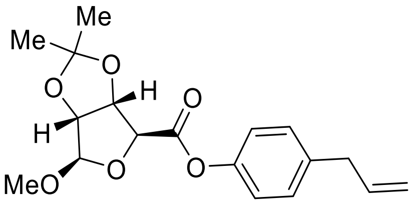

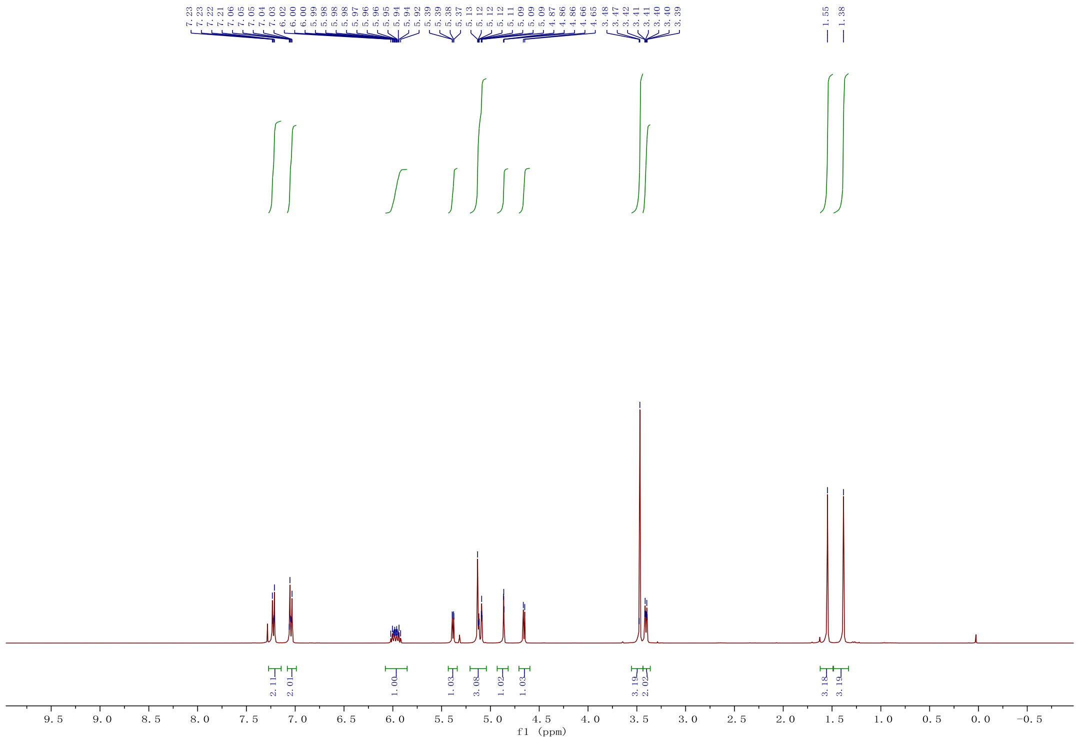

Supplementary Figure 32. ^{1}H NMR (400 MHz, Chloroform-*d*) of **sub. 2s**

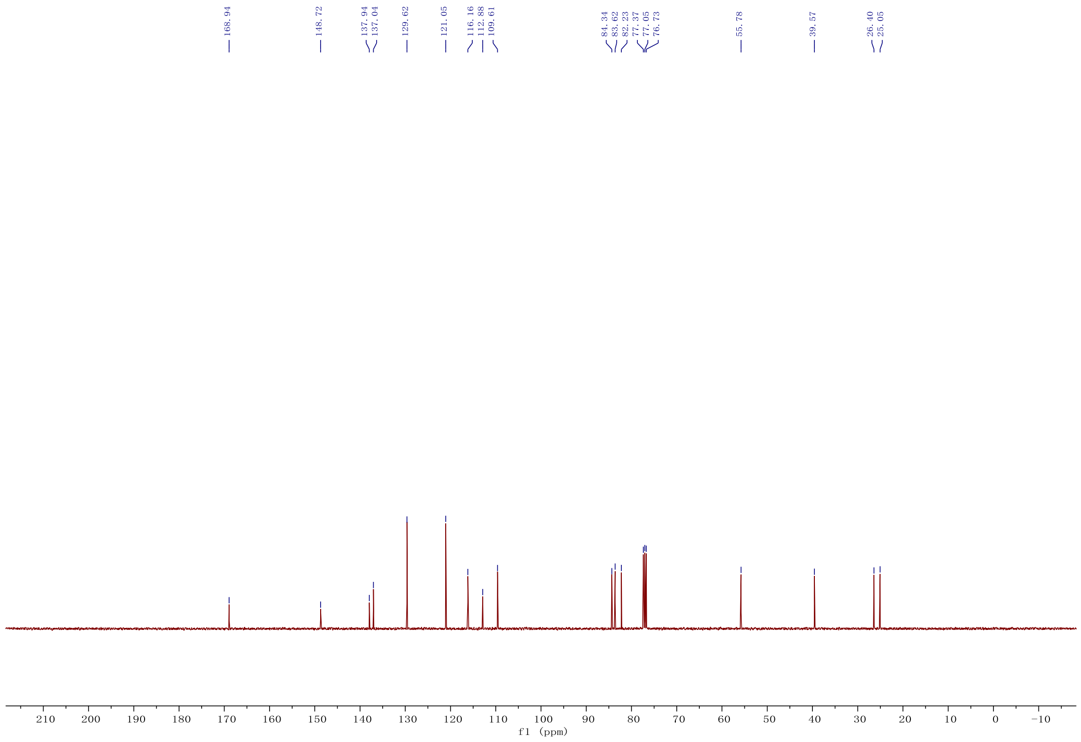

Supplementary Figure 33. ^{13}C NMR (101 MHz, Chloroform-*d*) of **sub. 2s**

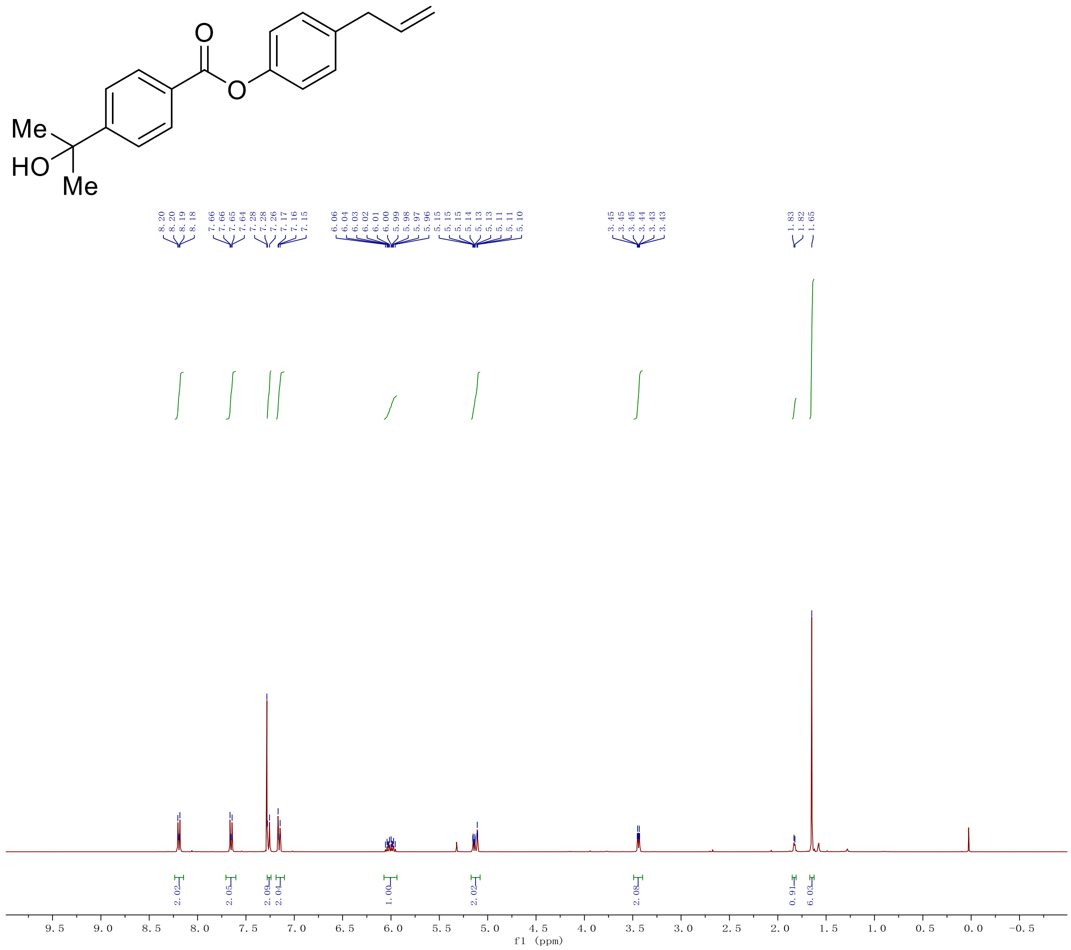

Supplementary Figure 34. ^{1}H NMR (400 MHz, Chloroform-*d*) of **sub. 2t**

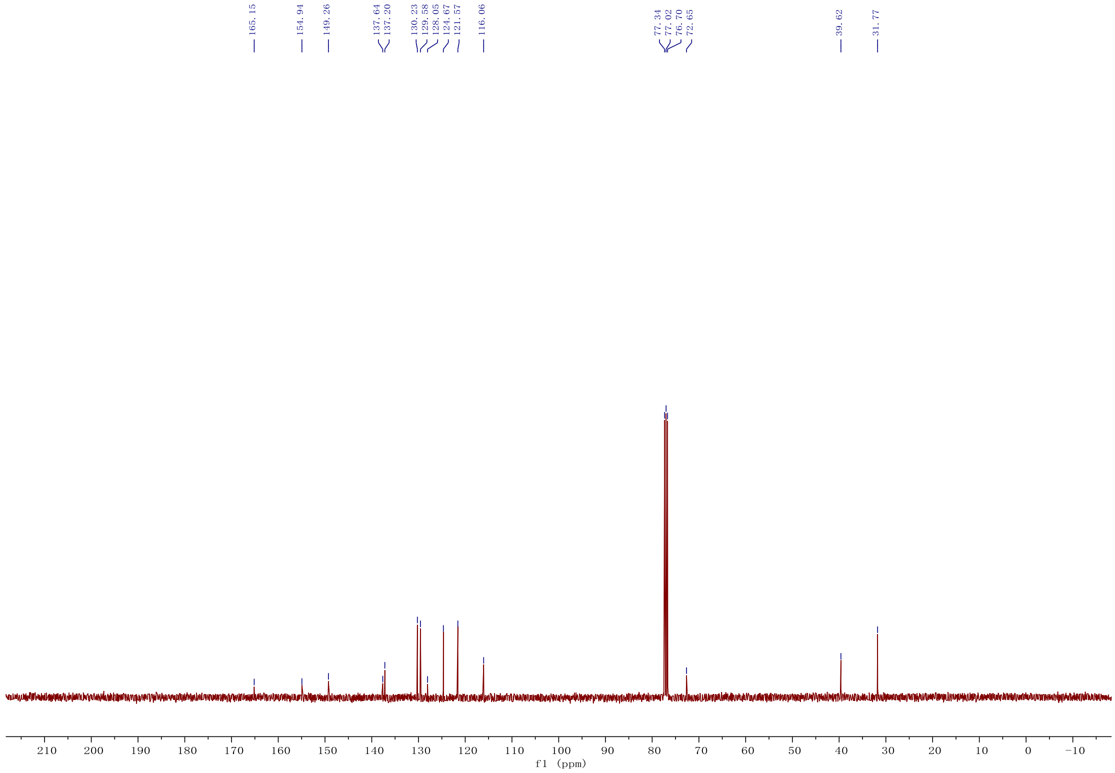

Supplementary Figure 35. ^{13}C NMR (101 MHz, Chloroform-*d*) of **sub. 2t**

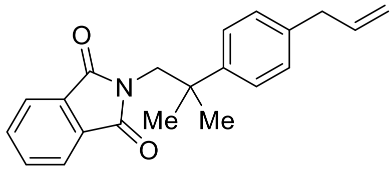

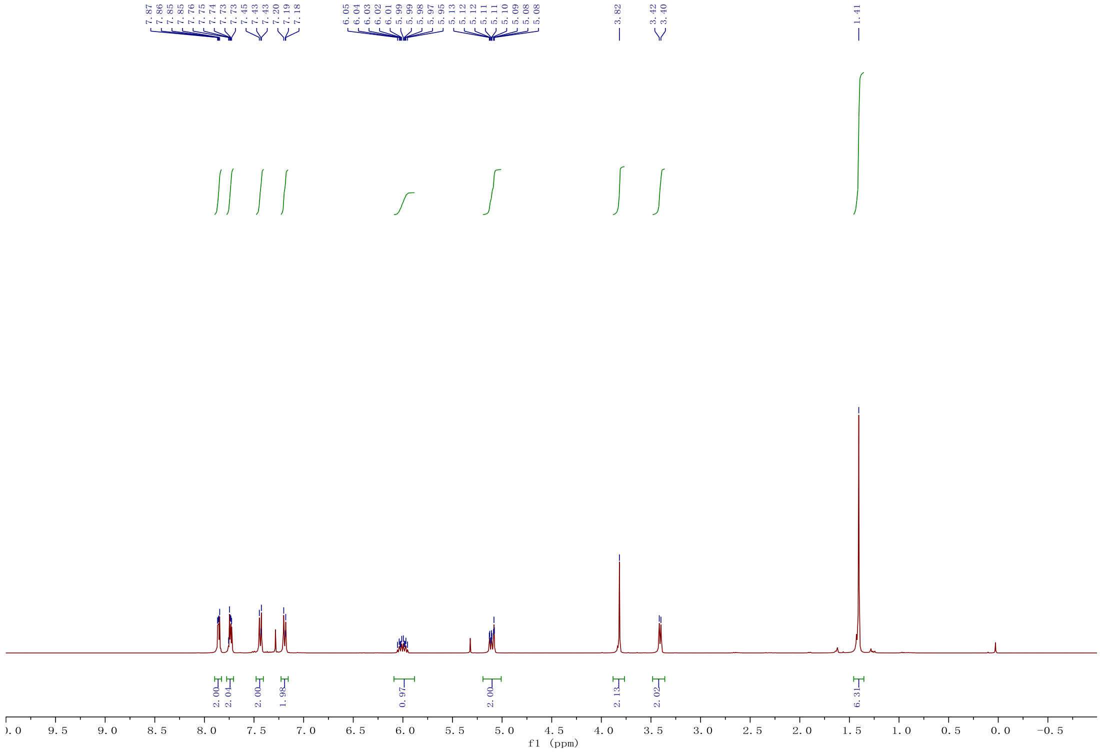

Supplementary Figure 36. ^{1}**H NMR** (400 MHz, Chloroform-*d*) of **sub. 2u**

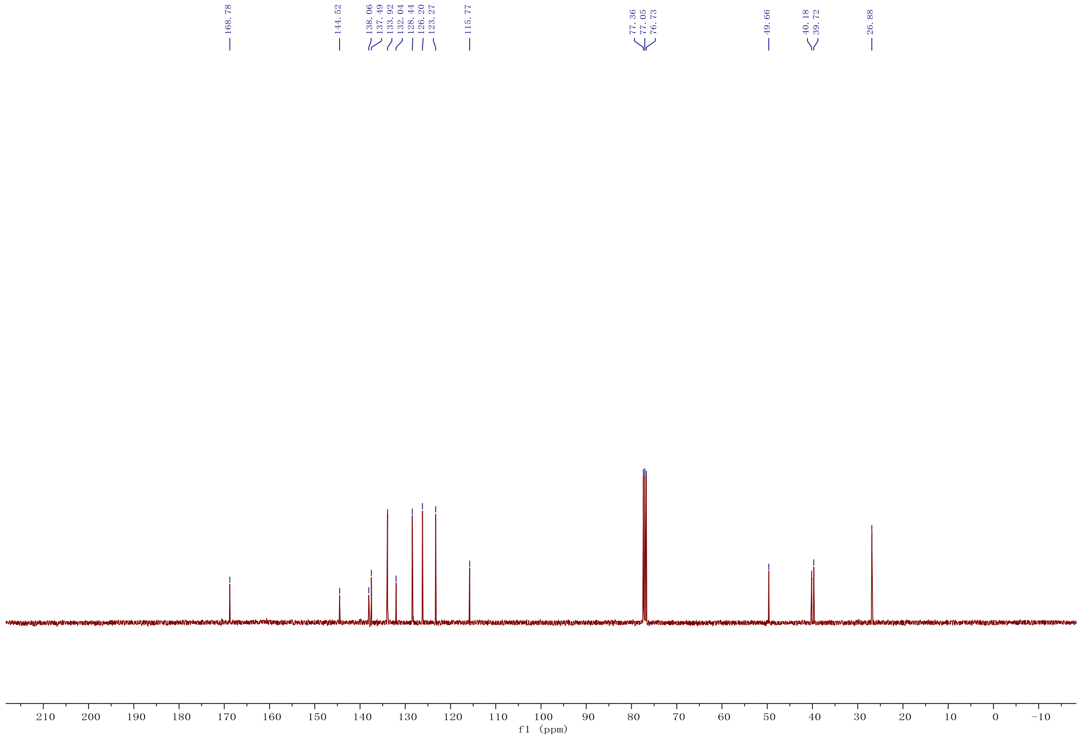

Supplementary Figure 37. ^{13}**C NMR** (101 MHz, Chloroform-*d*) of **sub. 2u**

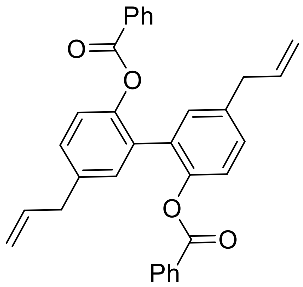

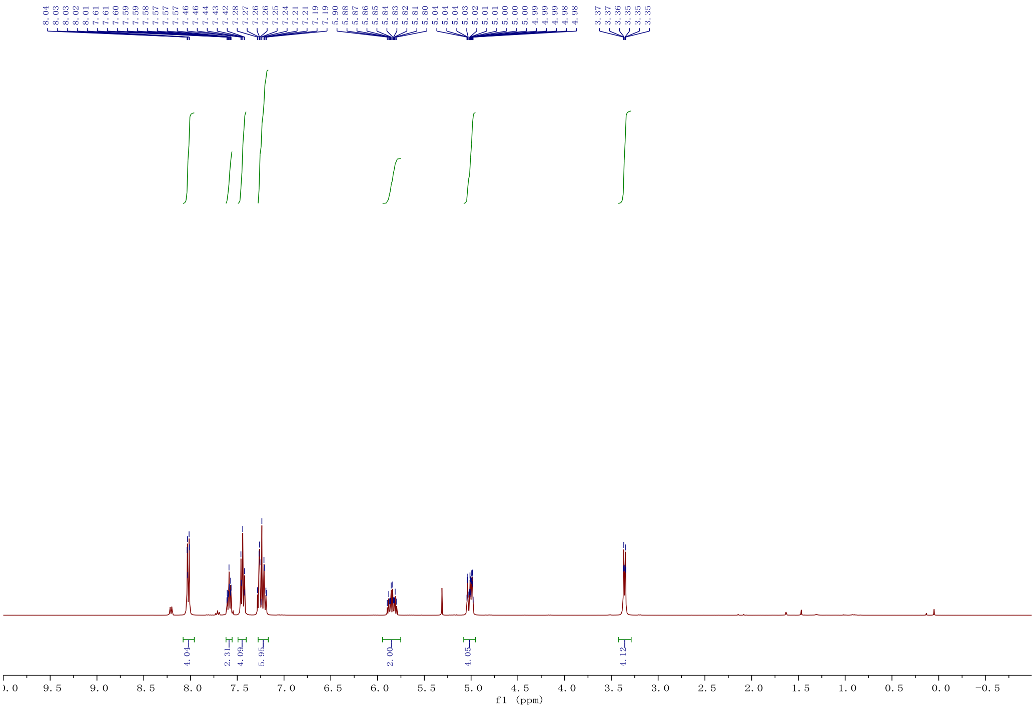

Supplementary Figure 38. **^{1}H NMR** (400 MHz, Chloroform-*d*) of **sub. 2v**

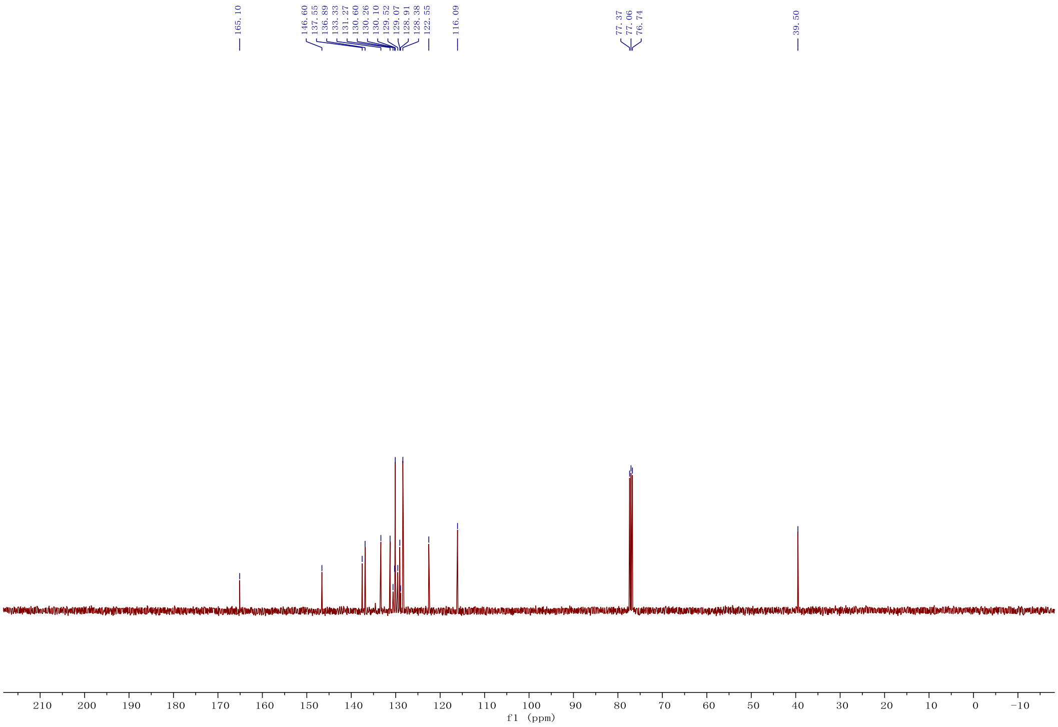

Supplementary Figure 39. **^{13}C NMR** (101 MHz, Chloroform-*d*) of **sub. 2v**

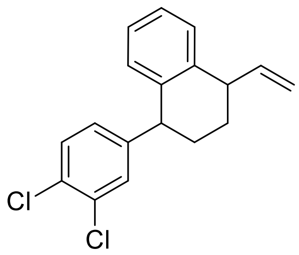

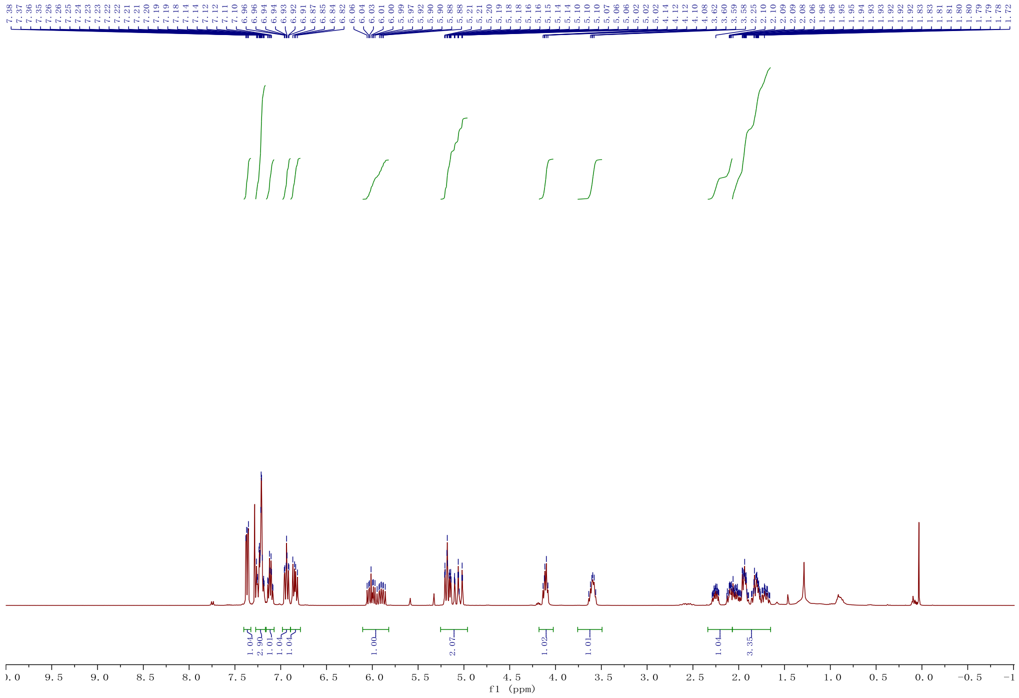

Supplementary Figure 40. ^{1}H NMR (400 MHz, Chloroform-*d*) of **sub. 2y**

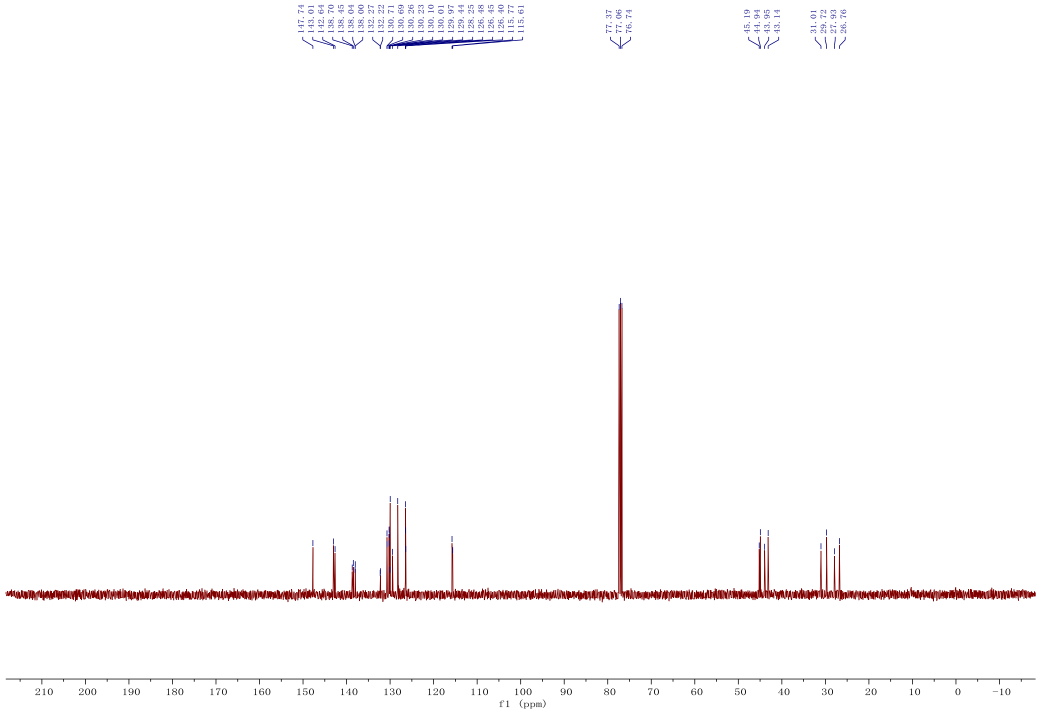

Supplementary Figure 41. ^{13}C NMR (101 MHz, Chloroform-*d*) of **sub. 2y**

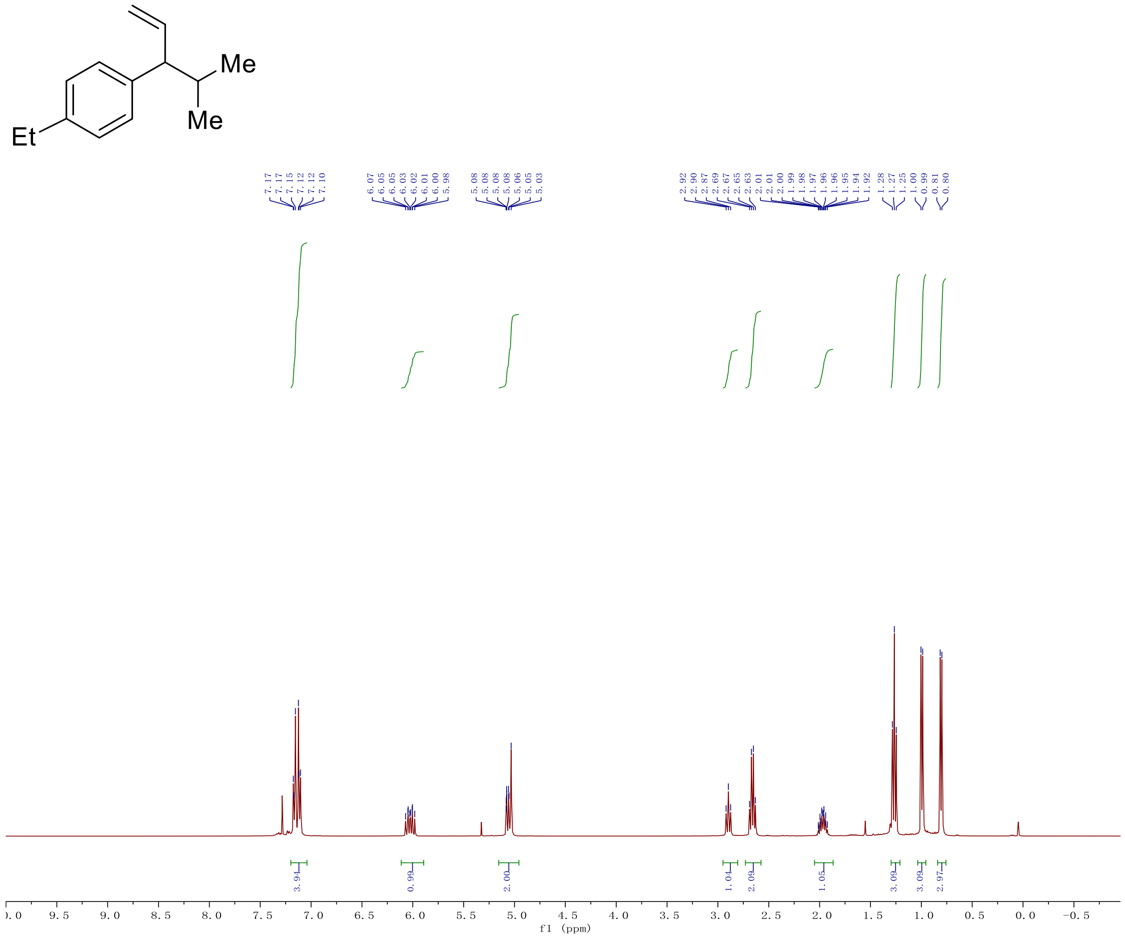

Supplementary Figure 42. ^{1}**H NMR** (400 MHz, Chloroform-*d*) of **sub. 2aa**

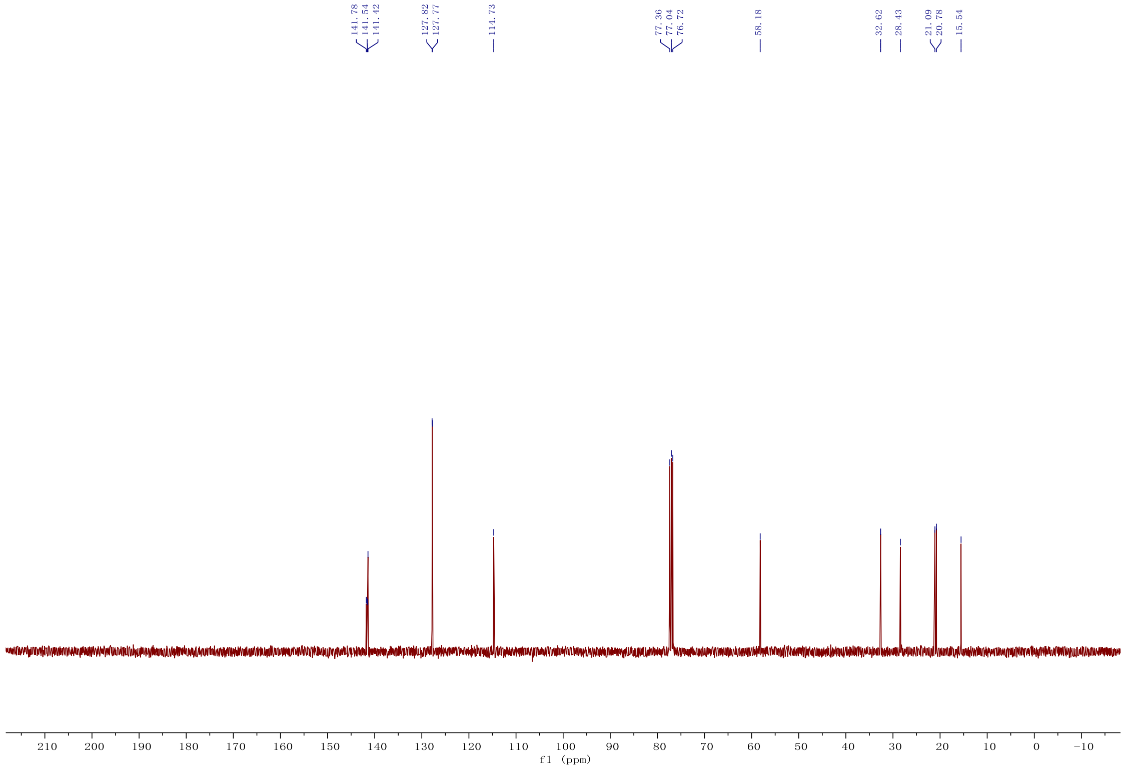

Supplementary Figure 43. ^{13}**C NMR** (101 MHz, Chloroform-*d*) of **sub. 2aa**

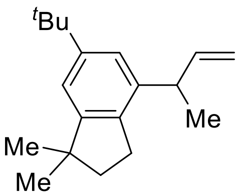

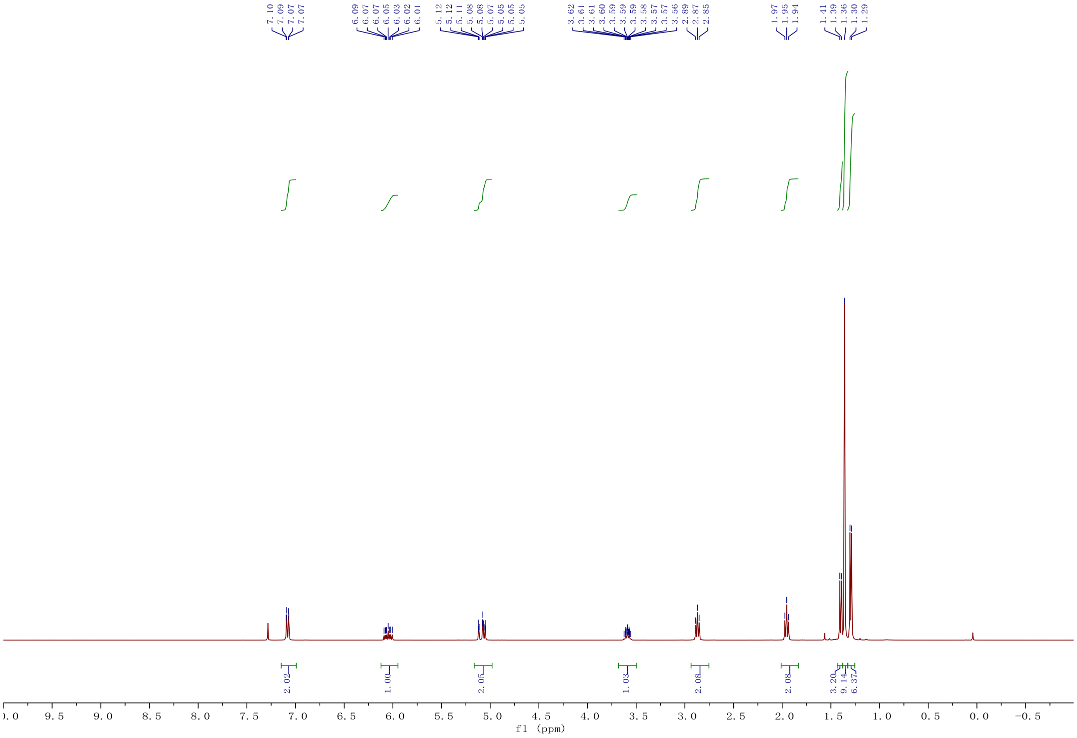

Supplementary Figure 44. ^{1}**H NMR** (400 MHz, Chloroform-*d*) of **sub. 2ab**

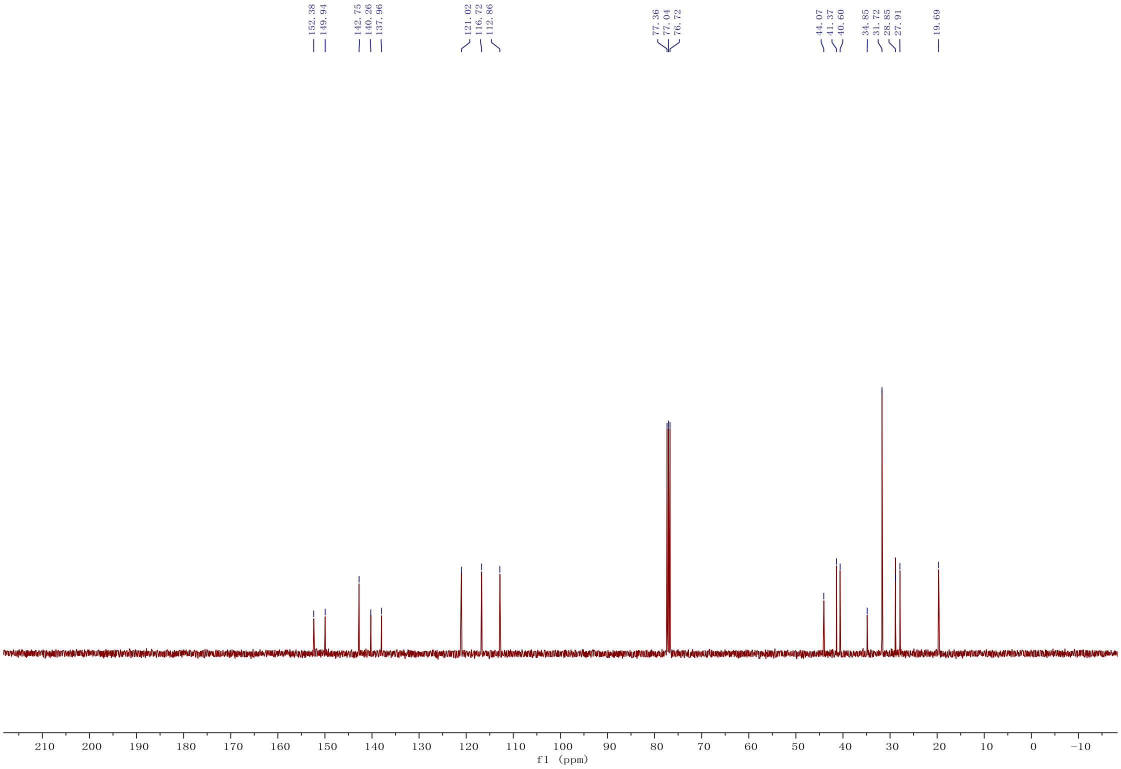

Supplementary Figure 45. ^{13}**C NMR** (101 MHz, Chloroform-*d*) of **sub. 2ab**

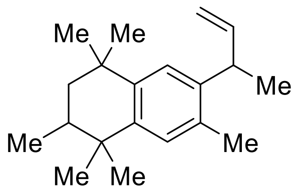

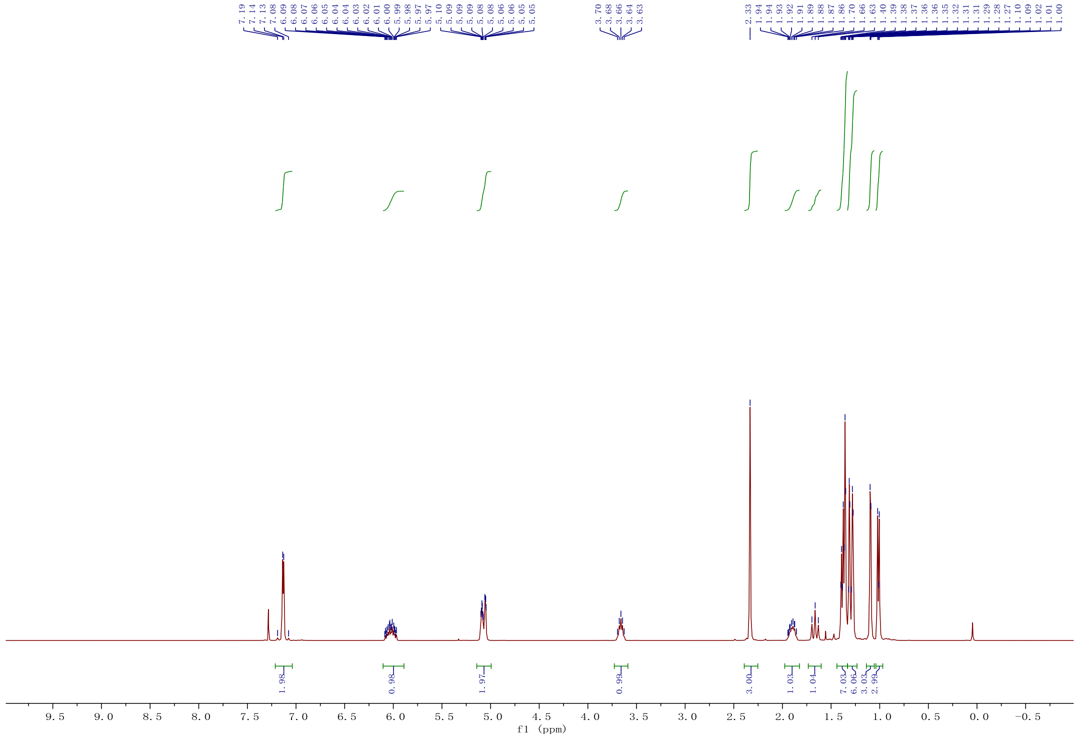

Supplementary Figure 46. ^{1}**H NMR** (400 MHz, Chloroform-*d*) of **sub. 2ac**

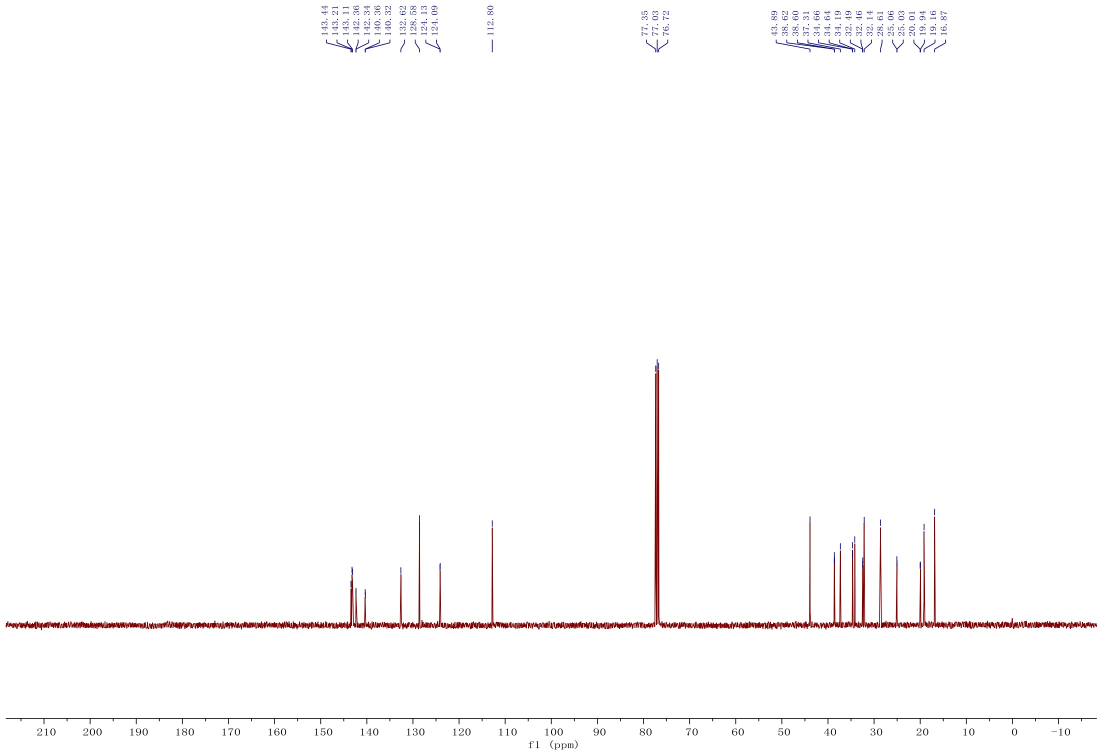

Supplementary Figure 47. ^{13}**C NMR** (101 MHz, Chloroform-*d*) of **sub. 2ac**

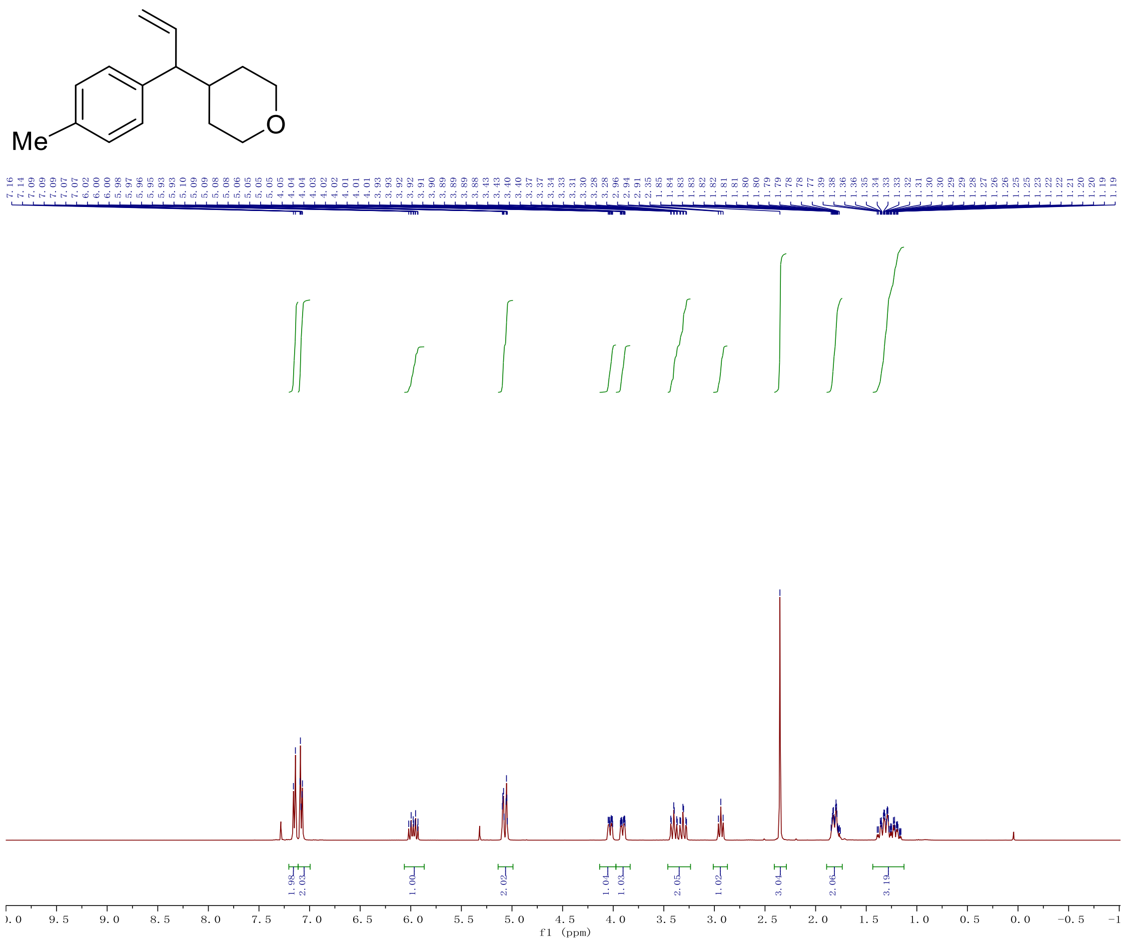

Supplementary Figure 48. ^{1}H NMR (400 MHz, Chloroform-*d*) of **sub. 2ad**

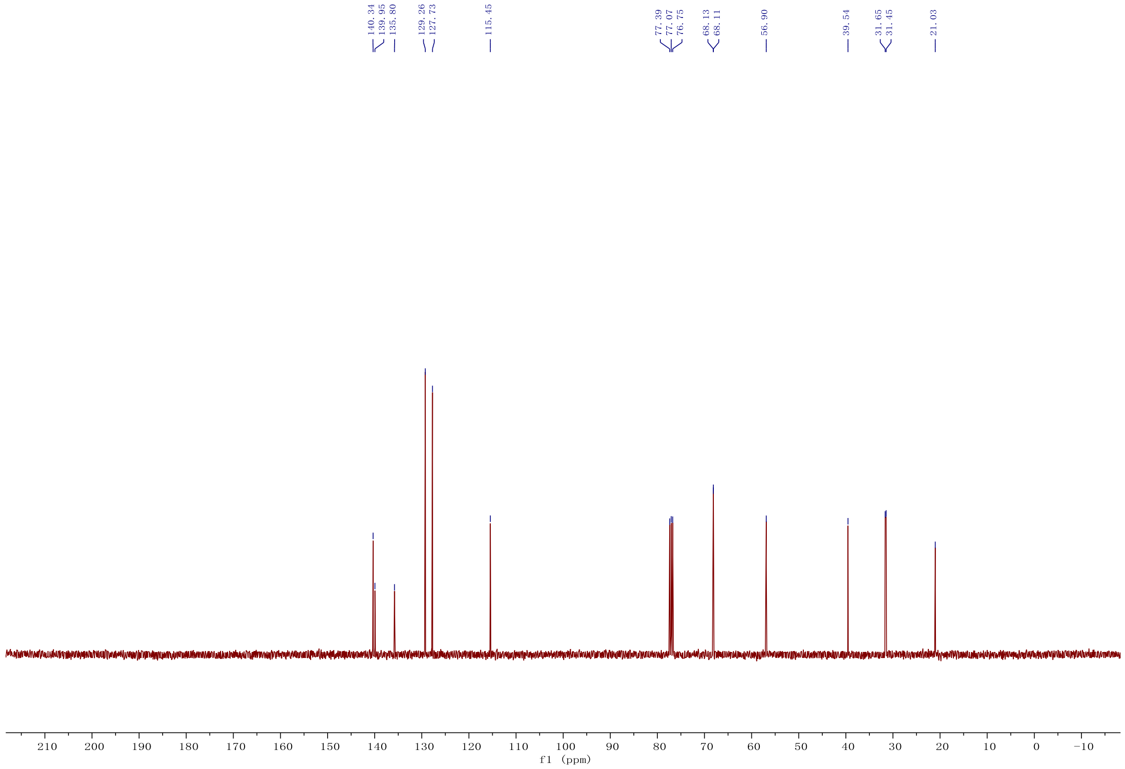

Supplementary Figure 49. ^{13}C NMR (101 MHz, Chloroform-*d*) of **sub. 2ad**

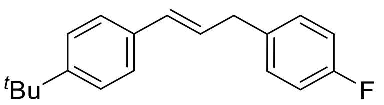

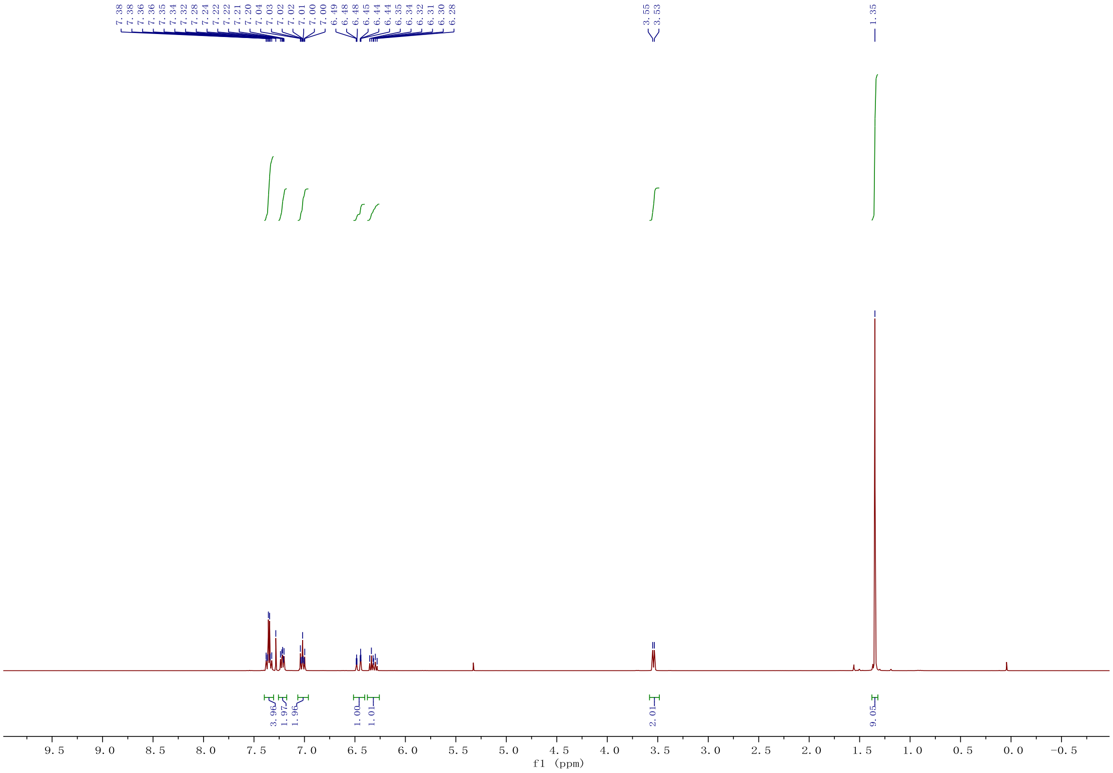

Supplementary Figure 50. ^{1}**H NMR** (400 MHz, Chloroform-*d*) of **sub. 2ah**

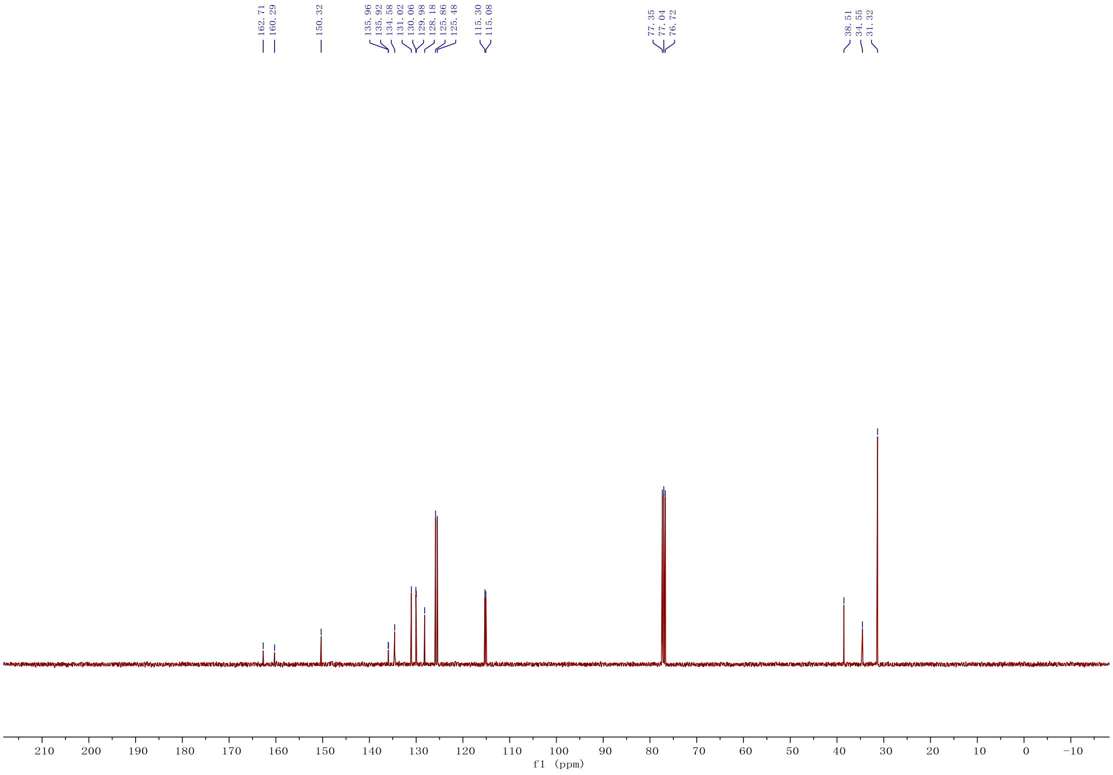

Supplementary Figure 51. ^{13}**C NMR** (101 MHz, Chloroform-*d*) of **sub. 2ah**

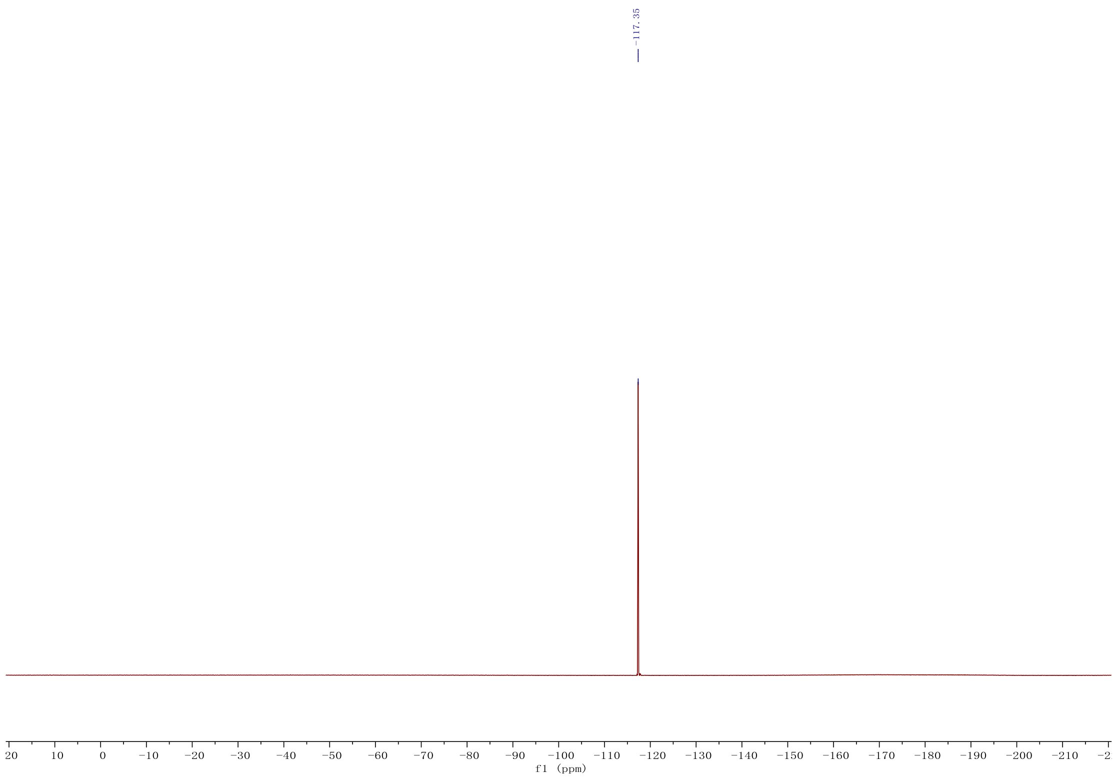

Supplementary Figure 52. ^{19}**F NMR** (376 MHz, Chloroform-*d*) of **sub. 2ah**

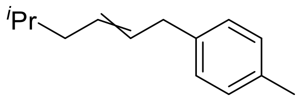

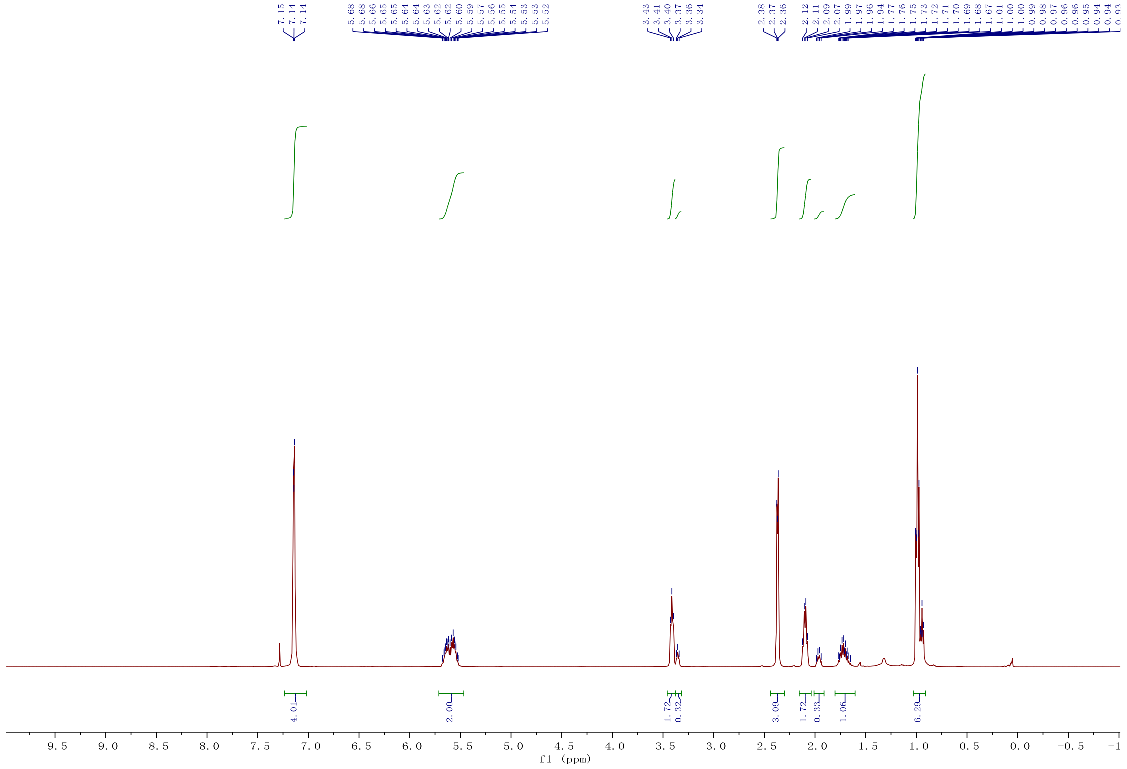

Supplementary Figure 53. ^{1}**H NMR** (400 MHz, Chloroform-*d*) of **sub. 3a**

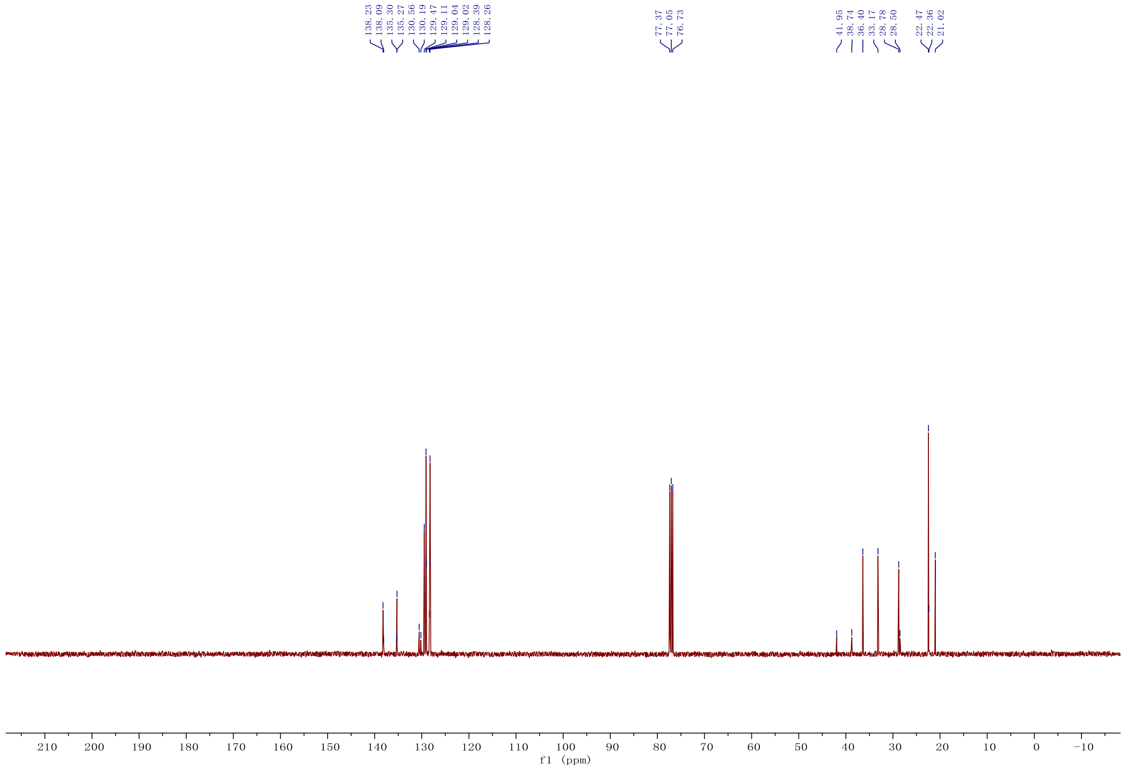

Supplementary Figure 54. ^{13}**C NMR** (101 MHz, Chloroform-*d*) of **sub. 3a**

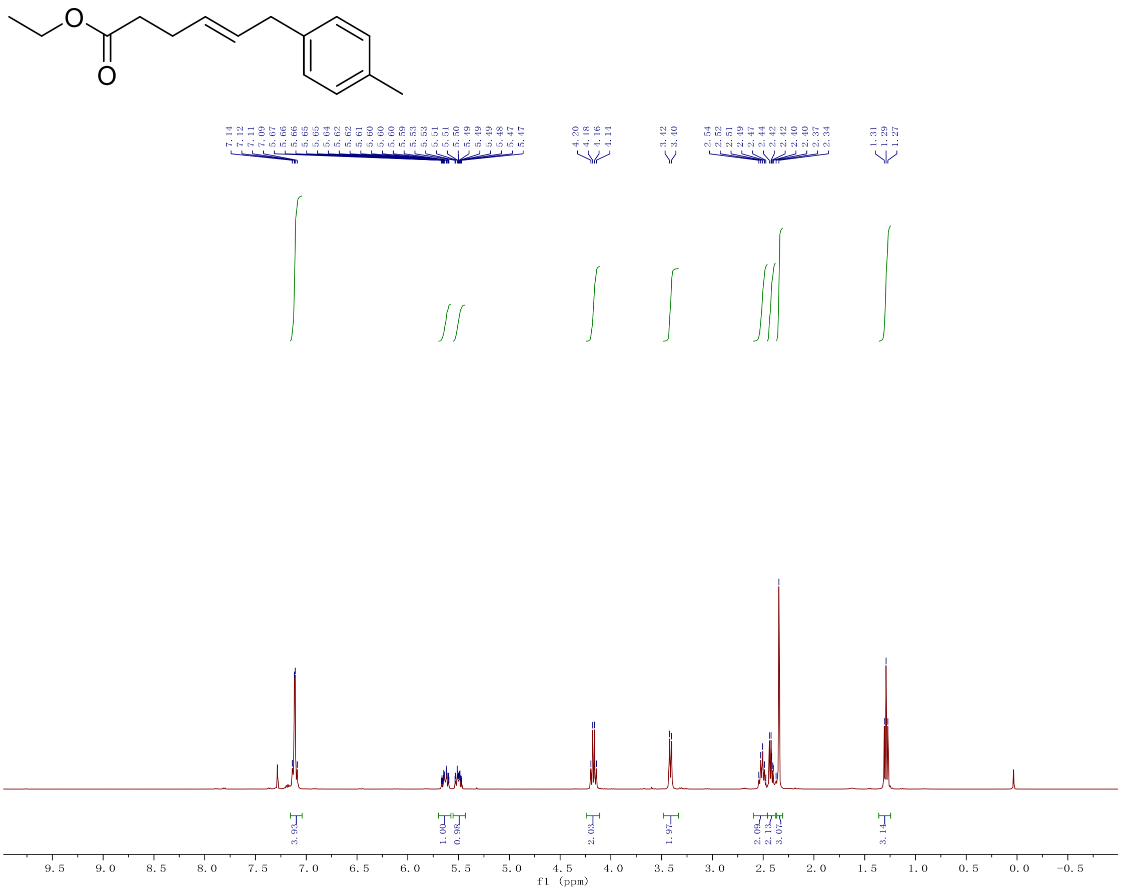

Supplementary Figure 55. ^{1}**H NMR** (400 MHz, Chloroform-*d*) of **sub. 3b**

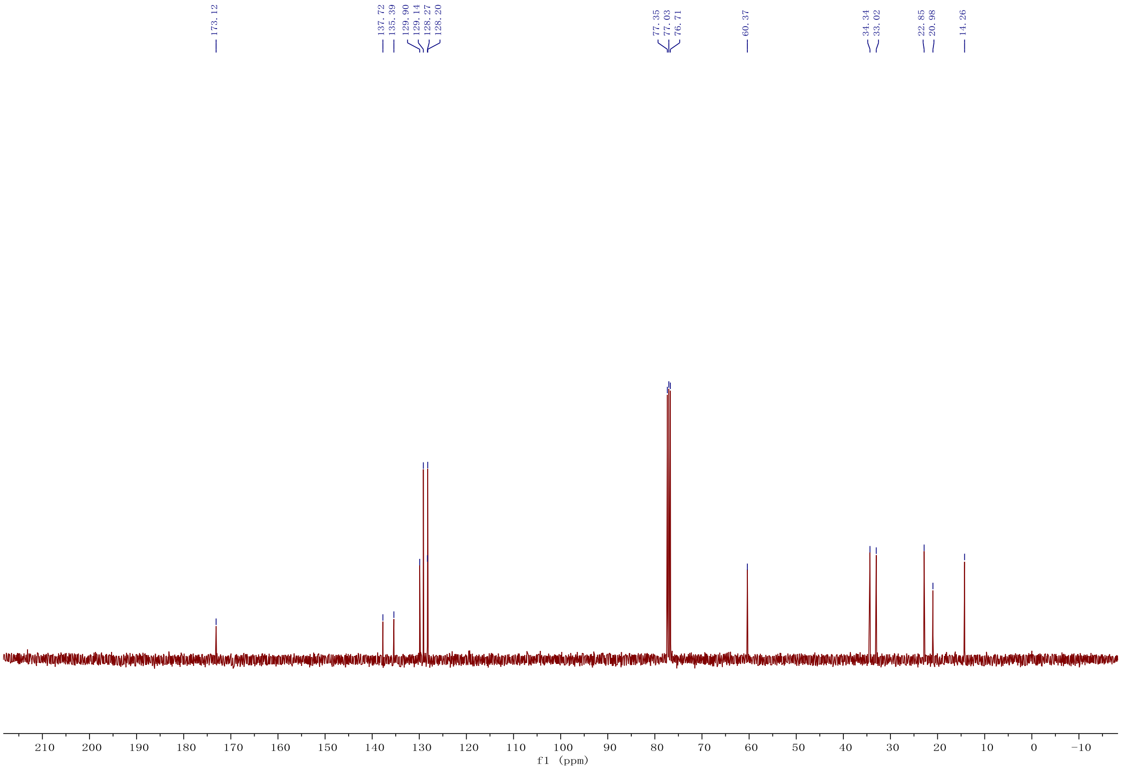

Supplementary Figure 56. ^{13}**C NMR** (101 MHz, Chloroform-*d*) of **sub. 3b**

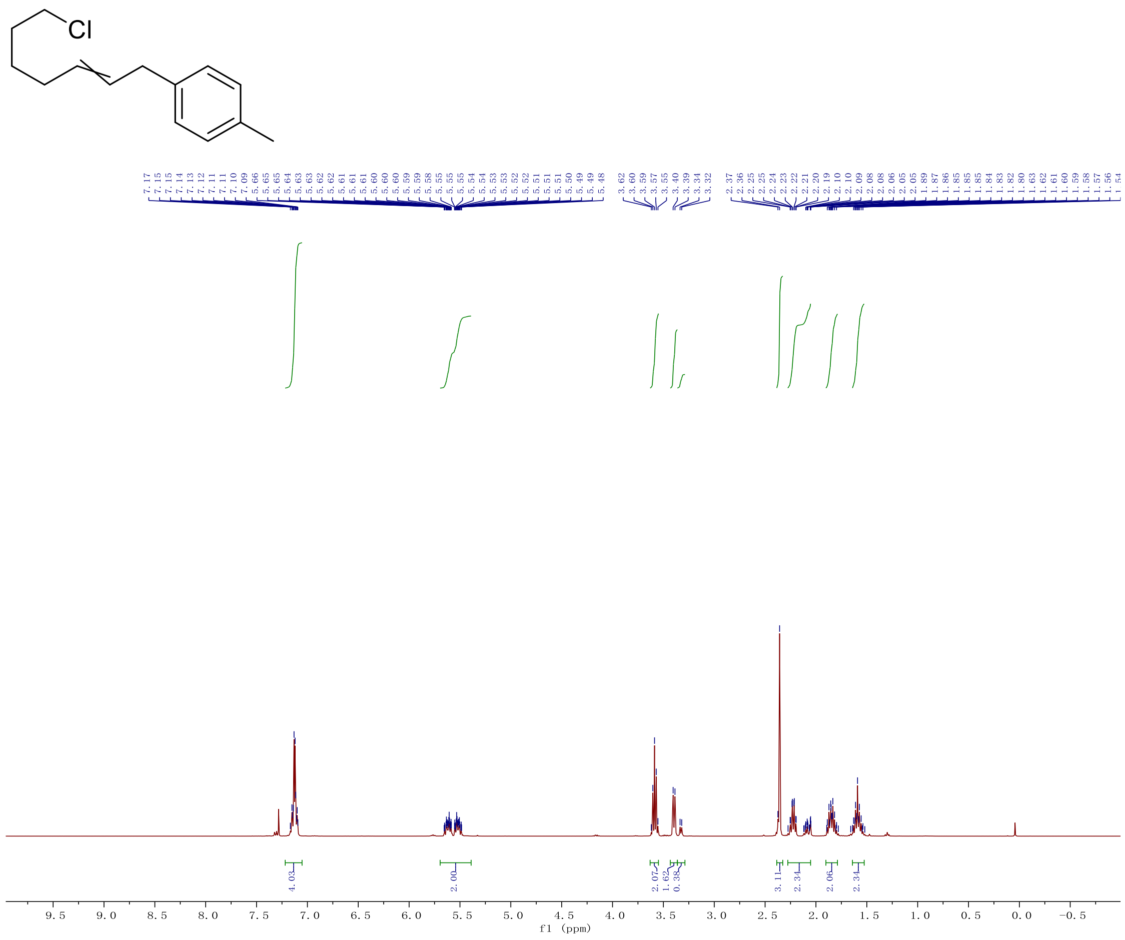

Supplementary Figure 57. **^{1}H NMR** (400 MHz, Chloroform-*d*) of **sub. 3c**

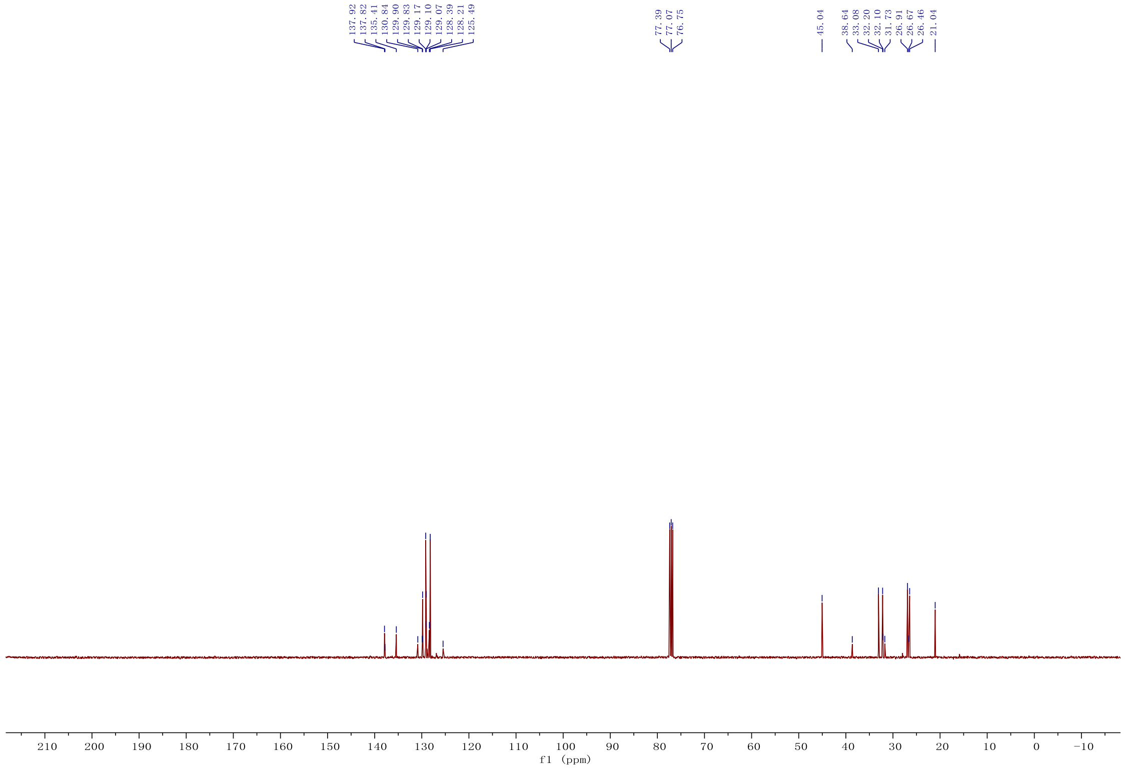

Supplementary Figure 58. **^{13}C NMR** (101 MHz, Chloroform-*d*) of **sub. 3c**

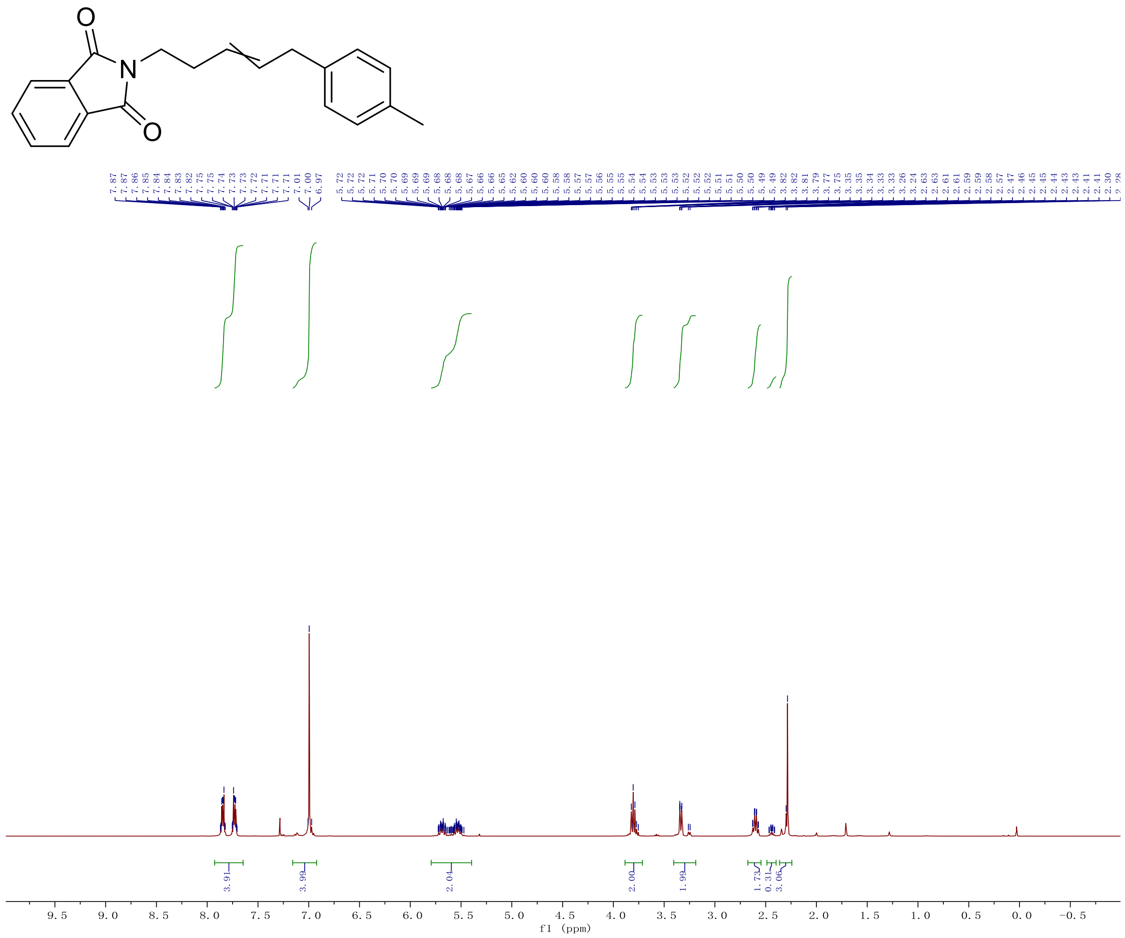

Supplementary Figure 59. **^{1}H NMR** (400 MHz, Chloroform-*d*) of **sub. 3d**

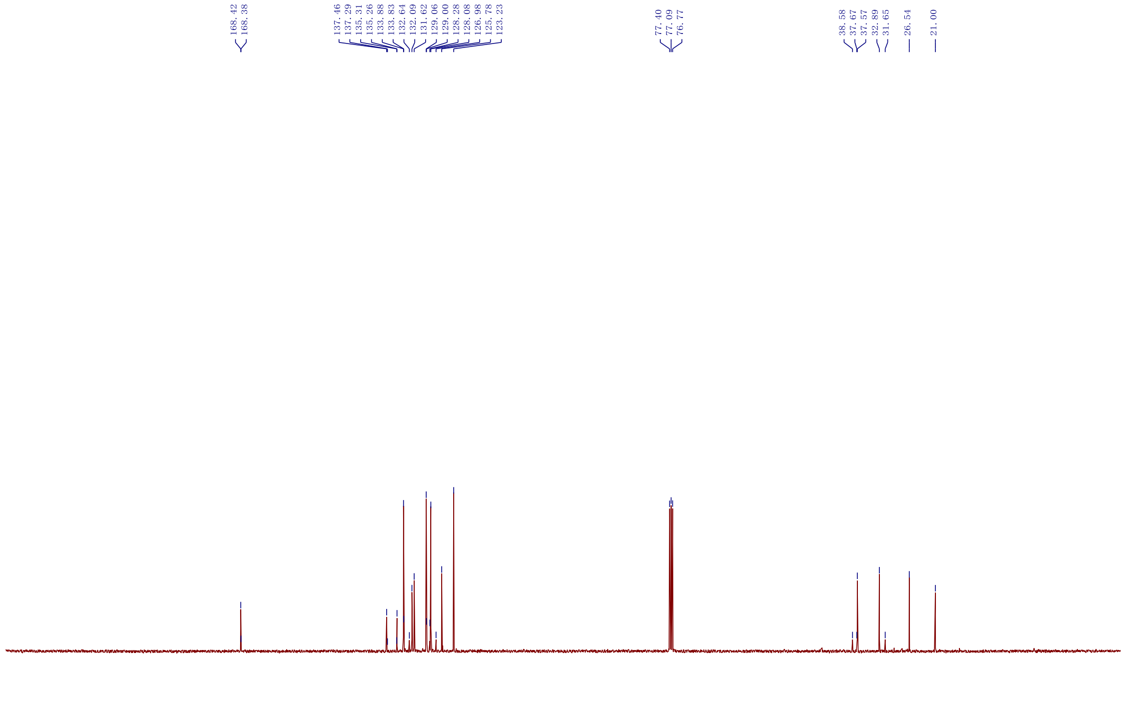

Supplementary Figure 60. **^{13}C NMR** (101 MHz, Chloroform-*d*) of **sub. 3d**

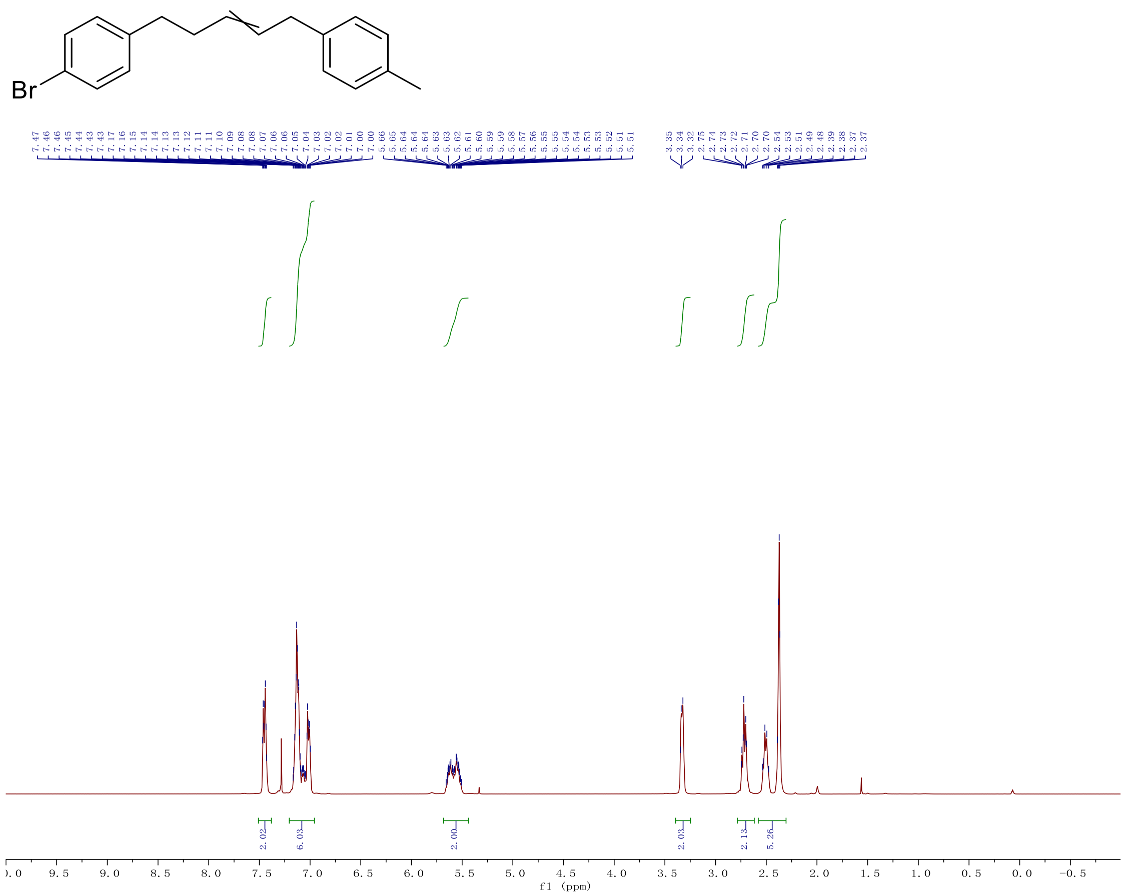

Supplementary Figure 61. ¹H NMR (400 MHz, Chloroform-*d*) of **sub. 3e**

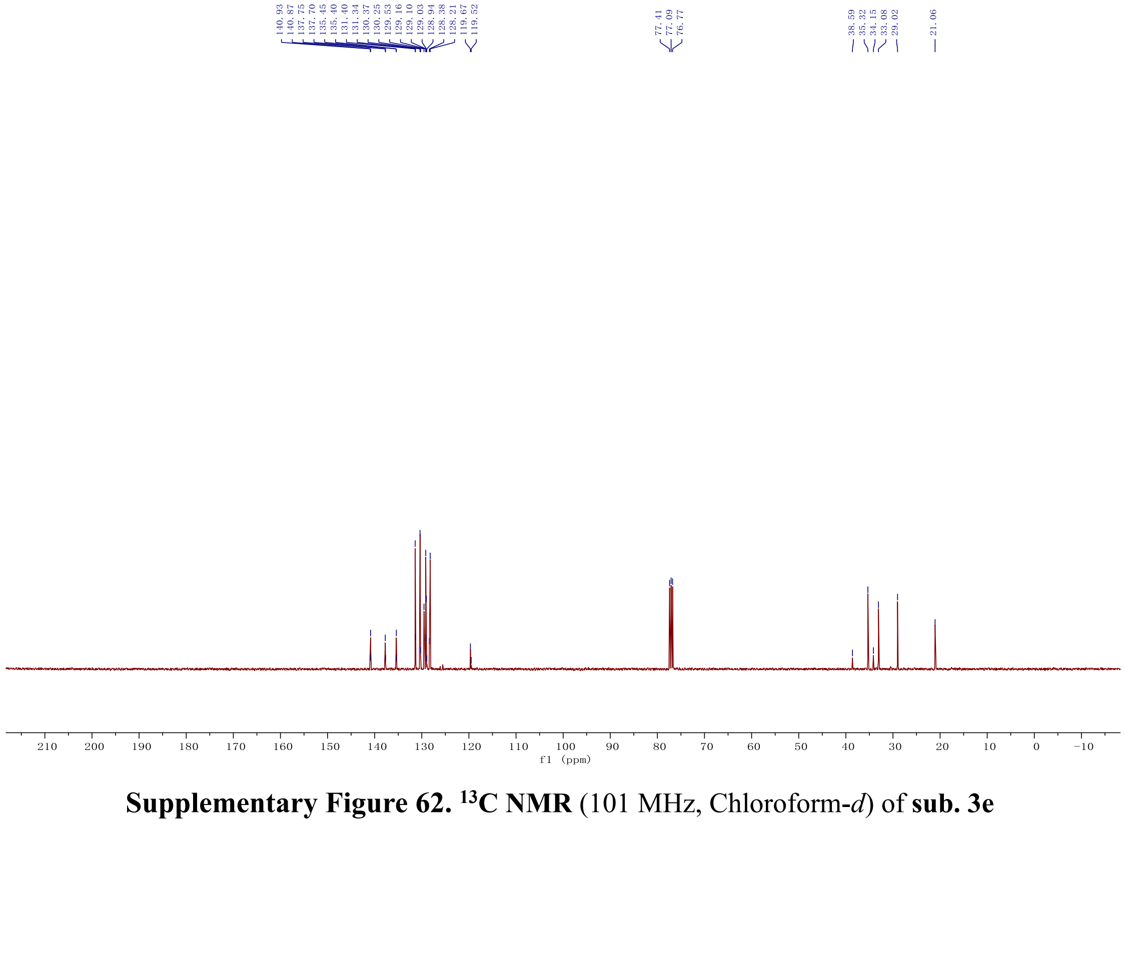

Supplementary Figure 62. ¹³C NMR (101 MHz, Chloroform-*d*) of **sub. 3e**

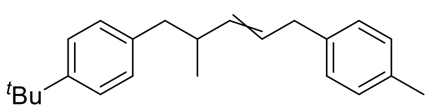

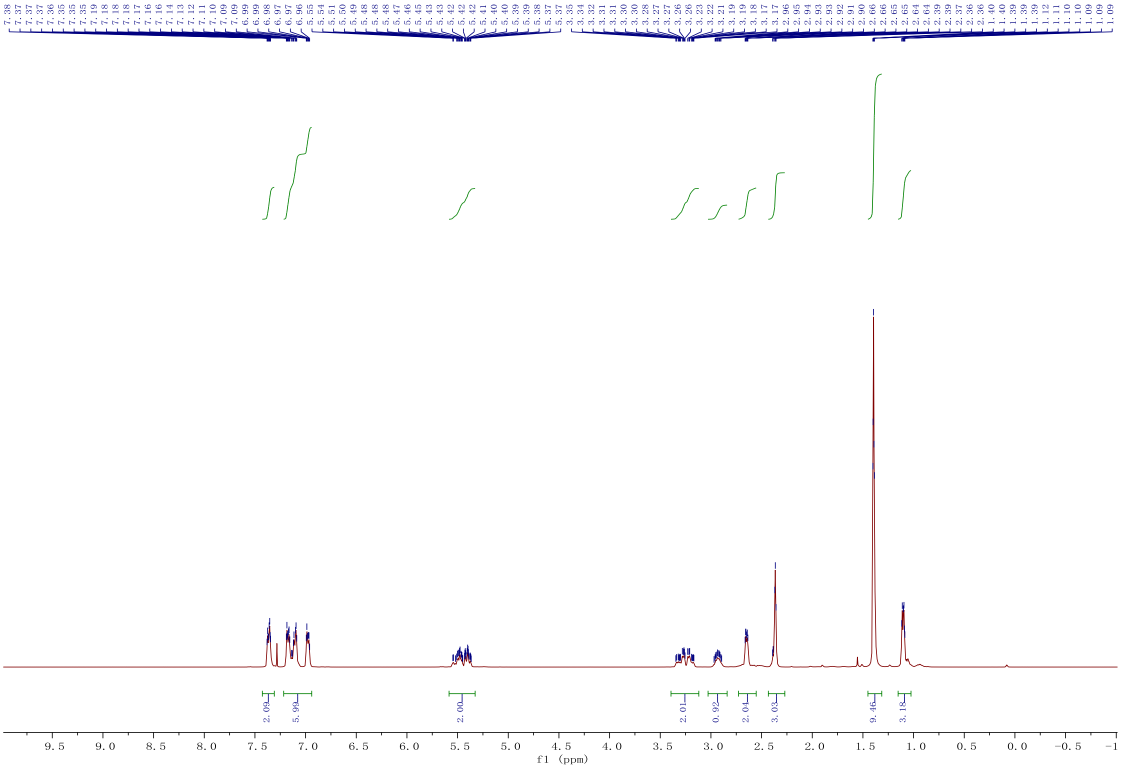

Supplementary Figure 63. ^{1}H NMR (400 MHz, Chloroform-*d*) of **sub. 3f**

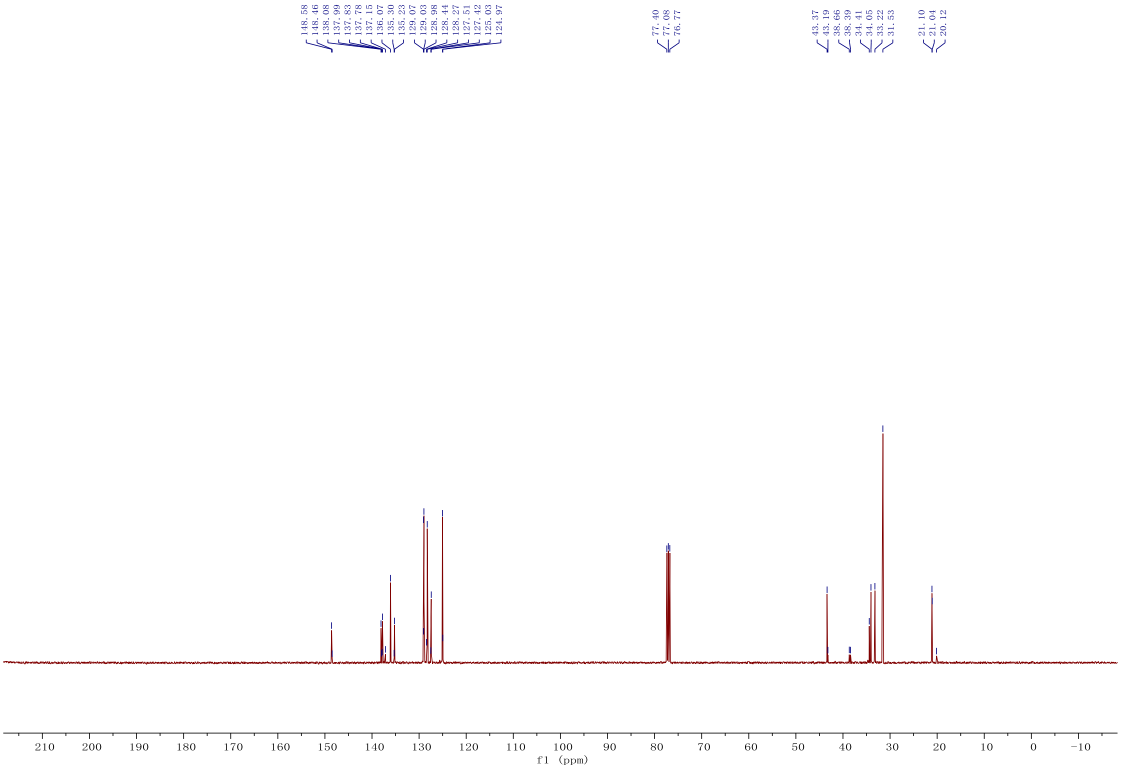

Supplementary Figure 64. ^{13}C NMR (101 MHz, Chloroform-*d*) of **sub. 3f**

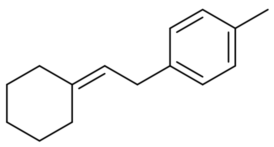

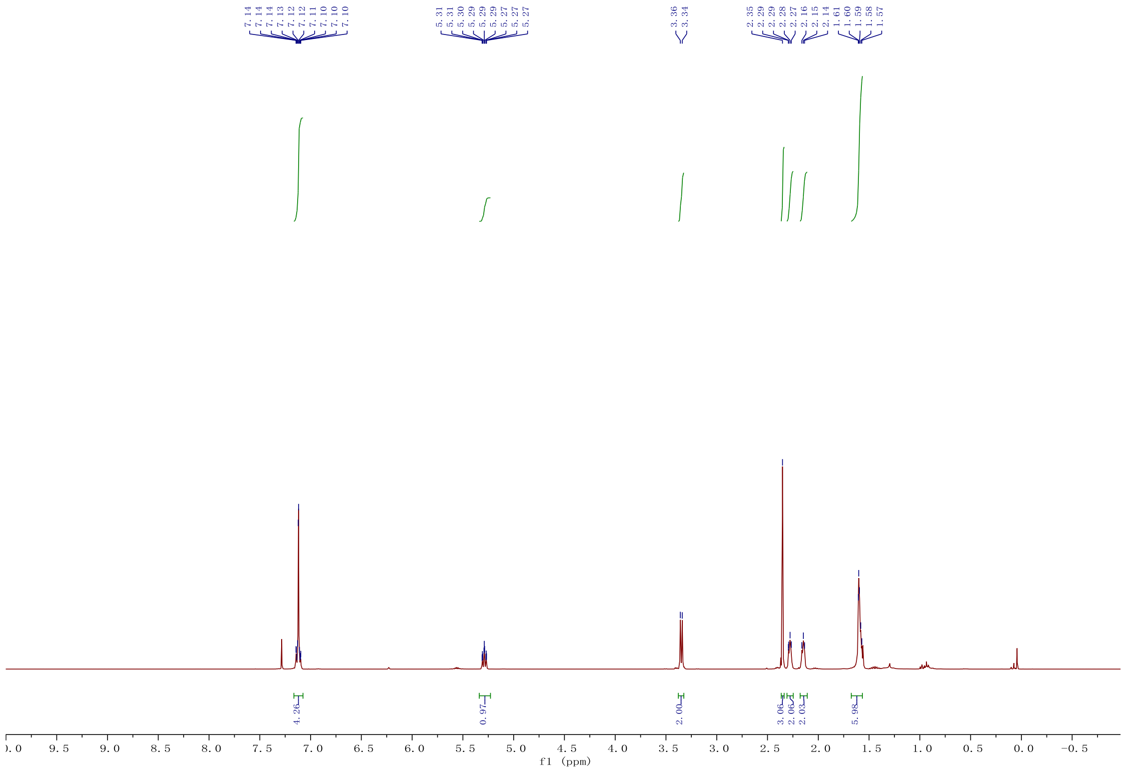

Supplementary Figure 65. ^{1}H NMR (400 MHz, Chloroform-*d*) of **sub. 3g**

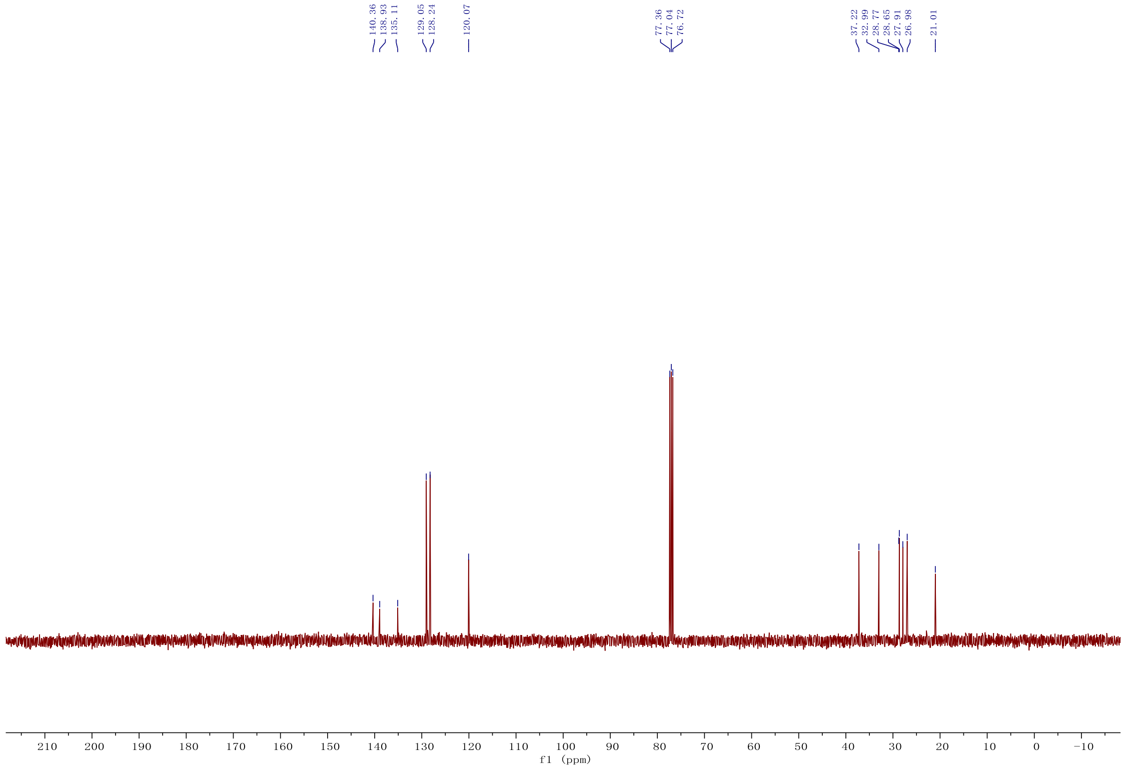

Supplementary Figure 66. ^{13}C NMR (101 MHz, Chloroform-*d*) of **sub. 3g**

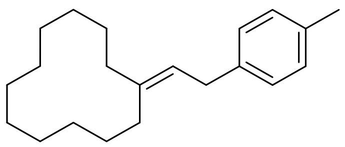

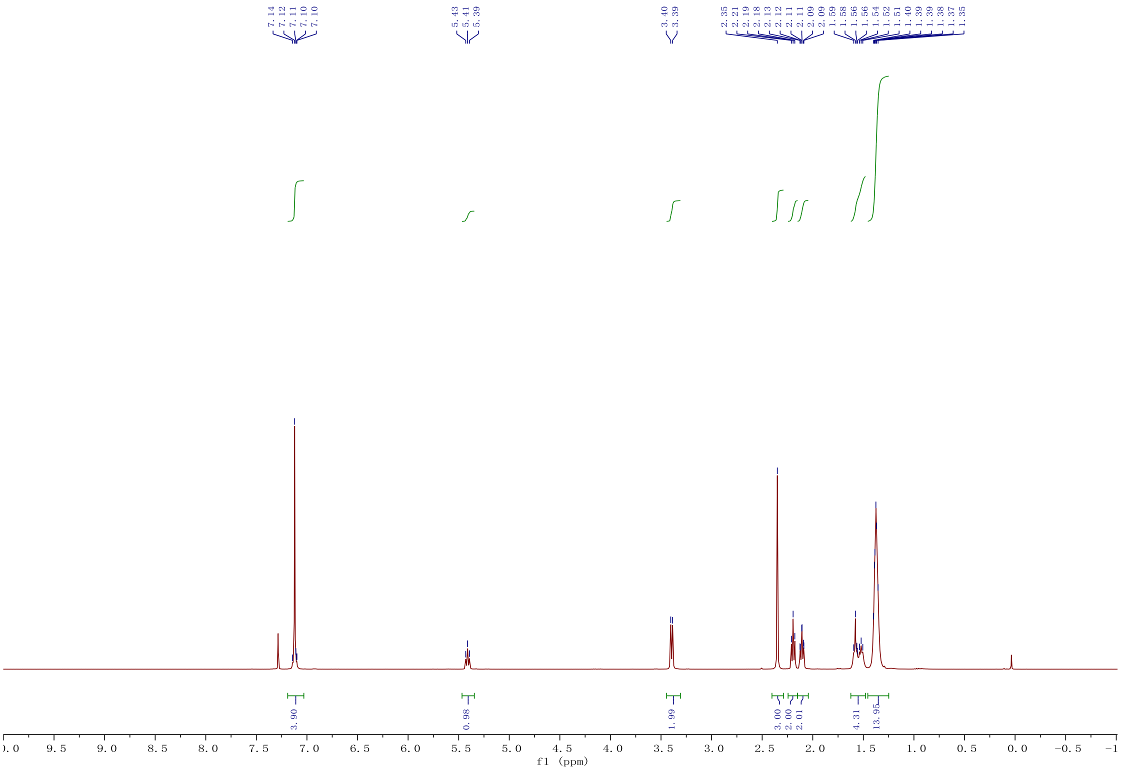

Supplementary Figure 67. ^{1}**H NMR** (400 MHz, Chloroform-*d*) of **sub. 3h**

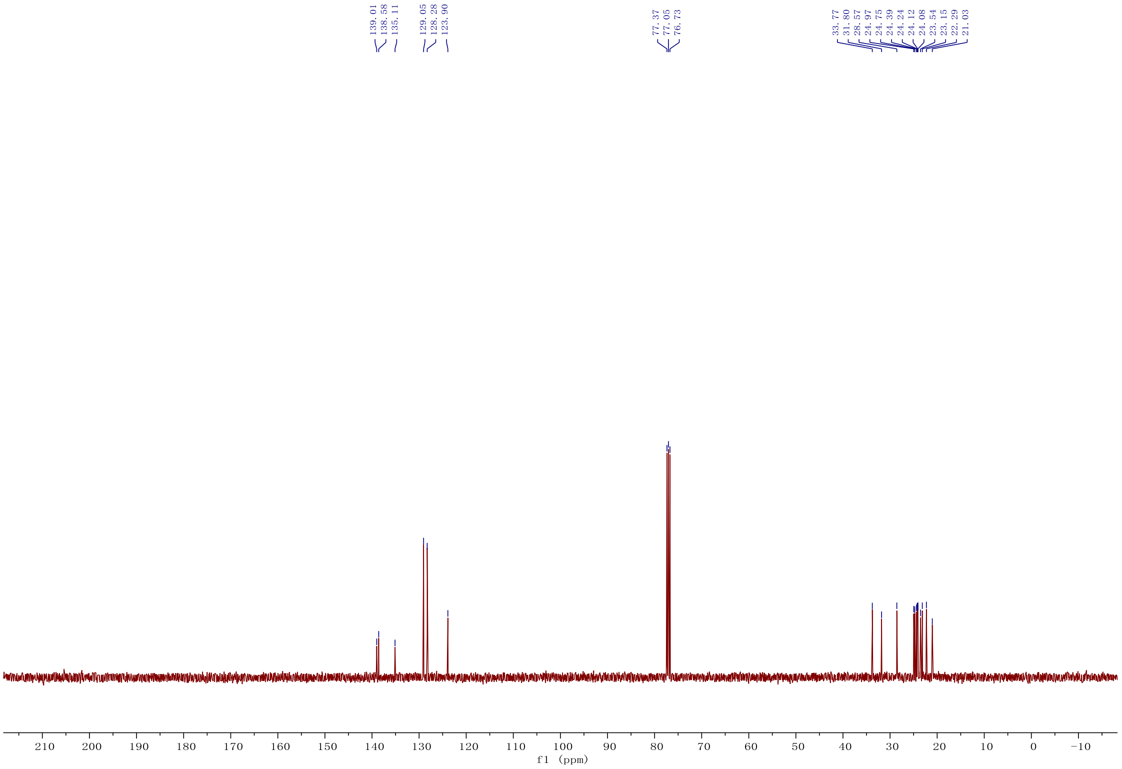

Supplementary Figure 68. ^{13}**C NMR** (101 MHz, Chloroform-*d*) of **sub. 3h**

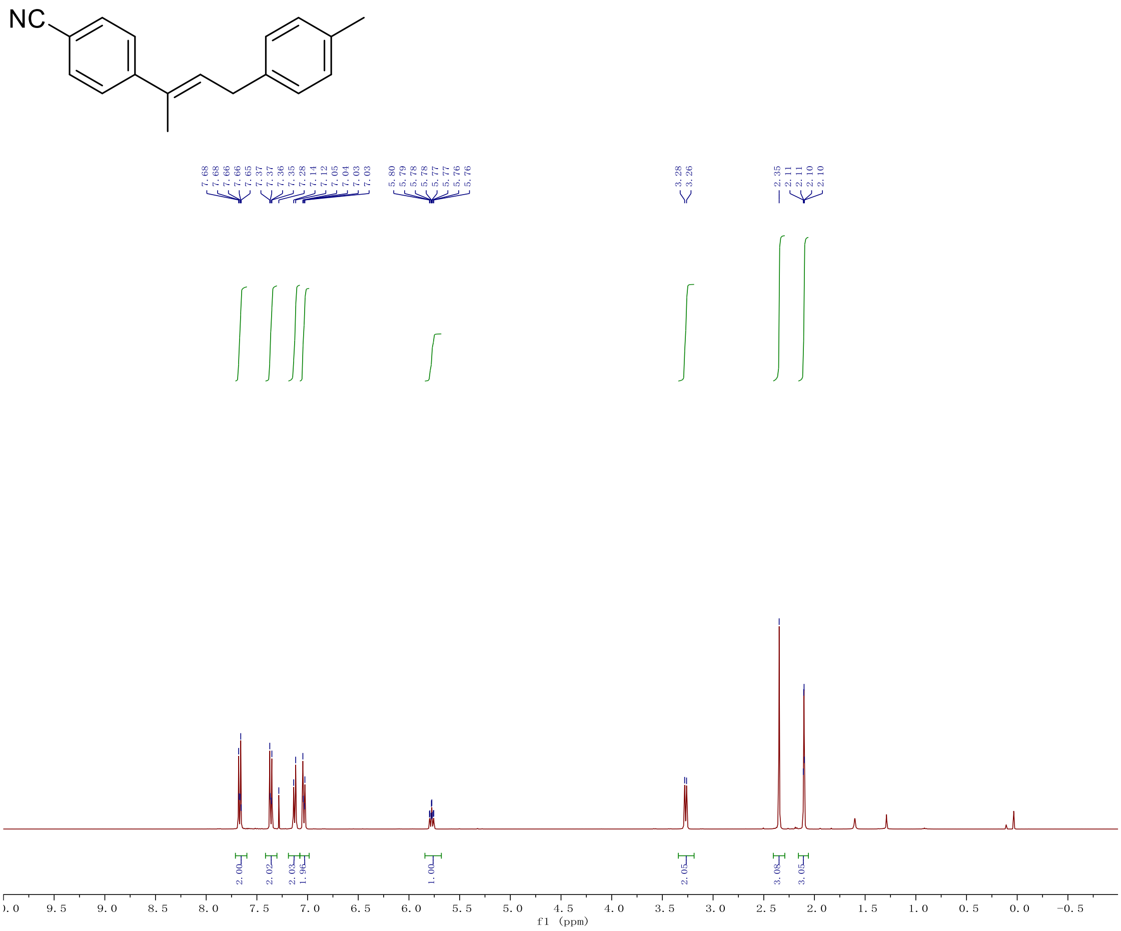

Supplementary Figure 69. ^{1}**H NMR** (400 MHz, Chloroform-*d*) of **sub. 3i**

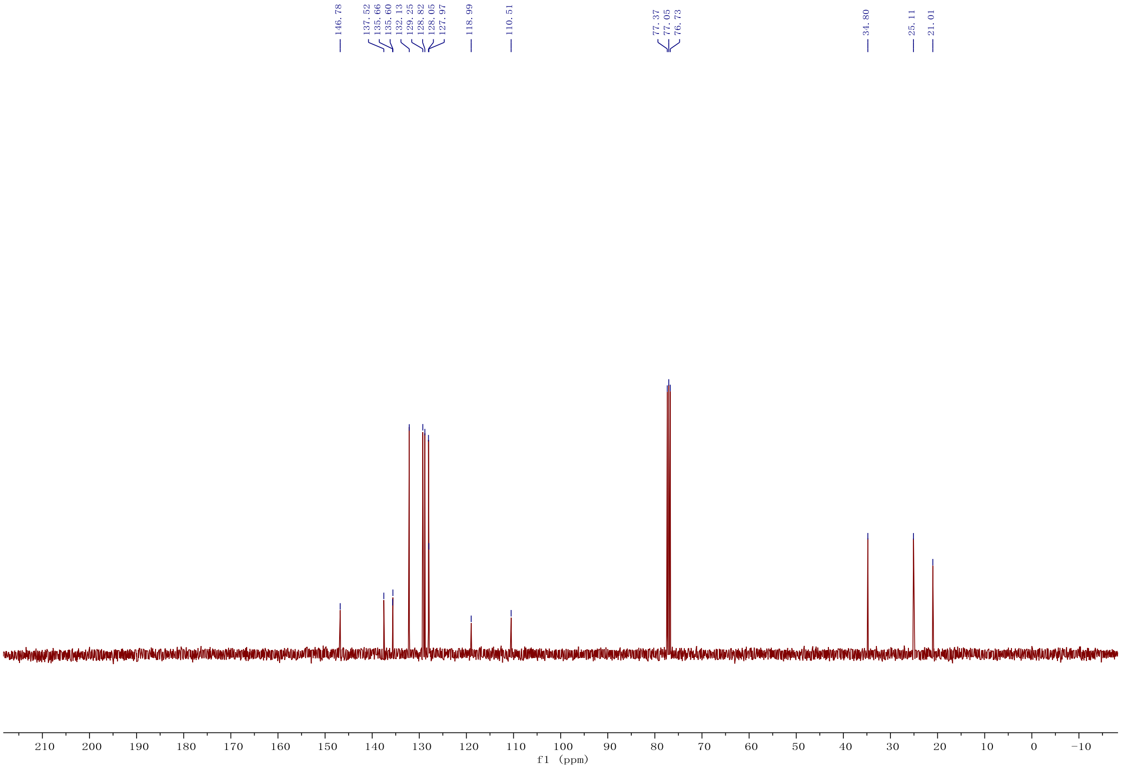

Supplementary Figure 70. ^{13}**C NMR** (101 MHz, Chloroform-*d*) of **sub. 3i**

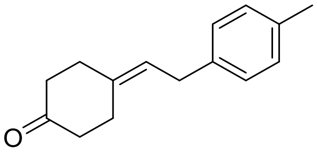

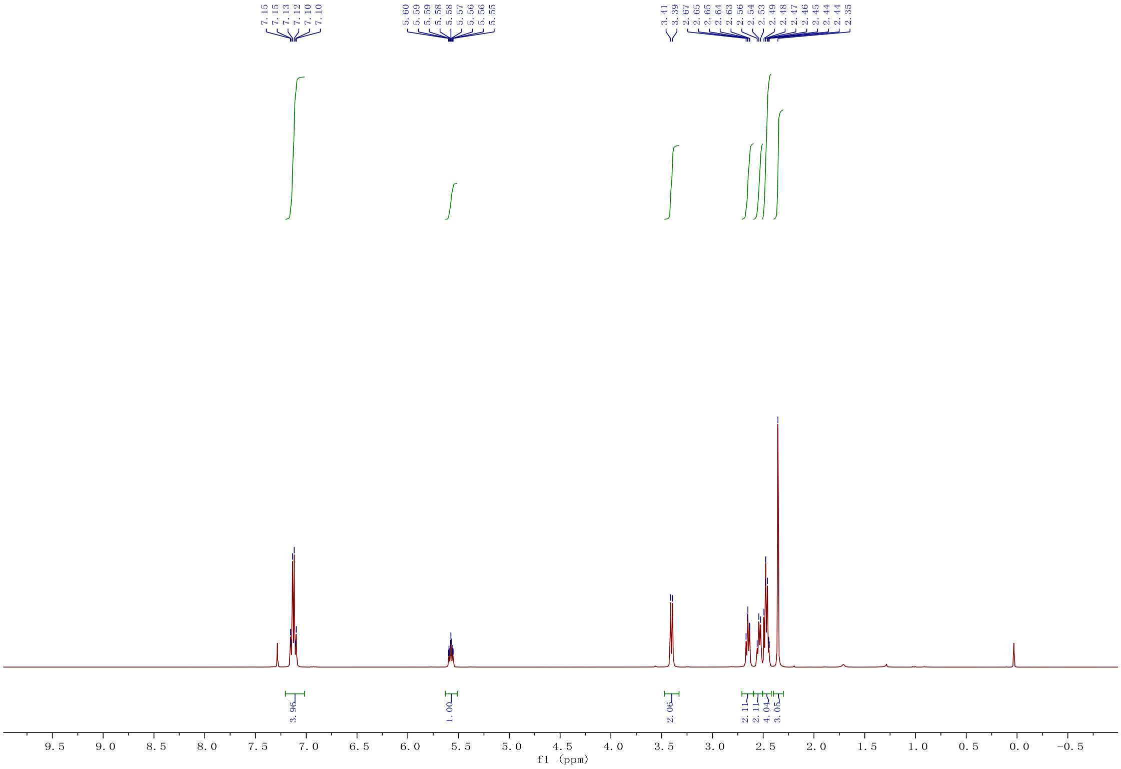

Supplementary Figure 71. ^{1}H NMR (400 MHz, Chloroform-*d*) of **sub. 3j**

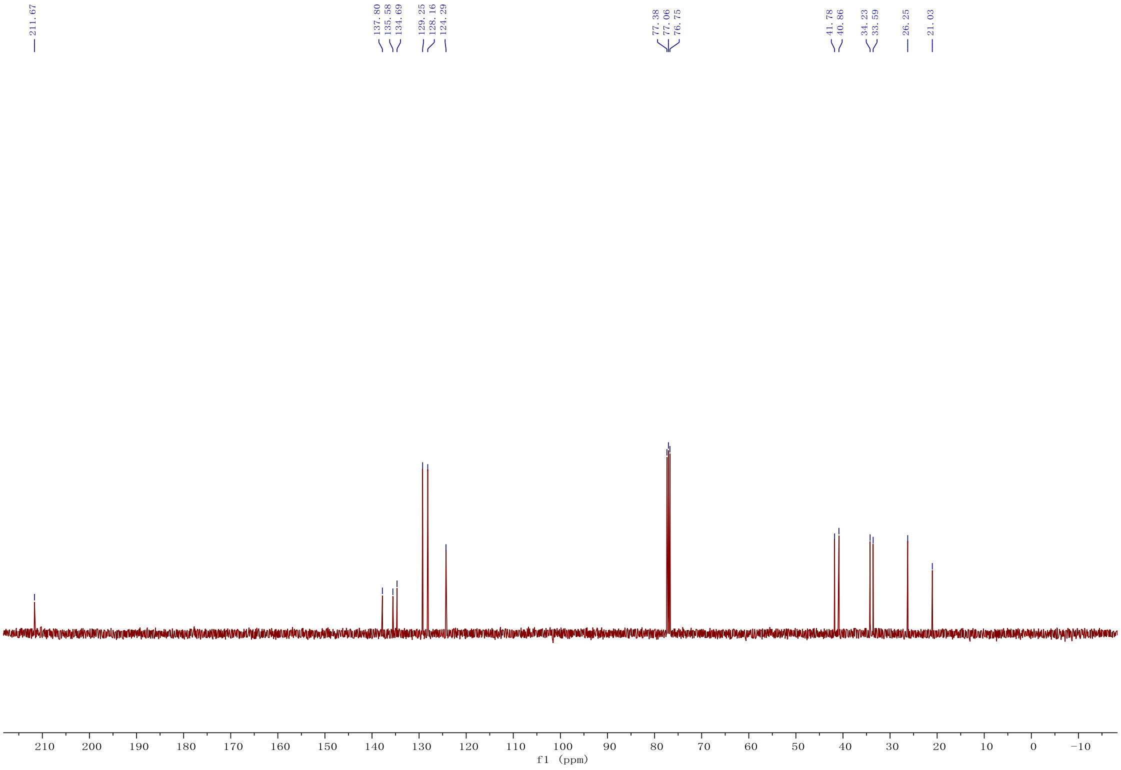

Supplementary Figure 72. ^{13}C NMR (101 MHz, Chloroform-*d*) of **sub. 3j**

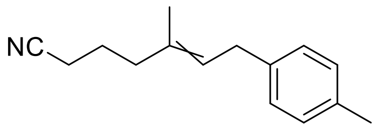

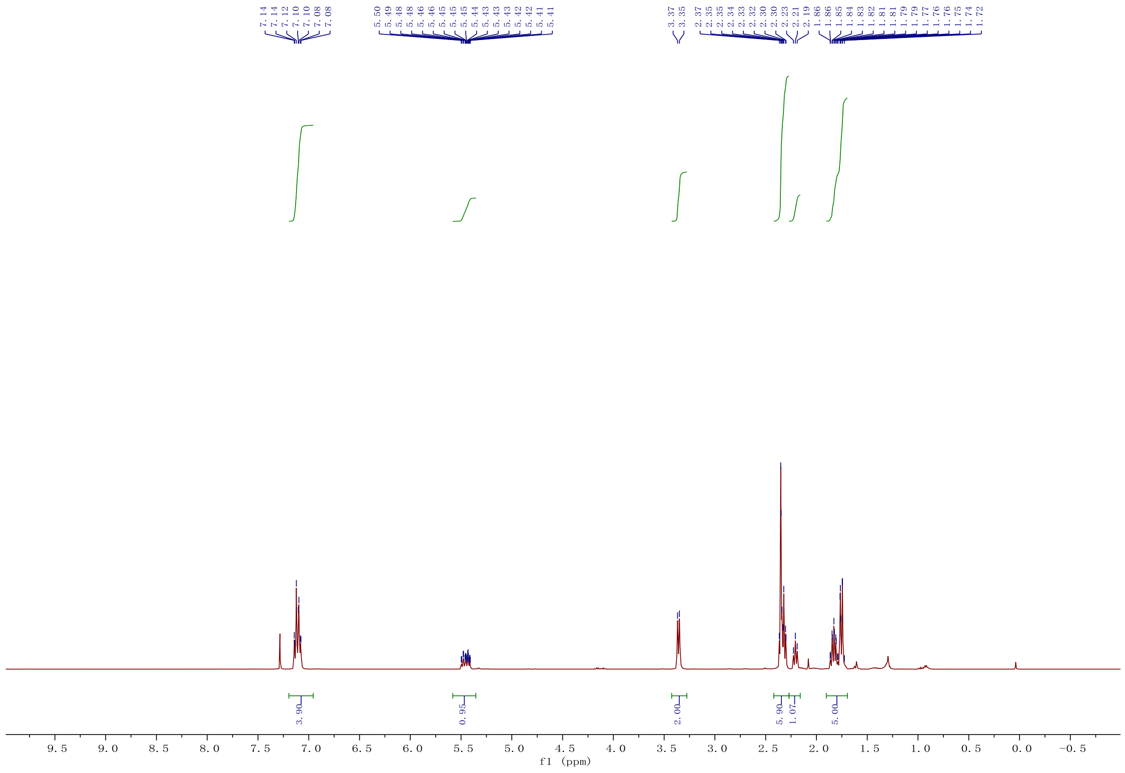

Supplementary Figure 73. ^{1}**H NMR** (400 MHz, Chloroform-*d*) of **sub. 3k**

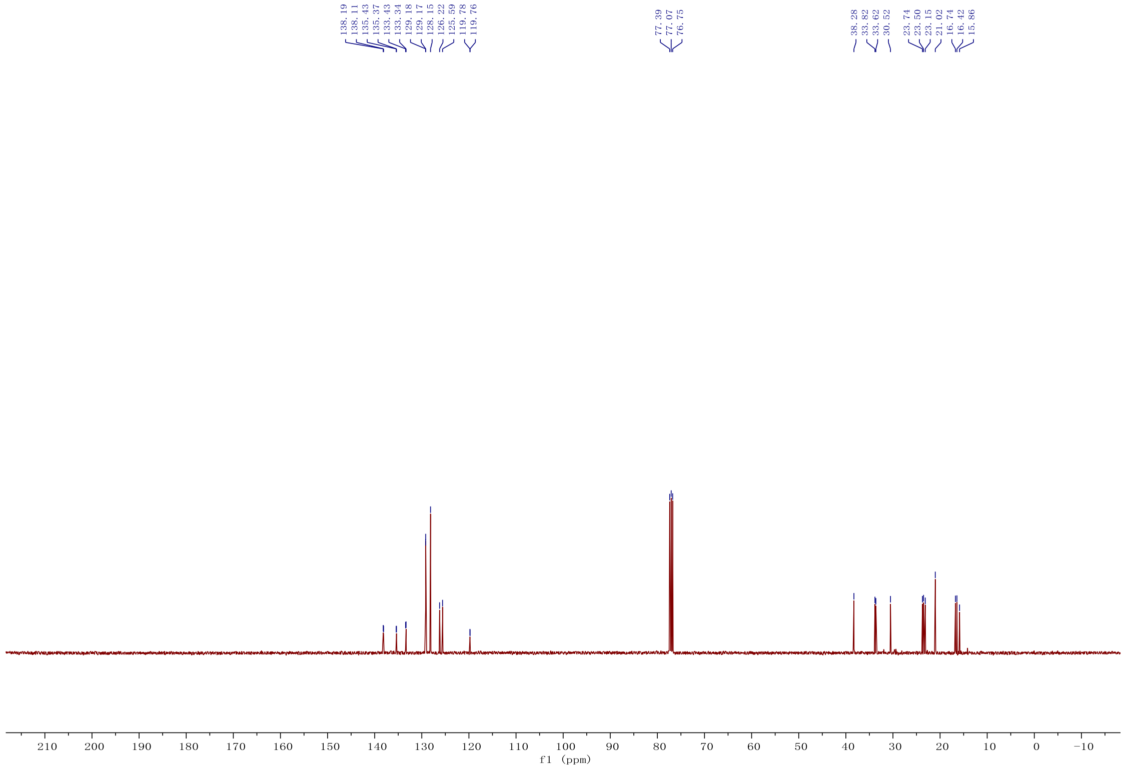

Supplementary Figure 74. ^{13}**C NMR** (101 MHz, Chloroform-*d*) of **sub. 3k**

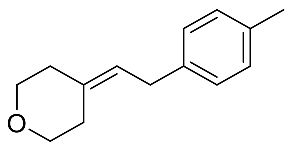

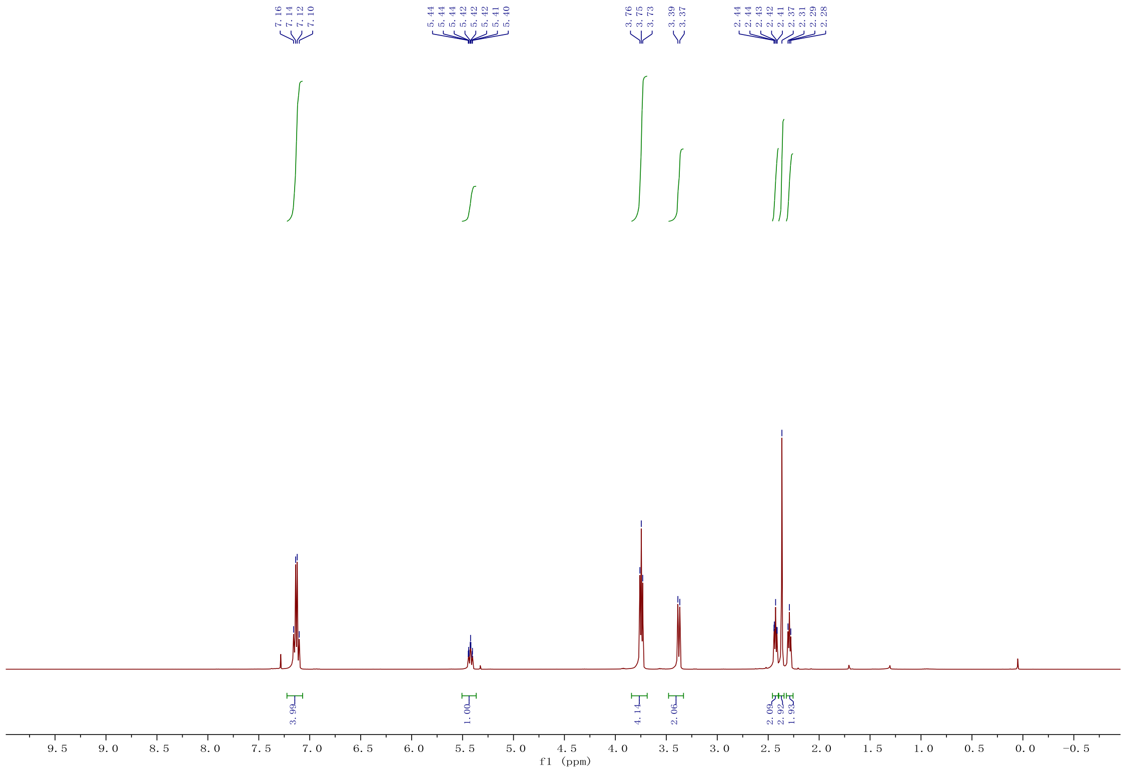

Supplementary Figure 75. ^{1}**H NMR** (400 MHz, Chloroform-*d*) of **sub. 3l**

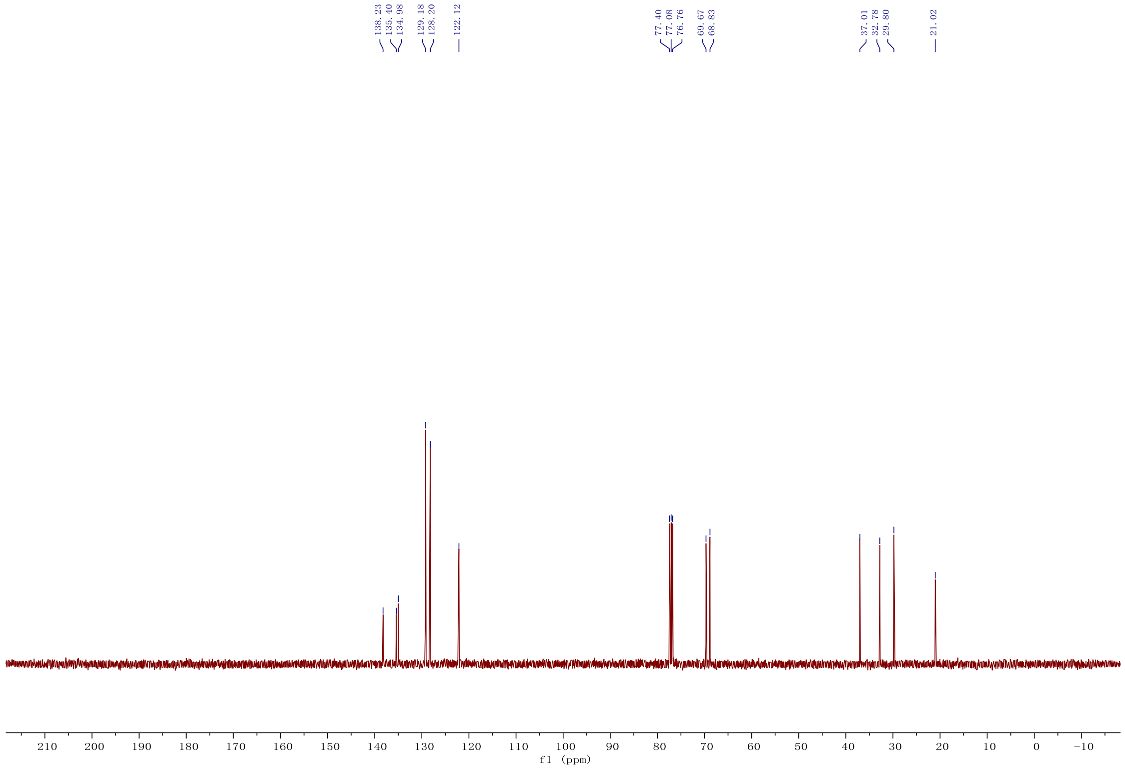

Supplementary Figure 76. ^{13}**C NMR** (101 MHz, Chloroform-*d*) of **sub. 3l**

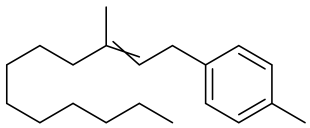

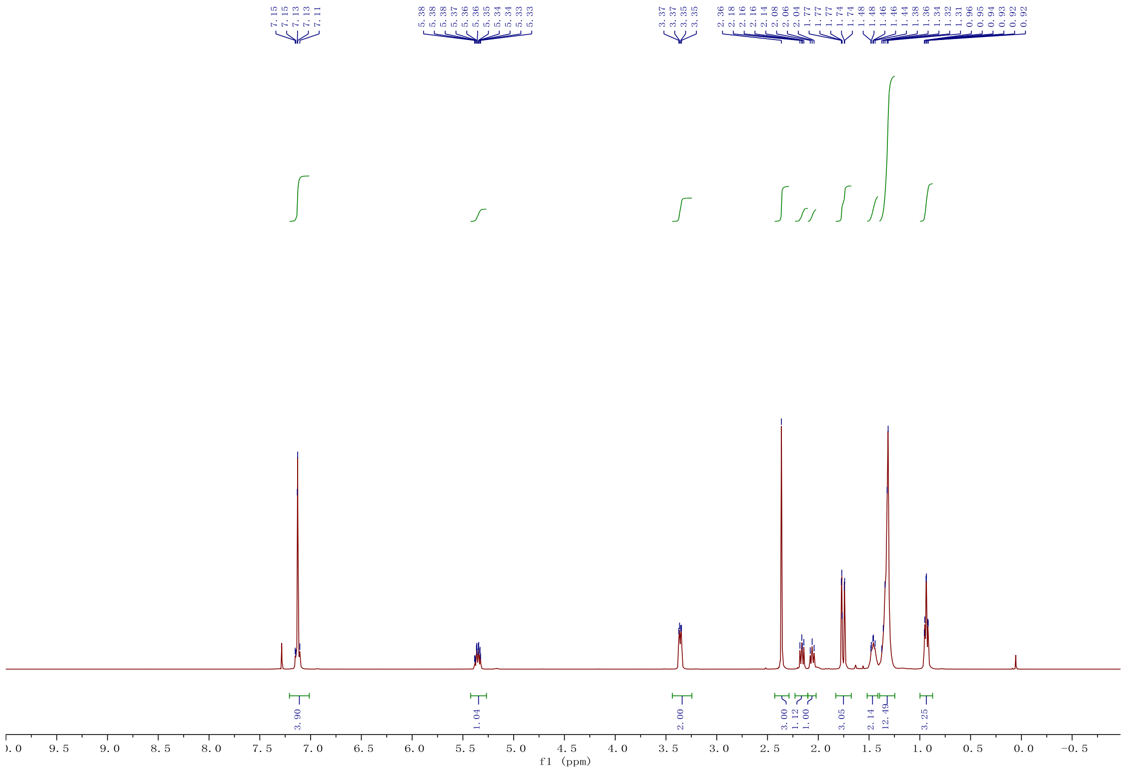

Supplementary Figure 77. ^{1}**H NMR** (400 MHz, Chloroform-*d*) of **sub. 3m**

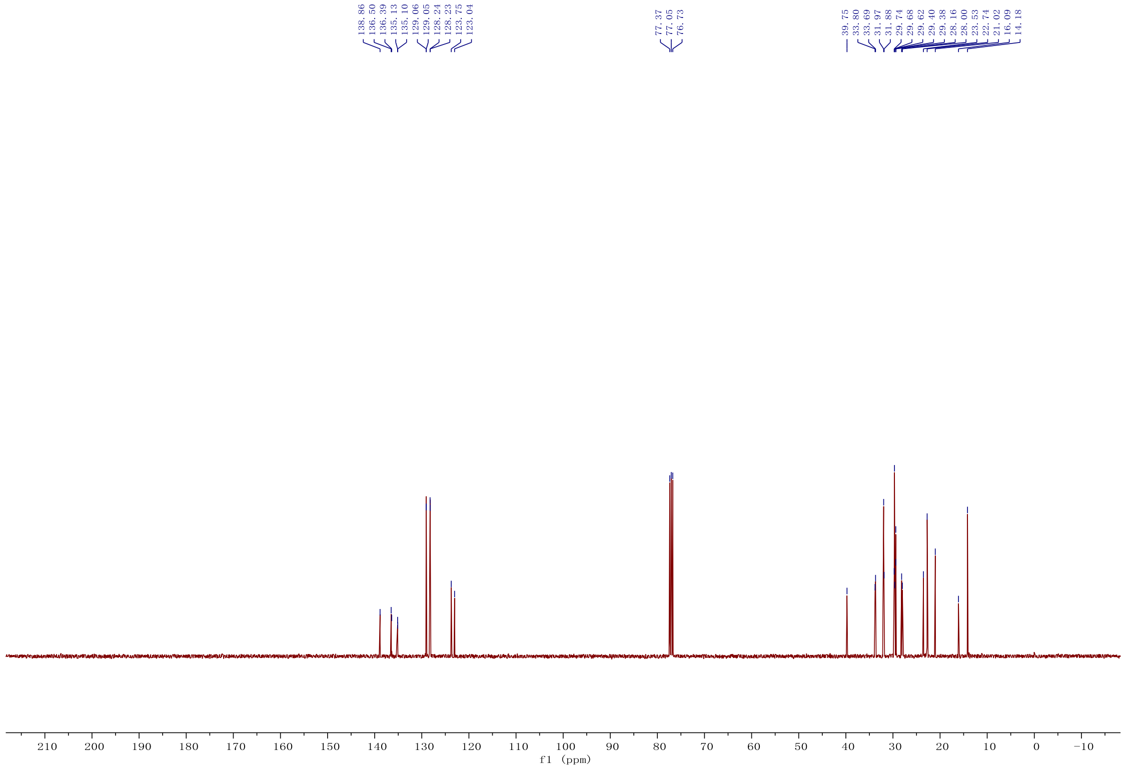

Supplementary Figure 78. ^{13}**C NMR** (101 MHz, Chloroform-*d*) of **sub. 3m**

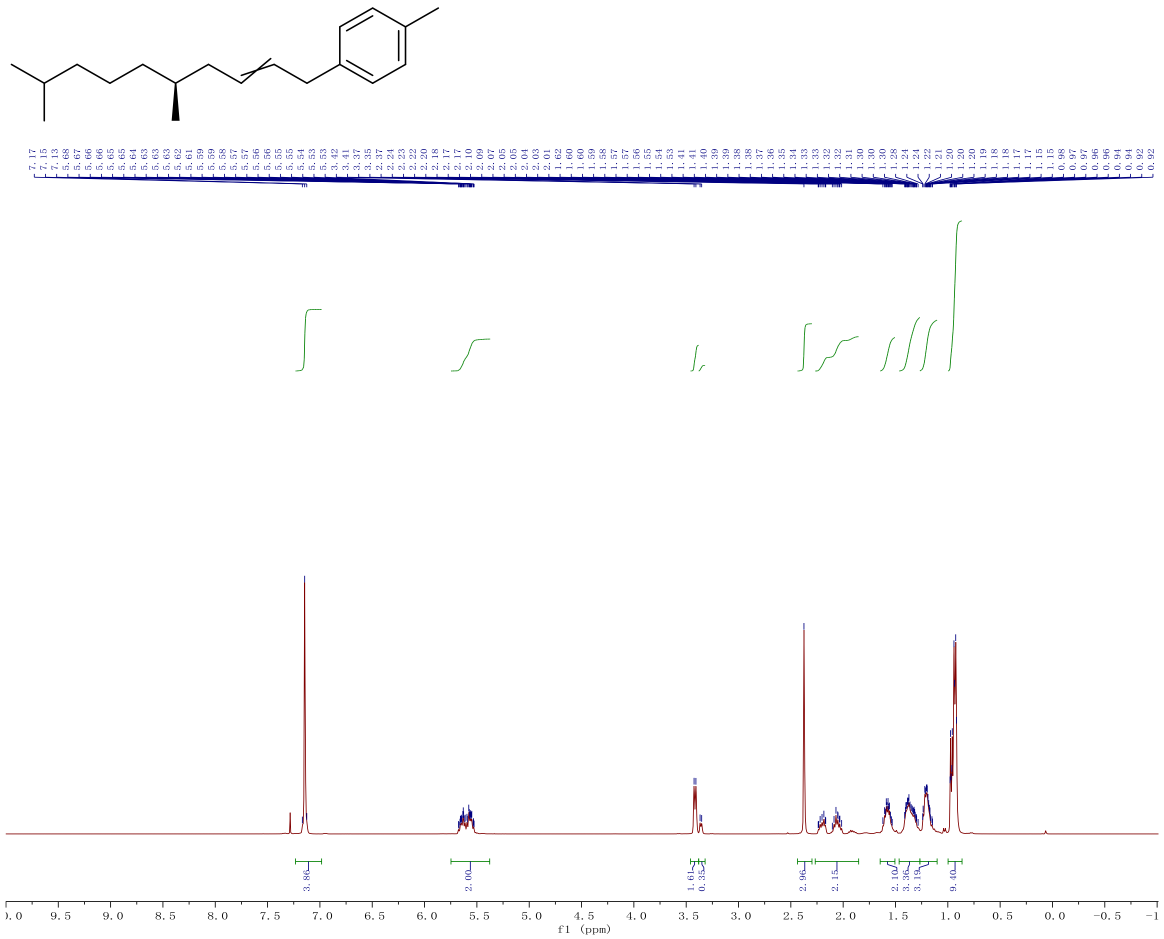

Supplementary Figure 79. **^{1}H NMR** (400 MHz, Chloroform-*d*) of **sub. 3n**

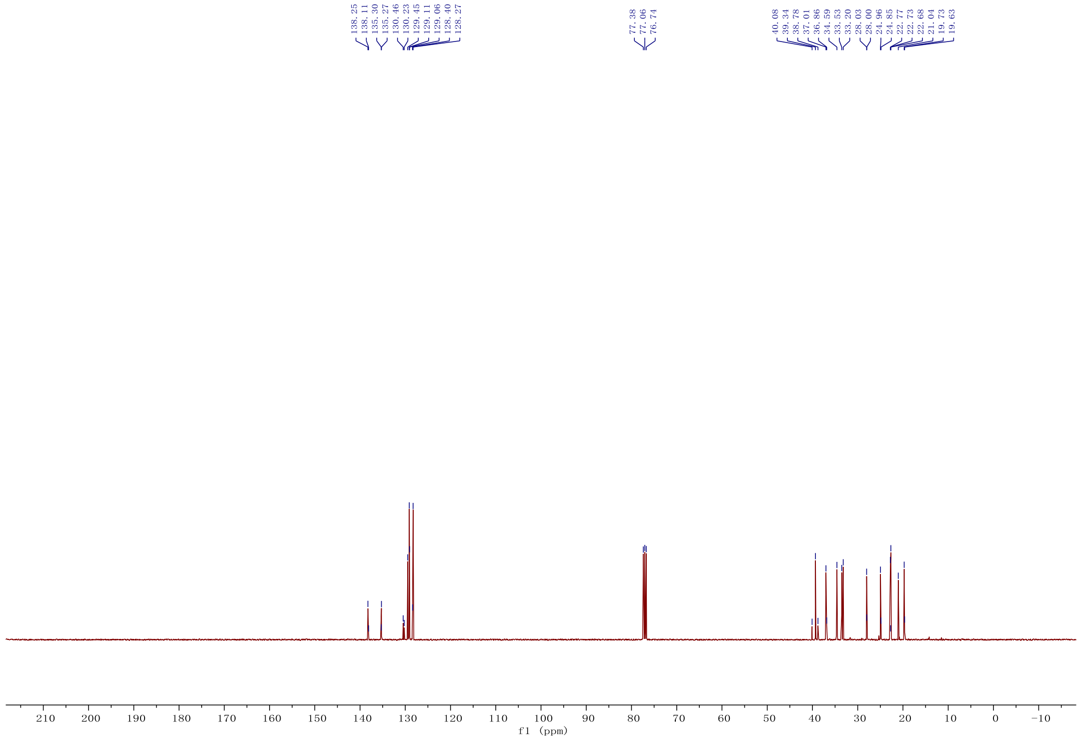

Supplementary Figure 80. **^{13}C NMR** (101 MHz, Chloroform-*d*) of **sub. 3n**

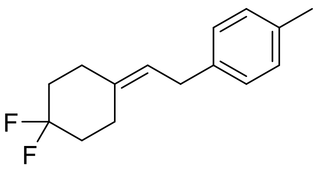

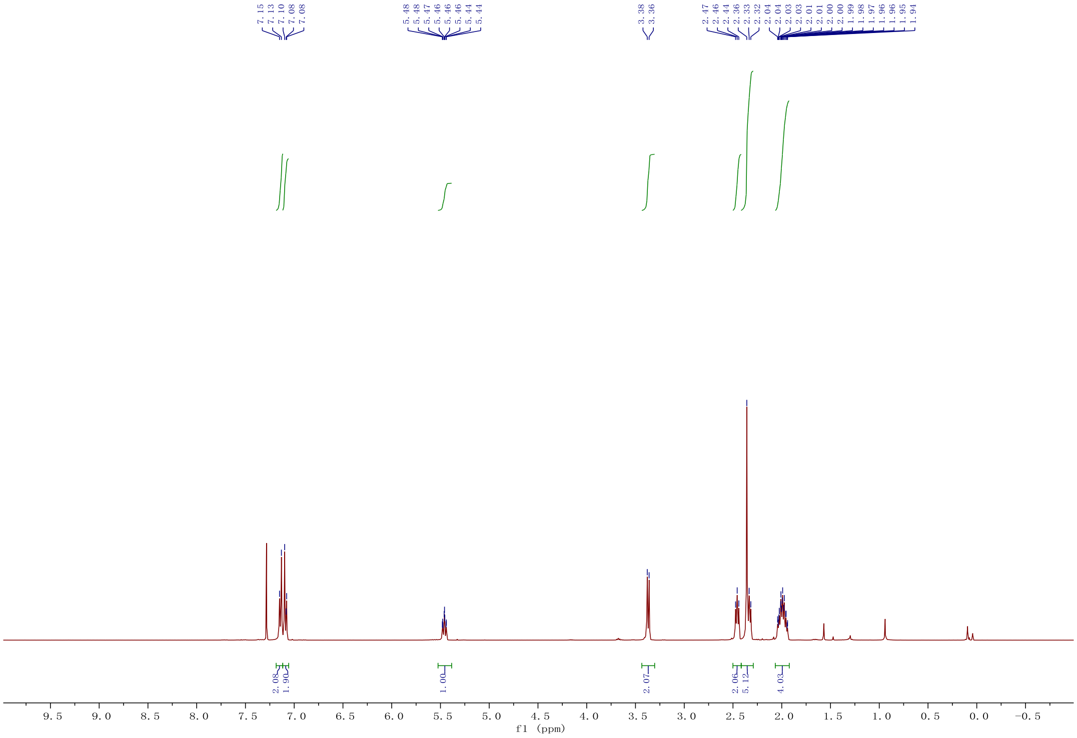

Supplementary Figure 81. ^{1}**H NMR** (400 MHz, Chloroform-*d*) of **sub. 3o**

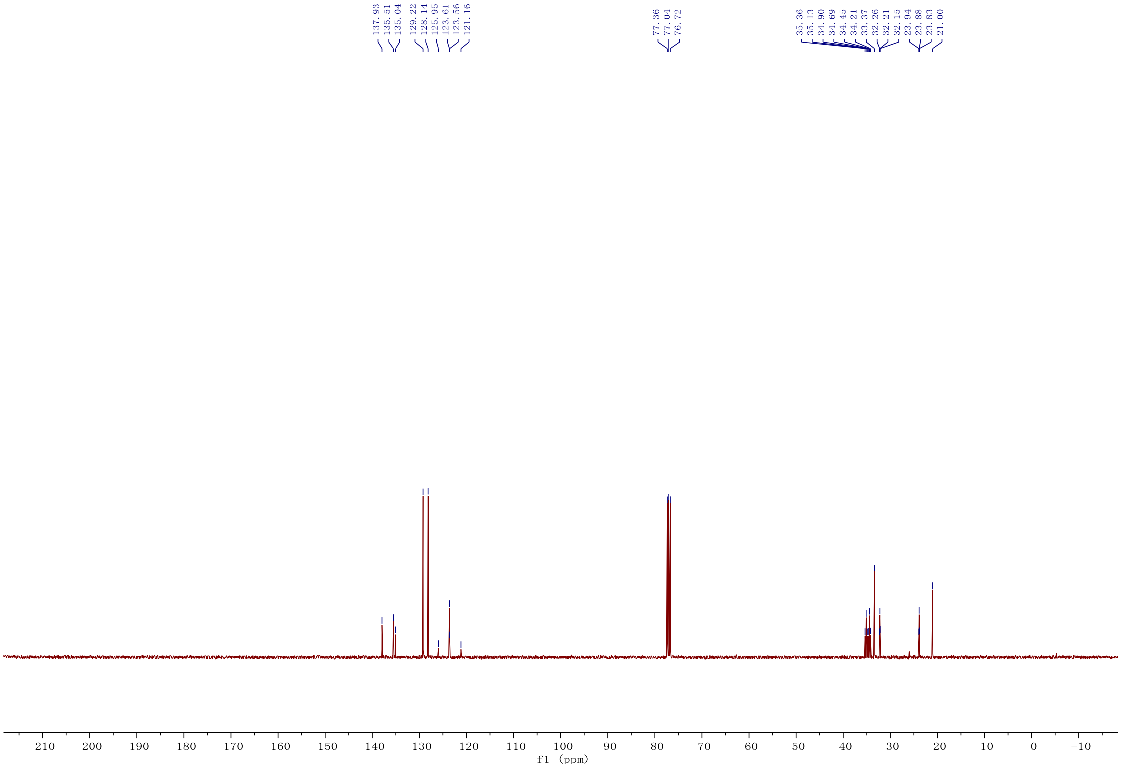

Supplementary Figure 82. ^{13}**C NMR** (101 MHz, Chloroform-*d*) of **sub. 3o**

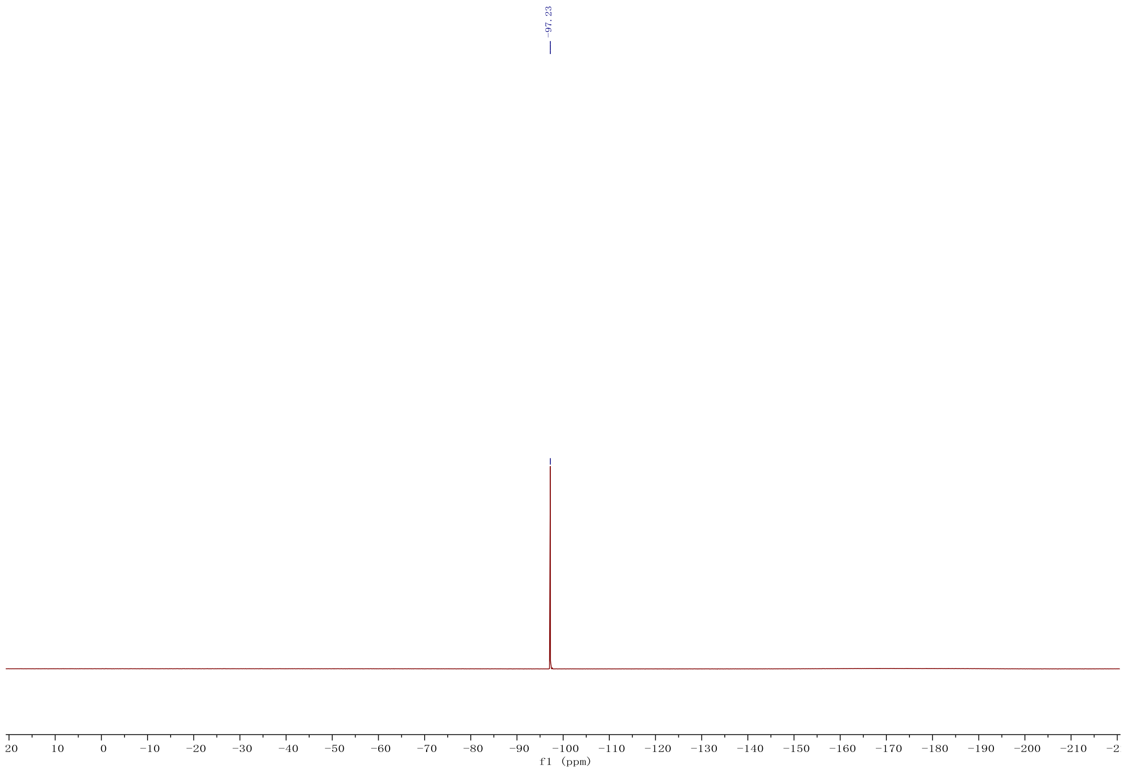

Supplementary Figure 83. ^{19}**F NMR** (376 MHz, Chloroform-*d*) of **sub. 3o**

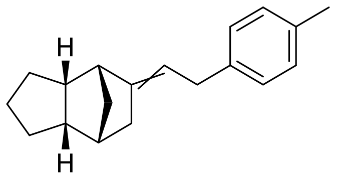

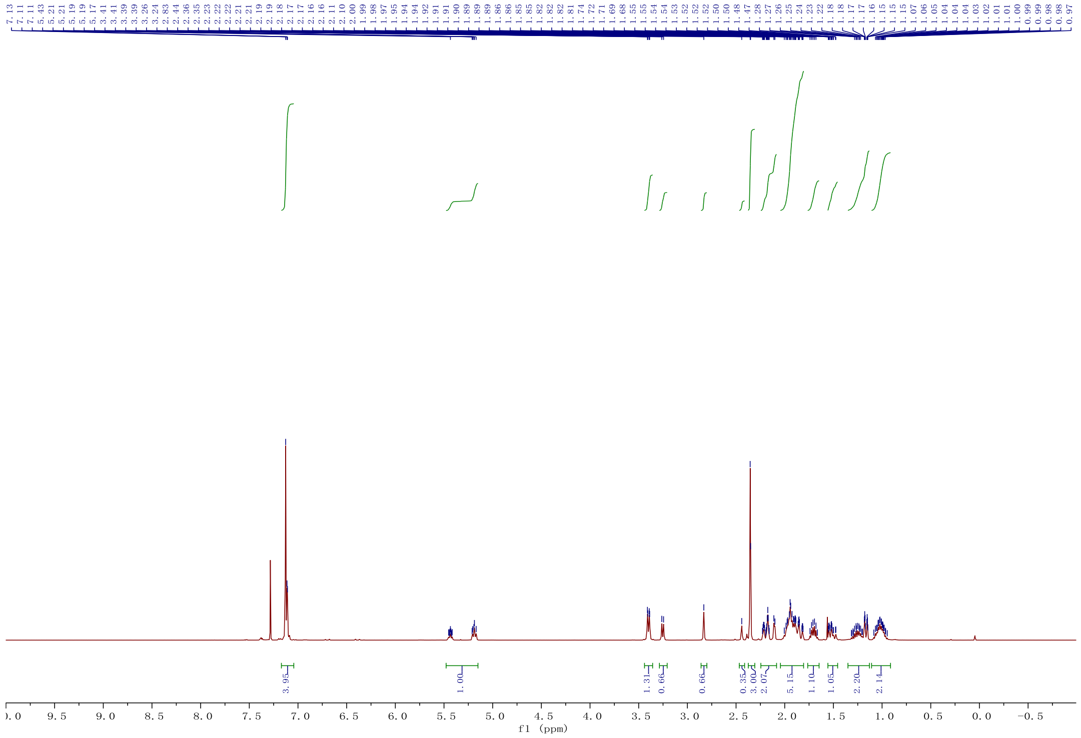

Supplementary Figure 84. ^{1}**H NMR** (400 MHz, Chloroform-*d*) of **sub. 3p**

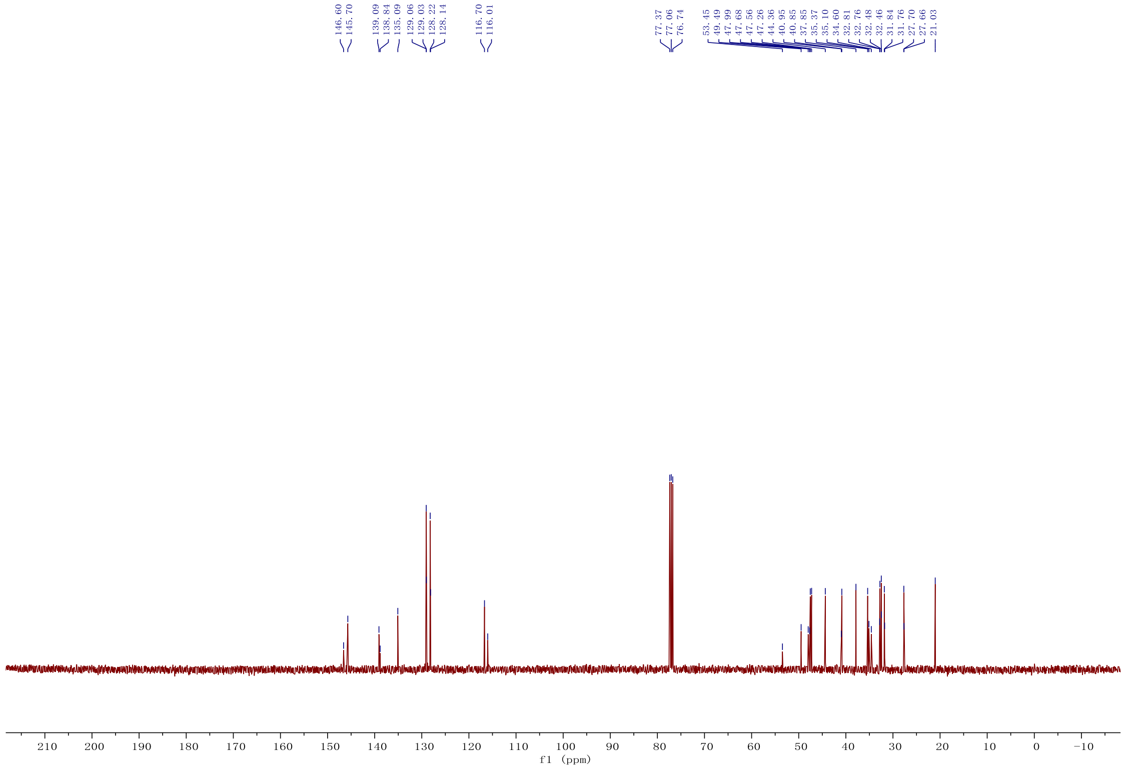

Supplementary Figure 85. ^{13}**C NMR** (101 MHz, Chloroform-*d*) of **sub. 3p**

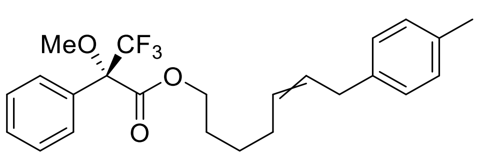

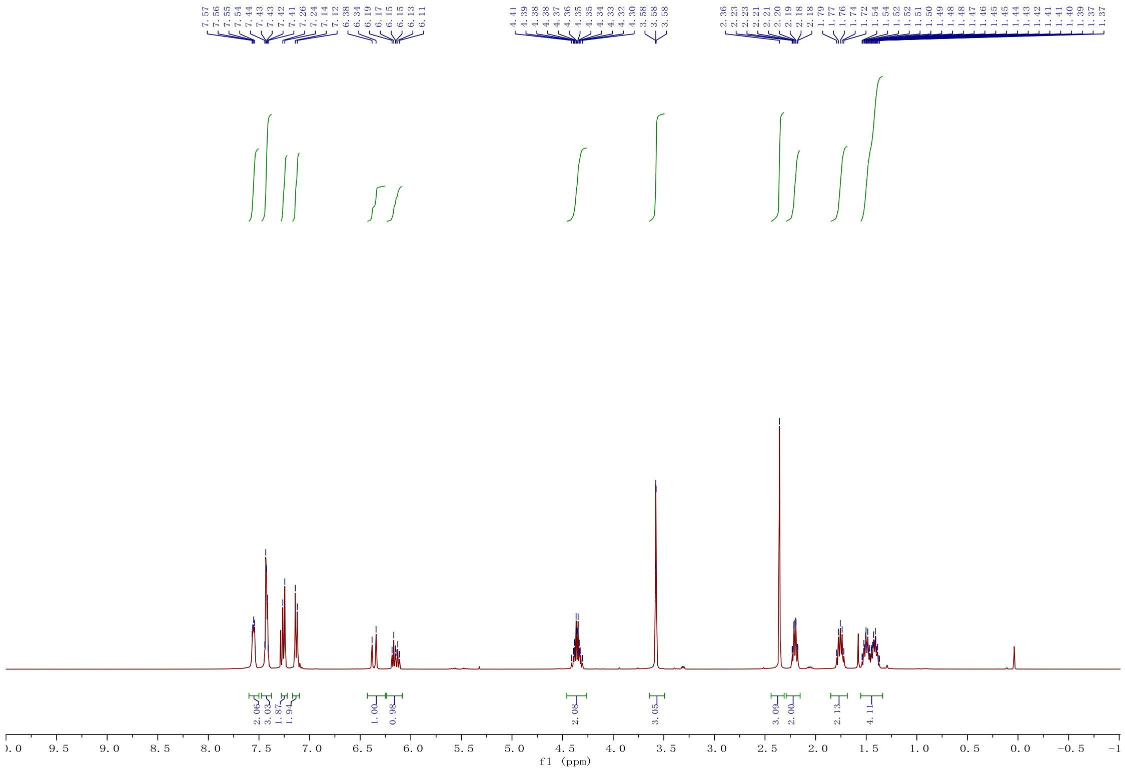

Supplementary Figure 86. **^{1}H NMR** (400 MHz, Chloroform-*d*) of **sub. 3q**

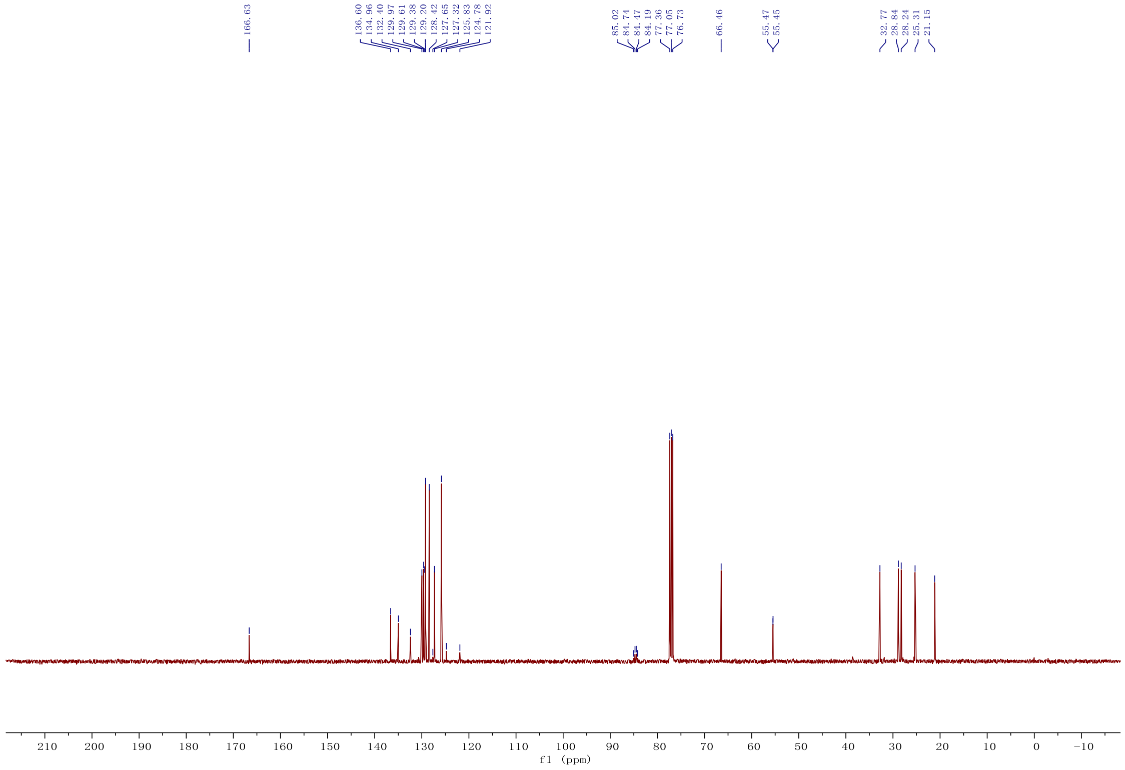

Supplementary Figure 87. **^{13}C NMR** (101 MHz, Chloroform-*d*) of **sub. 3q**

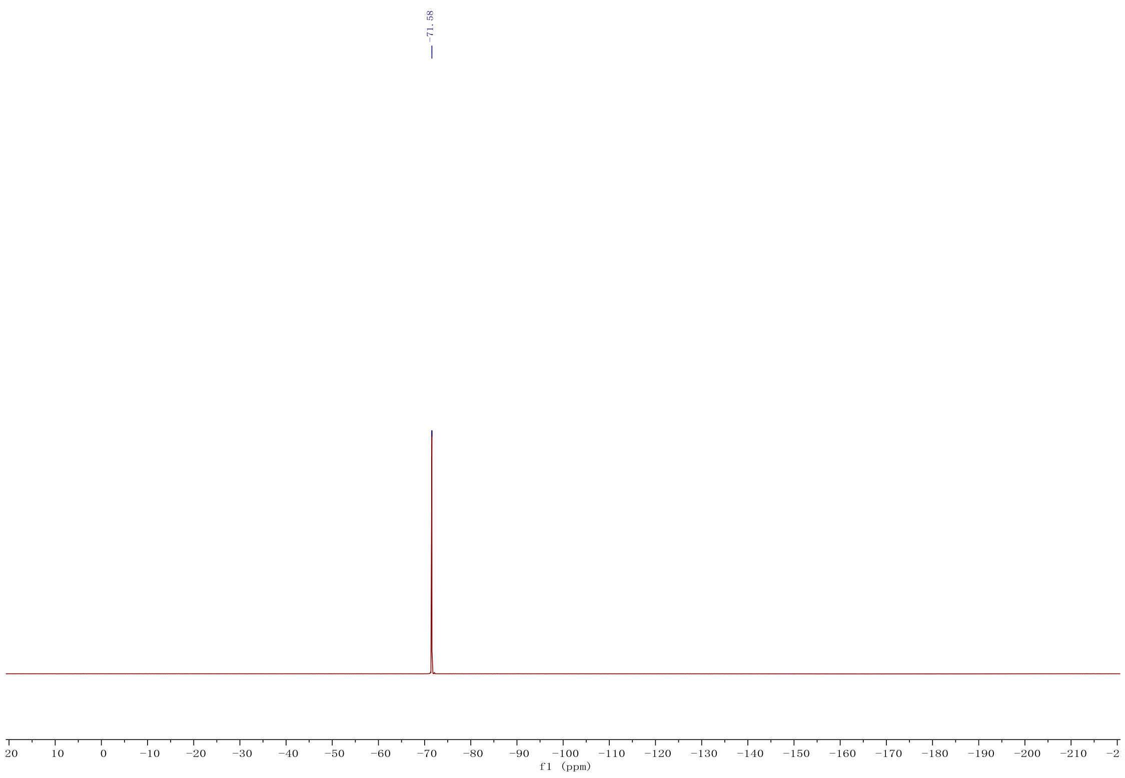

Supplementary Figure 88. ^{19}**F NMR** (376 MHz, Chloroform-*d*) of **sub. 3q**

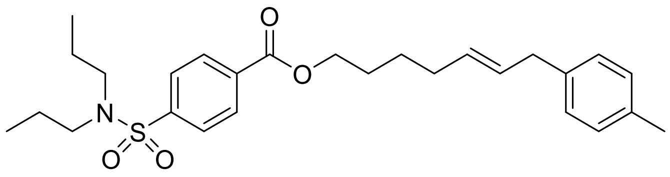

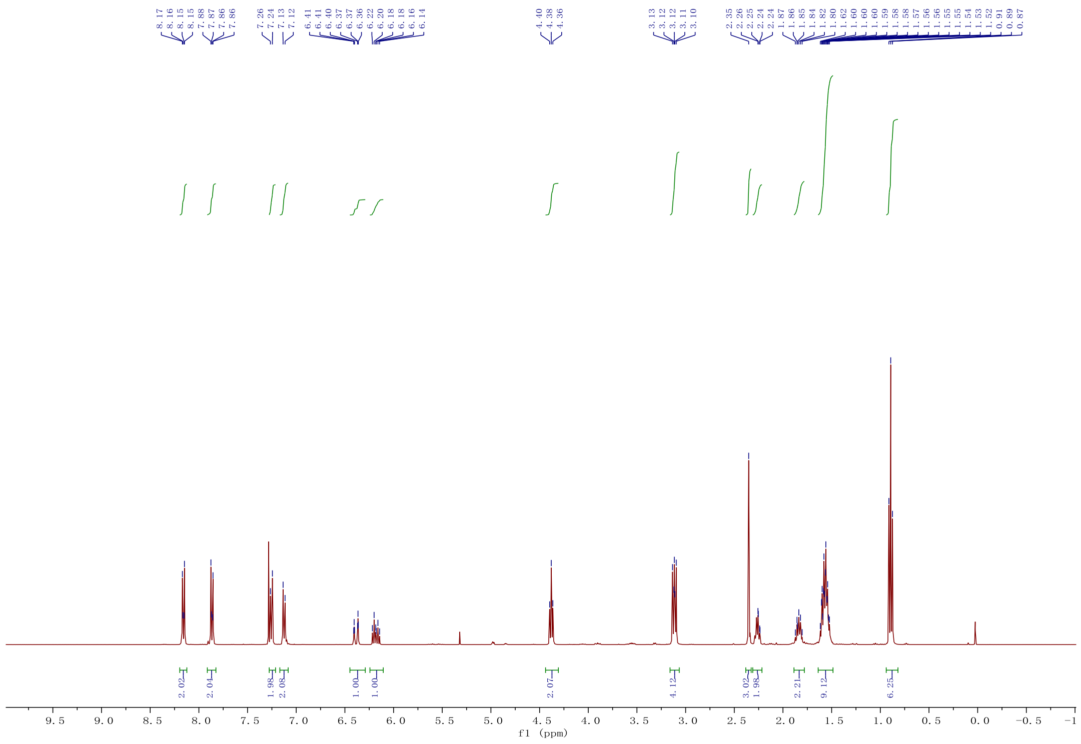

Supplementary Figure 89. ^{1}**H NMR** (400 MHz, Chloroform-*d*) of **sub. 3r**

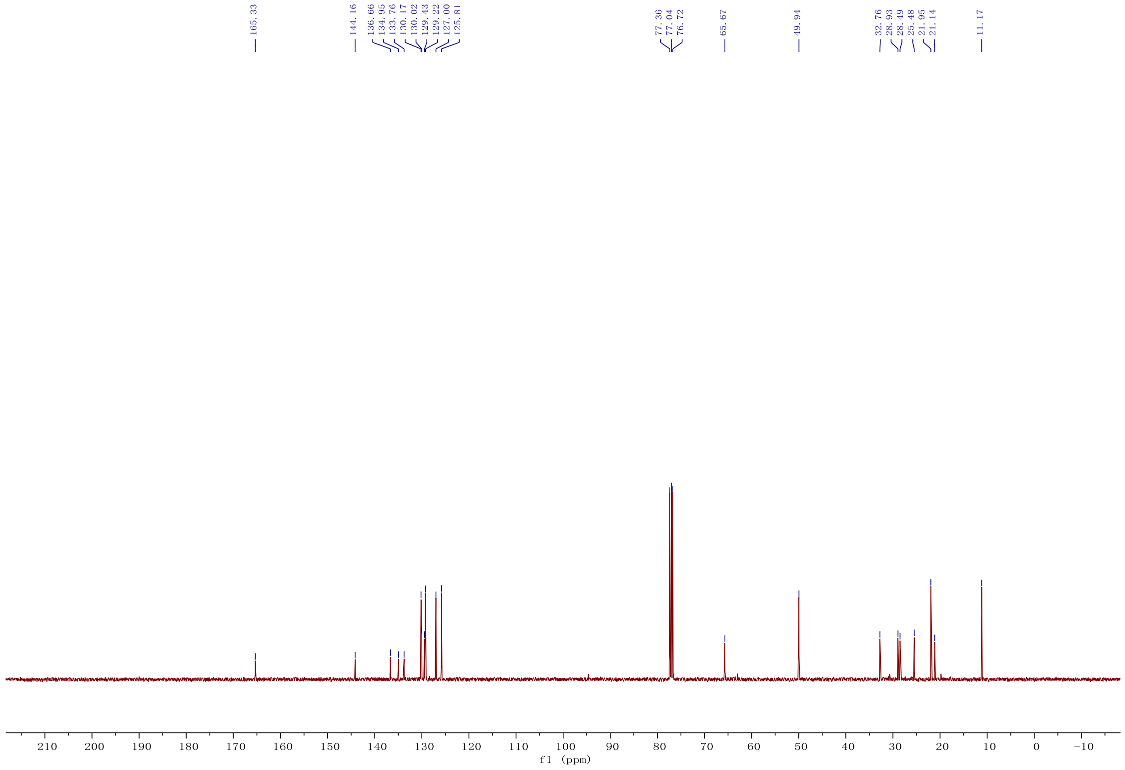

Supplementary Figure 90. ^{13}**C NMR** (101 MHz, Chloroform-*d*) of **sub. 3r**

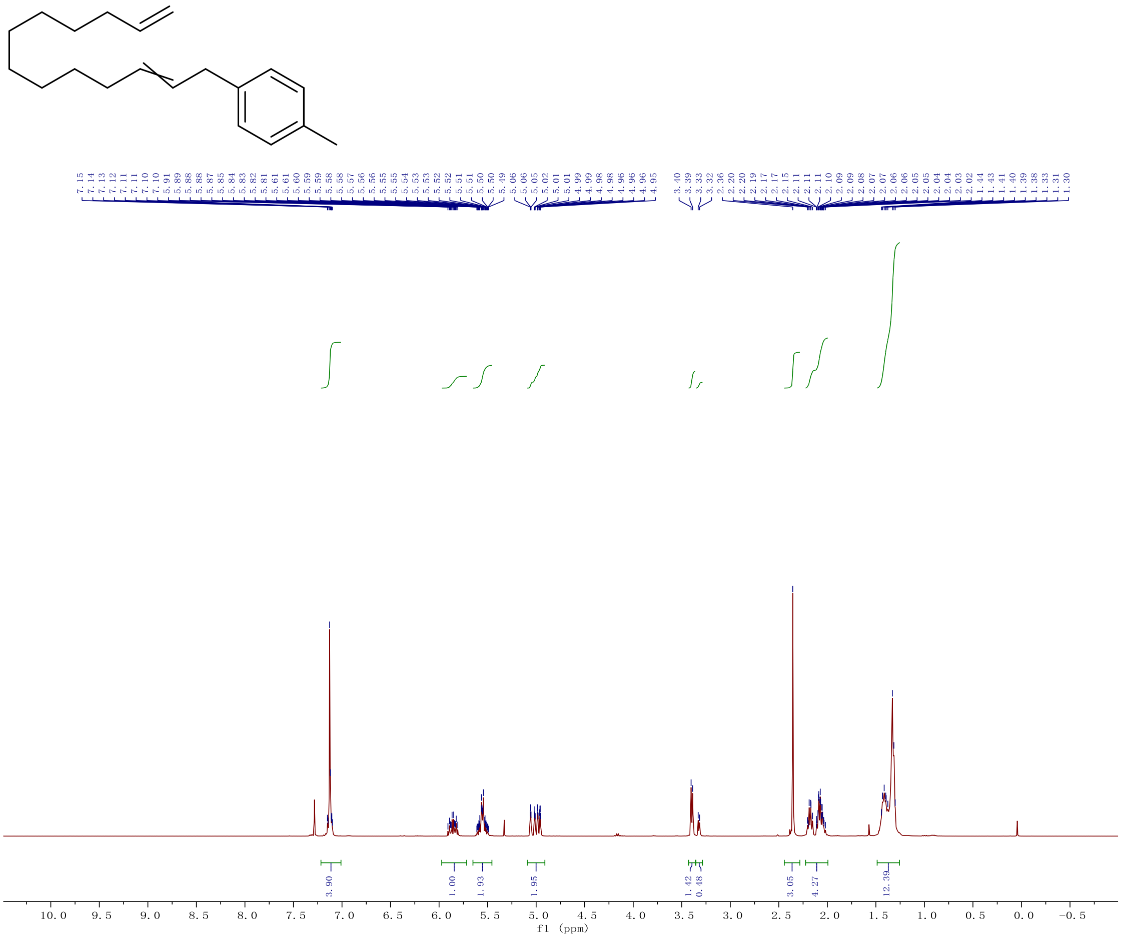

Supplementary Figure 91. ^{1}H NMR (400 MHz, Chloroform-*d*) of **sub. 3s**

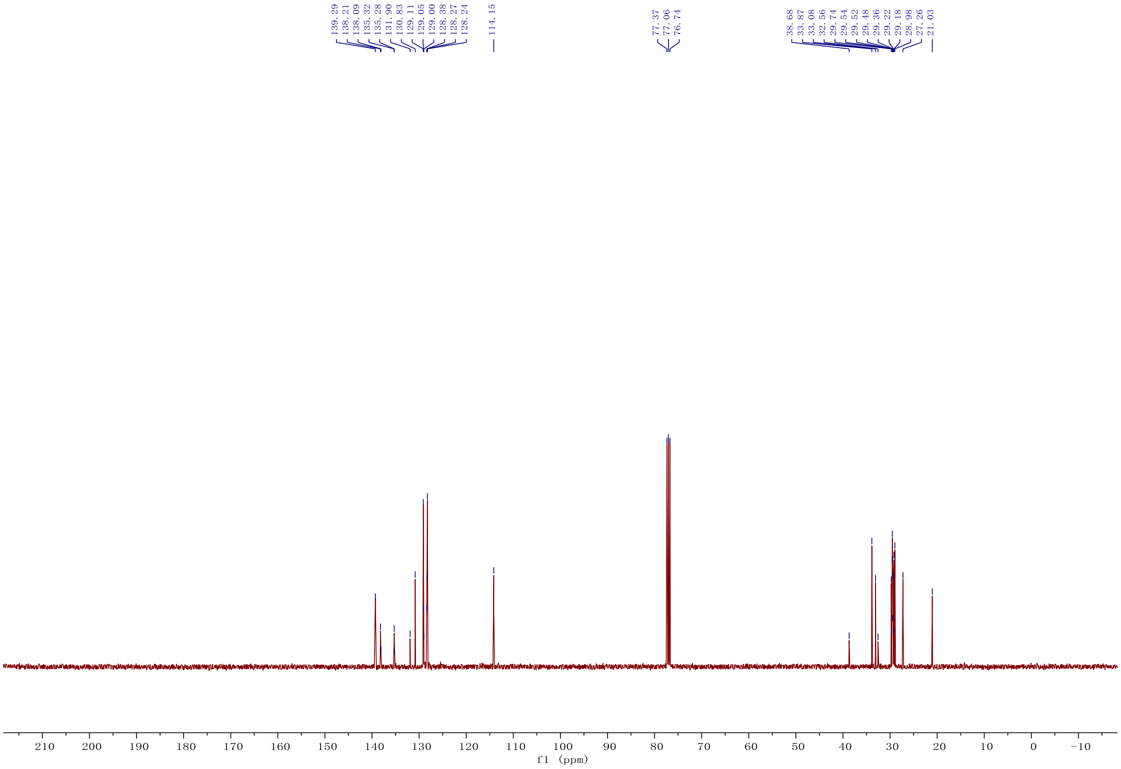

Supplementary Figure 92. ^{13}C NMR (101 MHz, Chloroform-*d*) of **sub. 3s**

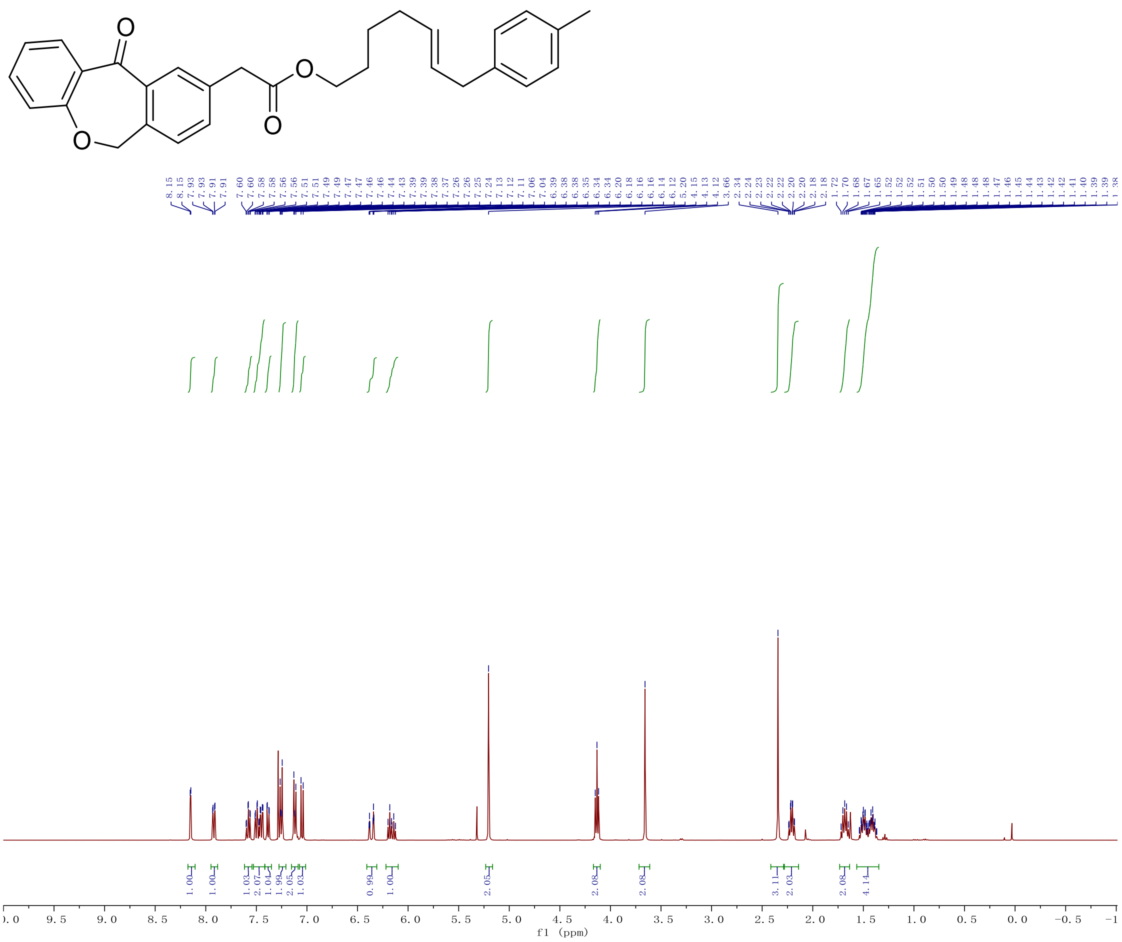

Supplementary Figure 93. **^{1}H NMR** (400 MHz, Chloroform-*d*) of **sub. 3t**

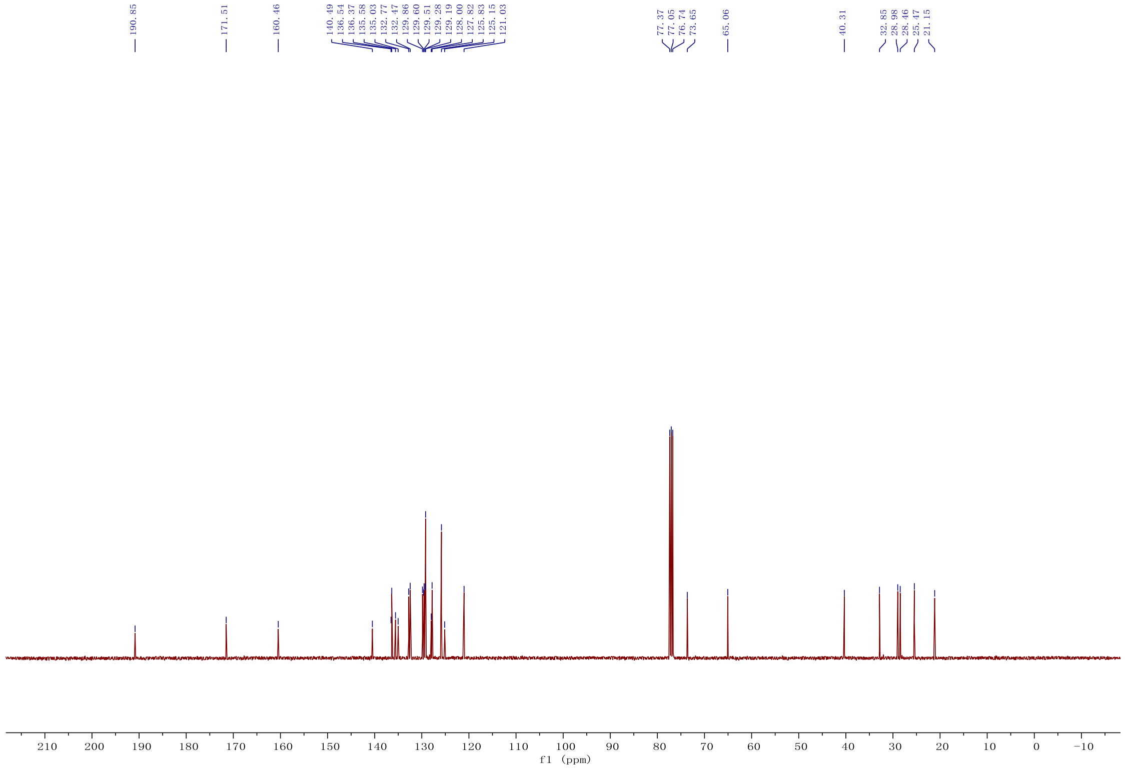

Supplementary Figure 94. **^{13}C NMR** (101 MHz, Chloroform-*d*) of **sub. 3t**

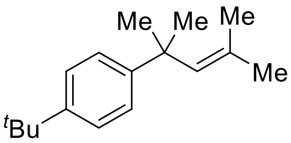

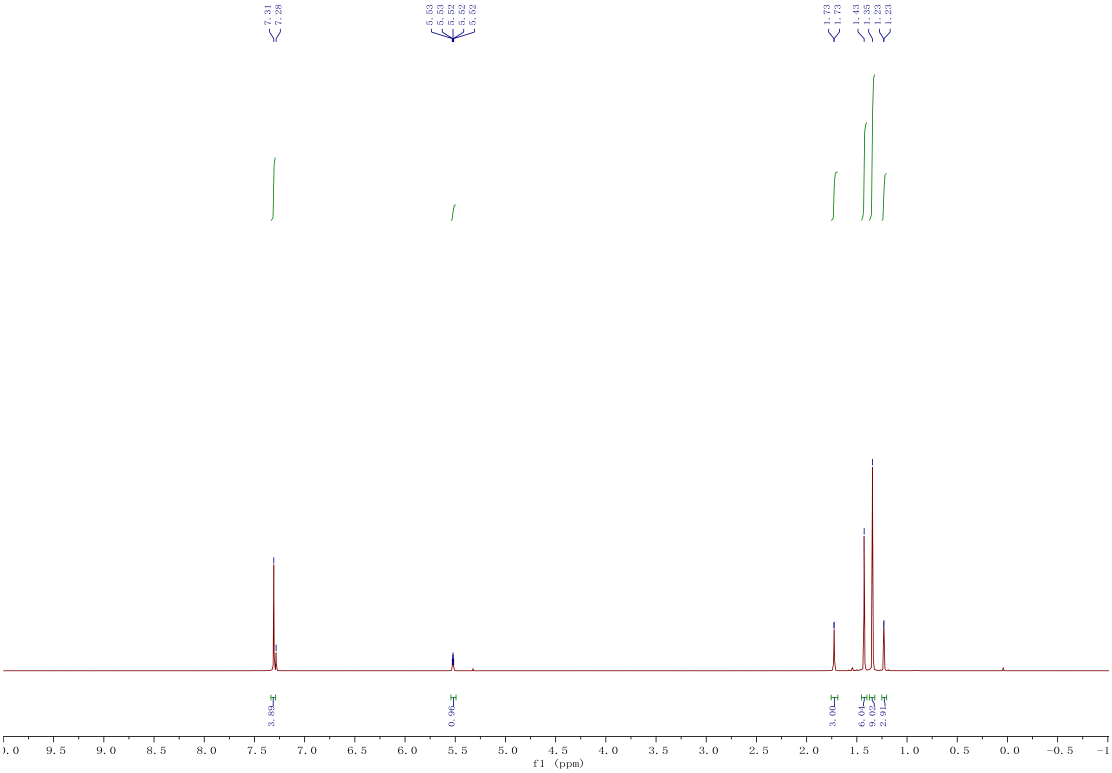

Supplementary Figure 95. ^{1}**H NMR** (400 MHz, Chloroform-*d*) of **sub. 4c**

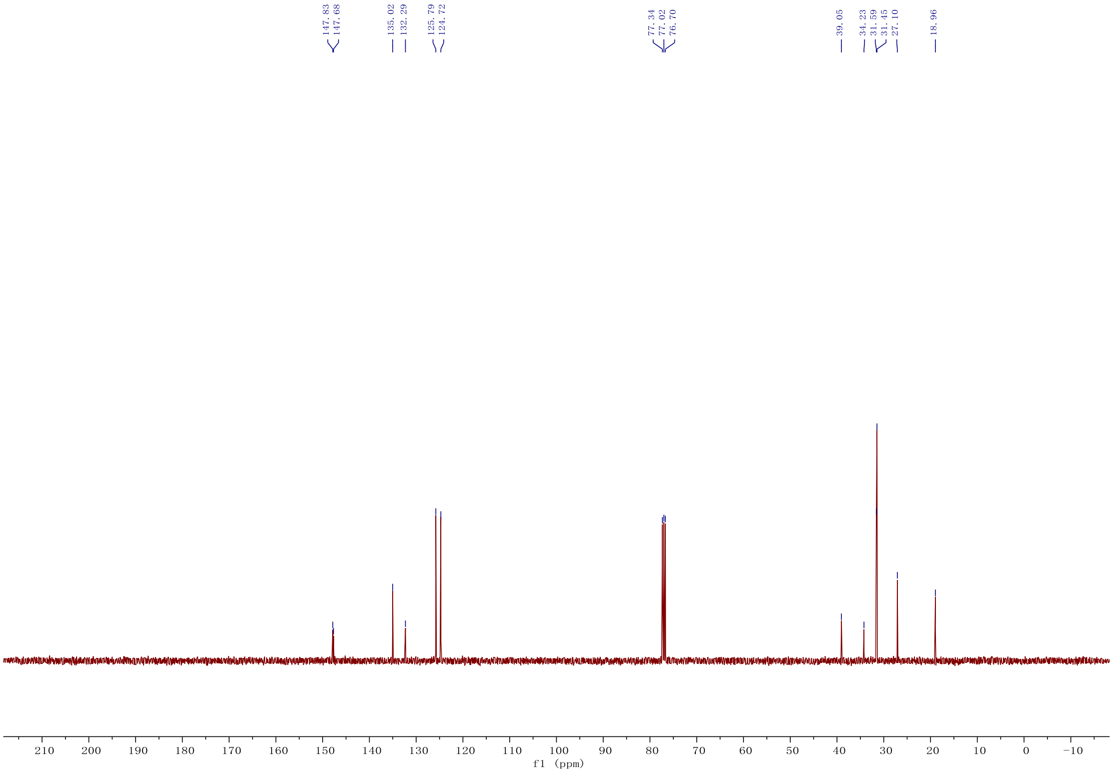

Supplementary Figure 96. ^{13}**C NMR** (101 MHz, Chloroform-*d*) of **sub. 4c**

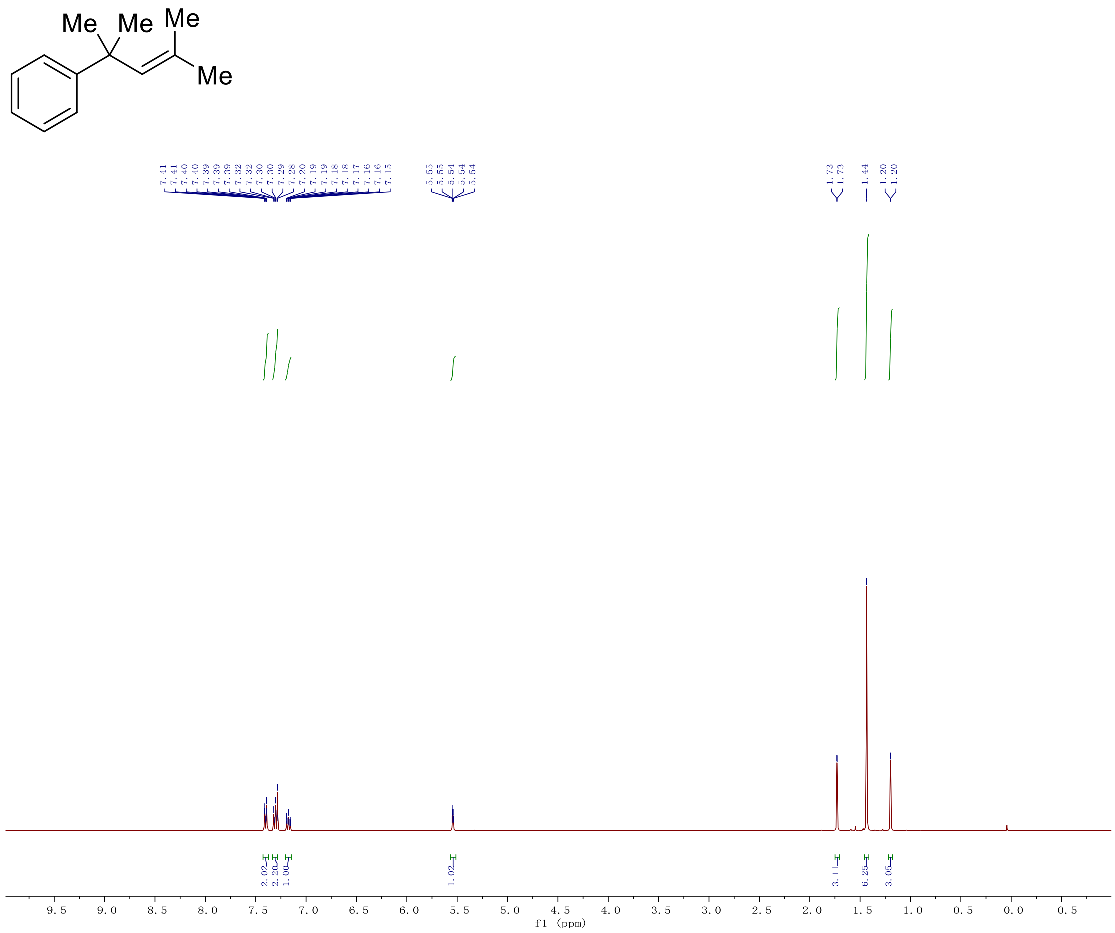

Supplementary Figure 97. ^{1}H NMR (400 MHz, Chloroform-*d*) of **sub. 4d**

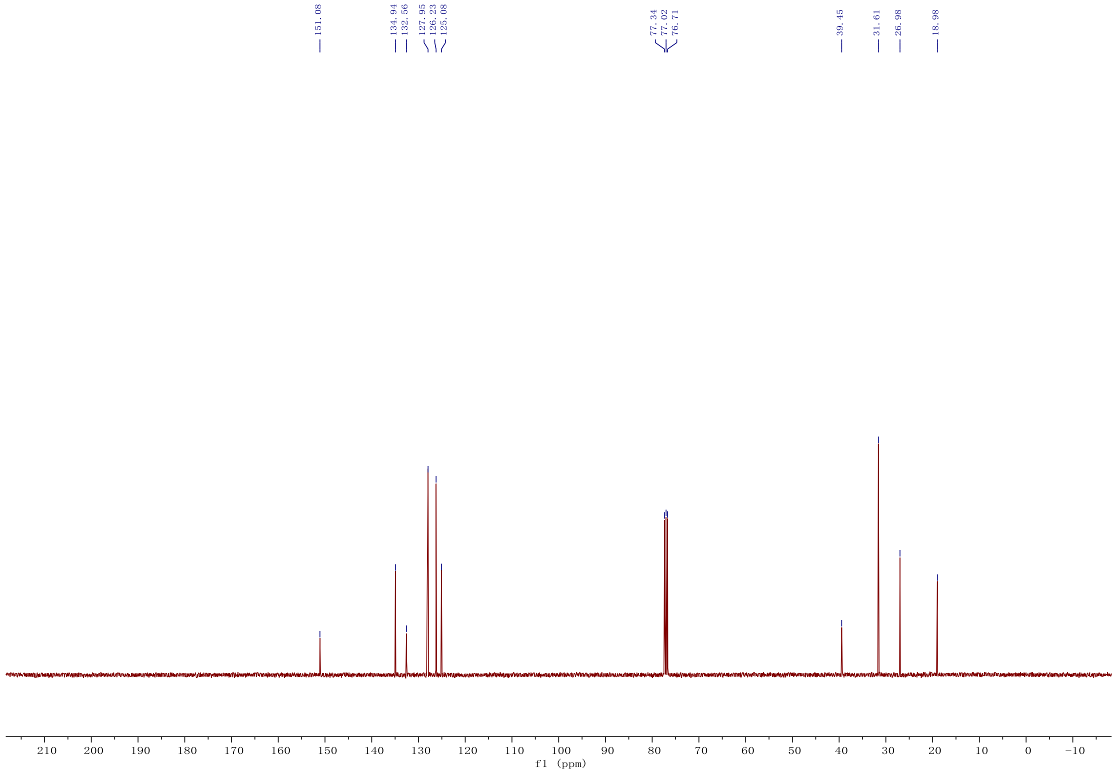

Supplementary Figure 98. ^{13}C NMR (101 MHz, Chloroform-*d*) of **sub. 4d**

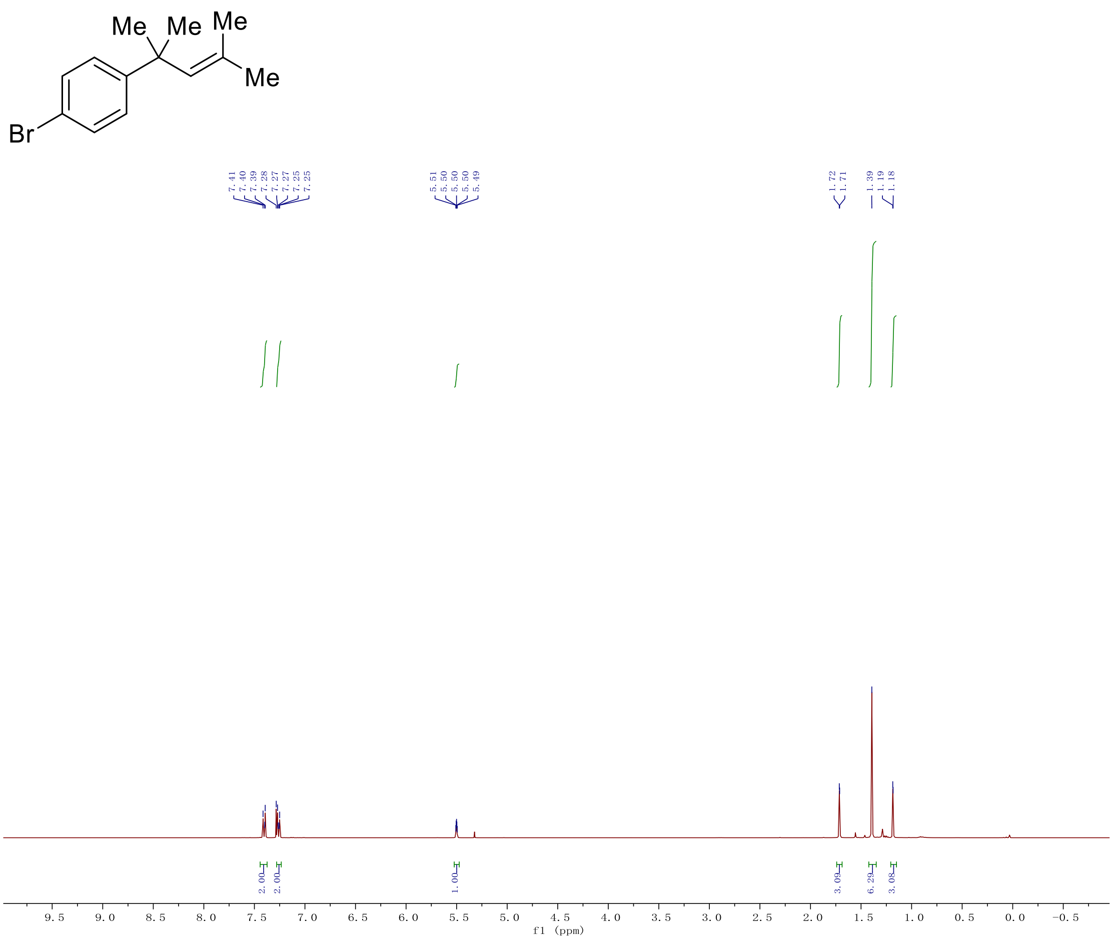

Supplementary Figure 99. ^{1}**H NMR** (400 MHz, Chloroform-*d*) of **sub. 4e**

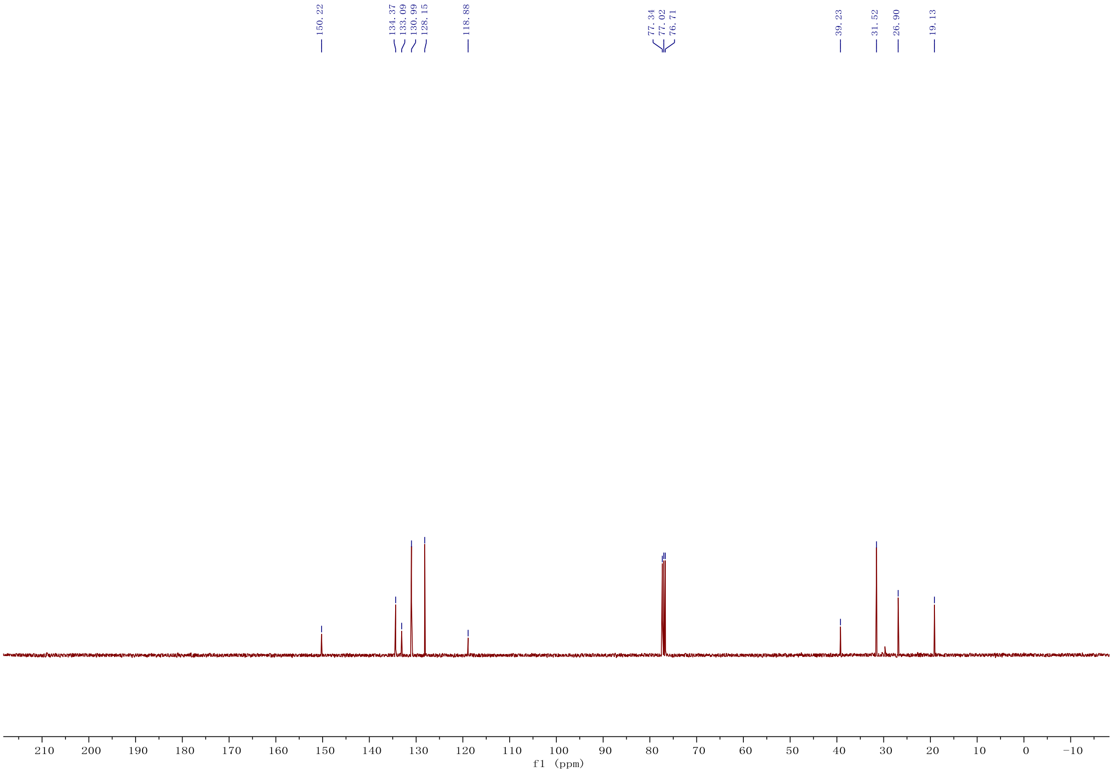

Supplementary Figure 100. ^{13}**C NMR** (101 MHz, Chloroform-*d*) of **sub. 4e**

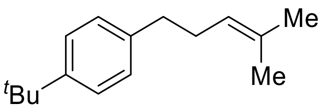

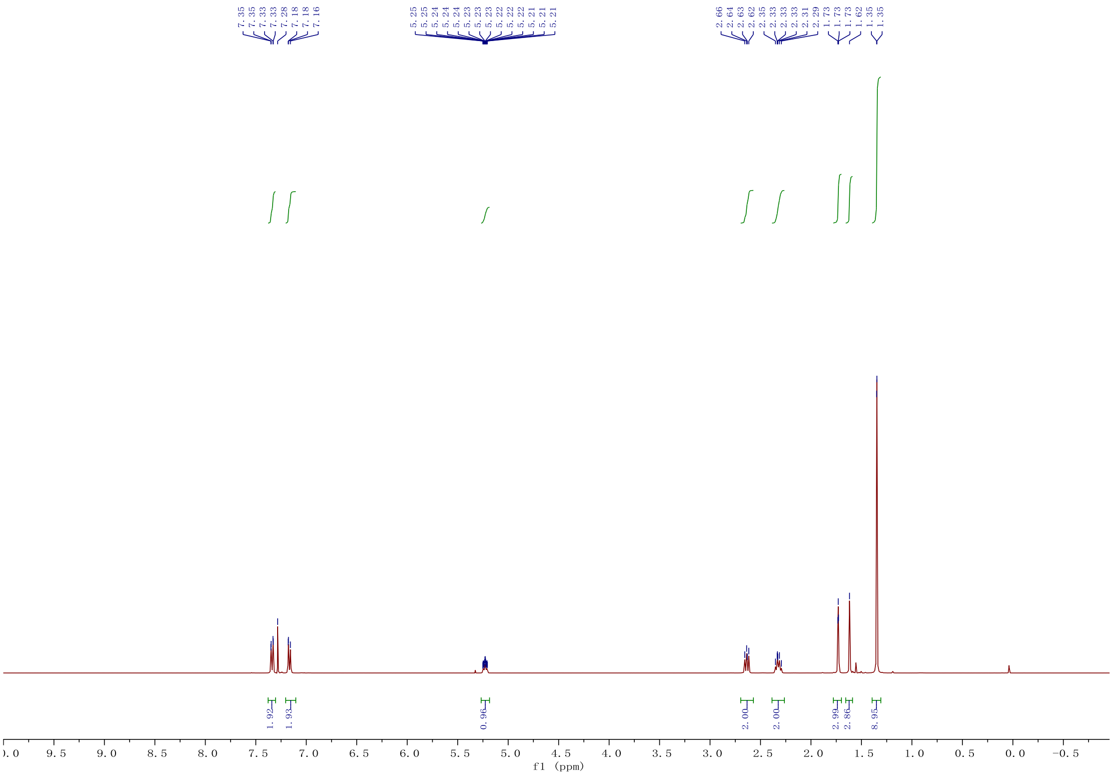

Supplementary Figure 101. ^{1}H NMR (400 MHz, Chloroform-*d*) of **sub. 4f**

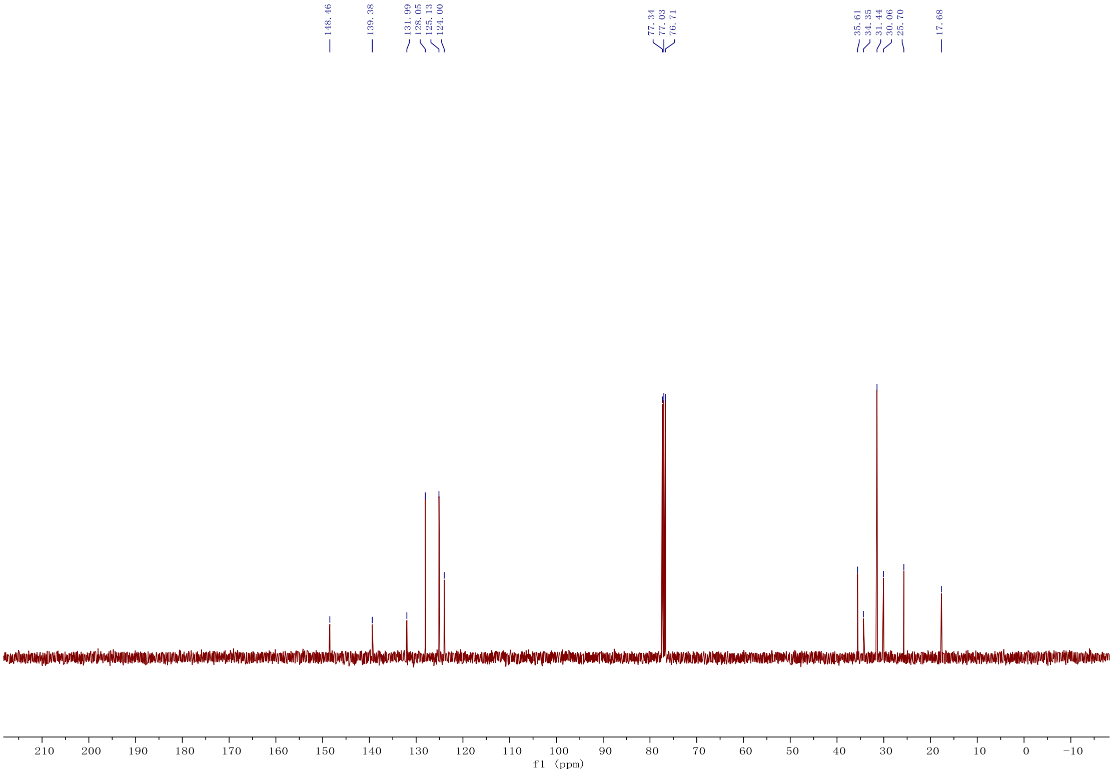

Supplementary Figure 102. ^{13}C **NMR** (101 MHz, Chloroform-*d*) of **sub. 4f**

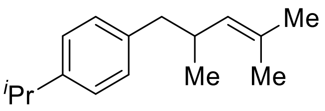

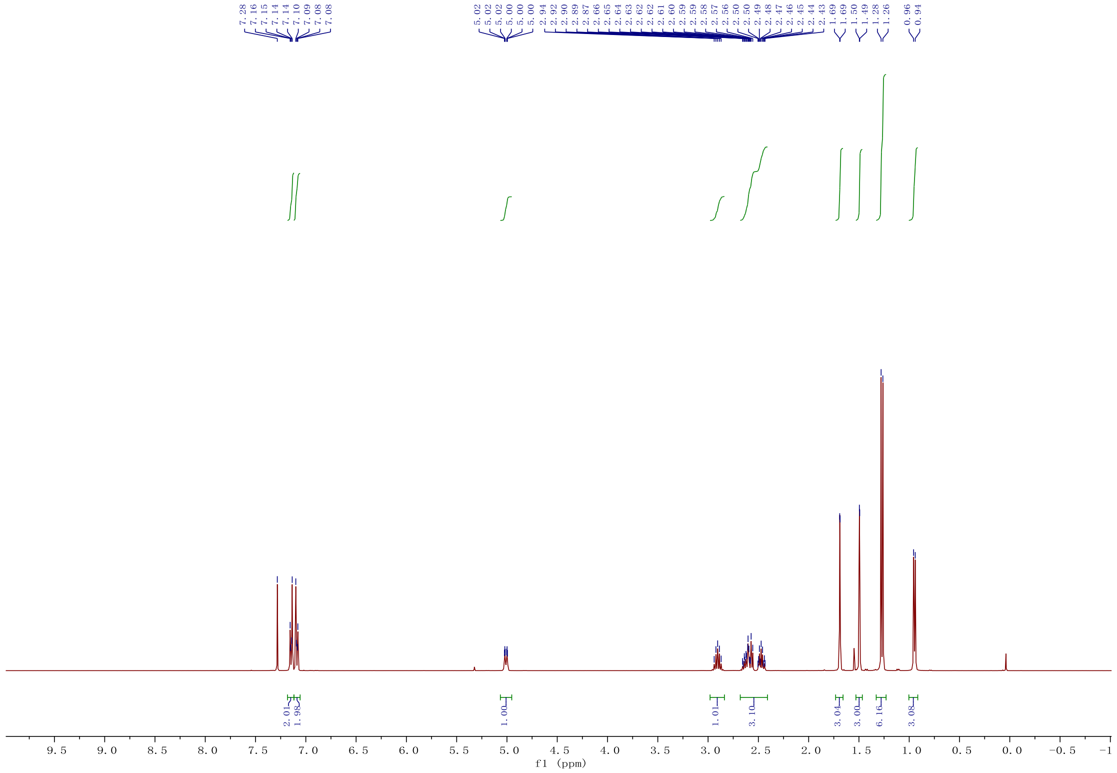

Supplementary Figure 103. ^{1}H NMR (400 MHz, Chloroform-*d*) of **sub. 4g**

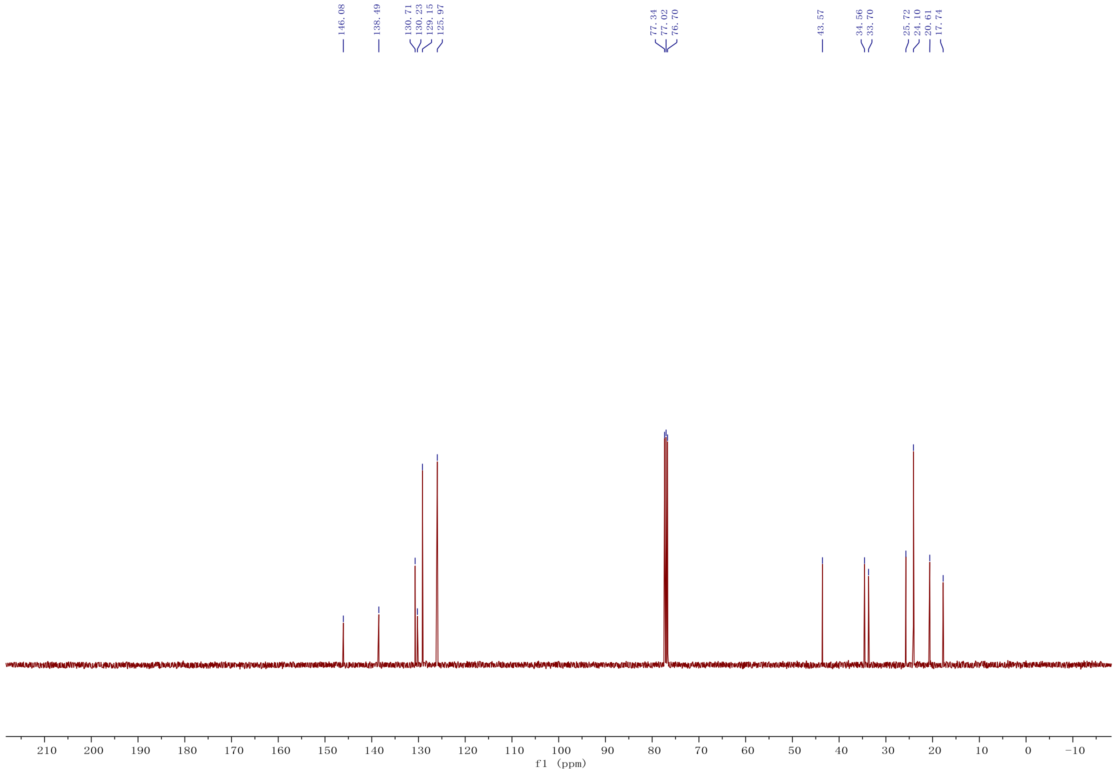

Supplementary Figure 104. ^{13}C NMR (101 MHz, Chloroform-*d*) of **sub. 4g**

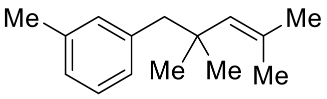

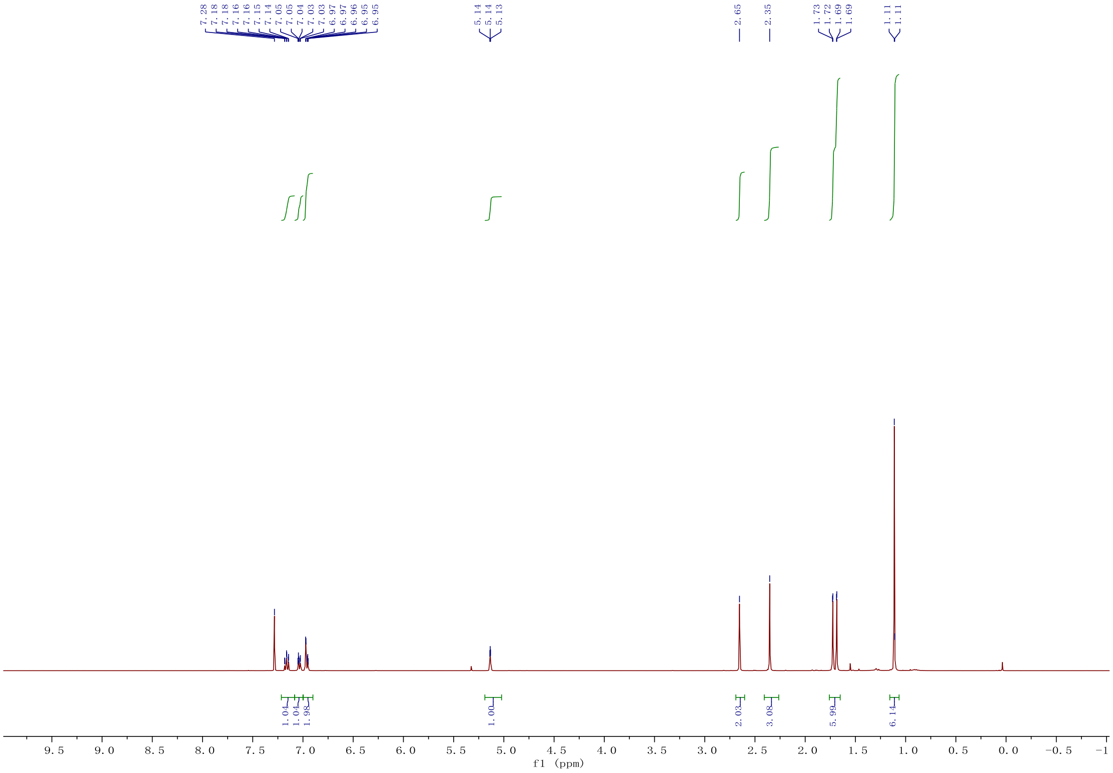

Supplementary Figure 105. ^{1}**H NMR** (400 MHz, Chloroform-*d*) of **sub. 4h**

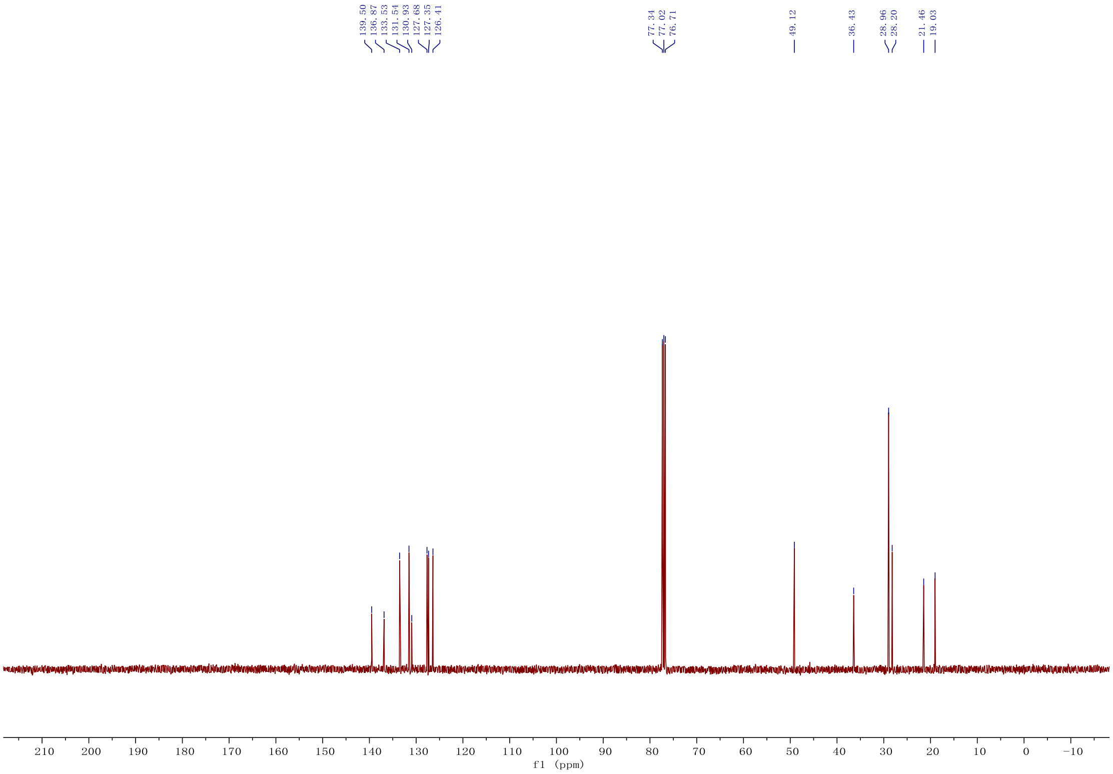

Supplementary Figure 106. ^{13}**C NMR** (101 MHz, Chloroform-*d*) of **sub. 4h**

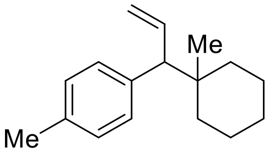

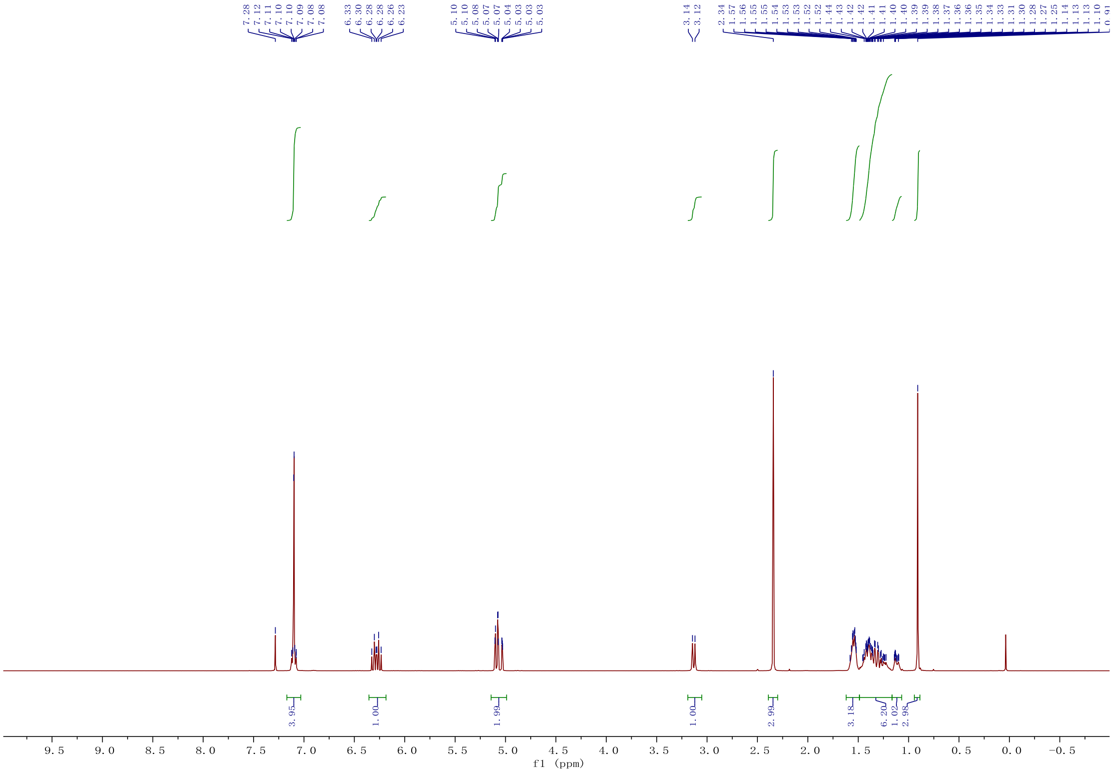

Supplementary Figure 107. **^{1}H NMR** (400 MHz, Chloroform-*d*) of **sub. 5a**

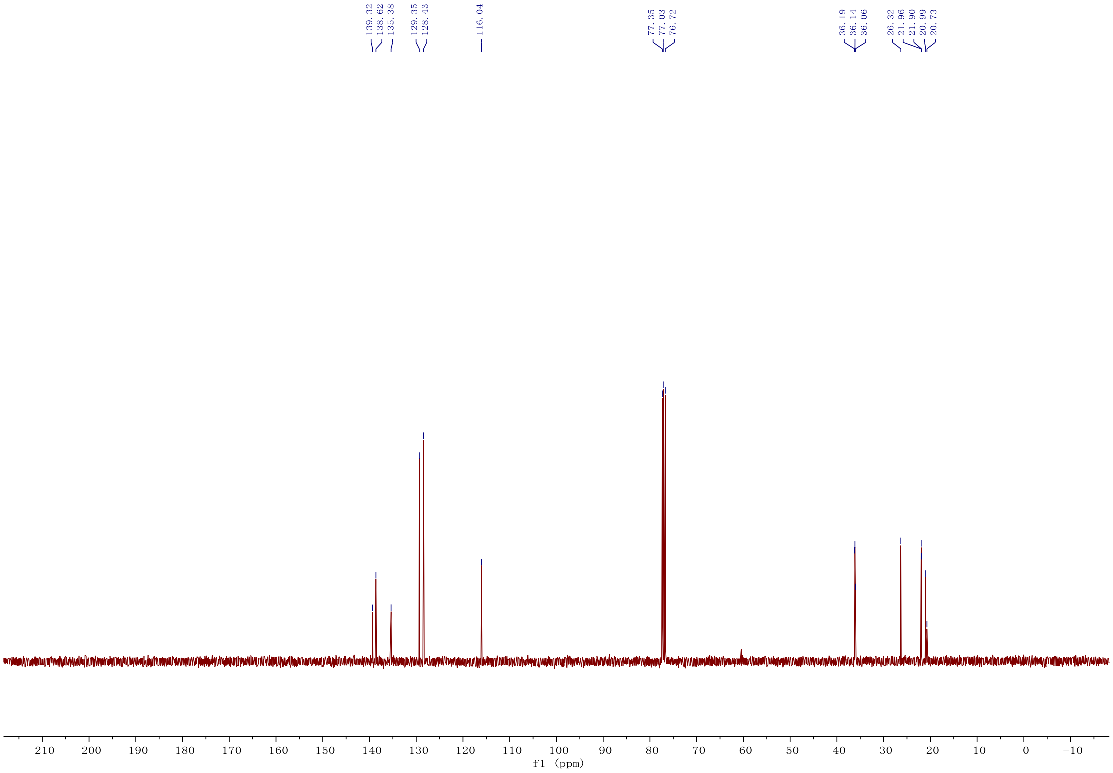

Supplementary Figure 108. **^{13}C NMR** (101 MHz, Chloroform-*d*) of **sub. 5a**

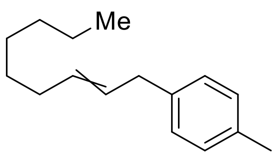

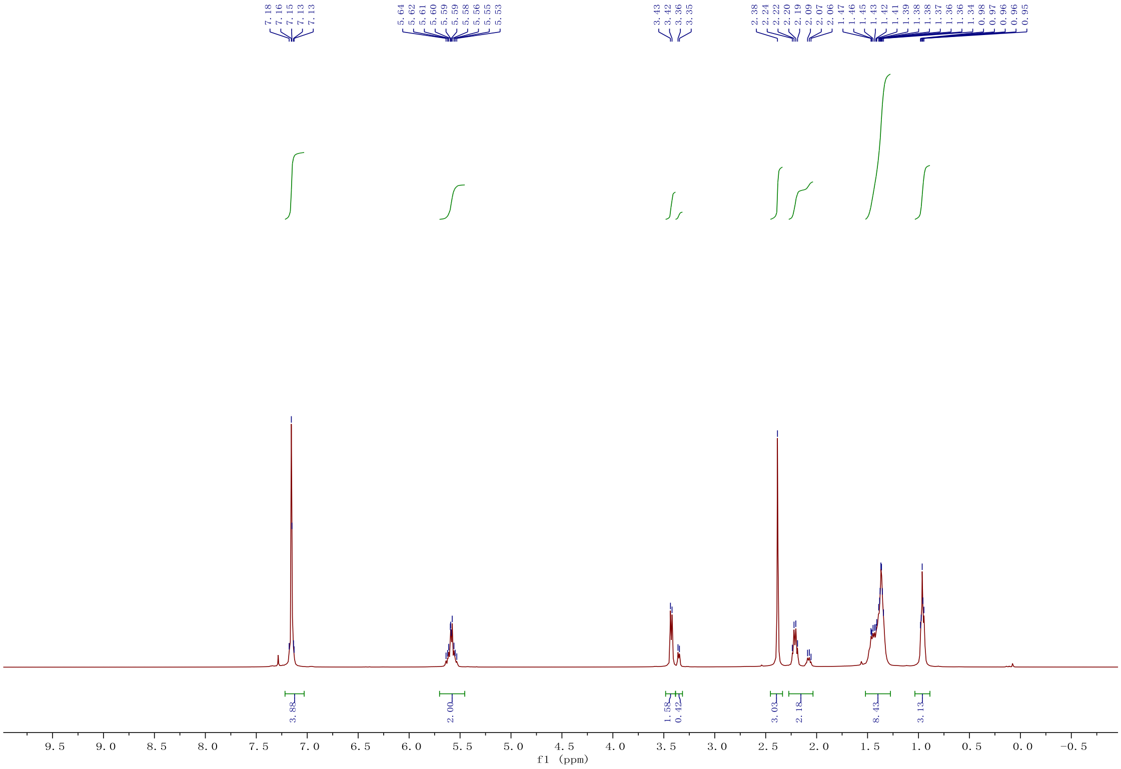

Supplementary Figure 109. ^{1}**H NMR** (400 MHz, Chloroform-*d*) of **sub. 5e**

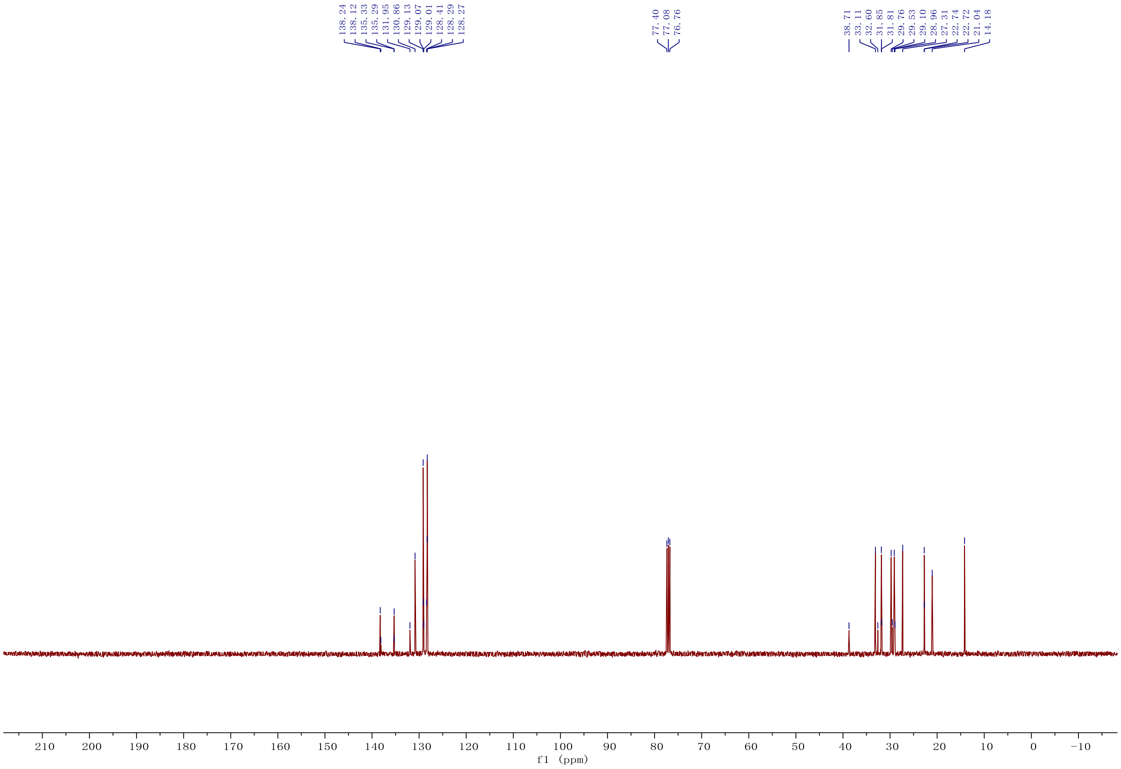

Supplementary Figure 110. ^{13}**C NMR** (101 MHz, Chloroform-*d*) of **sub. 5e**

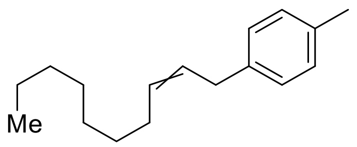

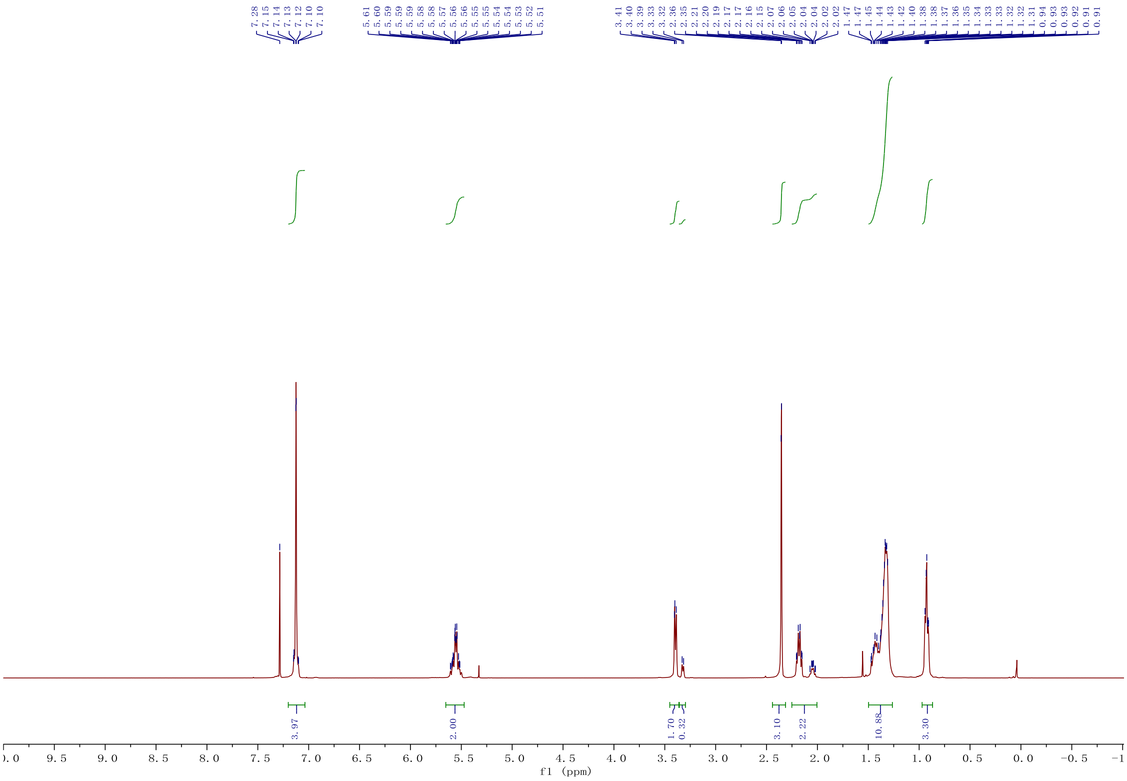

Supplementary Figure 111. ^{1}**H NMR** (400 MHz, Chloroform-*d*) of **sub. 5f**

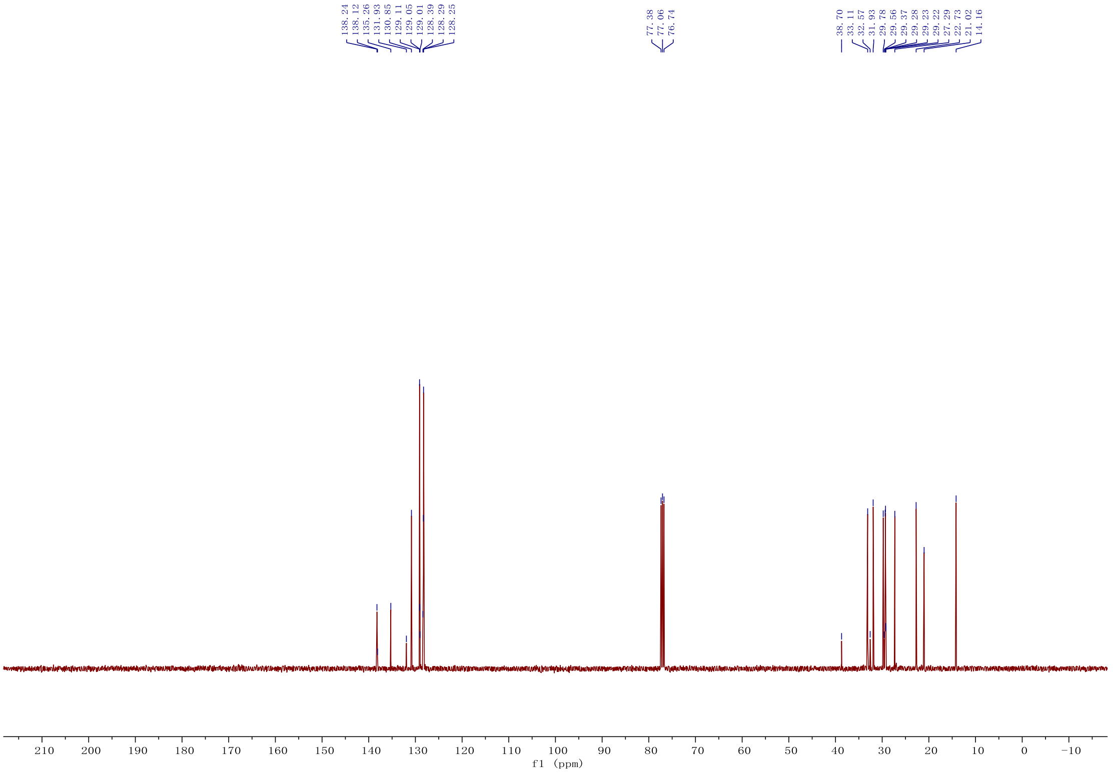

Supplementary Figure 112. ^{13}**C NMR** (101 MHz, Chloroform-*d*) of **sub. 5f**

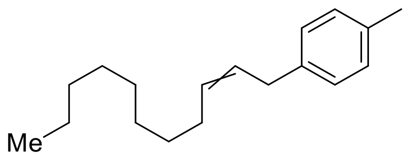

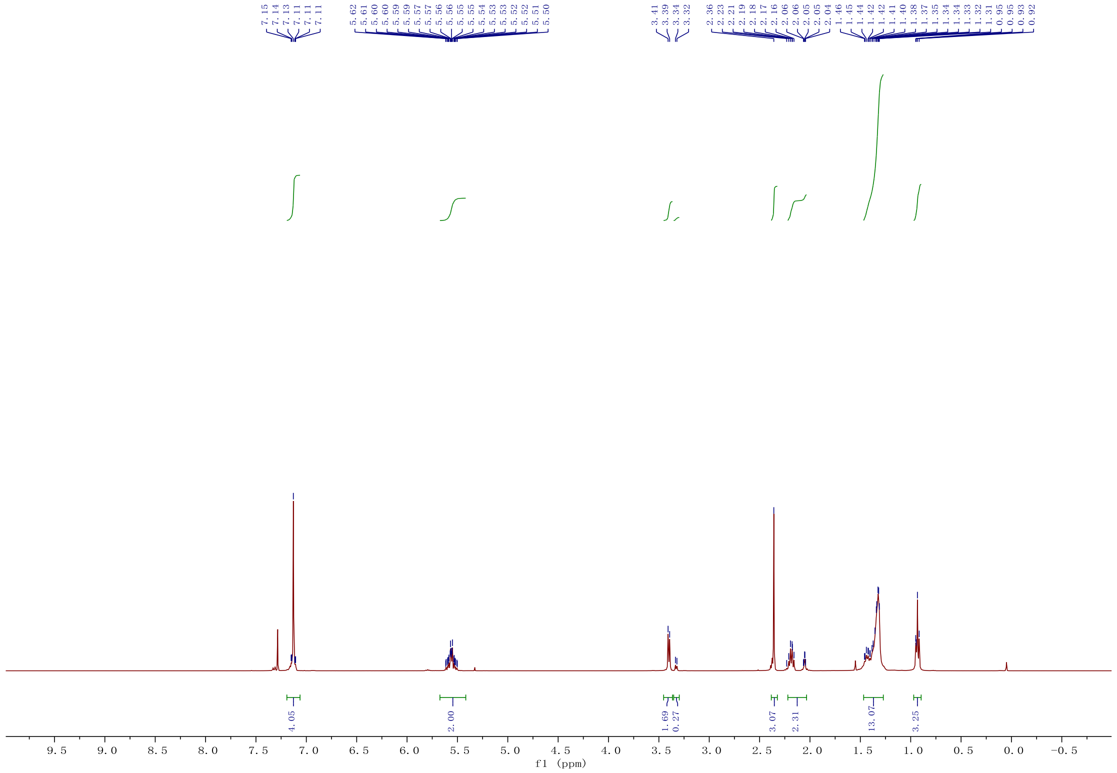

Supplementary Figure 113. ^{1}H NMR (400 MHz, Chloroform-*d*) of **sub. 5g**

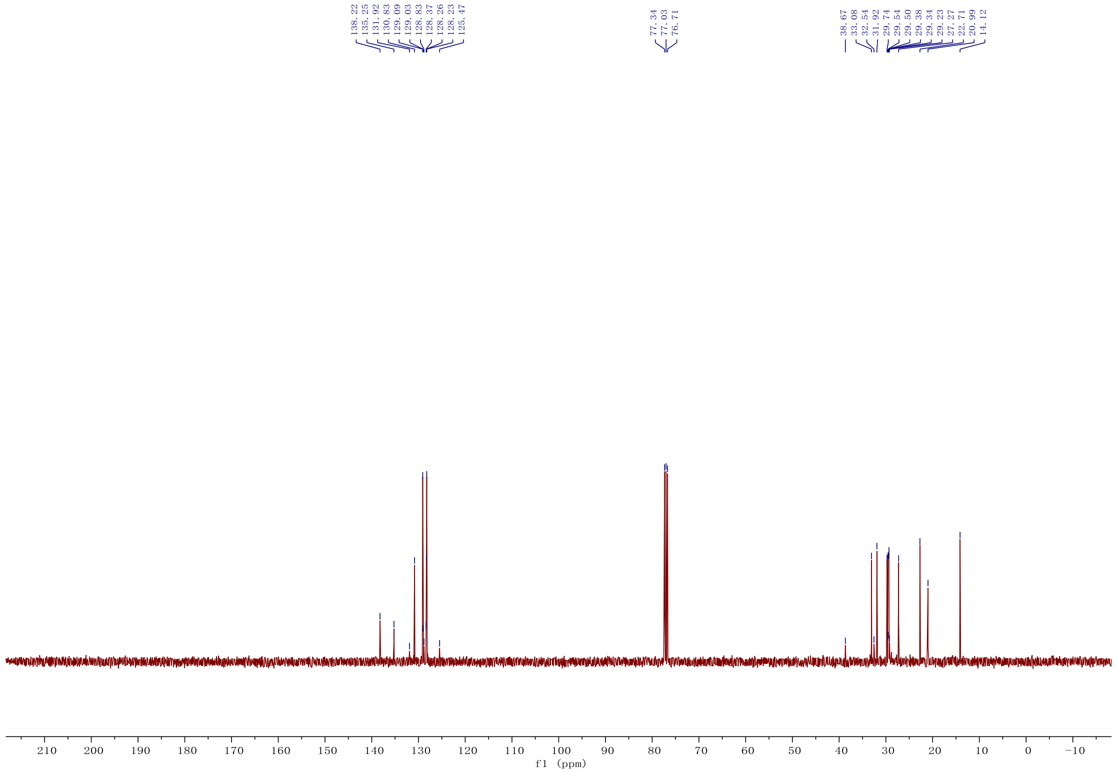

Supplementary Figure 114. ^{13}C NMR (101 MHz, Chloroform-*d*) of **sub. 5g**

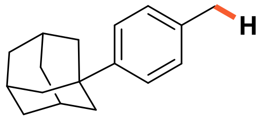

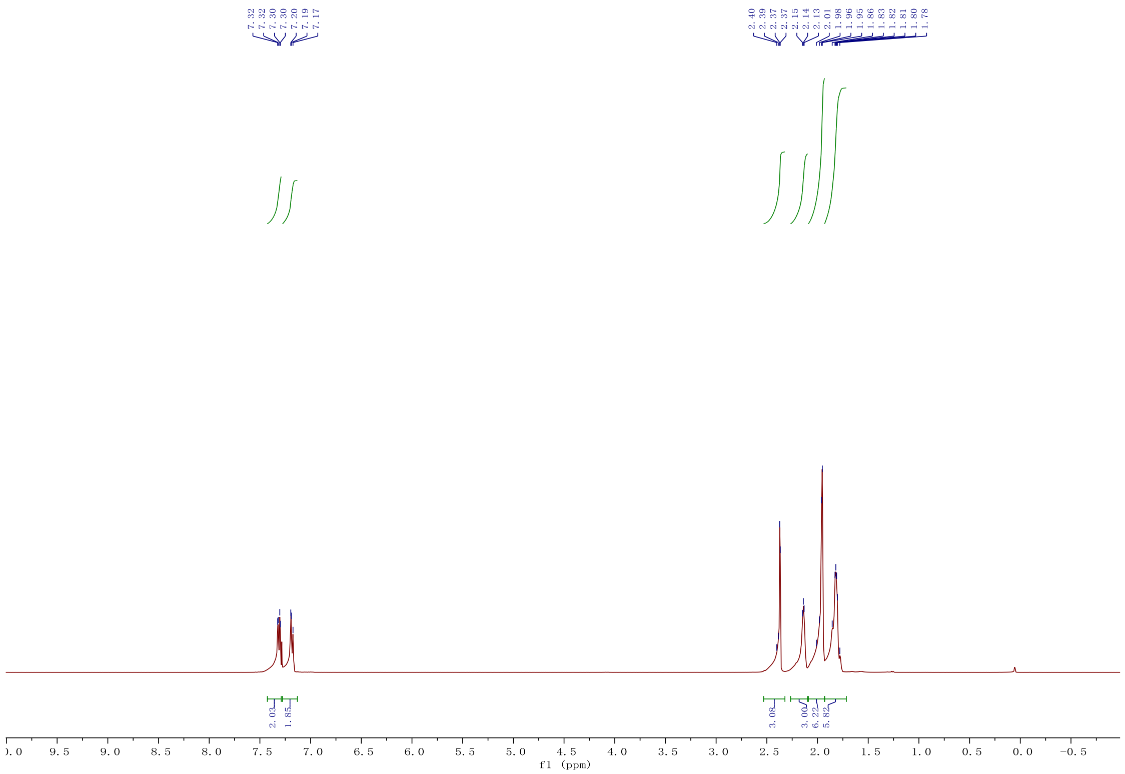

Supplementary Figure 115. ^{1}**H NMR** (400 MHz, Chloroform-*d*) of **2e**

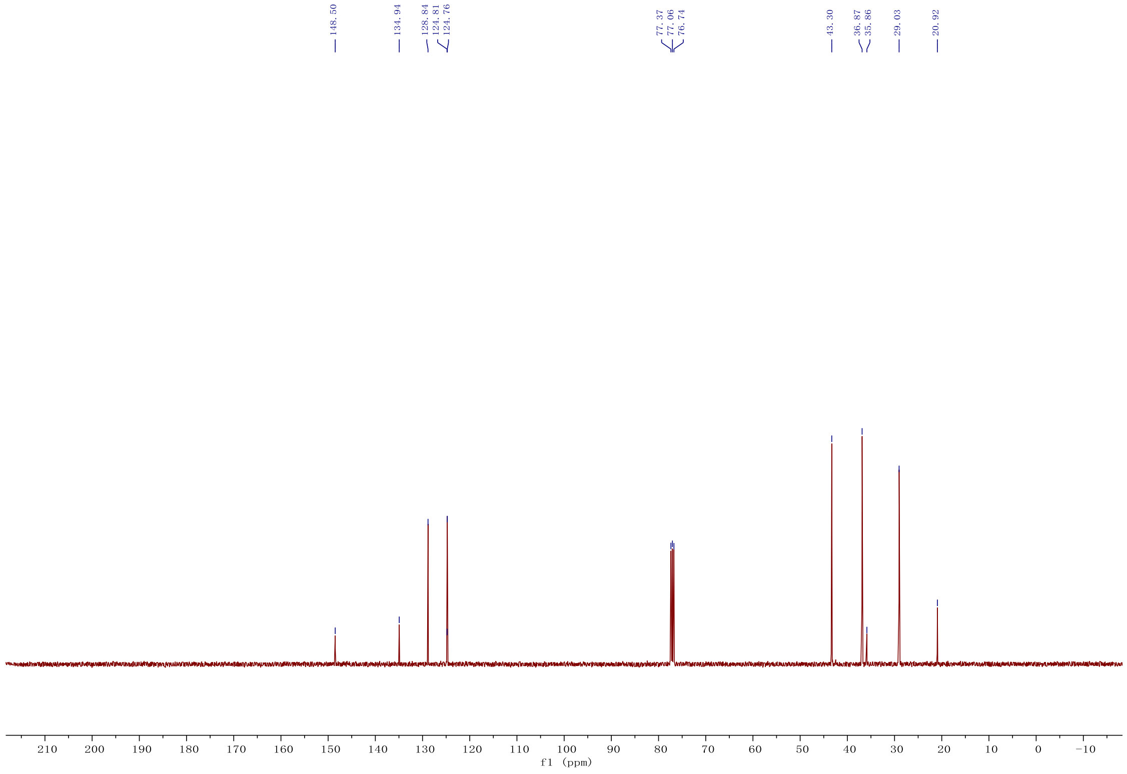

Supplementary Figure 116. ^{13}**C NMR** (101 MHz, Chloroform-*d*) of **2e**

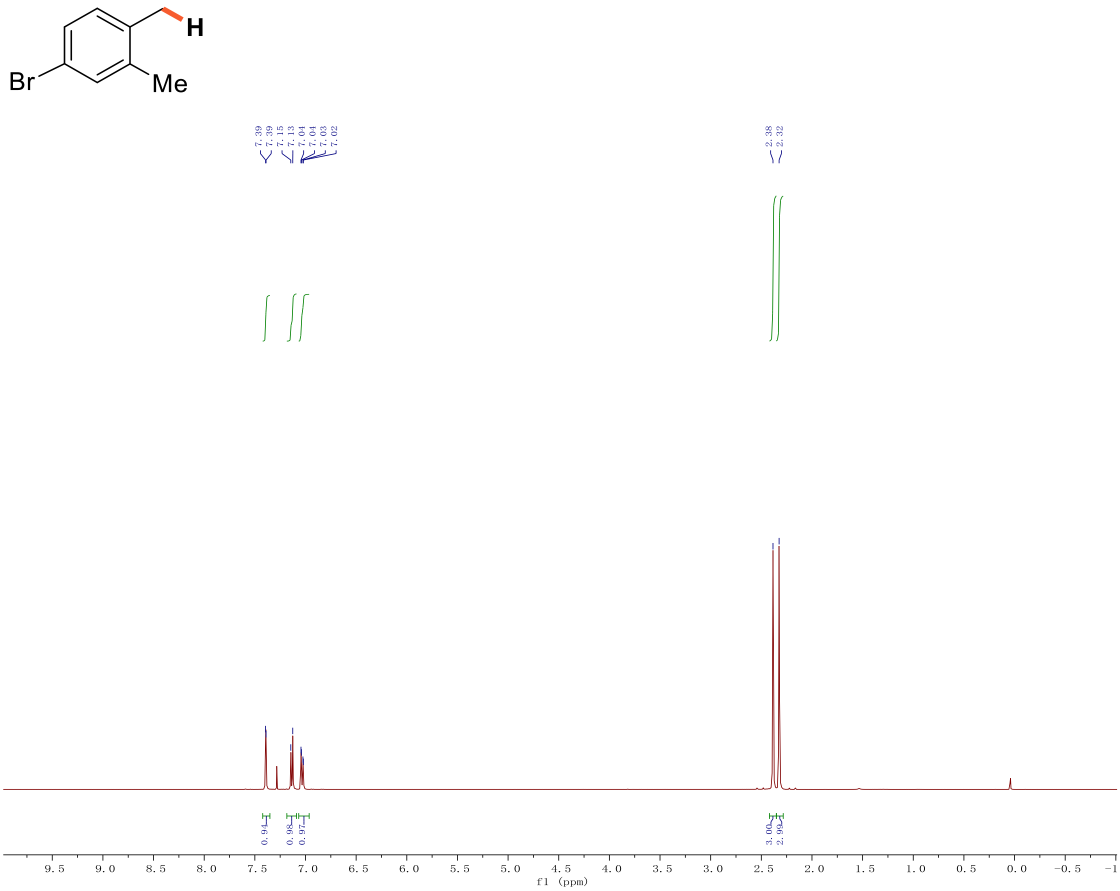

Supplementary Figure 117. ^{1}H NMR (400 MHz, Chloroform-*d*) of **2h**

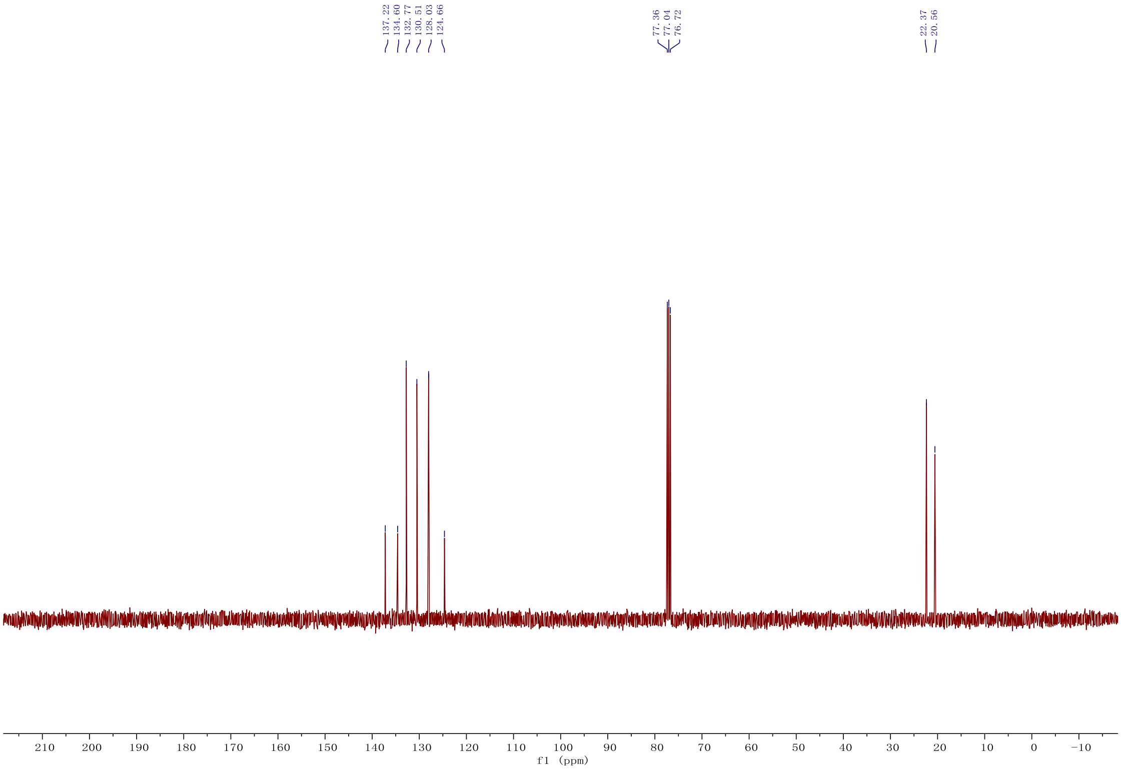

Supplementary Figure 118. ^{13}C NMR (101 MHz, Chloroform-*d*) of **2h**

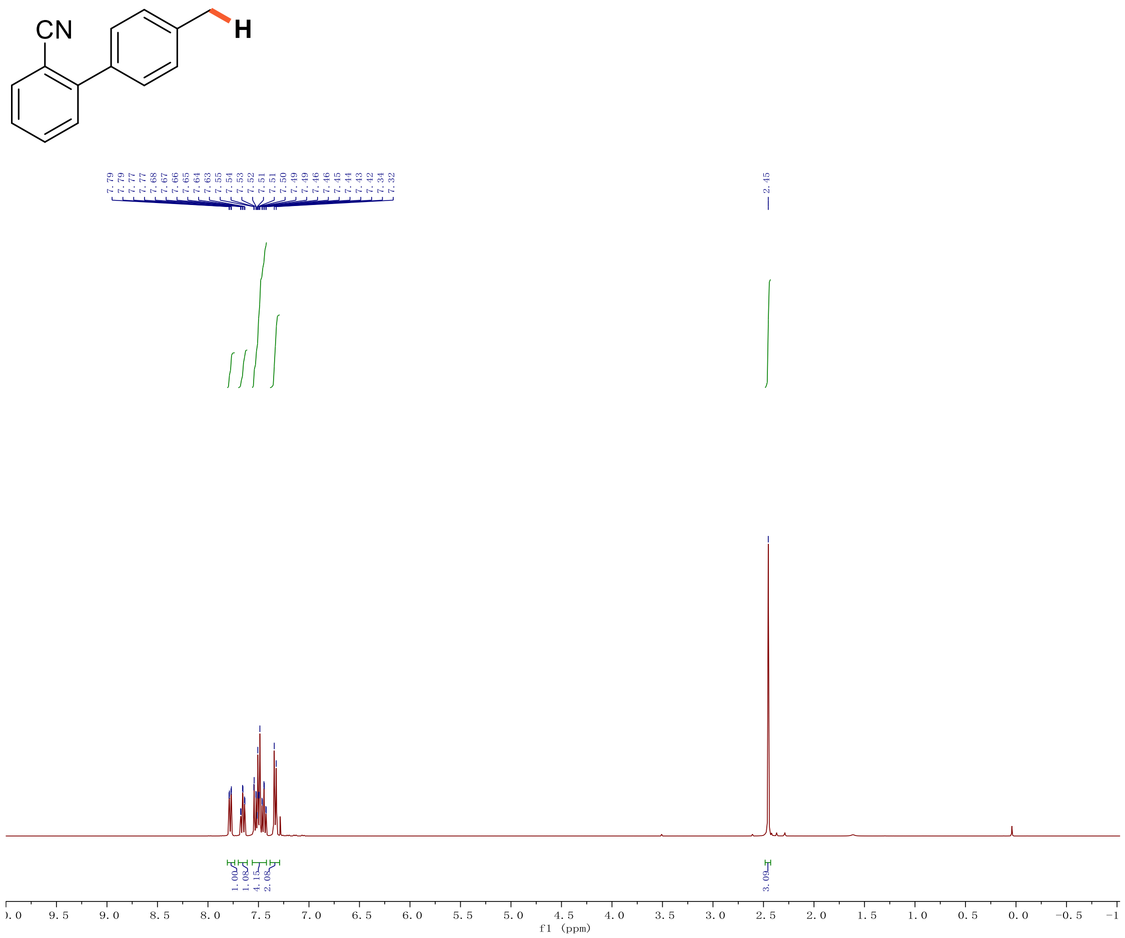

Supplementary Figure 119. ¹H NMR (400 MHz, Chloroform-*d*) of **2i**

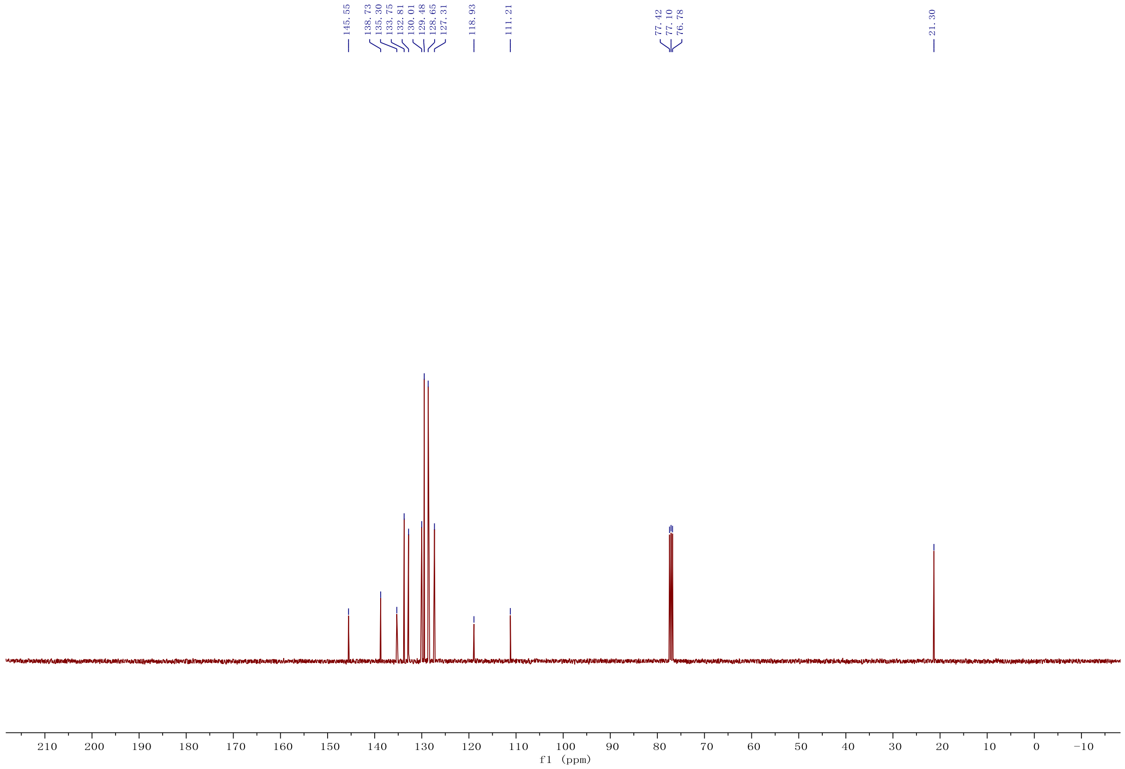

Supplementary Figure 120. ¹³C NMR (101 MHz, Chloroform-*d*) of **2i**

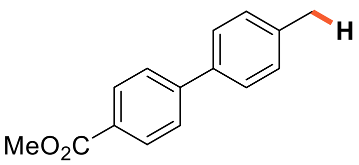

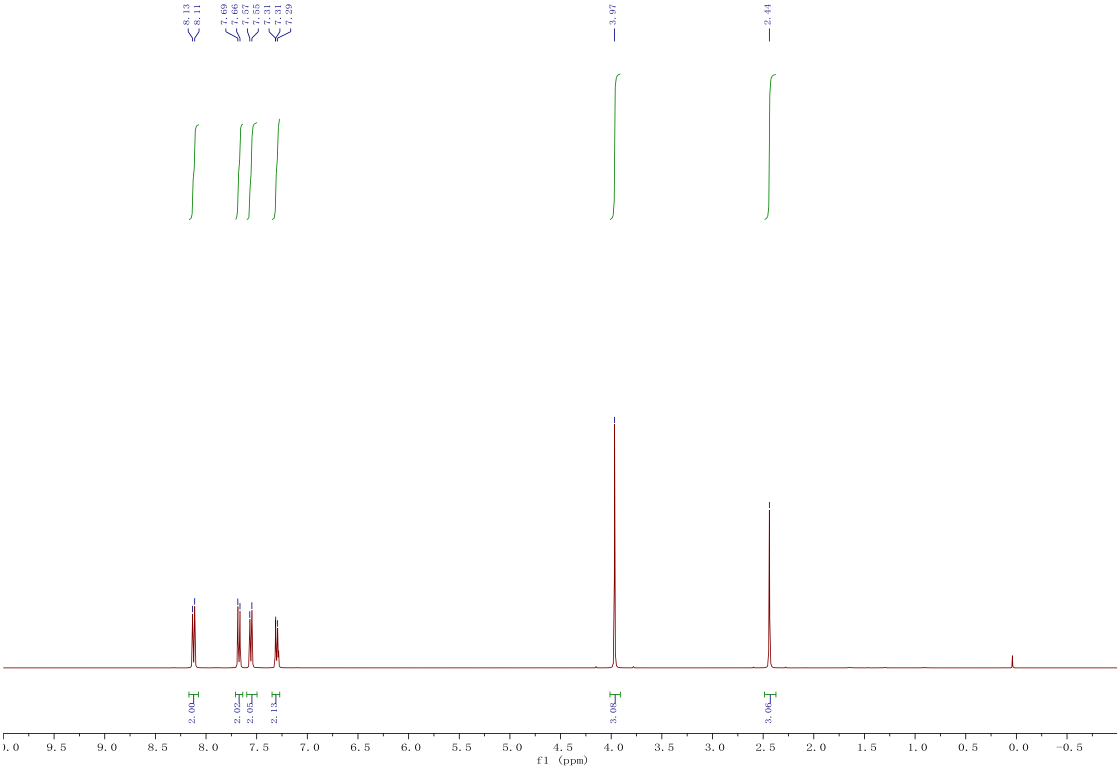

Supplementary Figure 121. **^{1}H NMR** (400 MHz, Chloroform-*d*) of **2j**

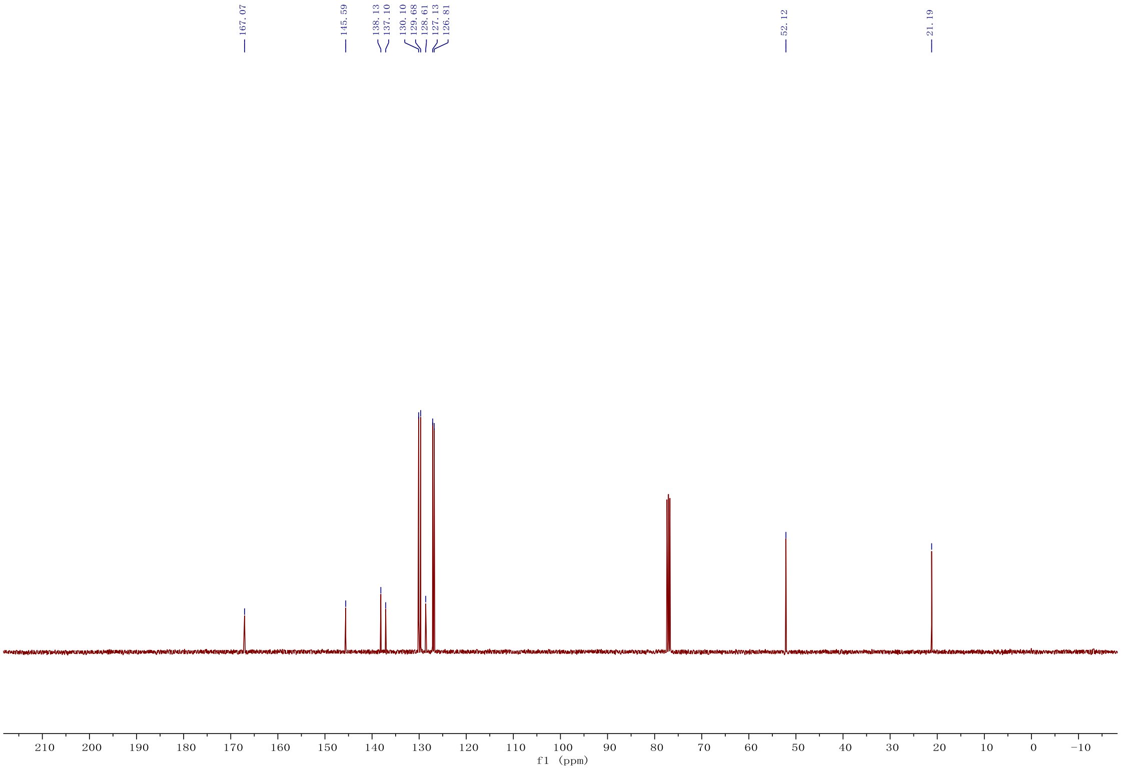

Supplementary Figure 122. **^{13}C NMR** (101 MHz, Chloroform-*d*) of **2j**

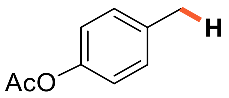

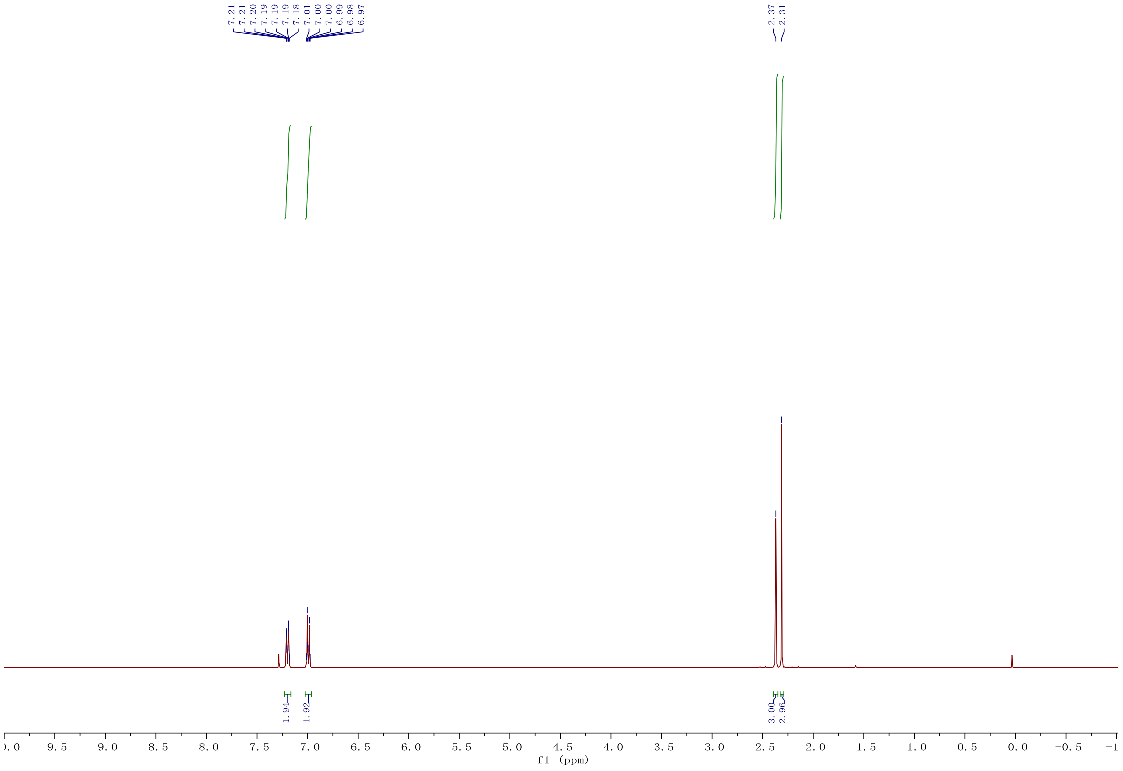

Supplementary Figure 123. ^{1}H NMR (400 MHz, Chloroform-*d*) of **2k**

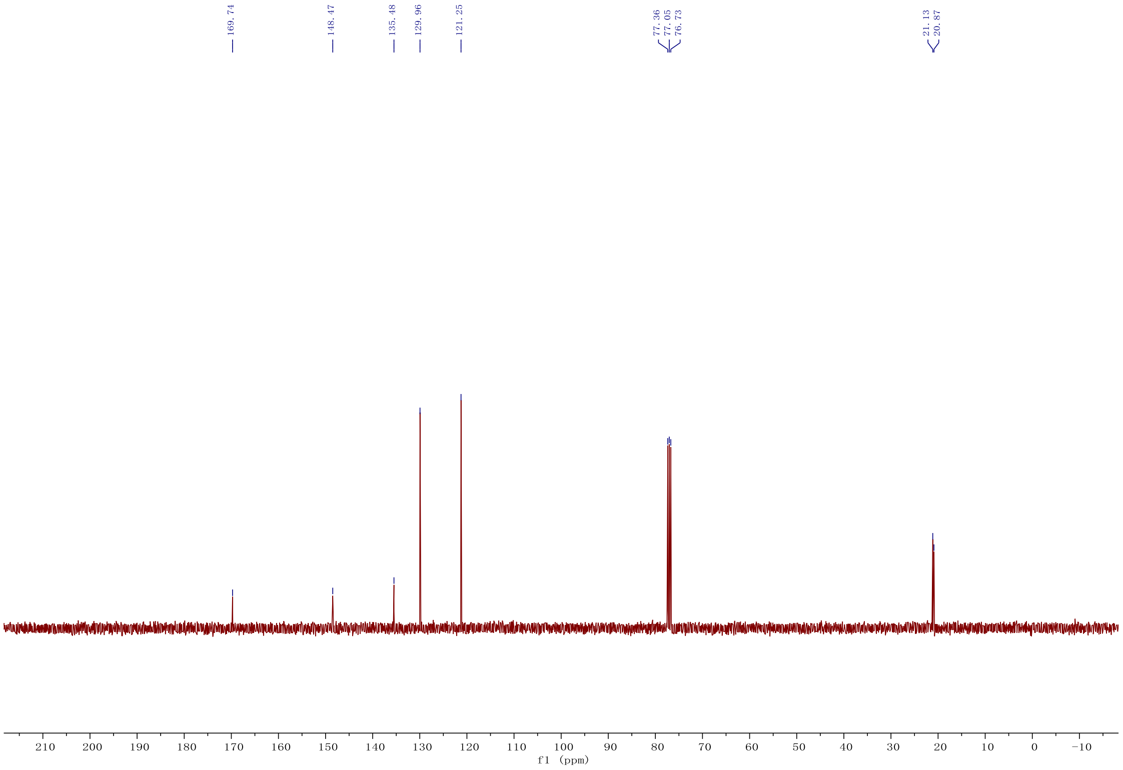

Supplementary Figure 124. ^{13}C NMR (101 MHz, Chloroform-*d*) of **2k**

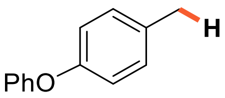

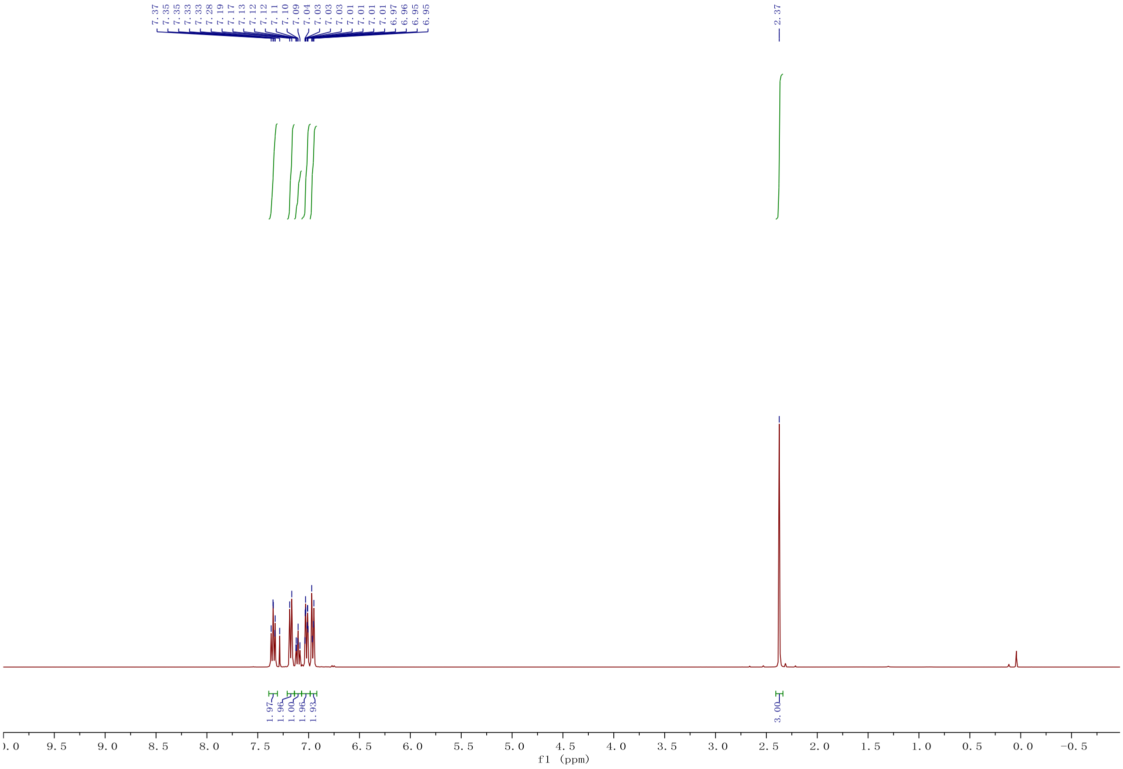

Supplementary Figure 125. ^{1}H NMR (400 MHz, Chloroform-*d*) of **2l**

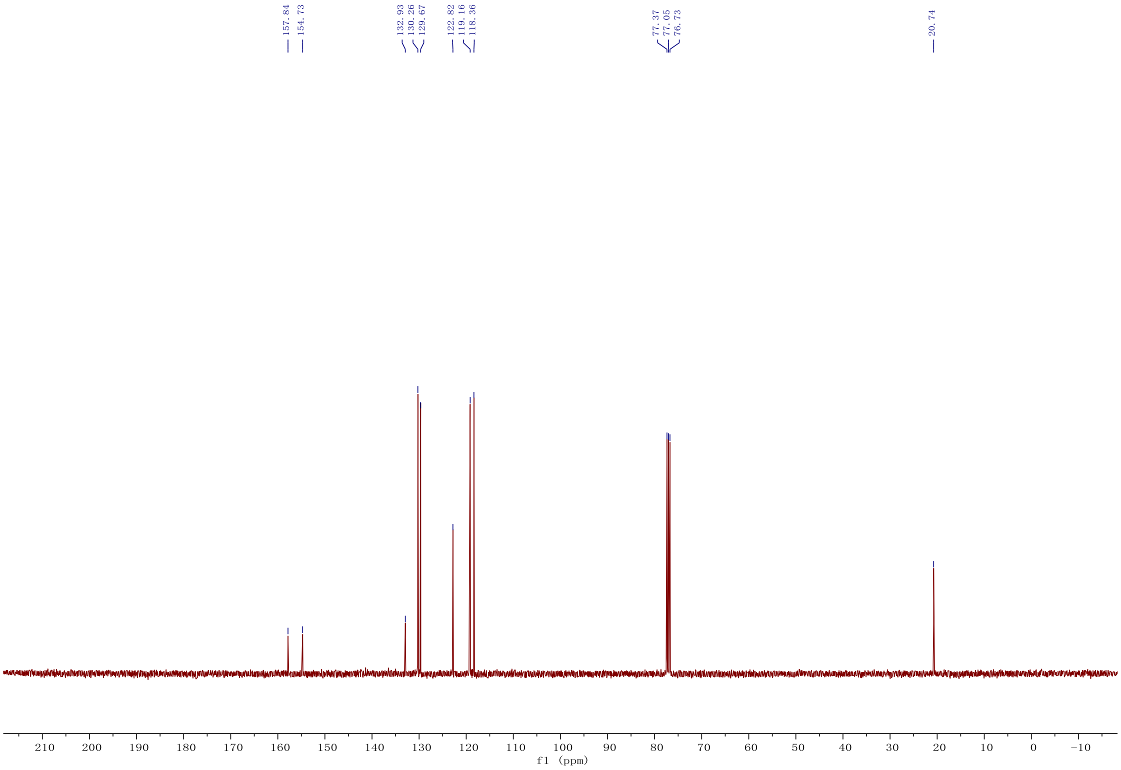

Supplementary Figure 126. ^{13}C NMR (101 MHz, Chloroform-*d*) of **2l**

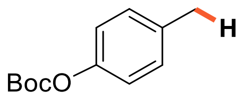

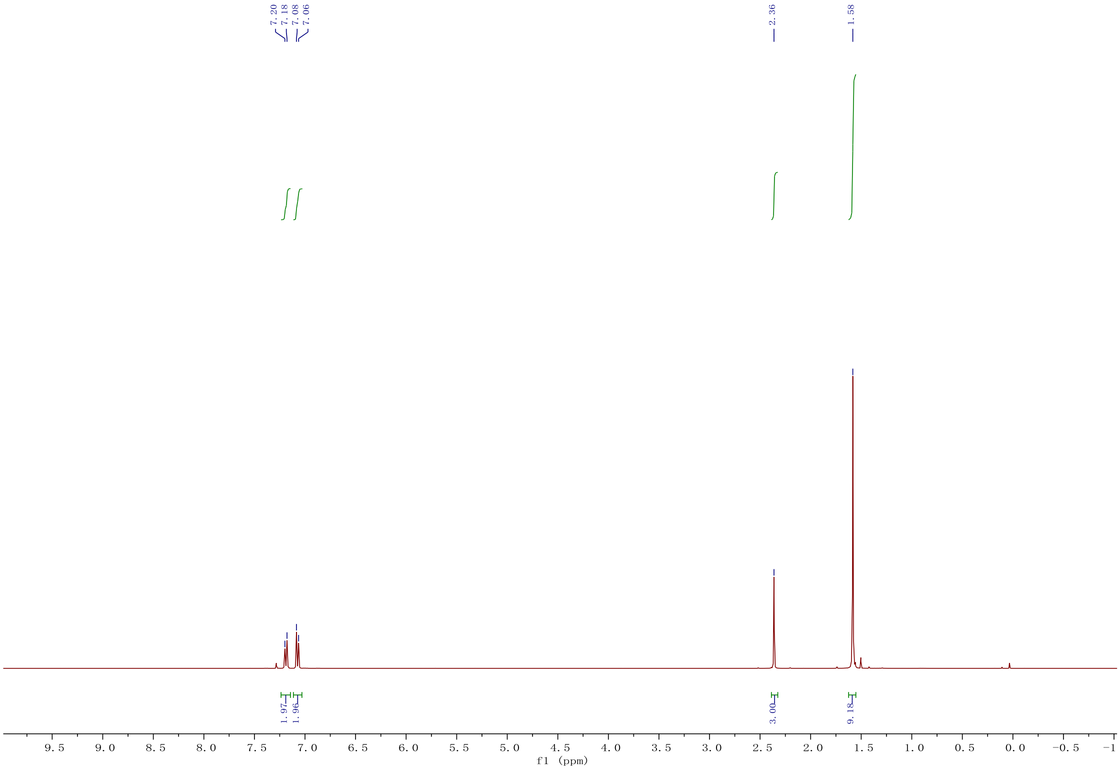

Supplementary Figure 127. ^{1}H NMR (400 MHz, Chloroform-*d*) of **2m**

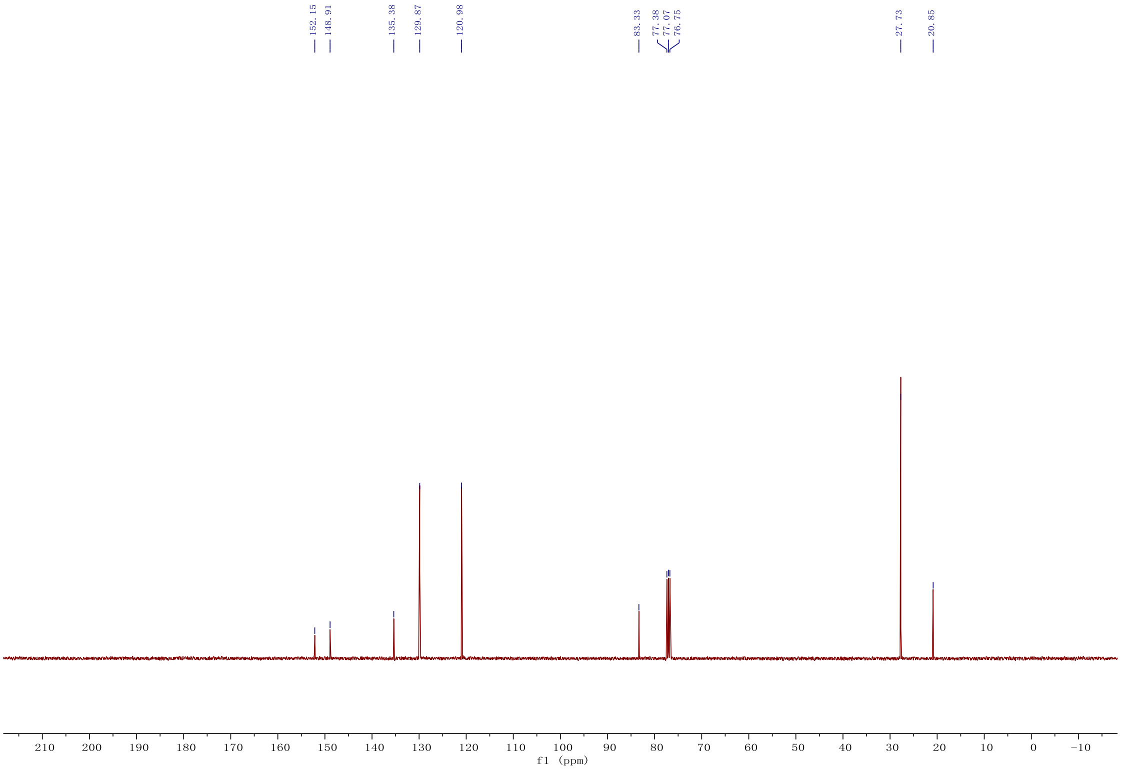

Supplementary Figure 128. ^{13}C NMR (101 MHz, Chloroform-*d*) of **2m**

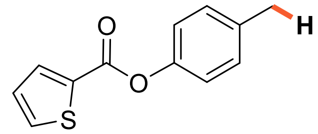

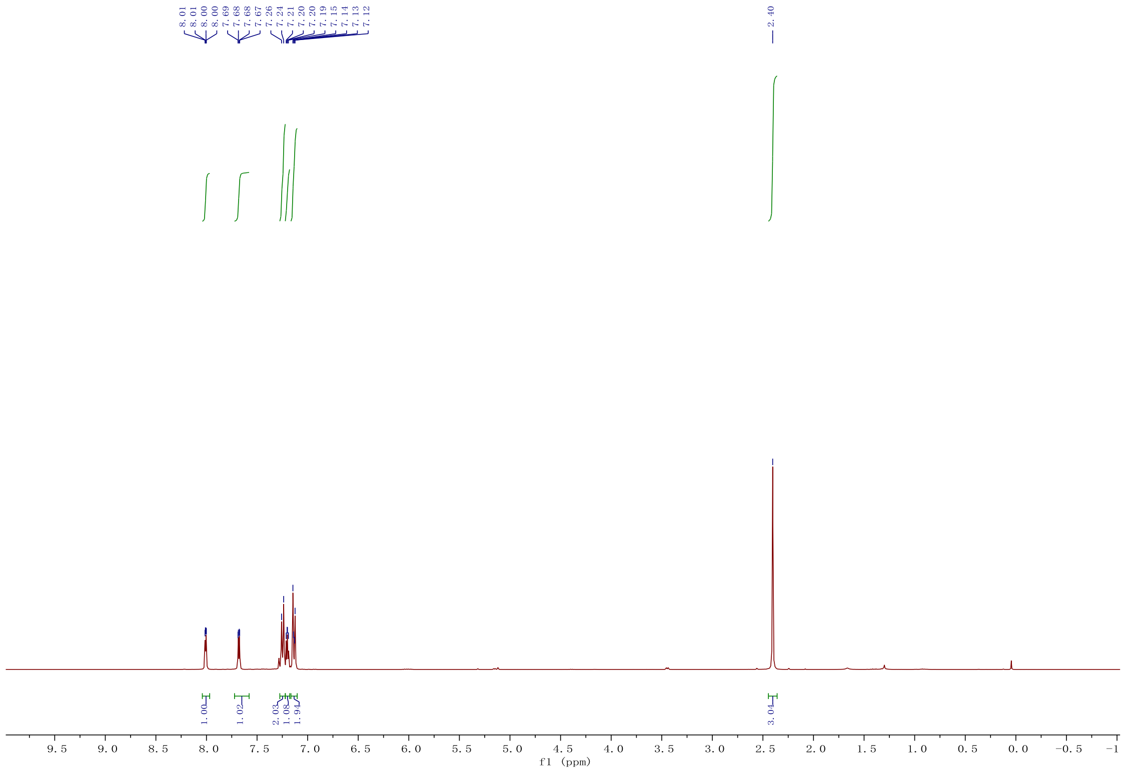

Supplementary Figure 129. ¹H NMR (400 MHz, Chloroform-*d*) of **2n**

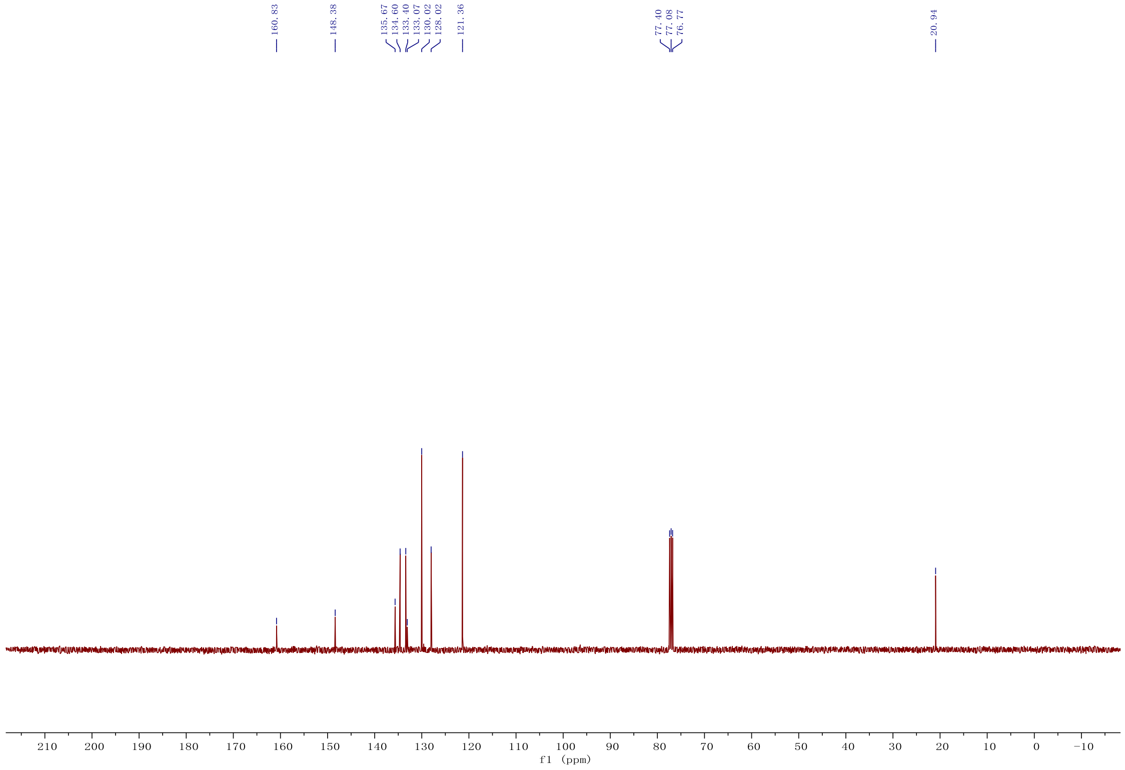

Supplementary Figure 130. ¹³C NMR (101 MHz, Chloroform-*d*) of **2n**

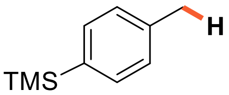

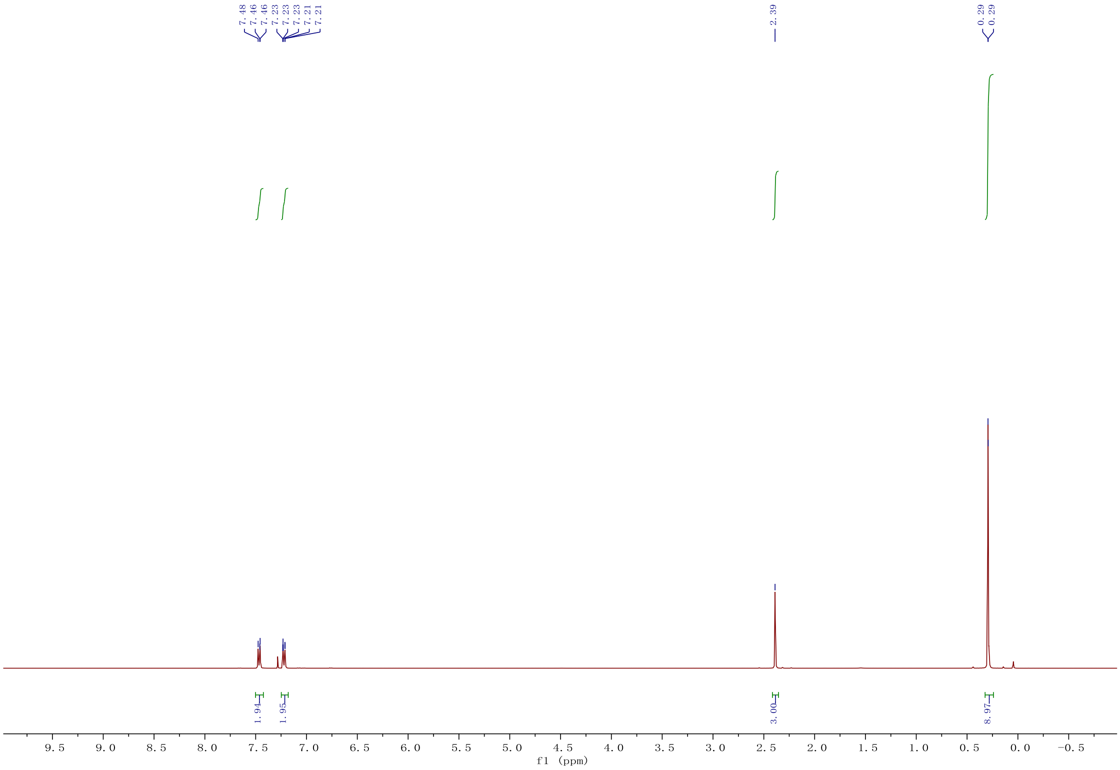

Supplementary Figure 131. ^{1}**H NMR** (400 MHz, Chloroform-*d*) of **2o**

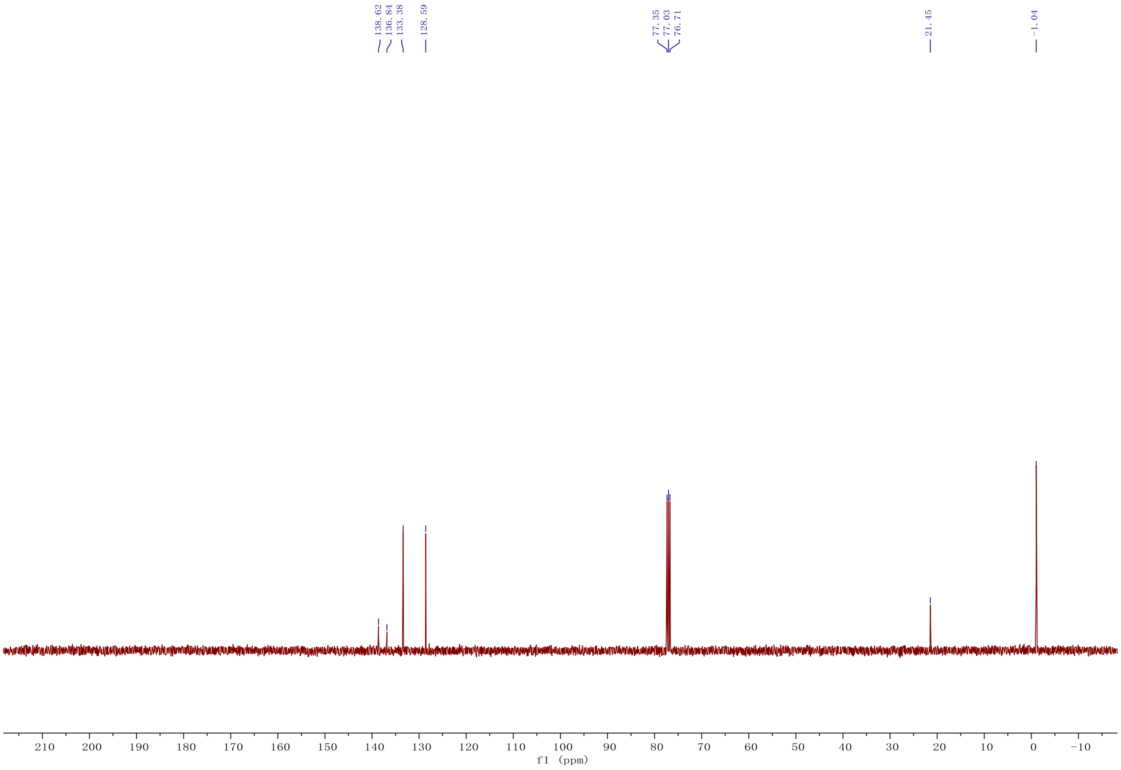

Supplementary Figure 132. ^{13}**C NMR** (101 MHz, Chloroform-*d*) of **2o**

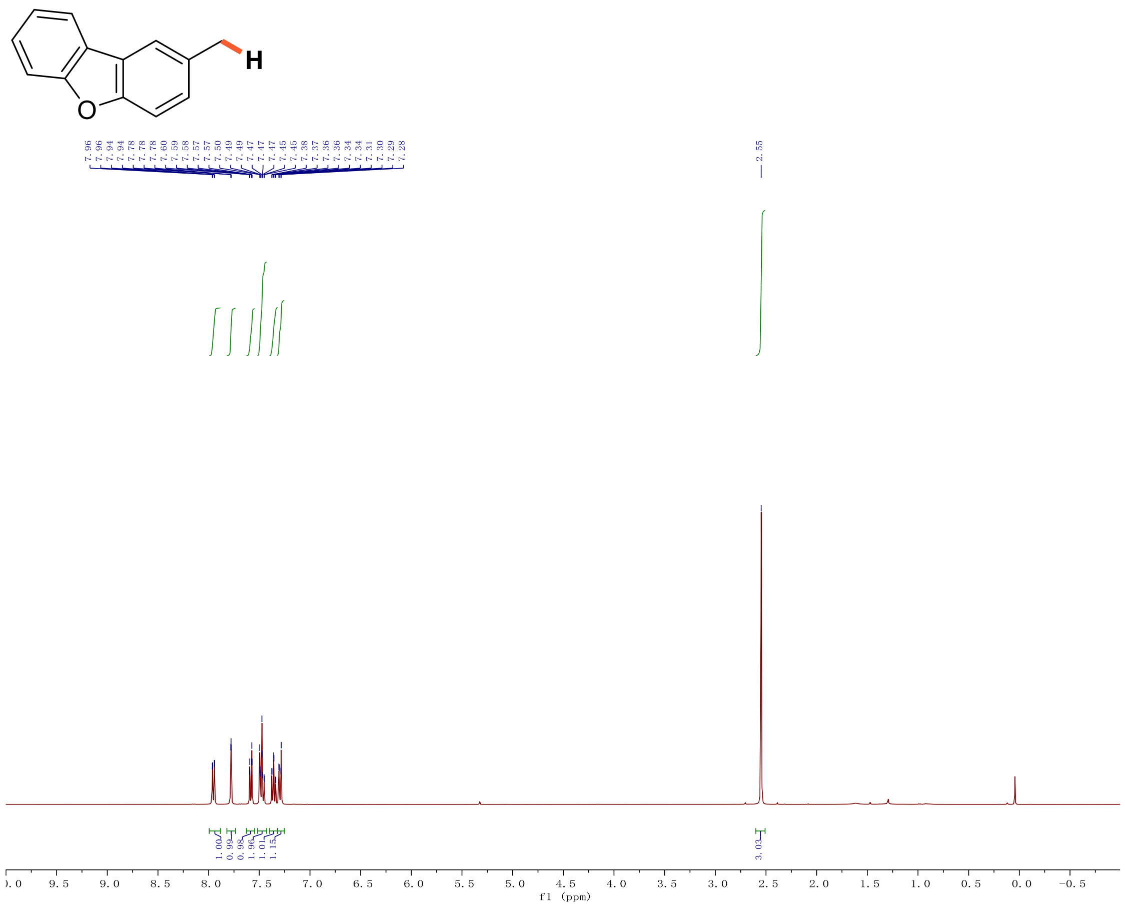

Supplementary Figure 133. ^{1}**H NMR** (400 MHz, Chloroform-*d*) of **2p**

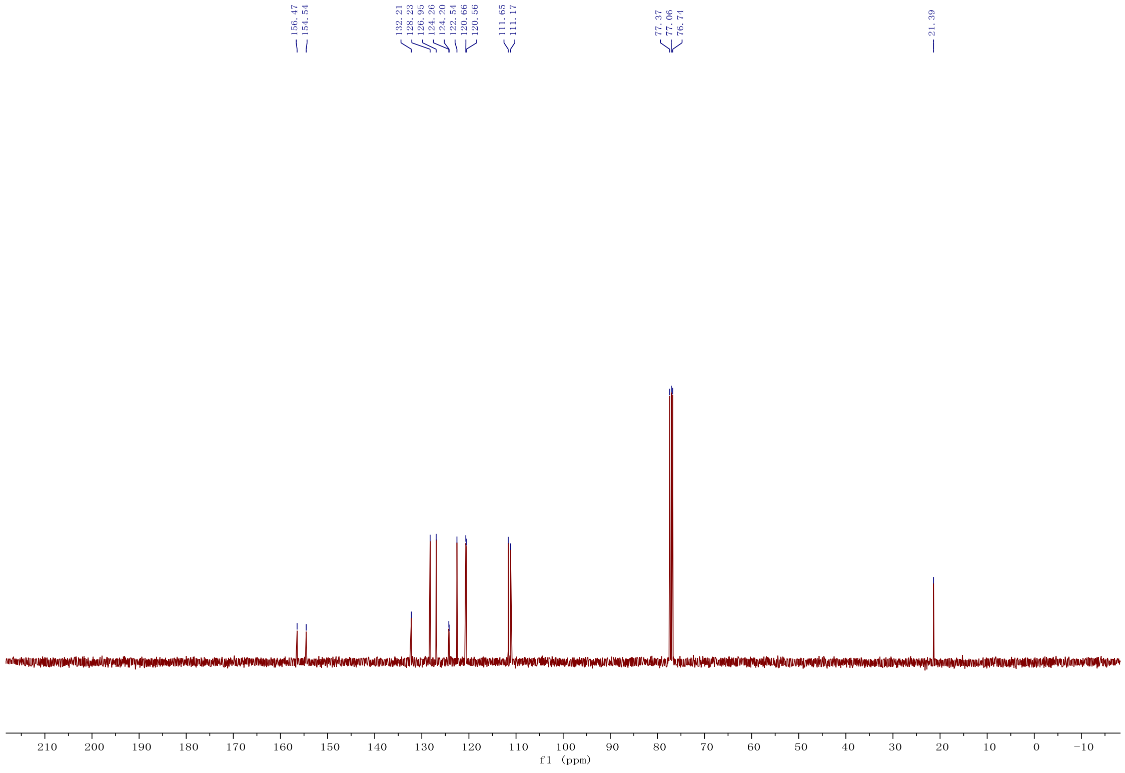

Supplementary Figure 134. ^{13}**C NMR** (101 MHz, Chloroform-*d*) of **2p**

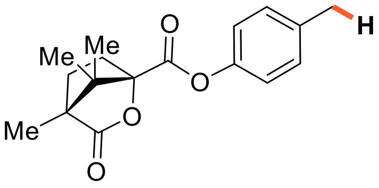

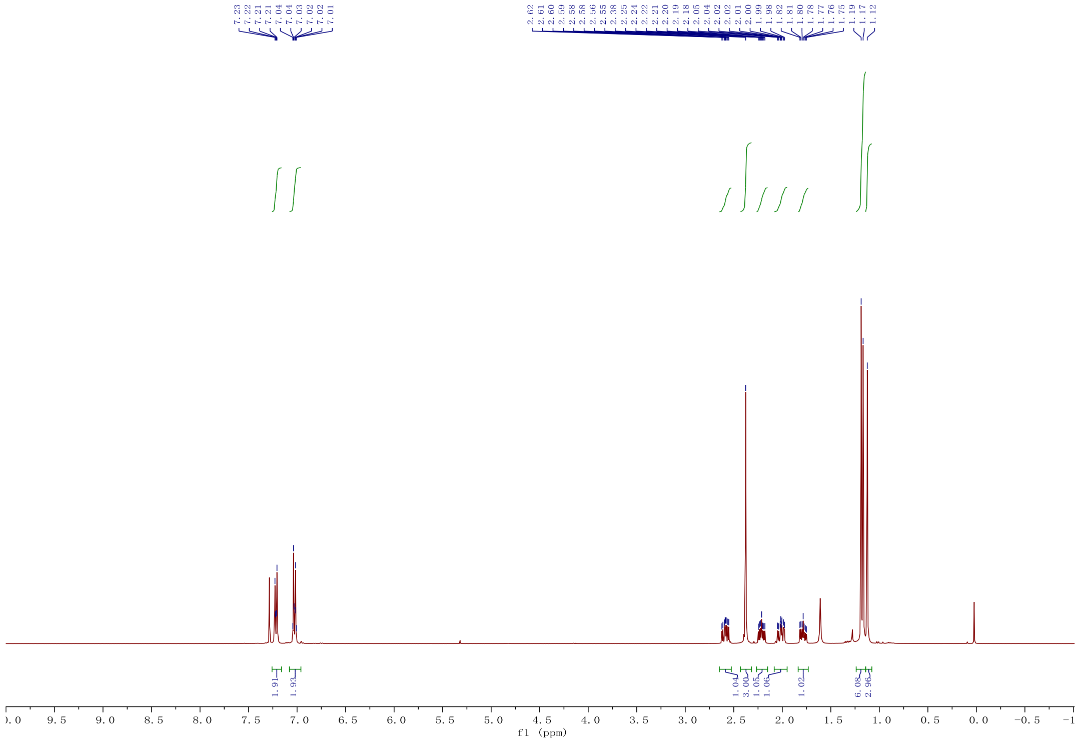

Supplementary Figure 135. ^{13}**C NMR** (101 MHz, Chloroform-*d*) of **2q**

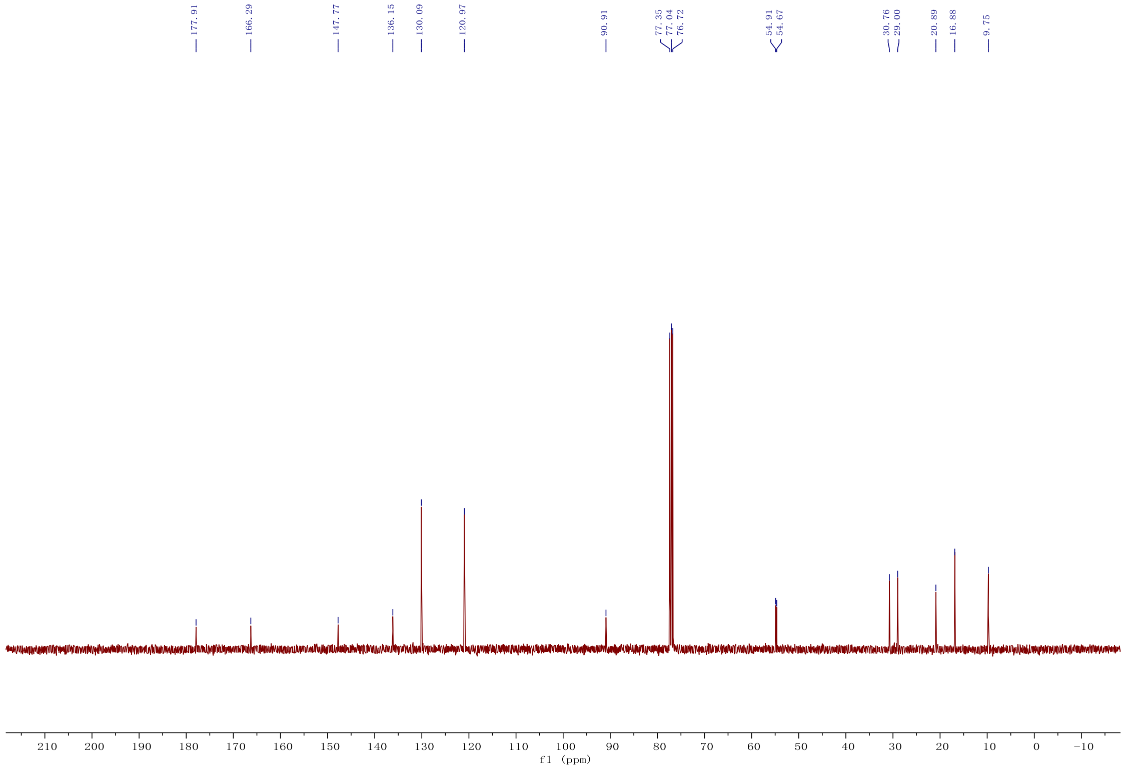

Supplementary Figure 136. ^{13}**C NMR** (101 MHz, Chloroform-*d*) of **2q**

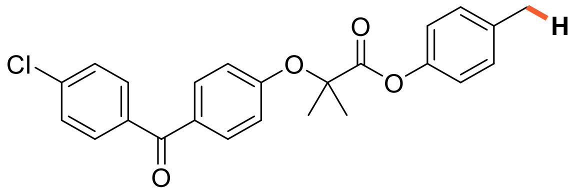

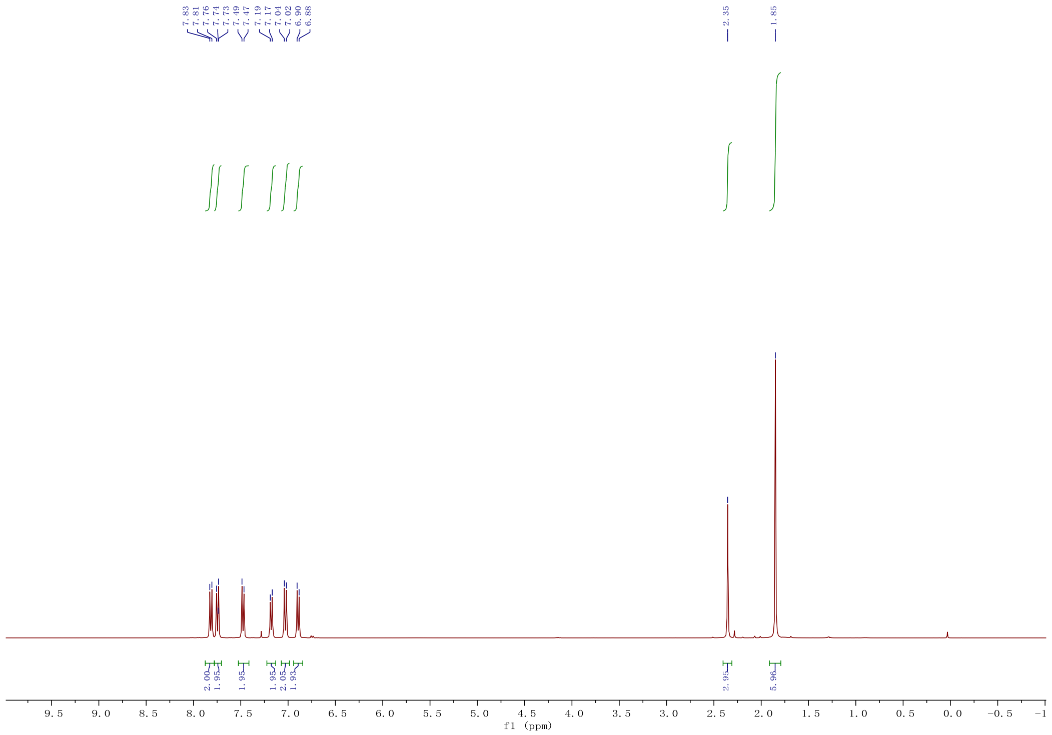

Supplementary Figure 137. ^{1}H NMR (400 MHz, Chloroform-*d*) of **2r**

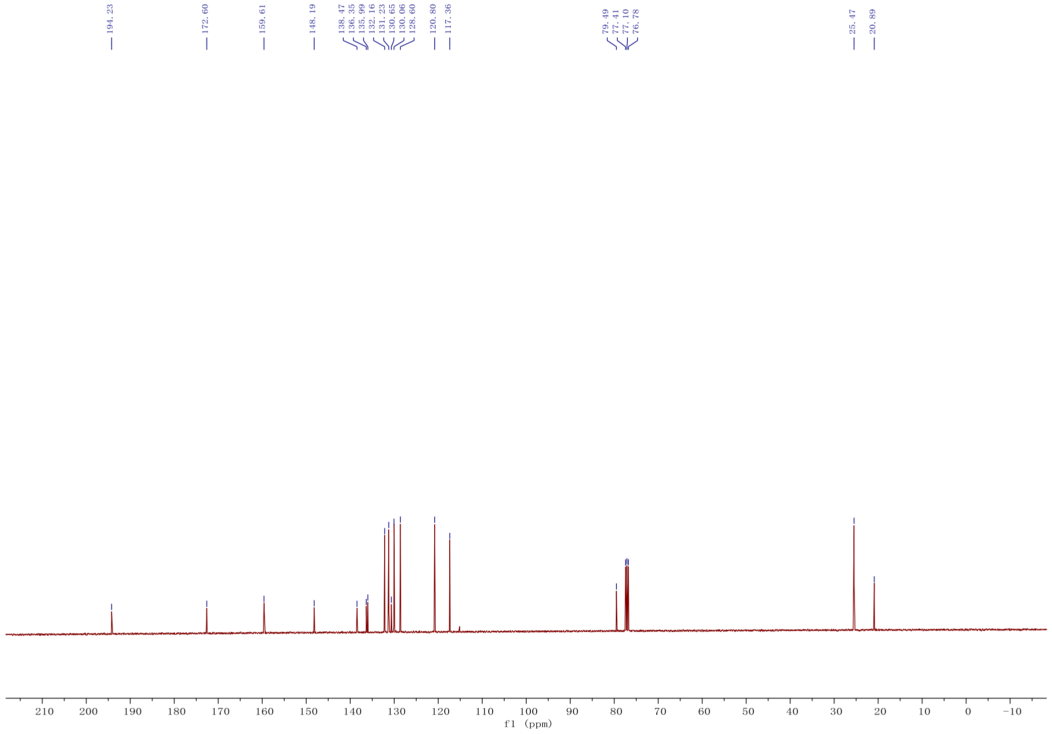

Supplementary Figure 138. ^{13}C NMR (101 MHz, Chloroform-*d*) of **2r**

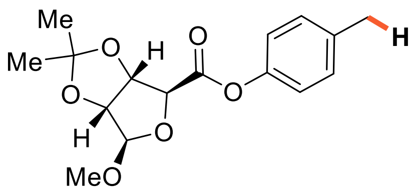

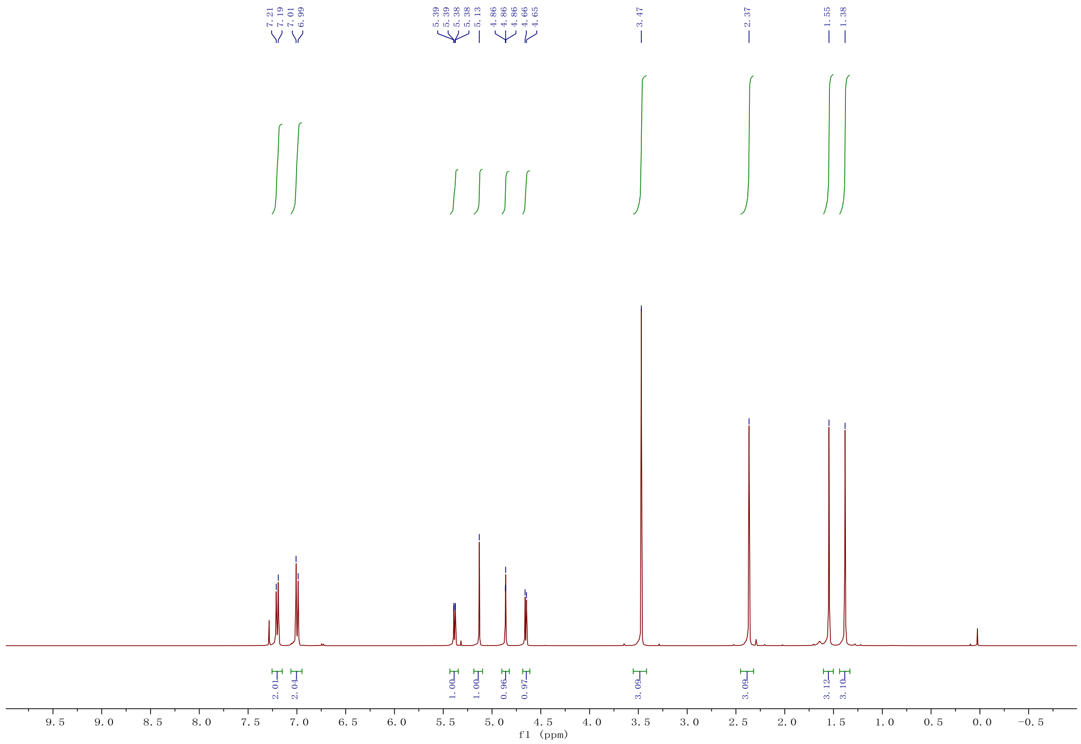

Supplementary Figure 139. ^{1}**H NMR** (400 MHz, Chloroform-*d*) of **2s**

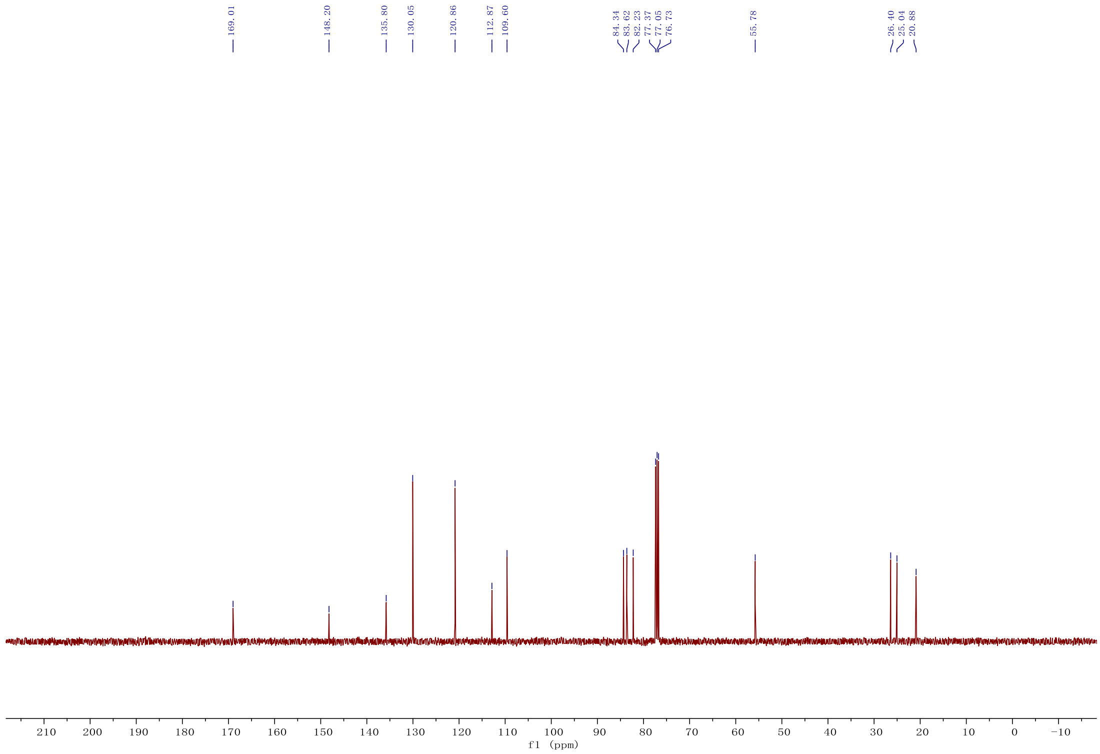

Supplementary Figure 140. ^{13}**C NMR** (101 MHz, Chloroform-*d*) of **2s**

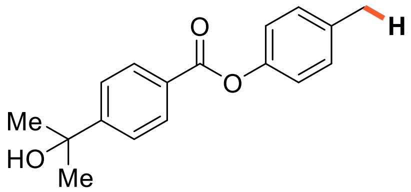

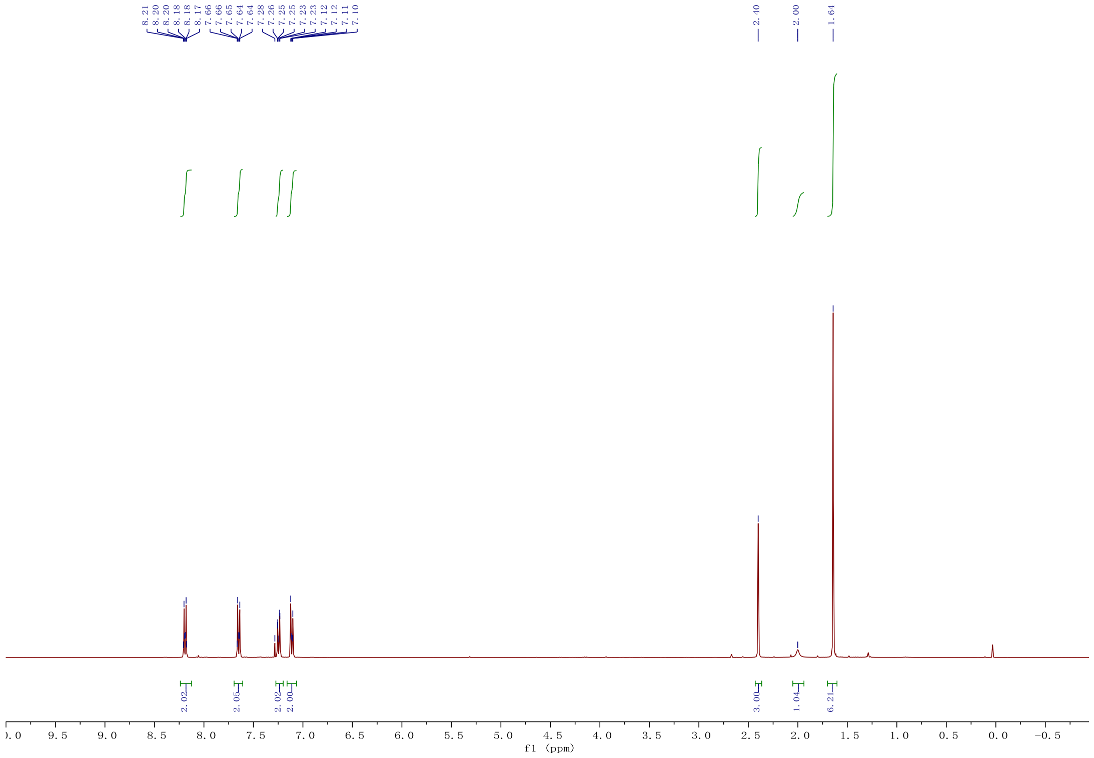

Supplementary Figure 141. **^{1}H NMR** (400 MHz, Chloroform-*d*) of **2t**

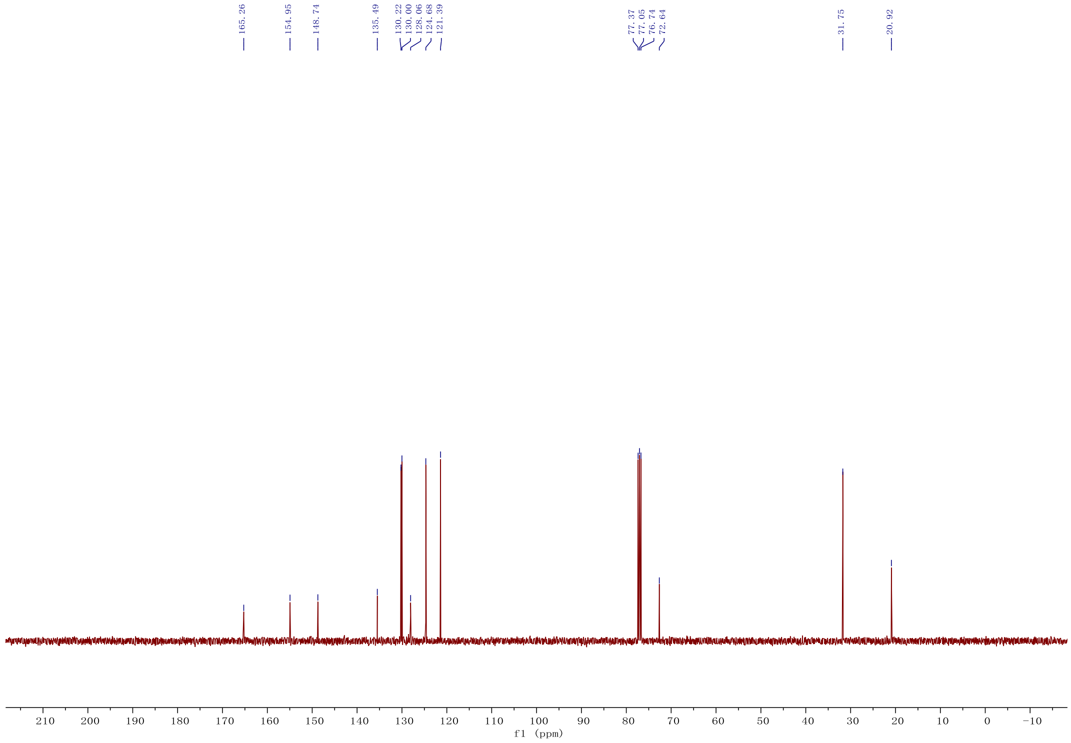

Supplementary Figure 142. **^{13}C NMR** (101 MHz, Chloroform-*d*) of **2t**

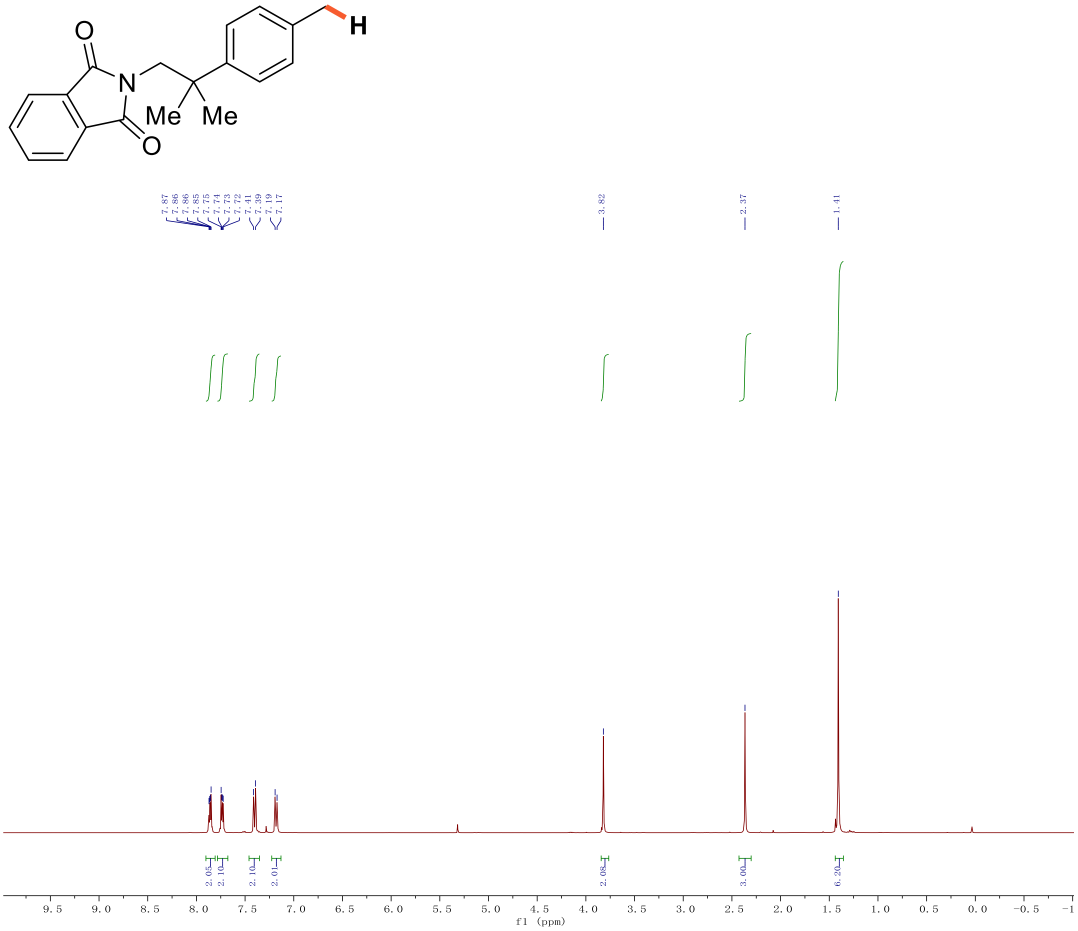

Supplementary Figure 143. ^{1}**H NMR** (400 MHz, Chloroform-*d*) of **2u**

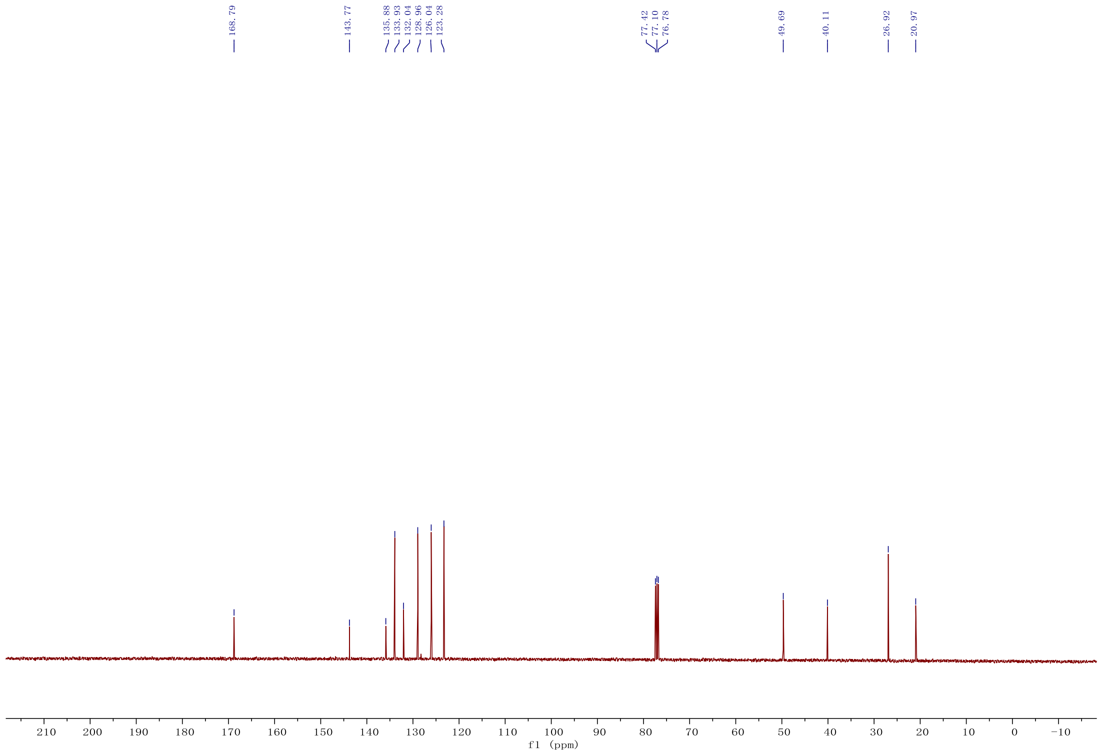

Supplementary Figure 144. ^{13}**C NMR** (101 MHz, Chloroform-*d*) of **2u**

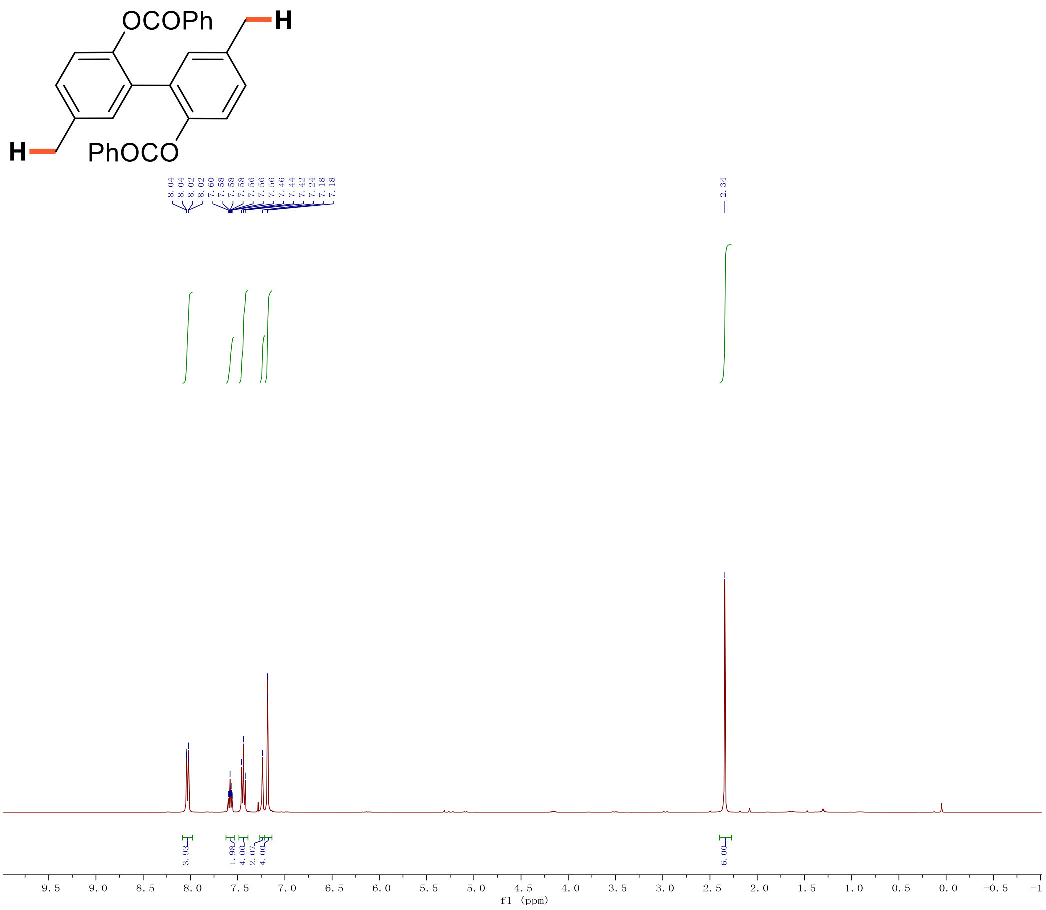

Supplementary Figure 145. ^{1}**H NMR** (400 MHz, Chloroform-*d*) of **2v**

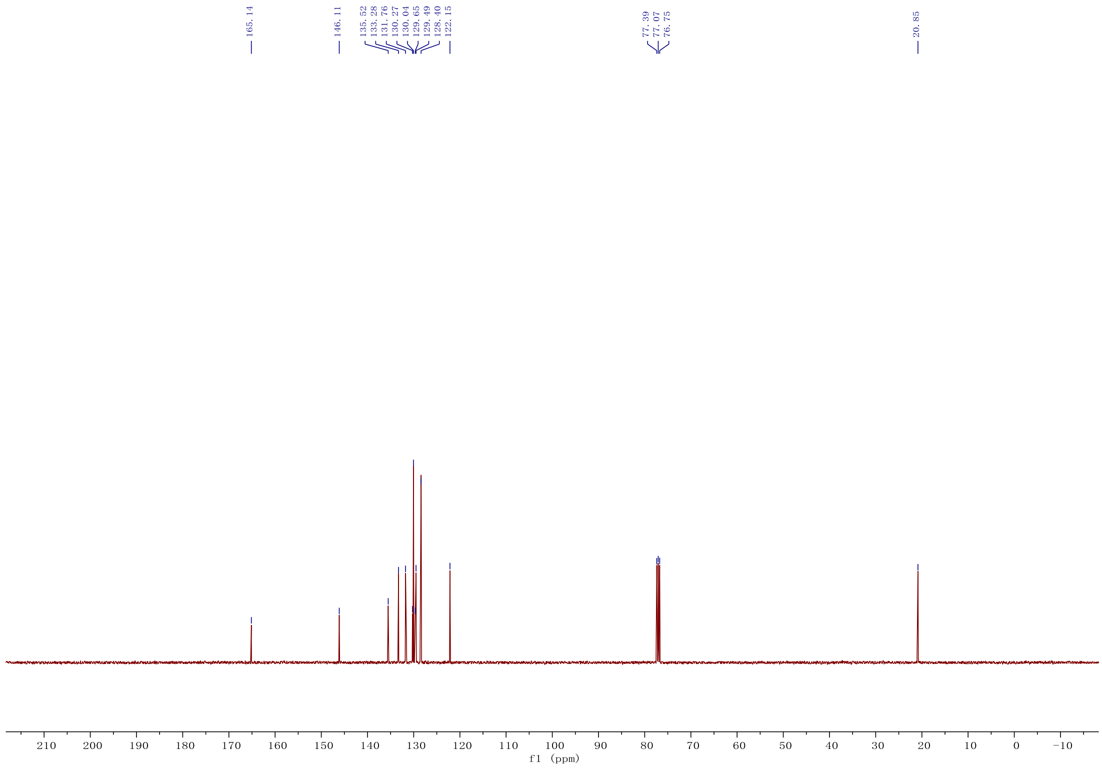

Supplementary Figure 146. ^{13}**C NMR** (101 MHz, Chloroform-*d*) of **2v**

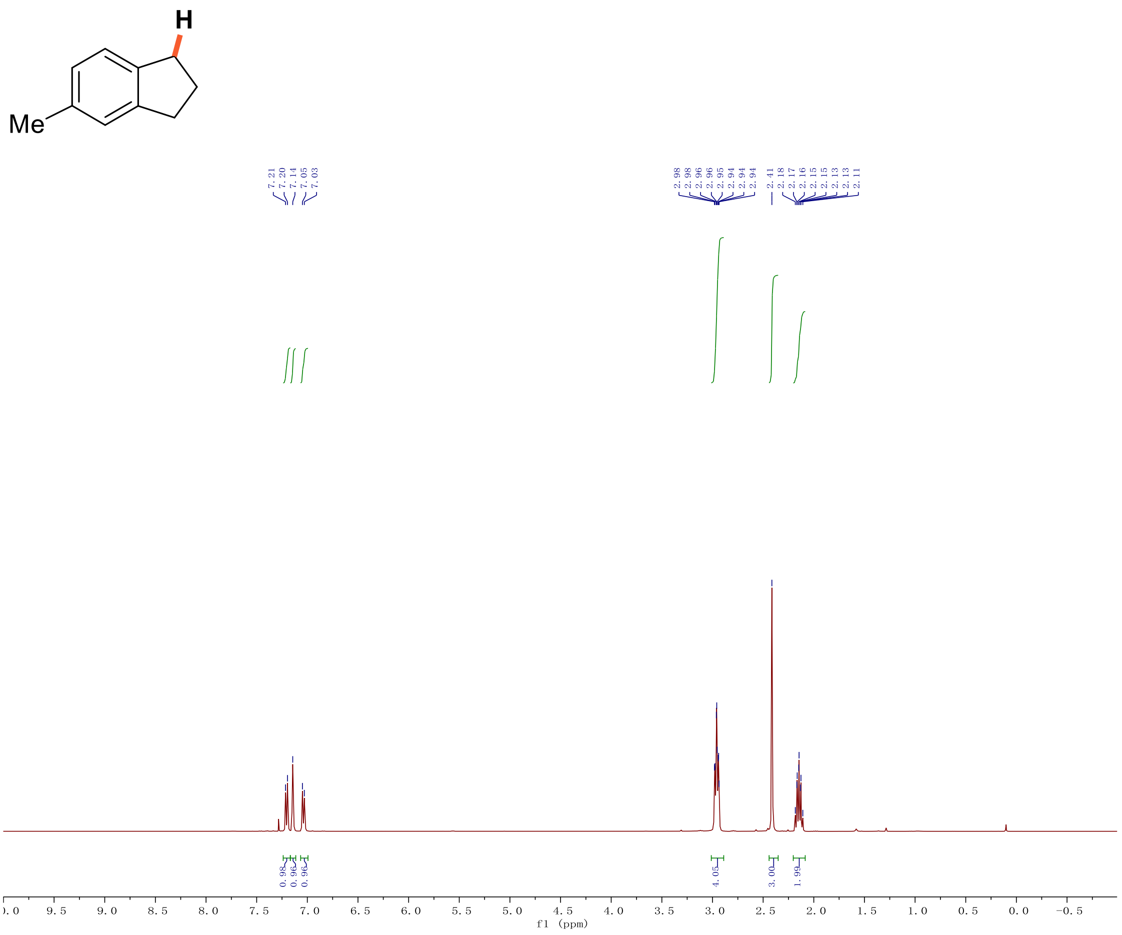

Supplementary Figure 147. ^{1}**H NMR** (400 MHz, Chloroform-*d*) of **2x**

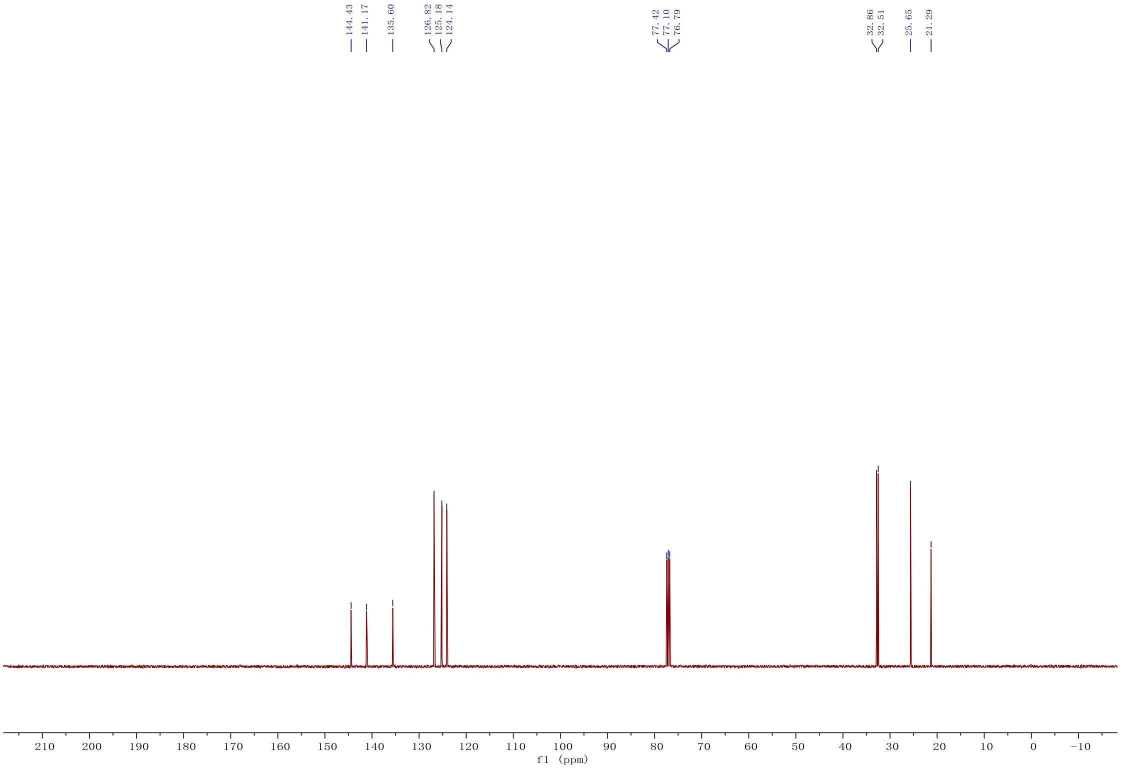

Supplementary Figure 148. ^{13}**C NMR** (101 MHz, Chloroform-*d*) of **2x**

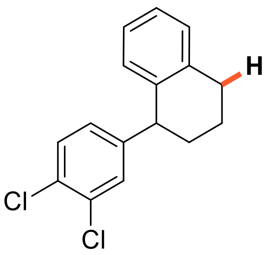

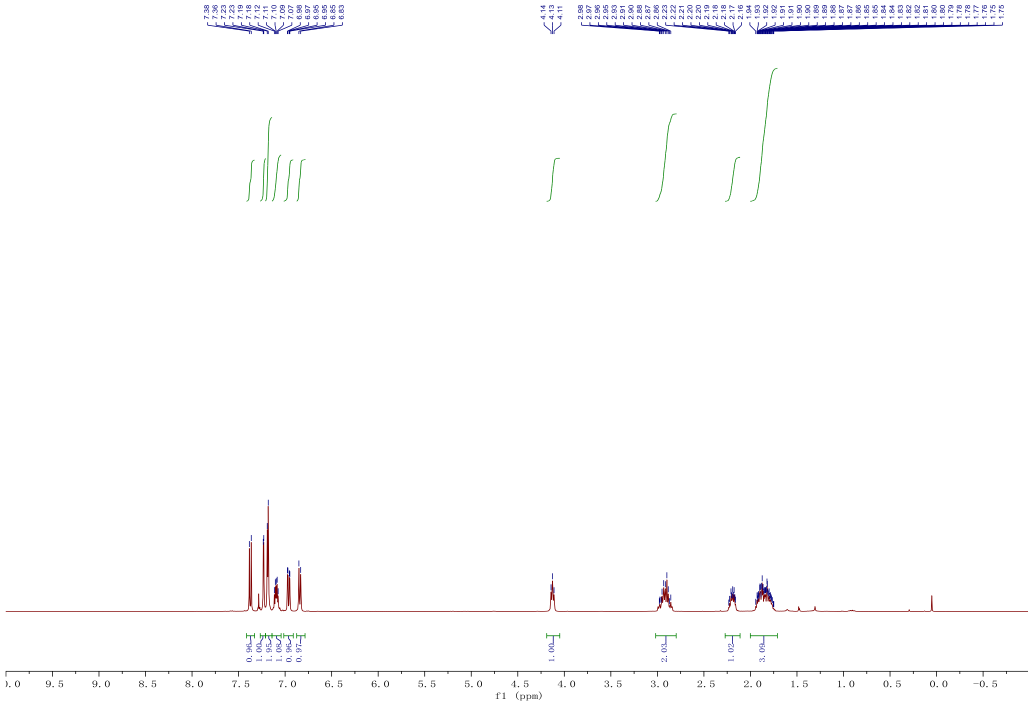

Supplementary Figure 149. ^{1}H NMR (400 MHz, Chloroform-*d*) of **2y**

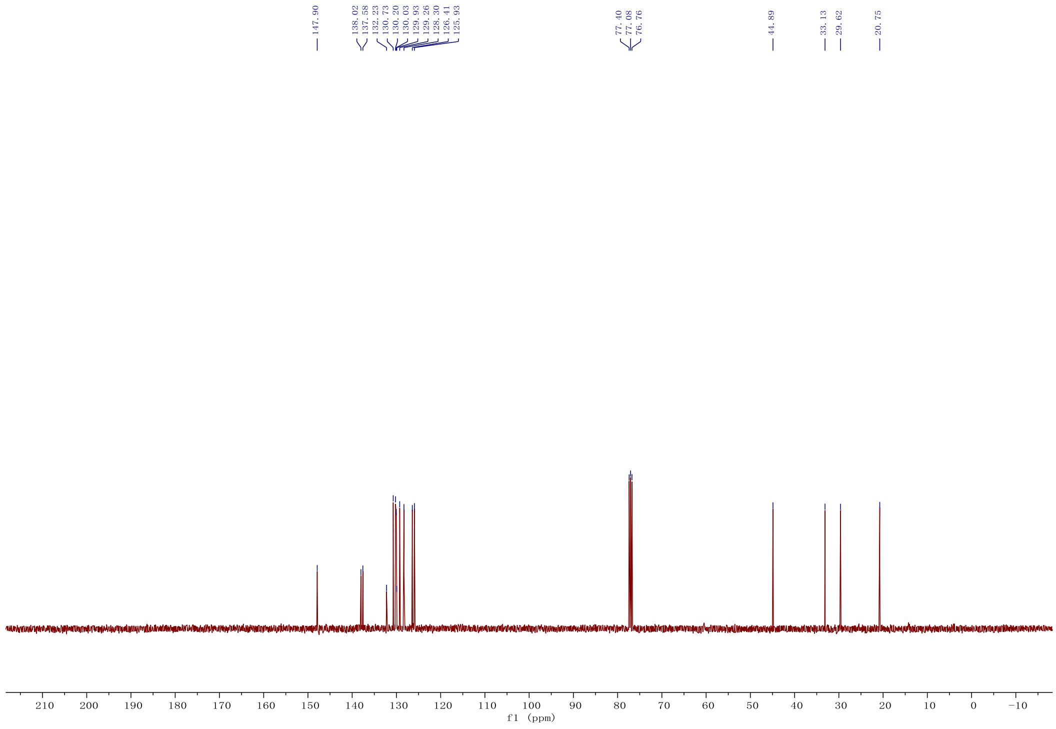

Supplementary Figure 150. ^{13}C NMR (101 MHz, Chloroform-*d*) of **2y**

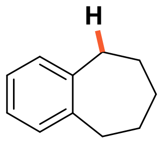

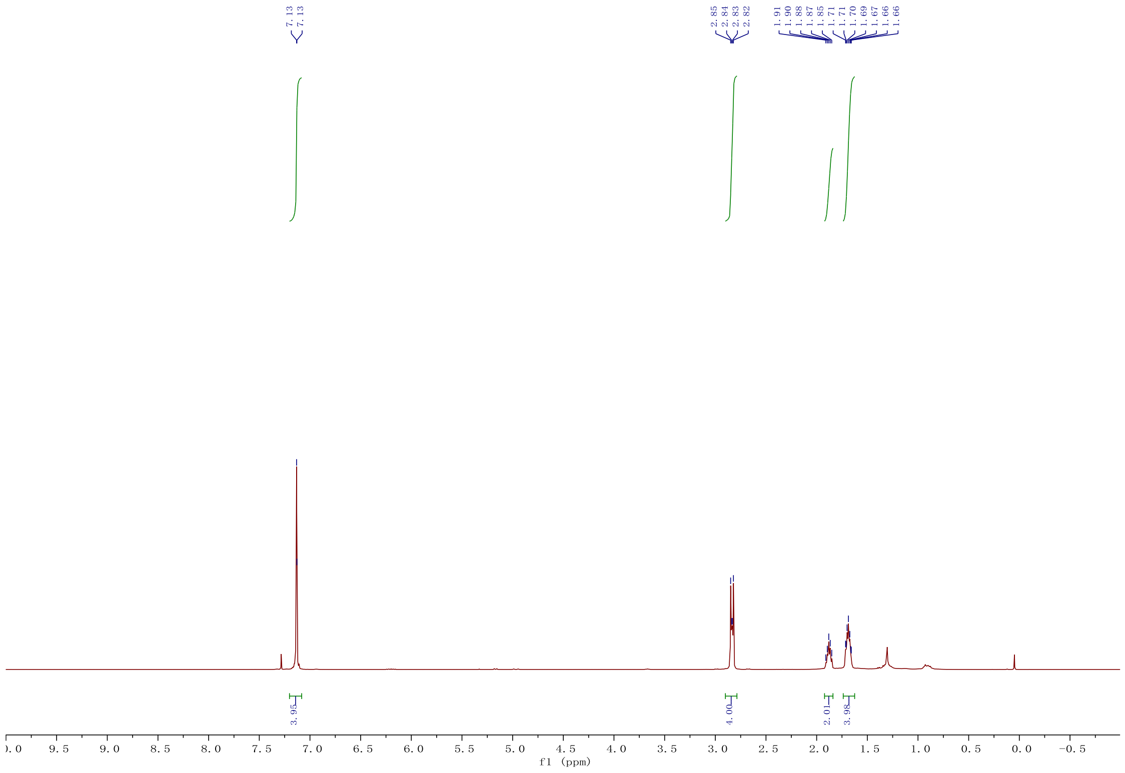

Supplementary Figure 151. ^{1}**H NMR** (400 MHz, Chloroform-*d*) of **2z**

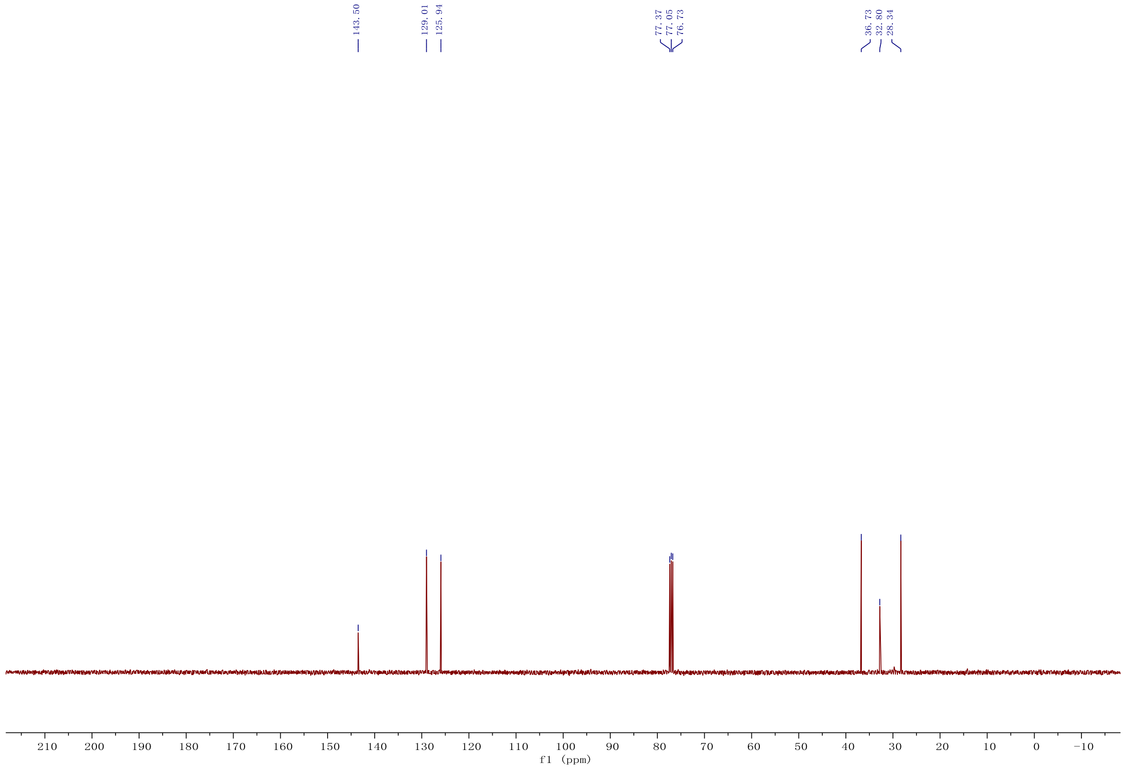

Supplementary Figure 152. ^{13}**C NMR** (101 MHz, Chloroform-*d*) of **2z**

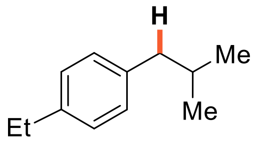

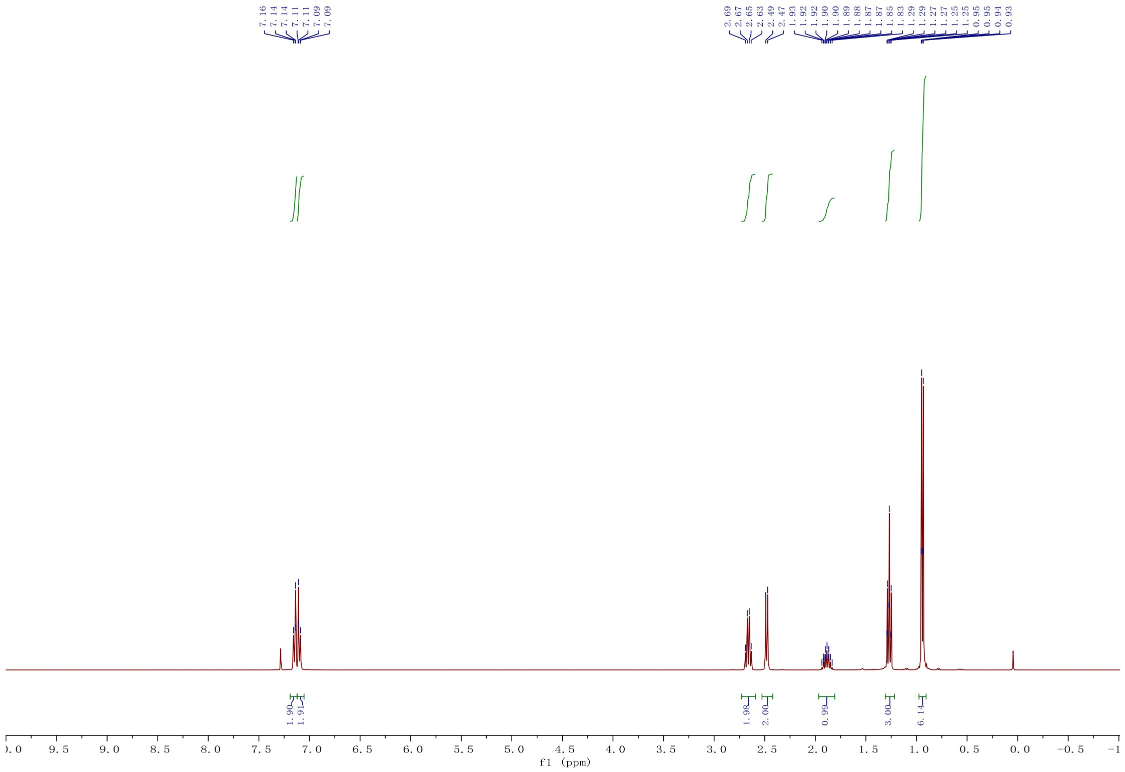

Supplementary Figure 153. ^{1}H NMR (400 MHz, Chloroform-*d*) of **2aa**

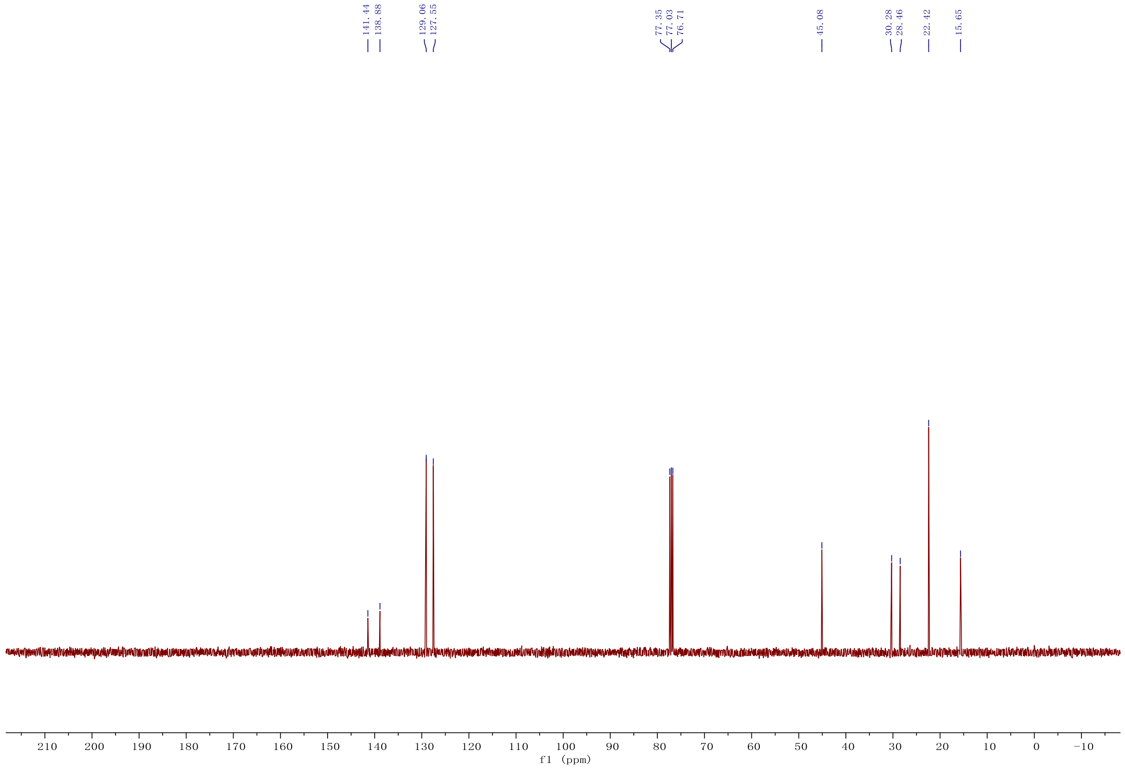

Supplementary Figure 154. ^{13}C NMR (101 MHz, Chloroform-*d*) of **2aa**

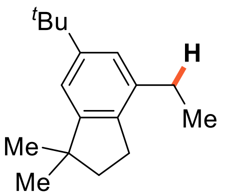

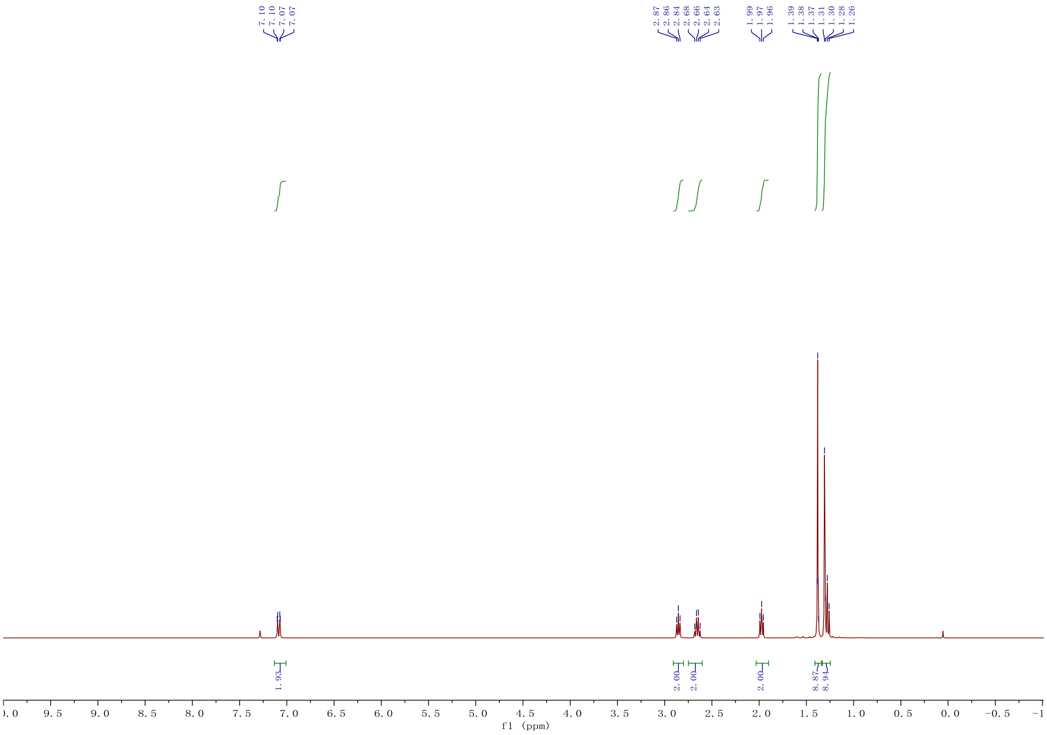

Supplementary Figure 155. ^{1}H NMR (400 MHz, Chloroform-*d*) of **2ab**

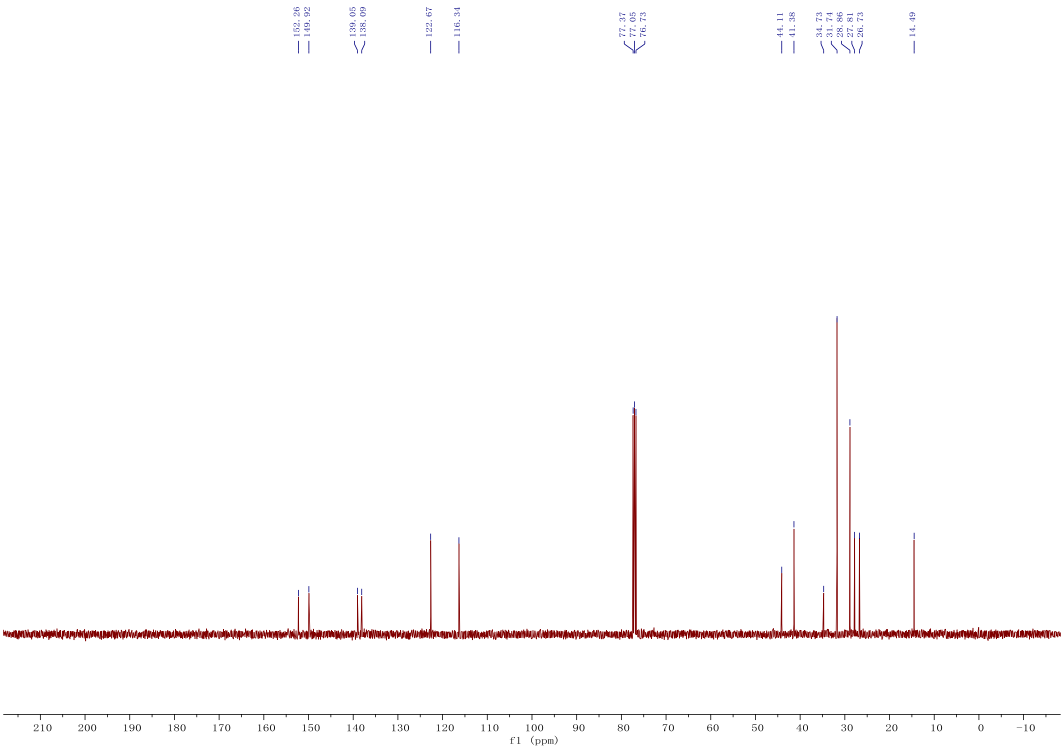

Supplementary Figure 156. ^{13}C NMR (101 MHz, Chloroform-*d*) of **2ab**

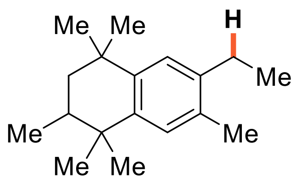

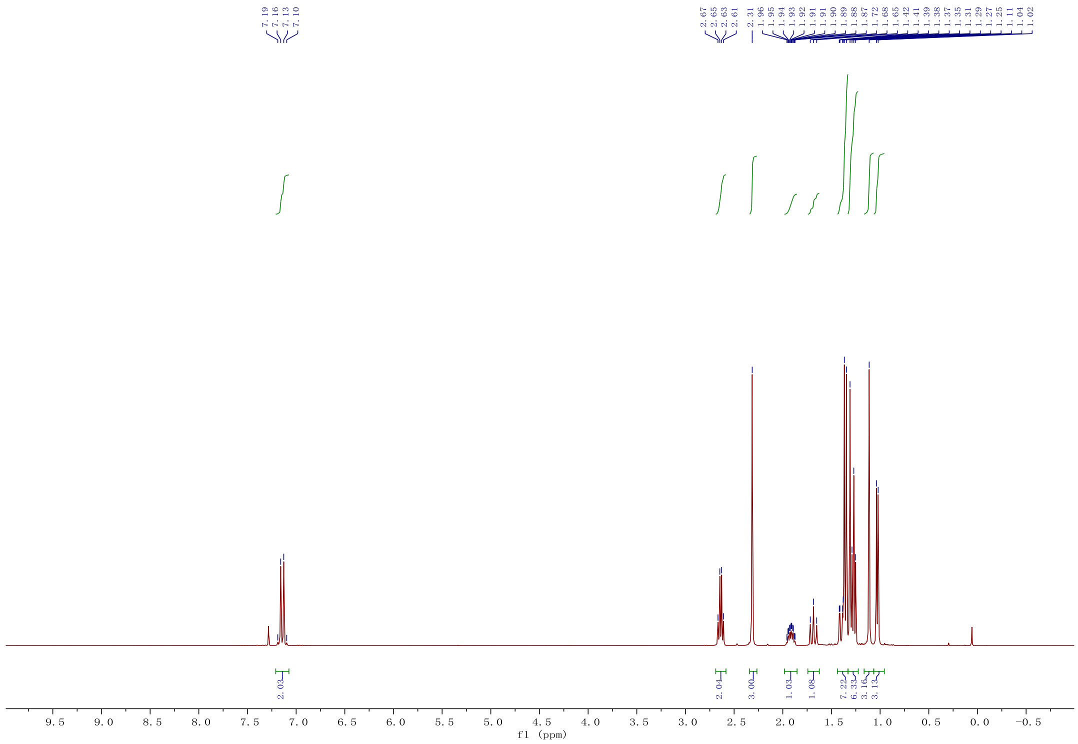

Supplementary Figure 157. ^{1}**H NMR** (400 MHz, Chloroform-*d*) of **2ac**

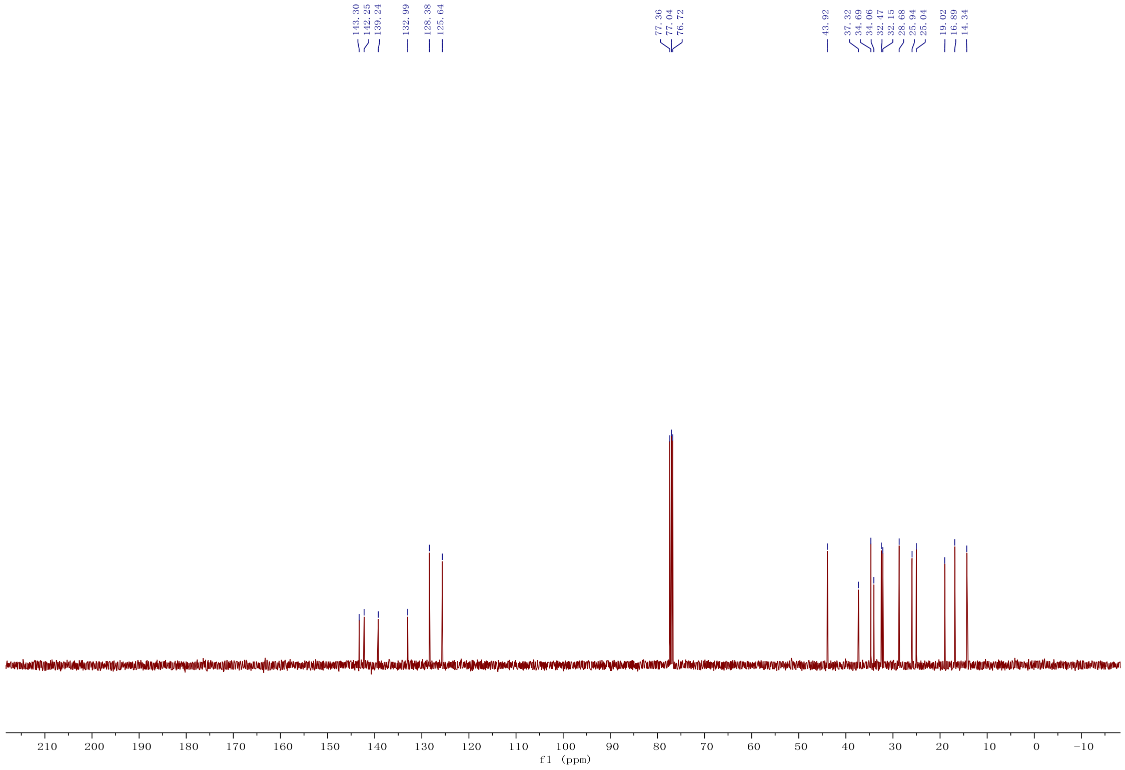

Supplementary Figure 158. ^{13}**C NMR** (101 MHz, Chloroform-*d*) of **2ac**

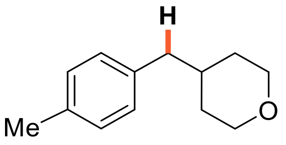

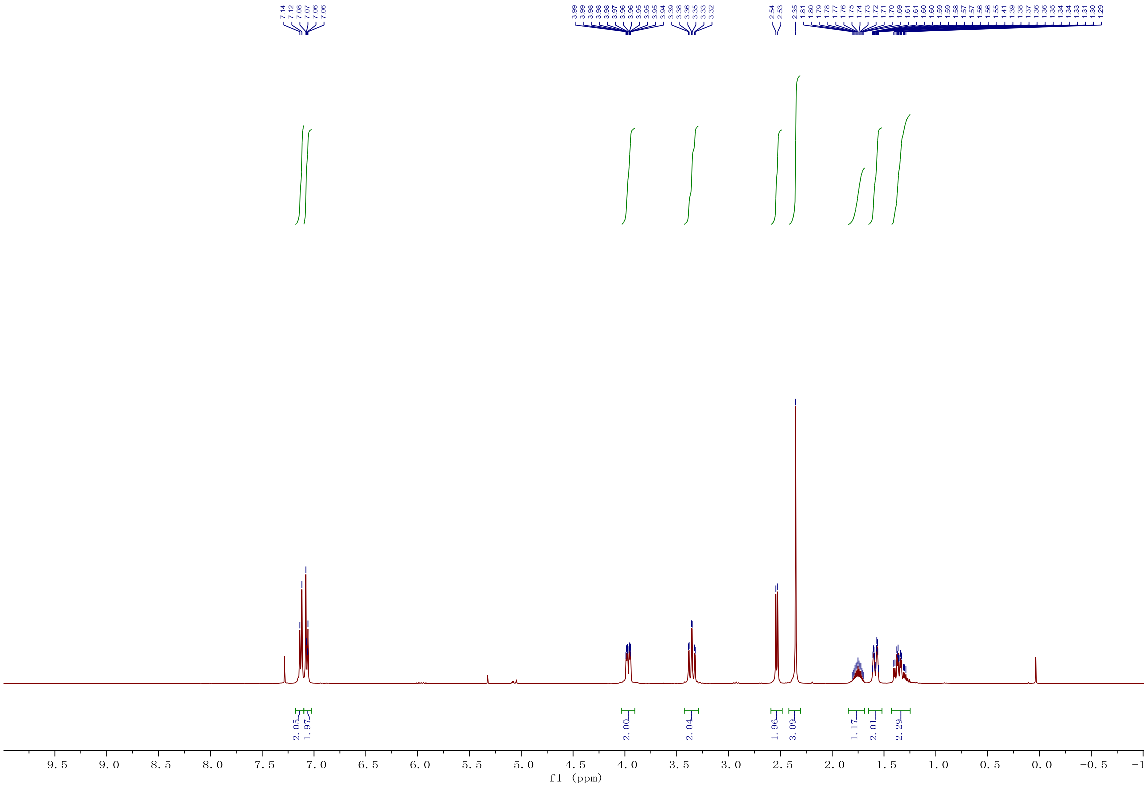

Supplementary Figure 159. ^{1}H NMR (400 MHz, Chloroform-*d*) of **2ad**

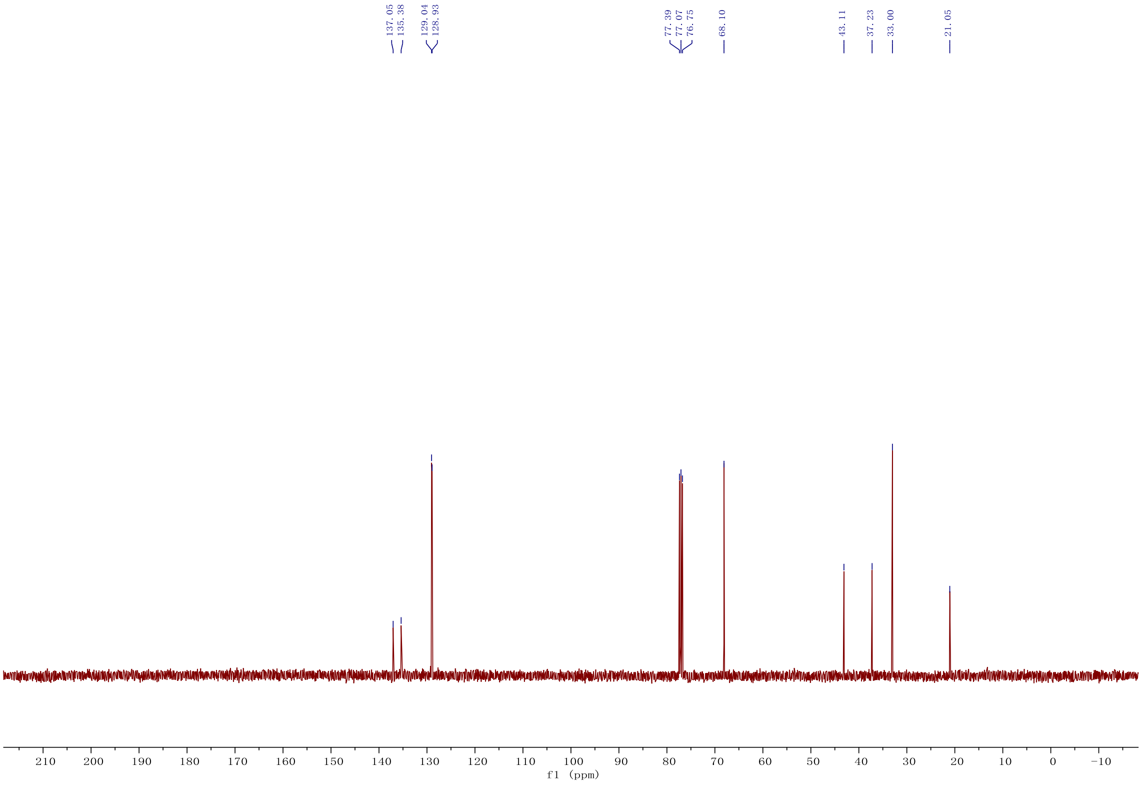

Supplementary Figure 160. ^{13}C NMR (101 MHz, Chloroform-*d*) of **2ad**

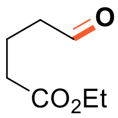

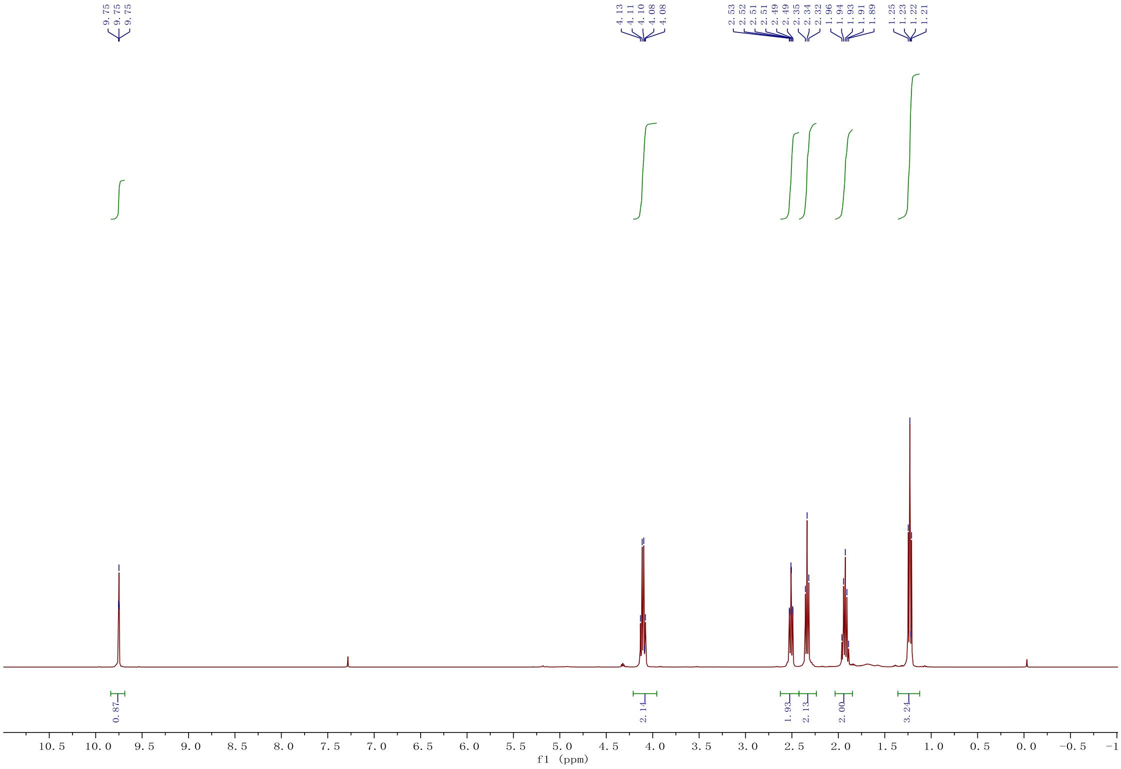

Supplementary Figure 161. ^{1}H NMR (400 MHz, Chloroform-*d*) of **3b**

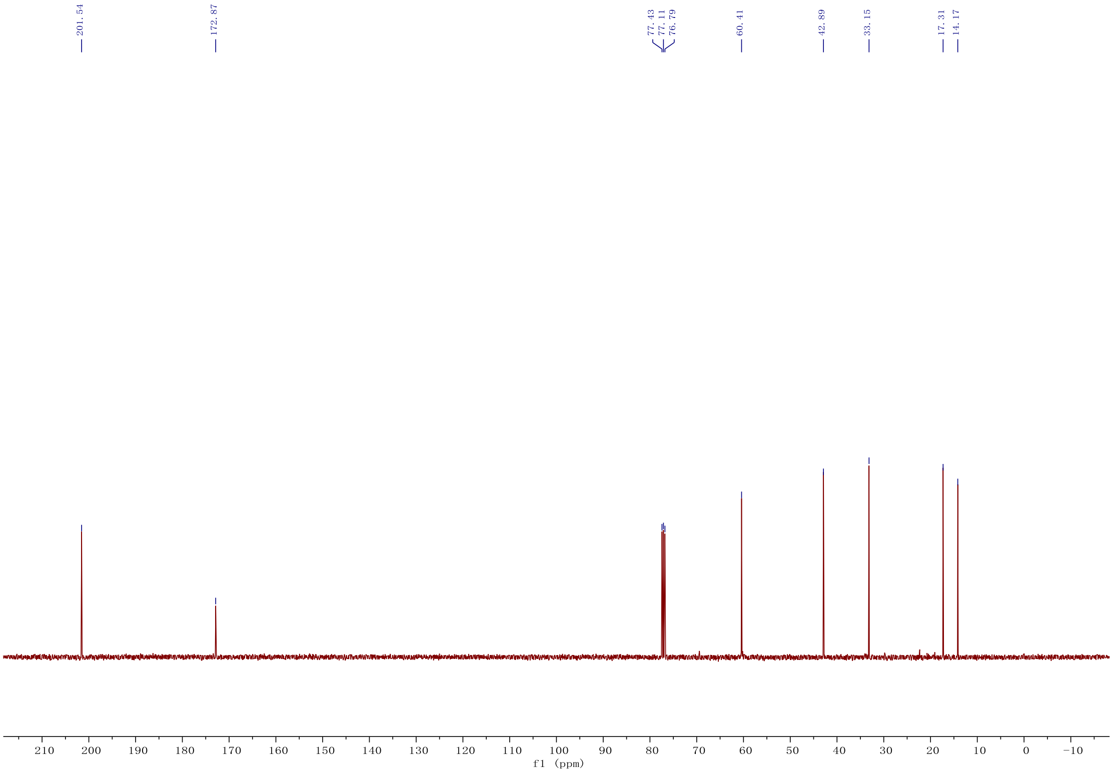

Supplementary Figure 162. ^{13}C NMR (101 MHz, Chloroform-*d*) of **3b**

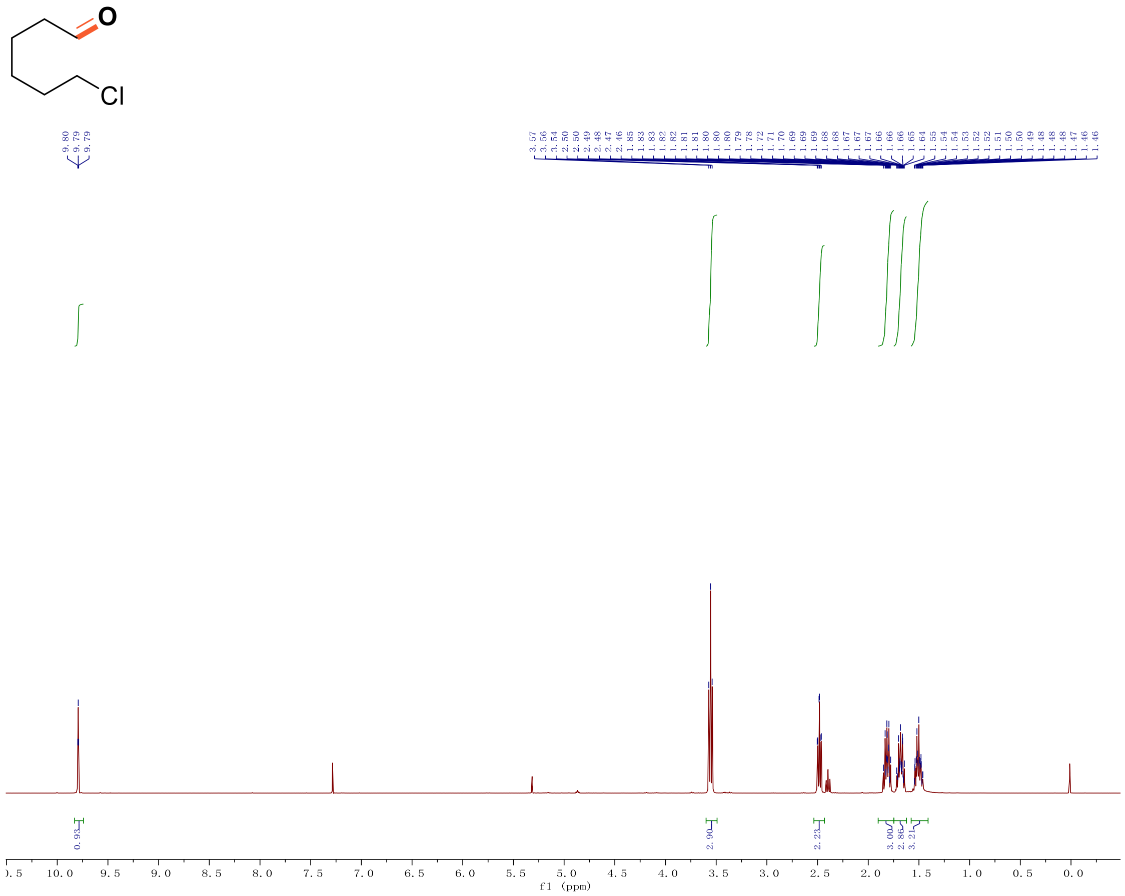

Supplementary Figure 163. ^{1}H NMR (400 MHz, Chloroform-*d*) of **3c**

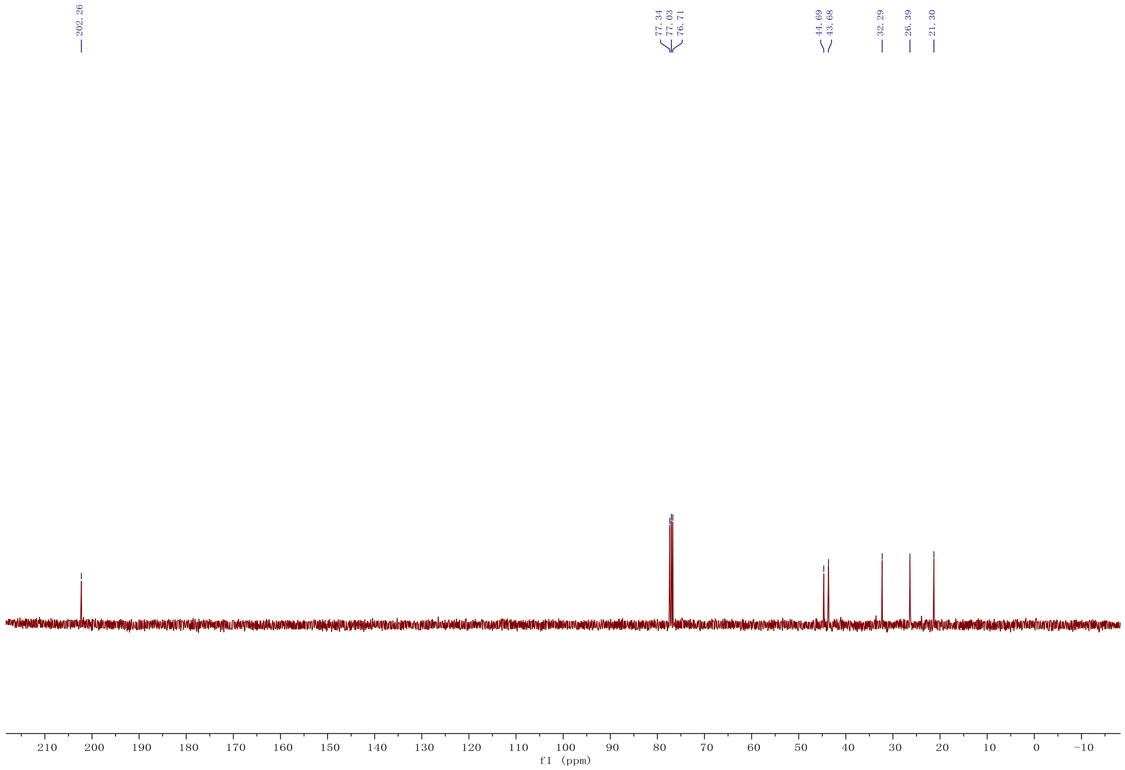

Supplementary Figure 164. ^{13}C NMR (101 MHz, Chloroform-*d*) of **3c**

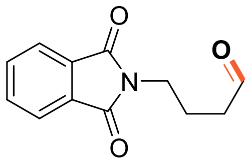

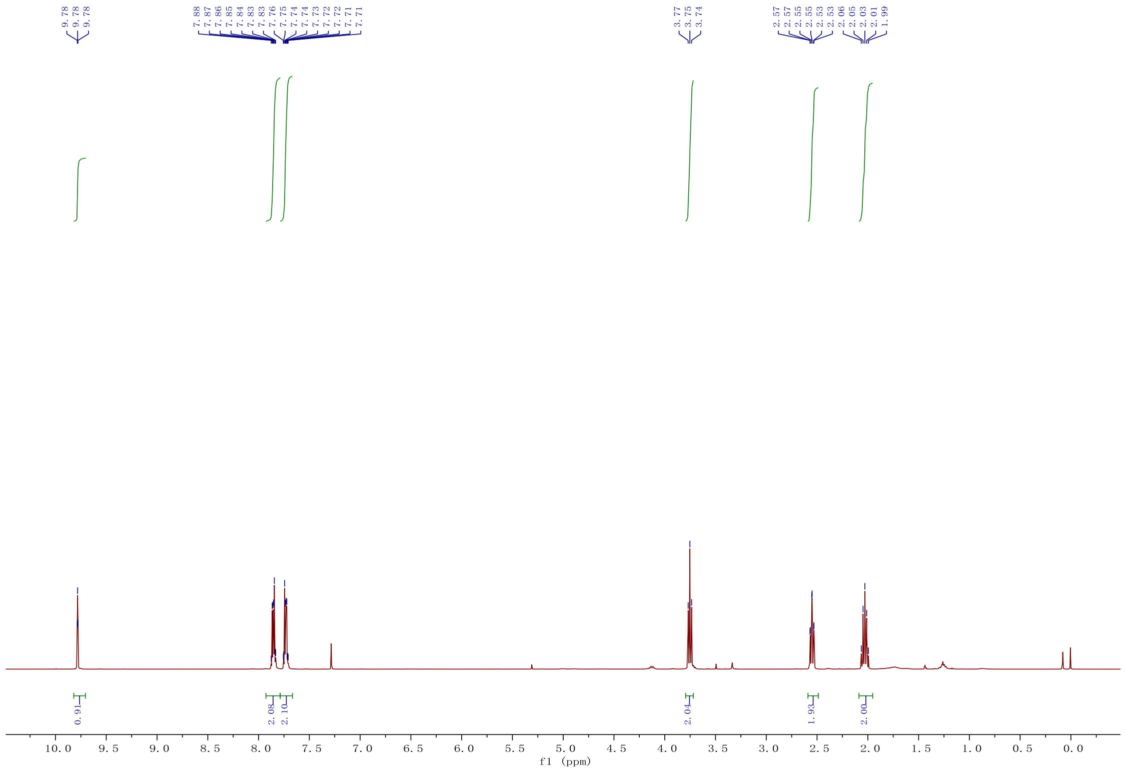

Supplementary Figure 165. **^{1}H NMR** (400 MHz, Chloroform-*d*) of **3d**

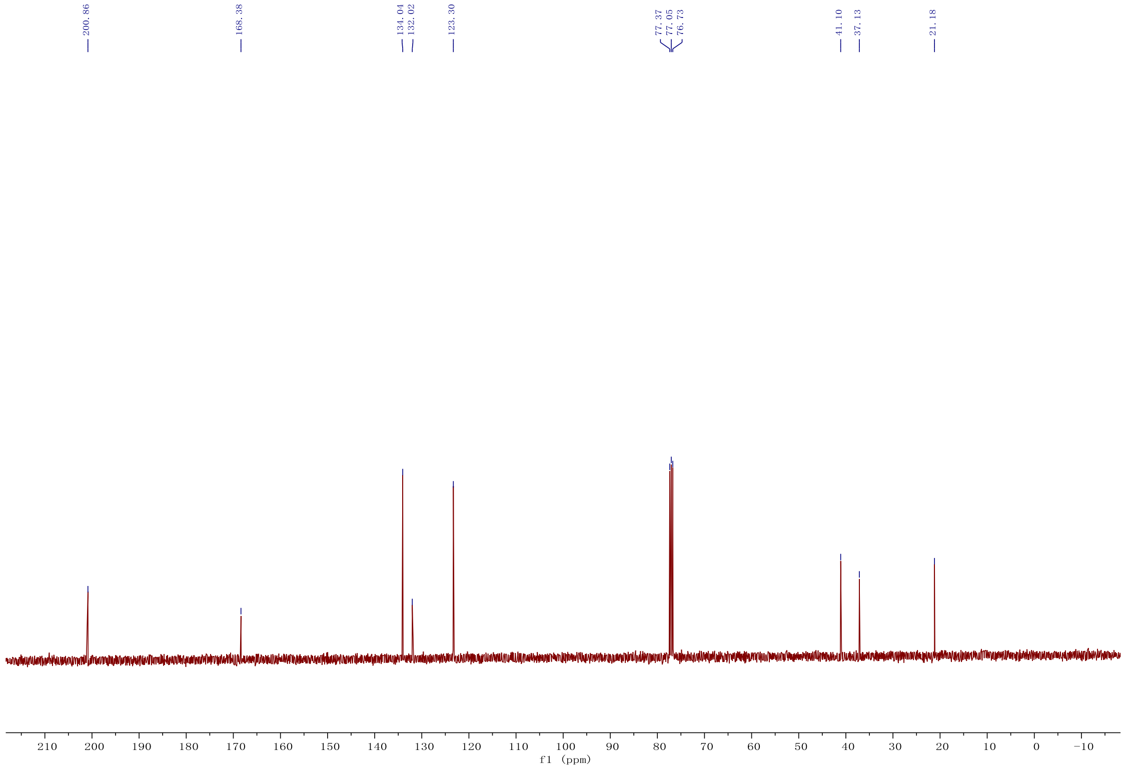

Supplementary Figure 166. **^{13}C NMR** (101 MHz, Chloroform-*d*) of **3d**

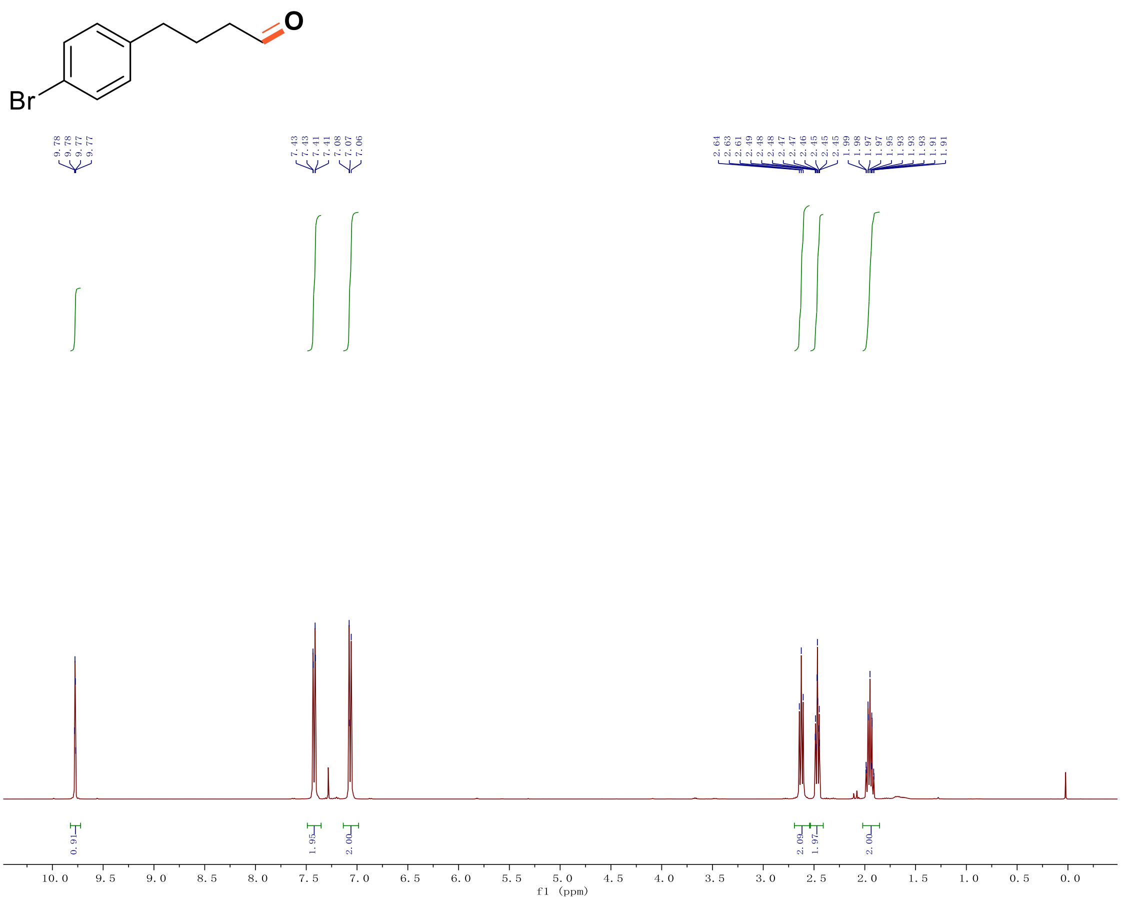

Supplementary Figure 167. ^{1}H NMR (400 MHz, Chloroform-*d*) of **3e**

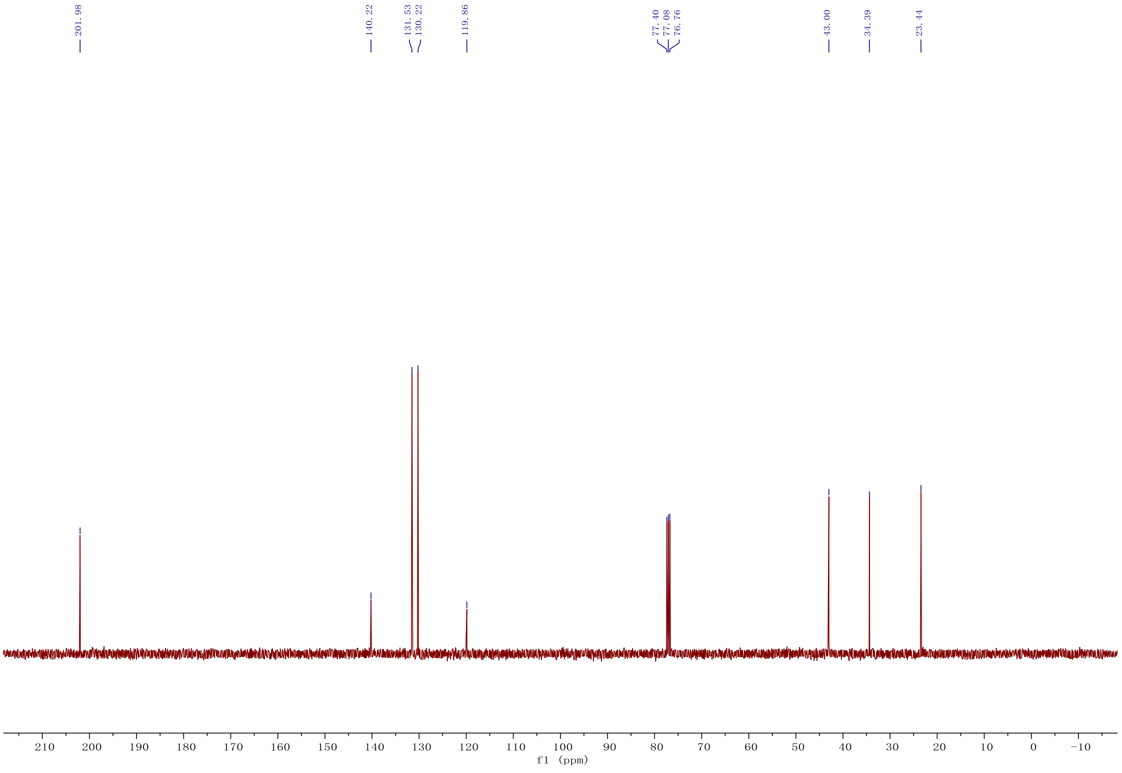

Supplementary Figure 168. ^{13}C NMR (101 MHz, Chloroform-*d*) of **3e**

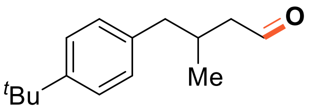

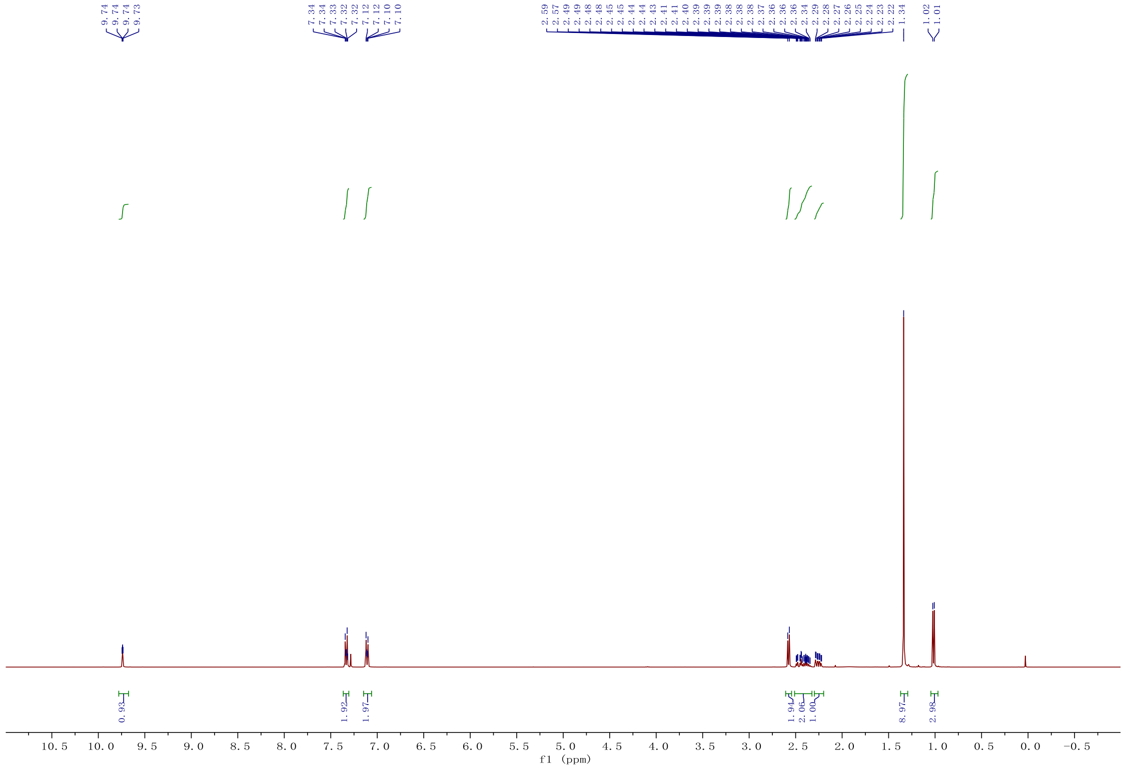

Supplementary Figure 169. ^{1}**H NMR** (400 MHz, Chloroform-*d*) of **3f**

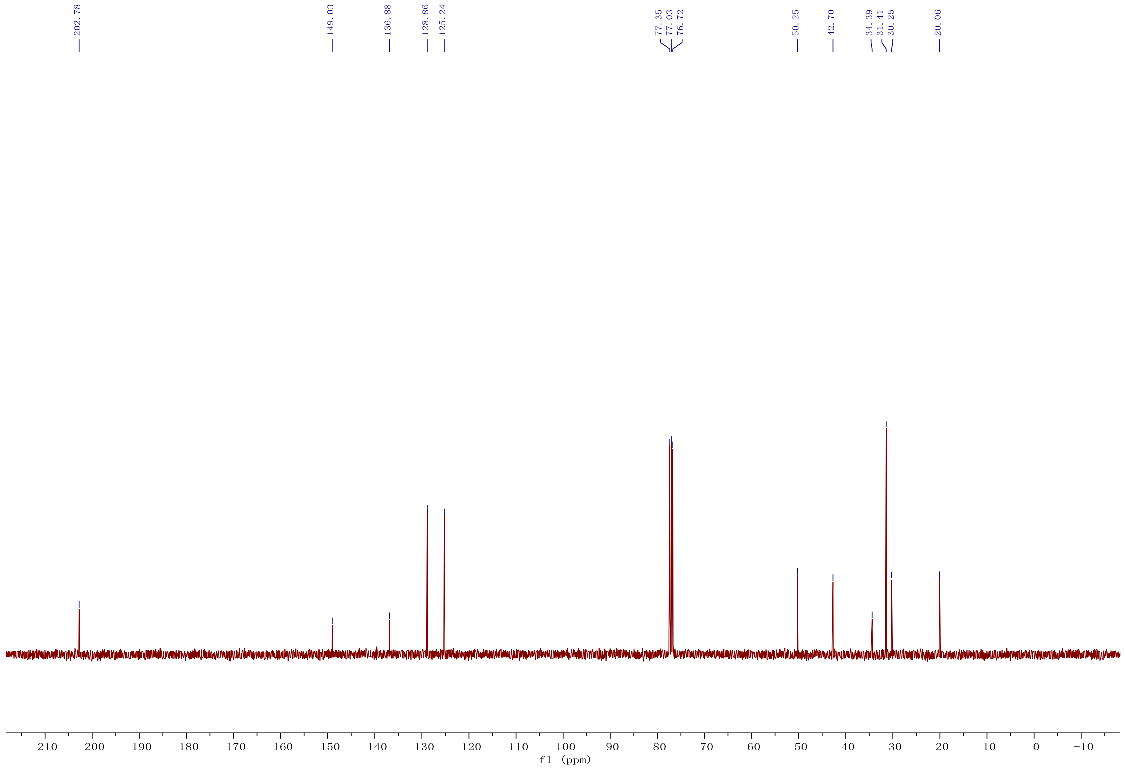

Supplementary Figure 170. ^{13}**C NMR** (101 MHz, Chloroform-*d*) of **3f**

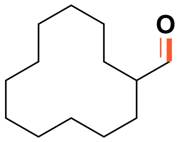

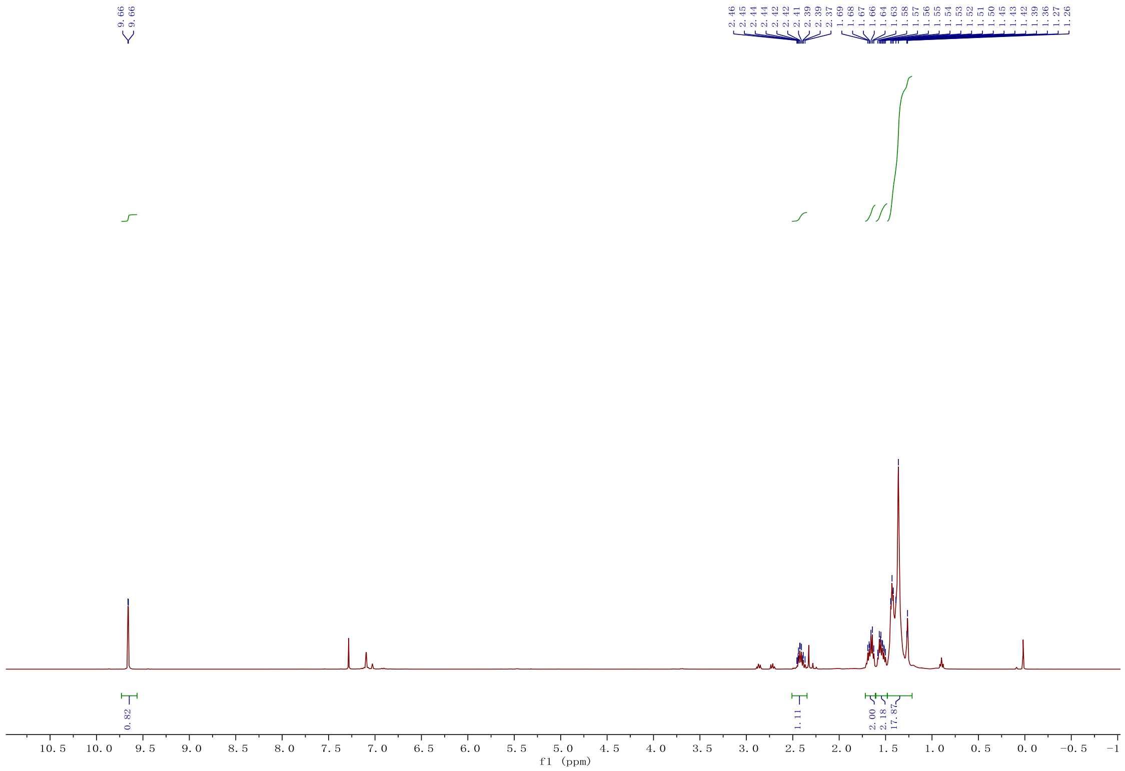

Supplementary Figure 171. ^{1}H NMR (400 MHz, Chloroform-*d*) of **3h**

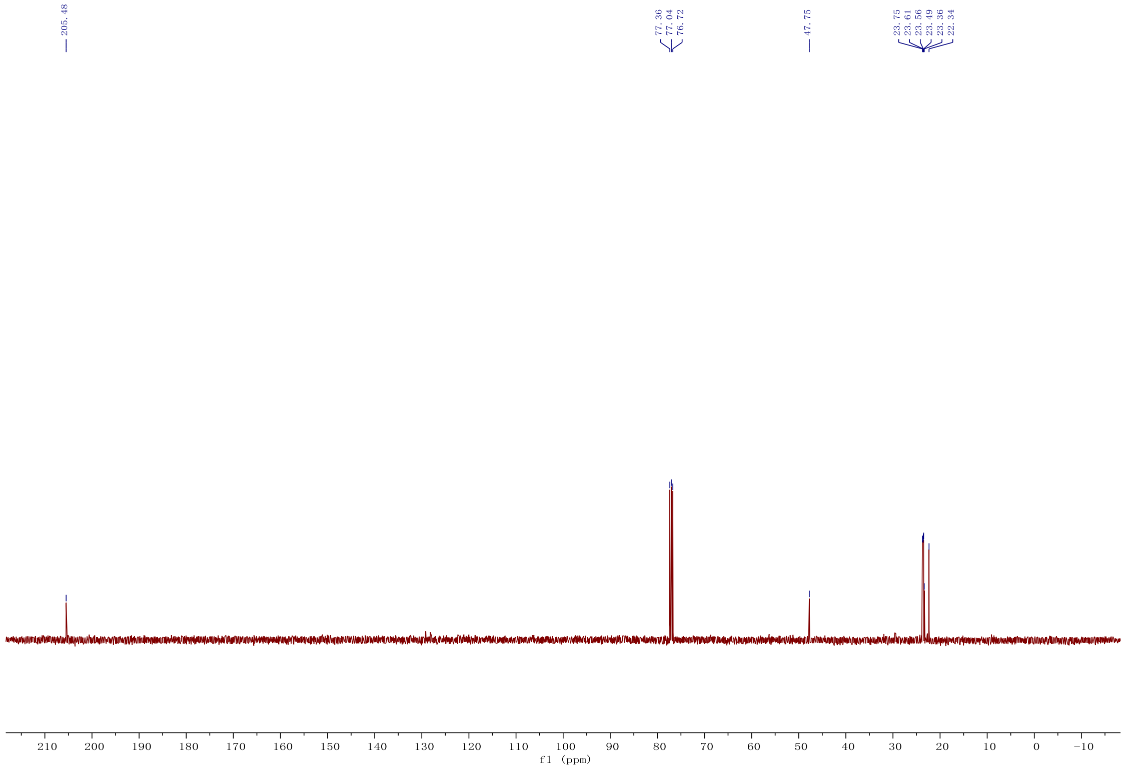

Supplementary Figure 172. ^{13}C NMR (101 MHz, Chloroform-*d*) of **3h**

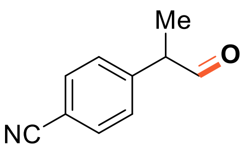

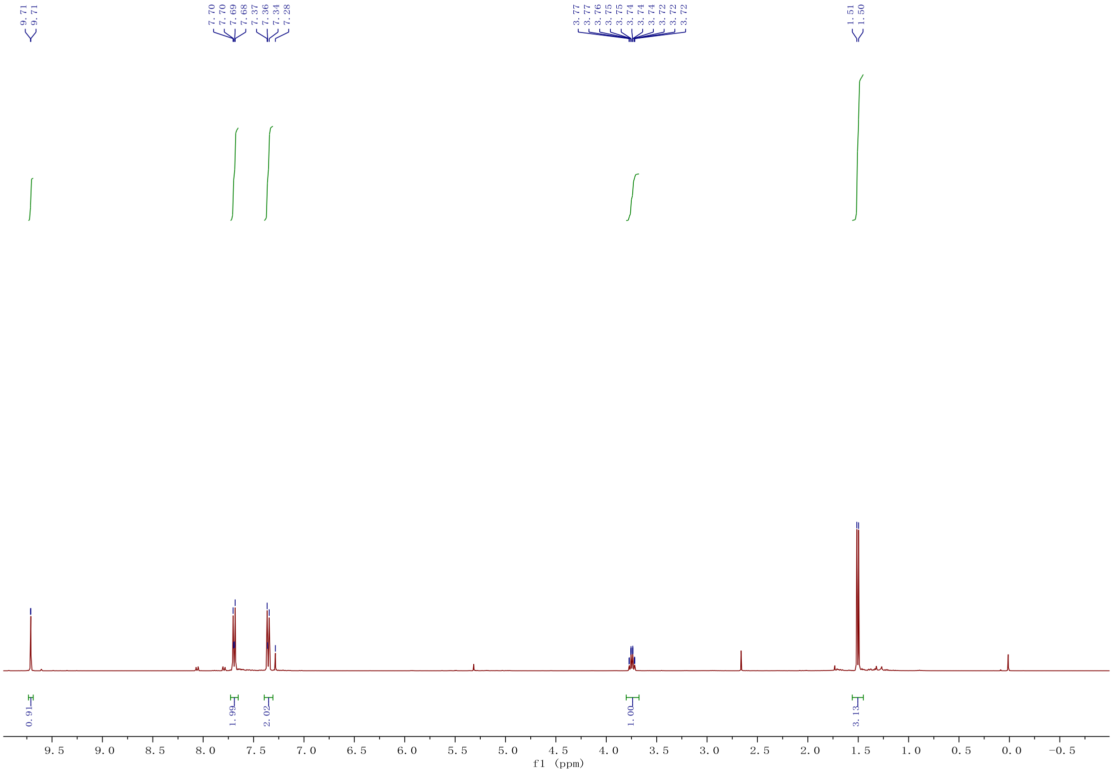

Supplementary Figure 173. ^{1}**H NMR** (400 MHz, Chloroform-*d*) of **3i**

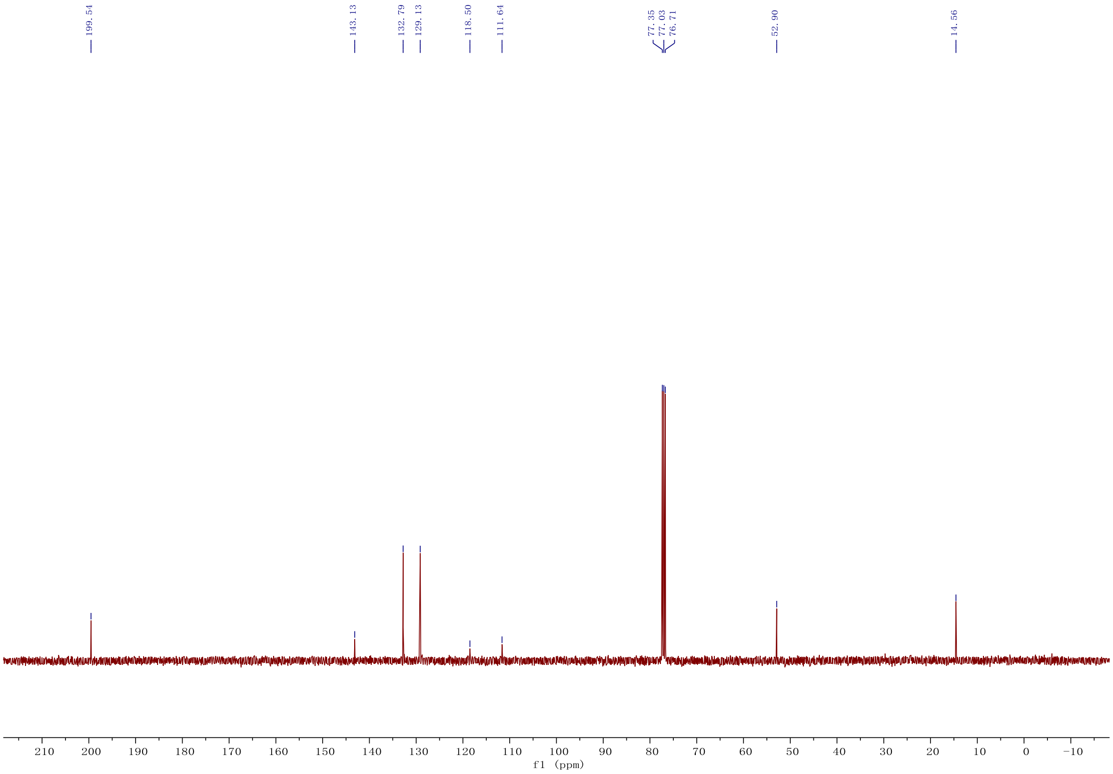

Supplementary Figure 174. ^{13}**C NMR** (101 MHz, Chloroform-*d*) of **3i**

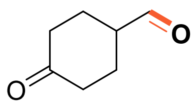

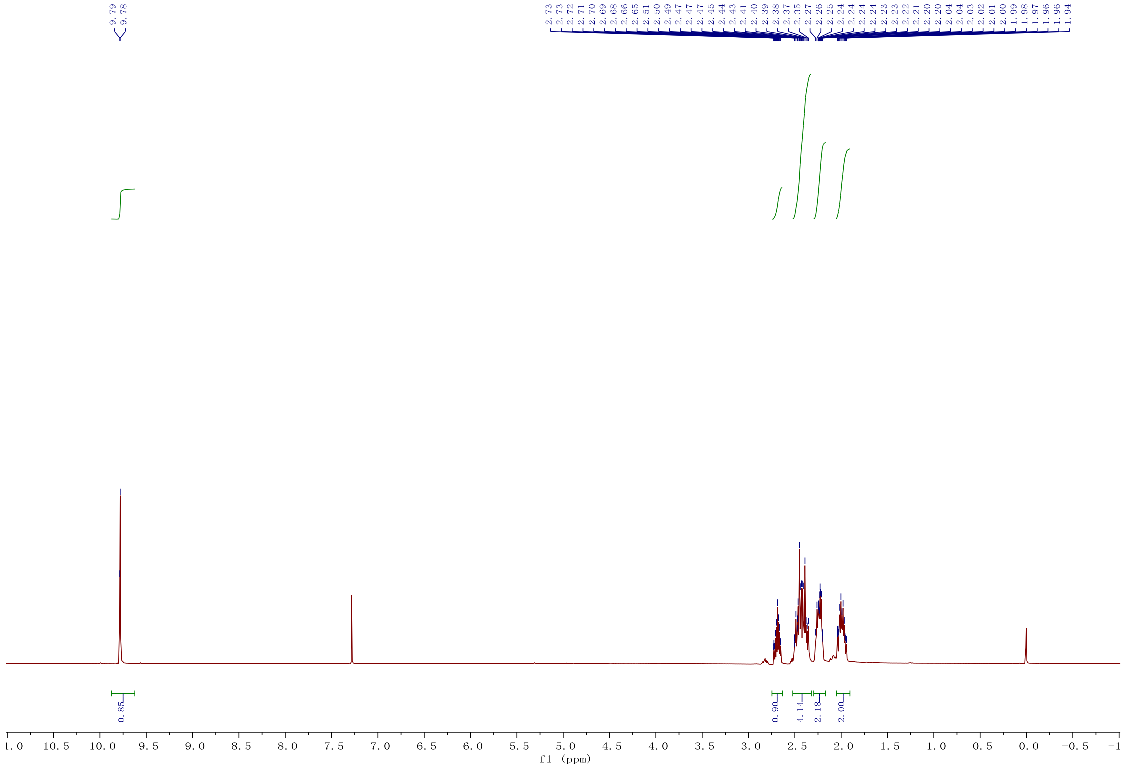

Supplementary Figure 175. ^{1}**H NMR** (400 MHz, Chloroform-*d*) of **3j**

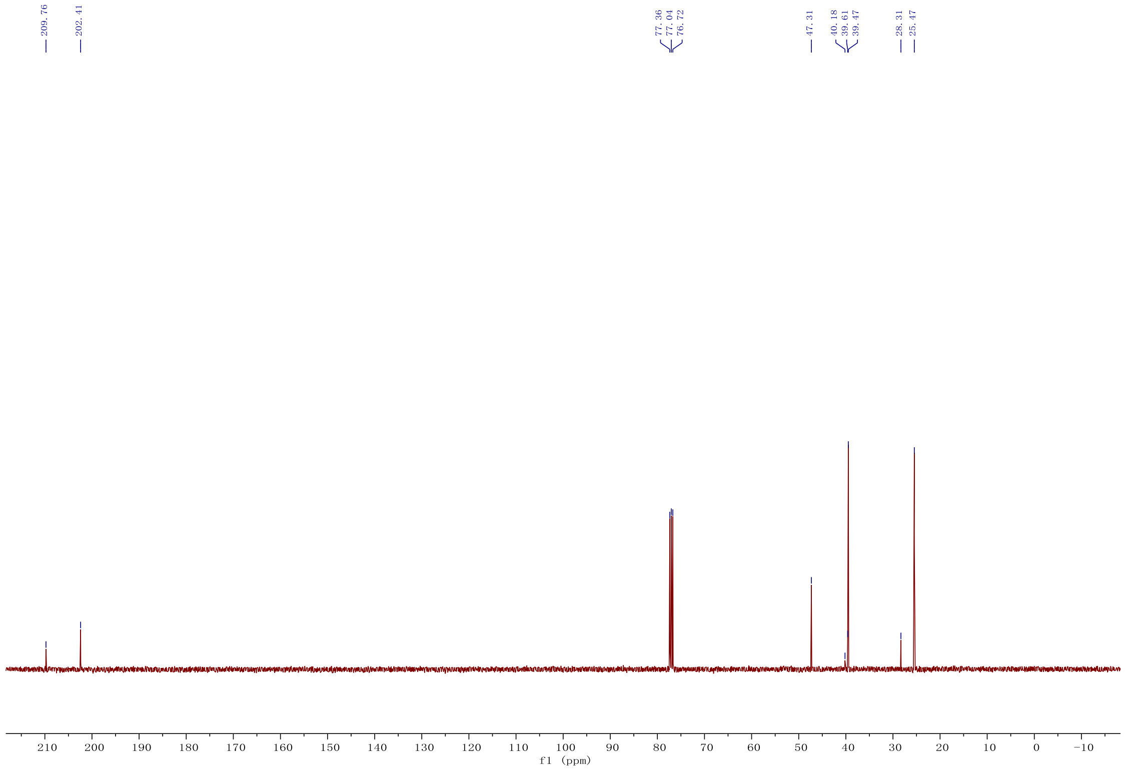

Supplementary Figure 176. ^{13}**C NMR** (101 MHz, Chloroform-*d*) of **3j**

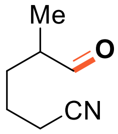

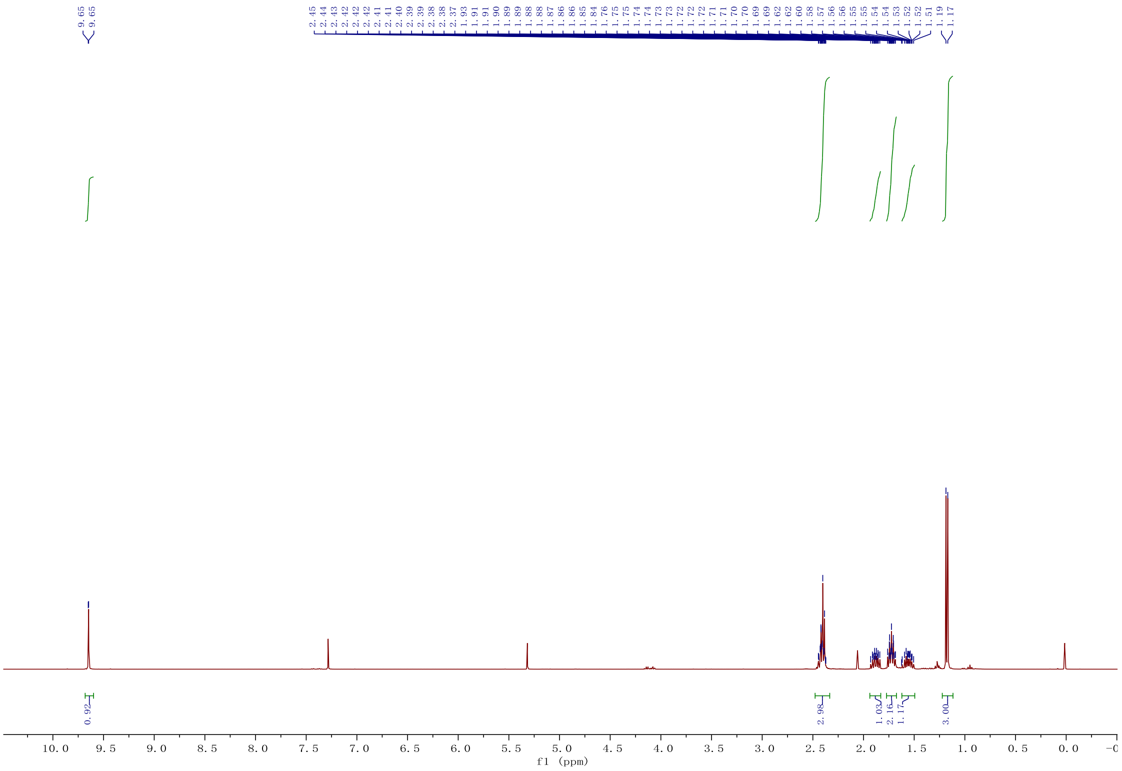

Supplementary Figure 177. ^{1}**H NMR** (400 MHz, Chloroform-*d*) of **3k**

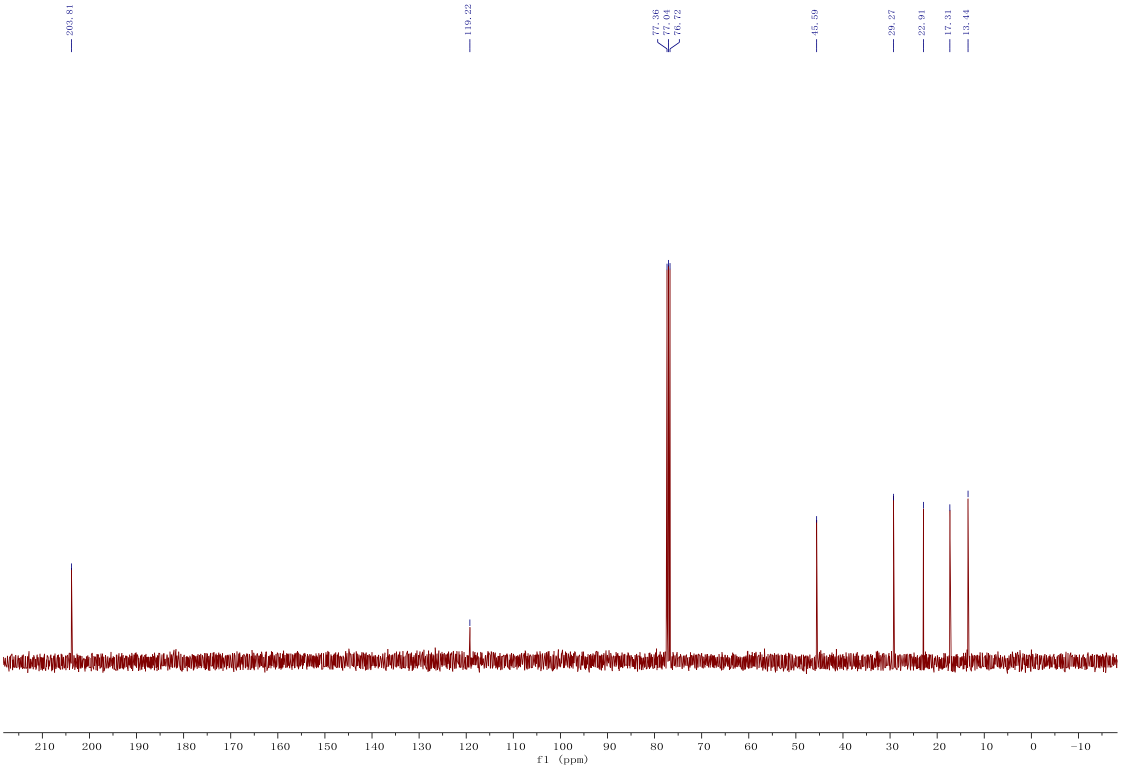

Supplementary Figure 178. ^{13}**C NMR** (101 MHz, Chloroform-*d*) of **3k**

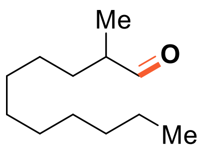

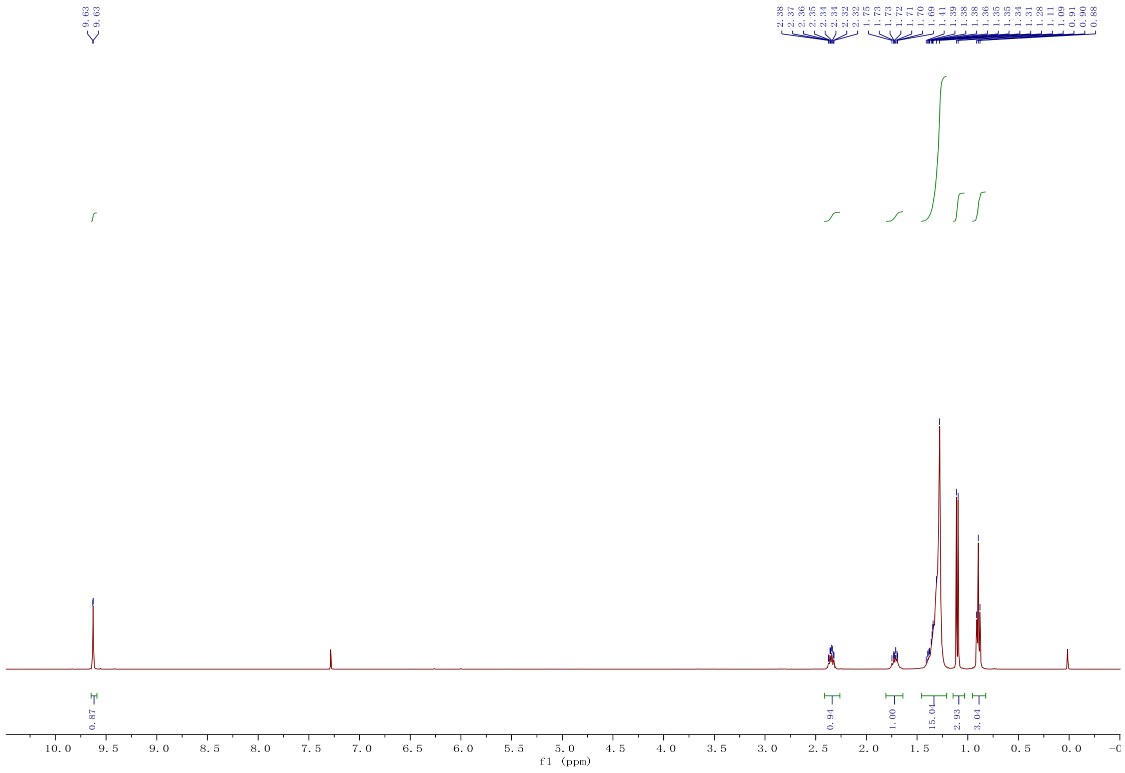

Supplementary Figure 179. ^{1}H NMR (400 MHz, Chloroform-*d*) of **3m**

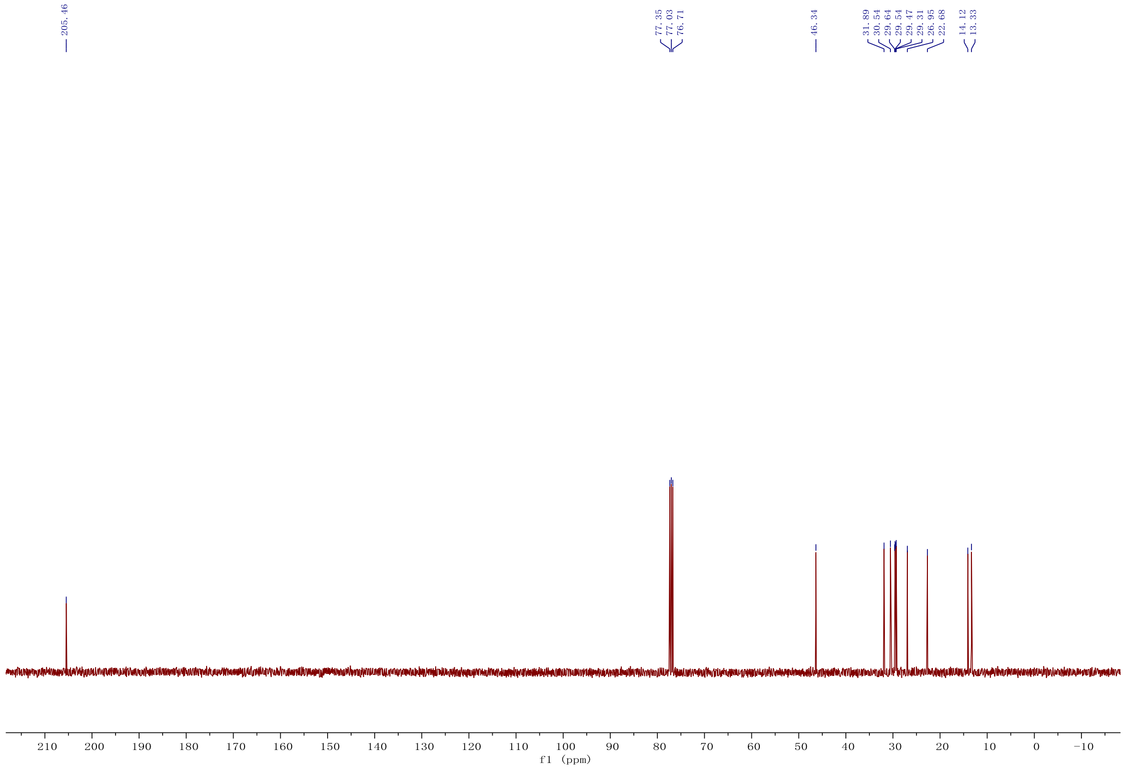

Supplementary Figure 180. ^{13}C NMR (101 MHz, Chloroform-*d*) of **3m**

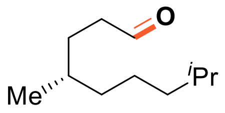

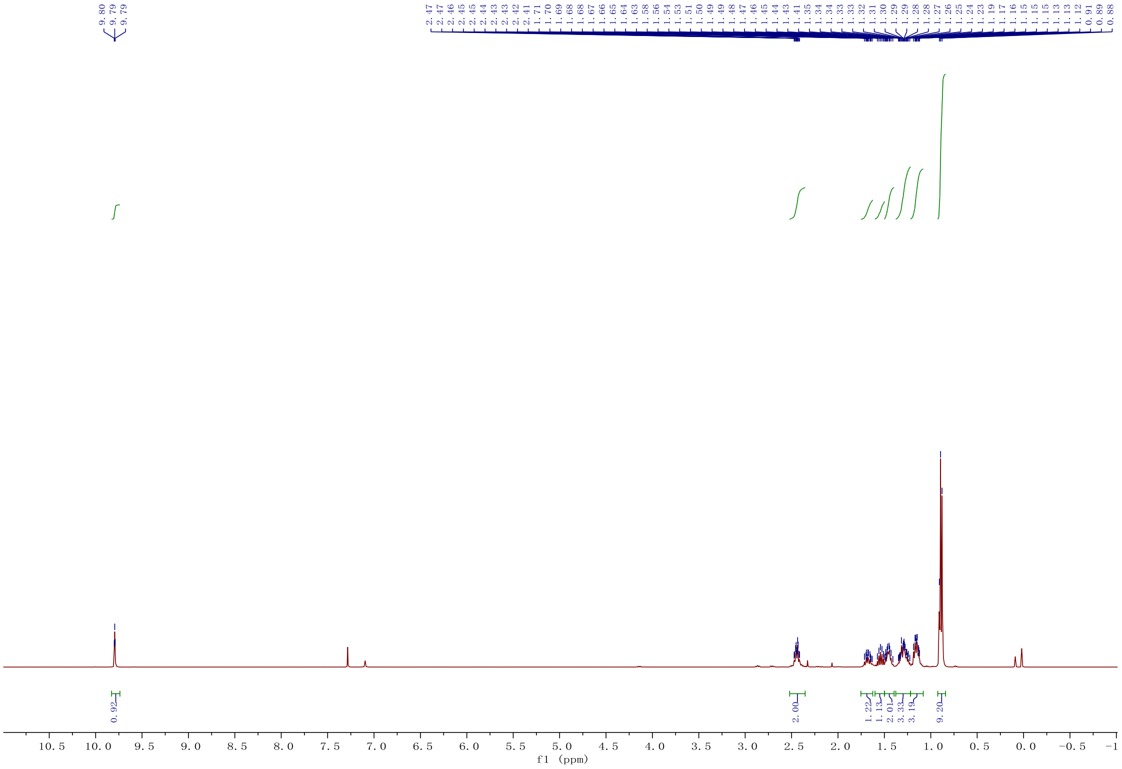

Supplementary Figure 181. **^{1}H NMR** (400 MHz, Chloroform-*d*) of **3n**

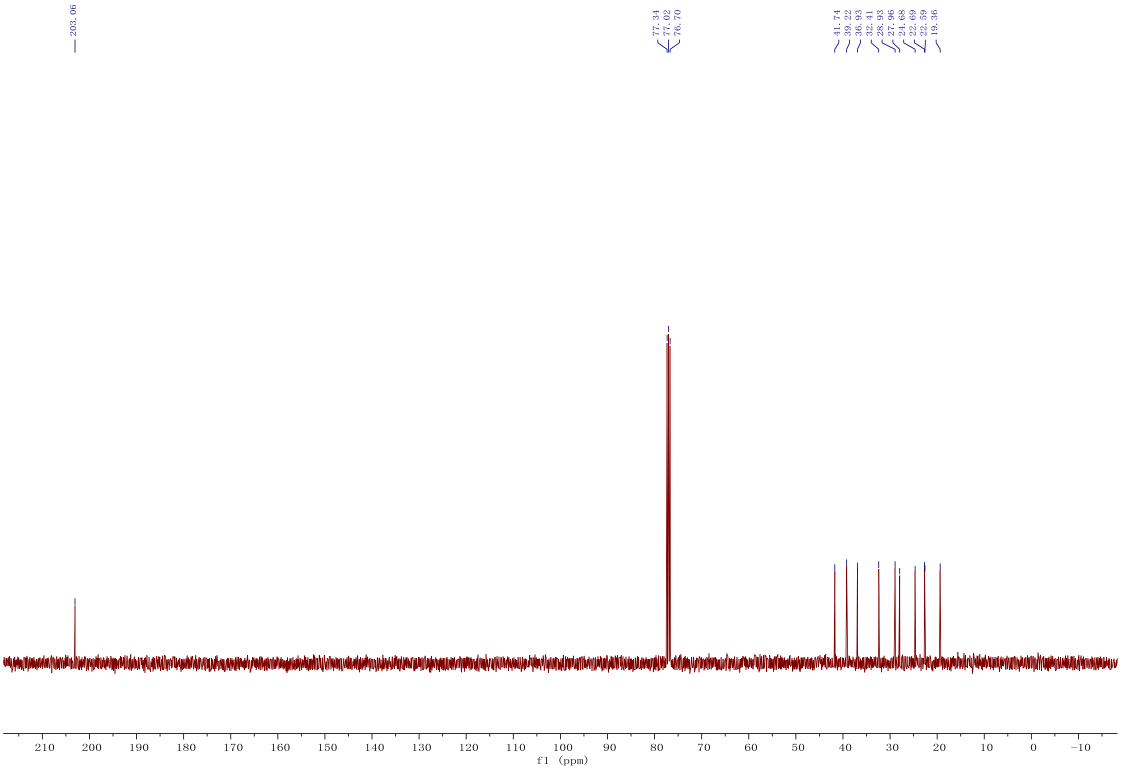

Supplementary Figure 182. **^{13}C NMR** (101 MHz, Chloroform-*d*) of **3n**

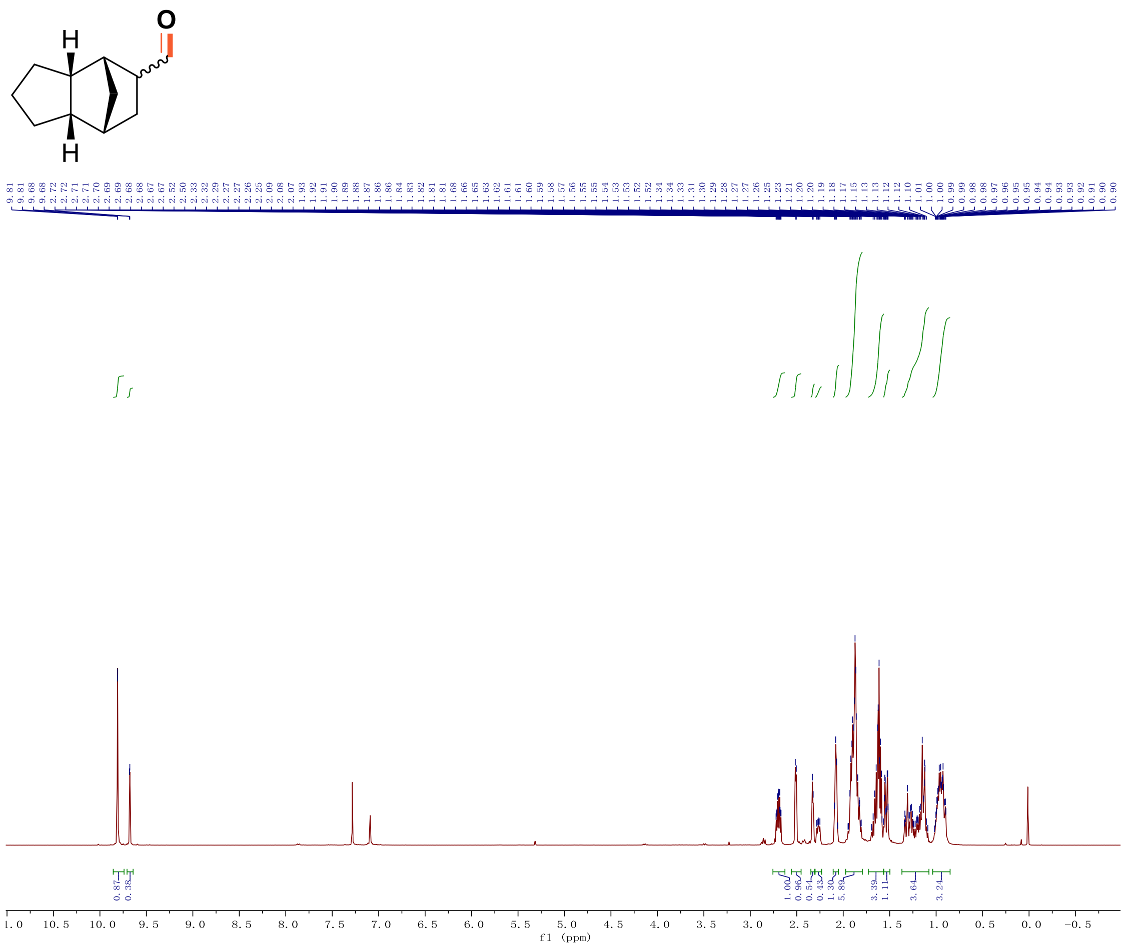

Supplementary Figure 183. ^{1}H NMR (400 MHz, Chloroform-*d*) of **3p**

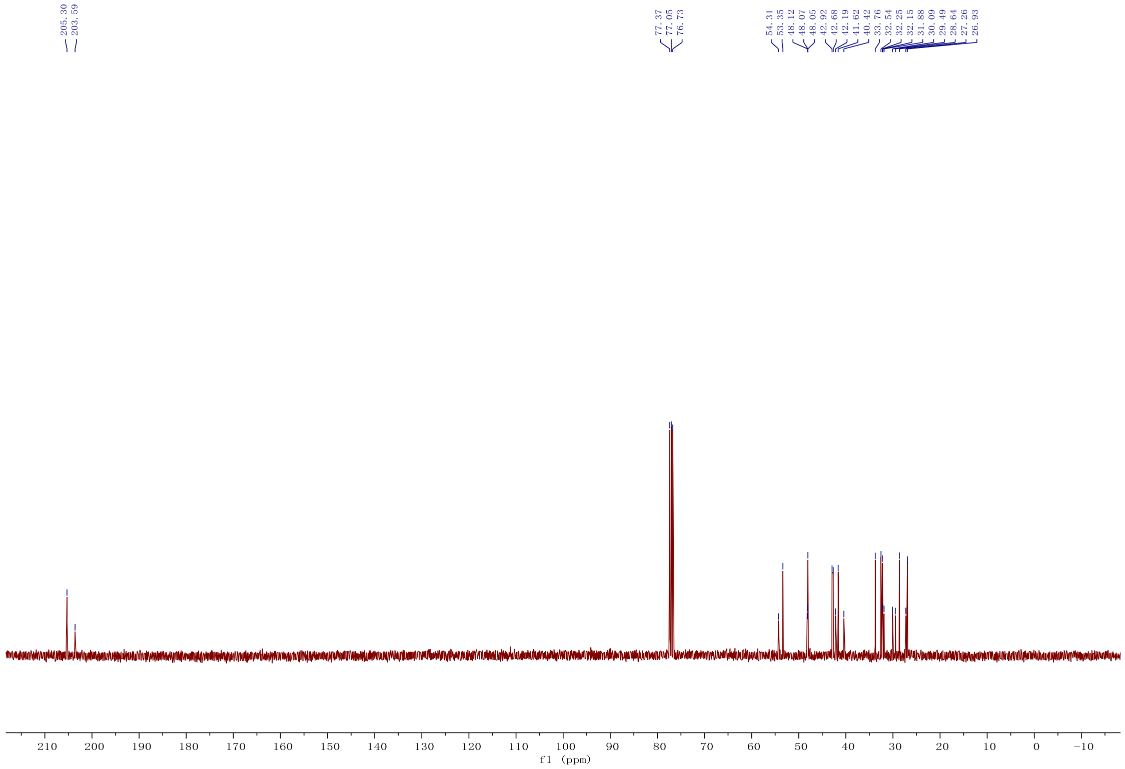

Supplementary Figure 184. ^{13}C NMR (101 MHz, Chloroform-*d*) of **3p**

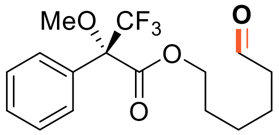

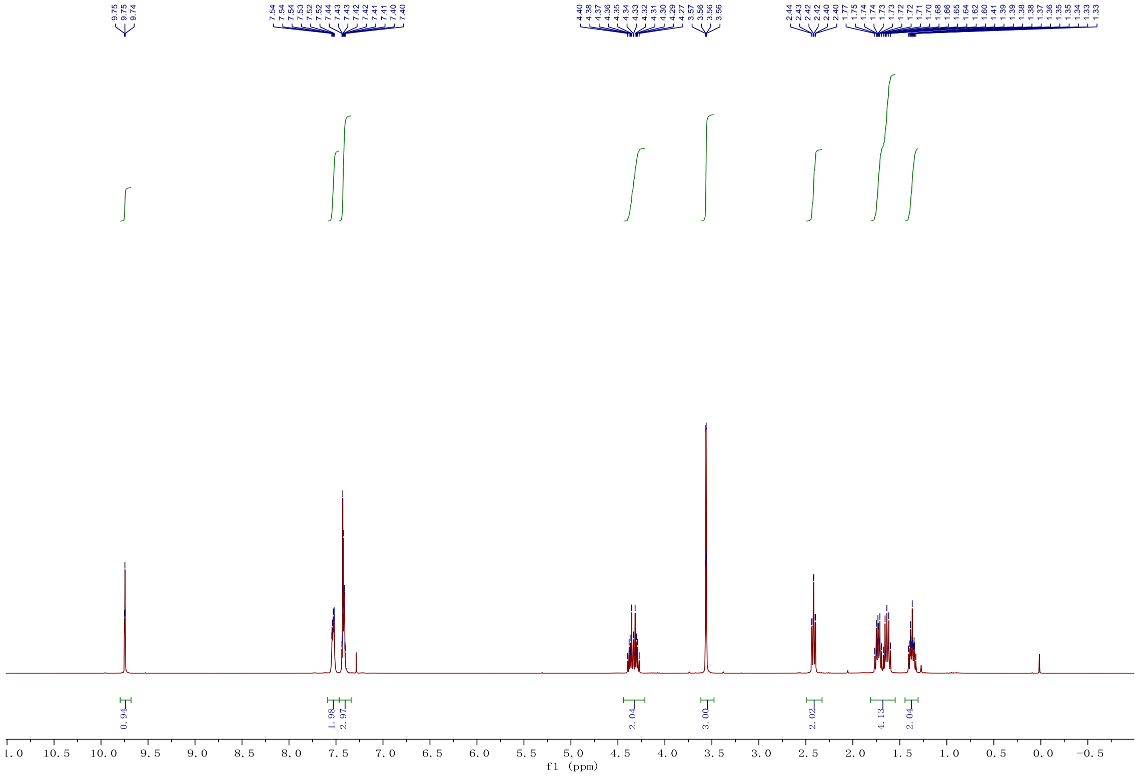

Supplementary Figure 185. **^{1}H NMR** (400 MHz, Chloroform-*d*) of **3q**

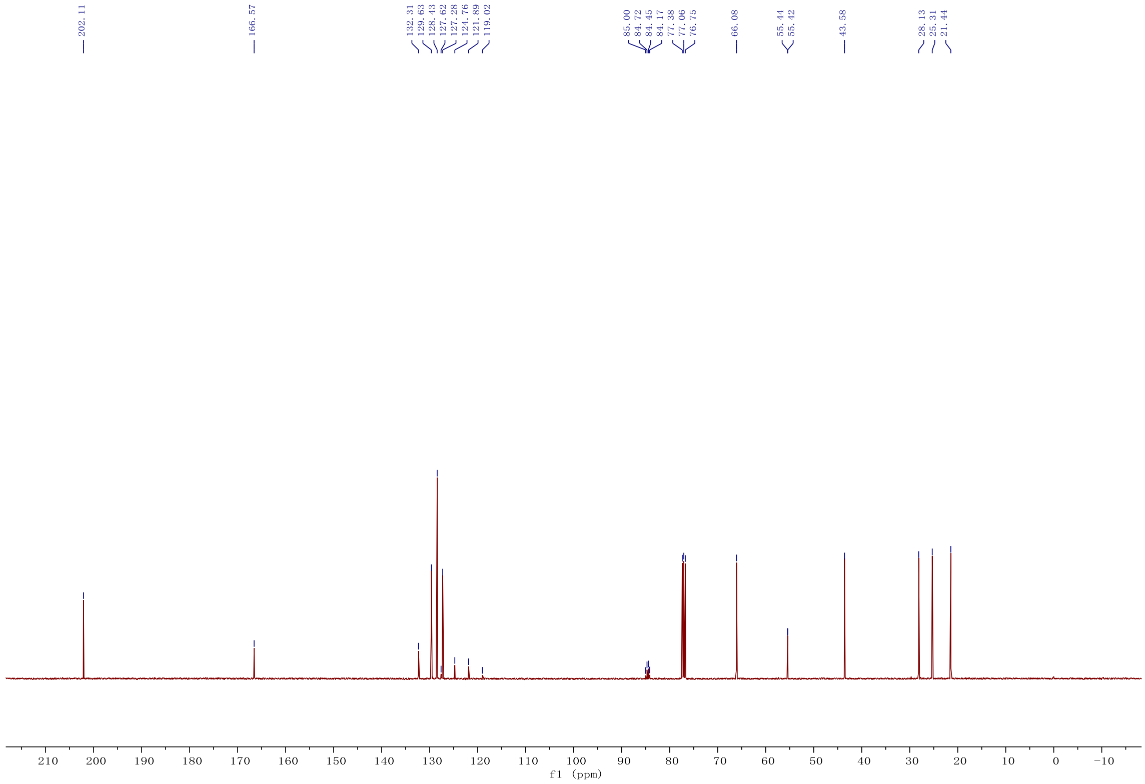

Supplementary Figure 186. **^{13}C NMR** (101 MHz, Chloroform-*d*) of **3q**

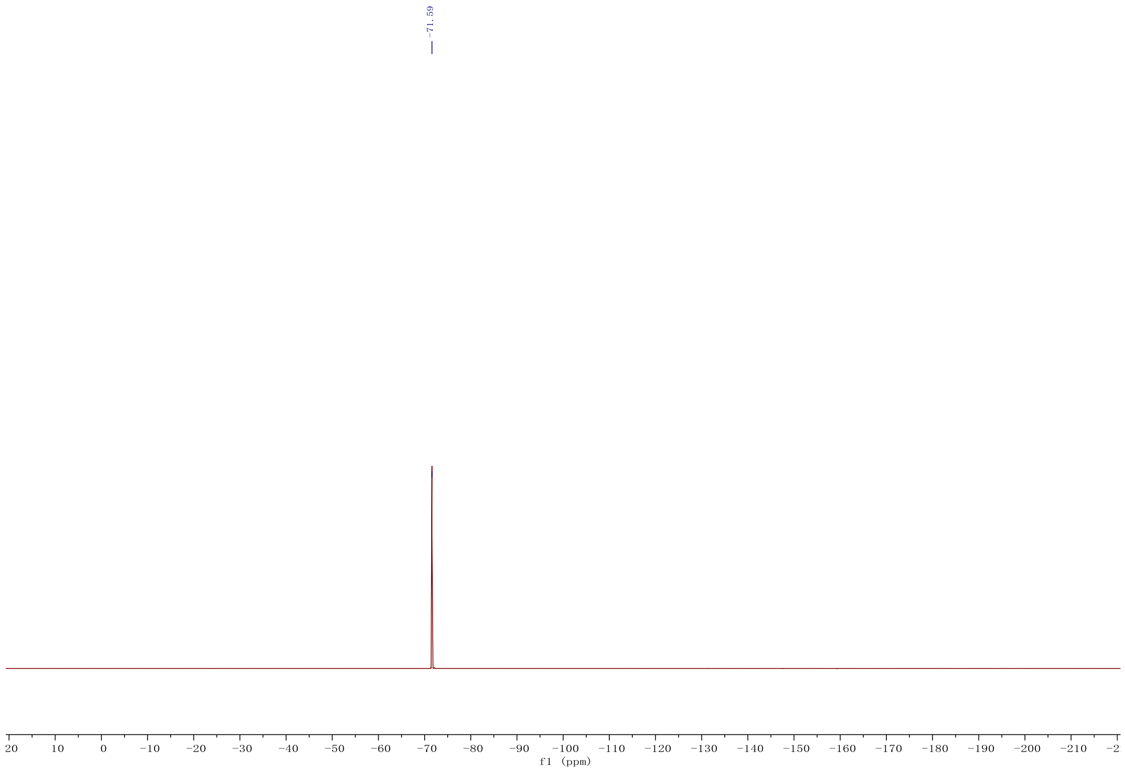

Supplementary Figure 187. ^{19}F NMR (376 MHz, Chloroform-*d*) of **3q**

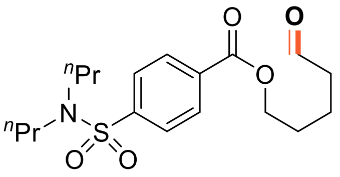

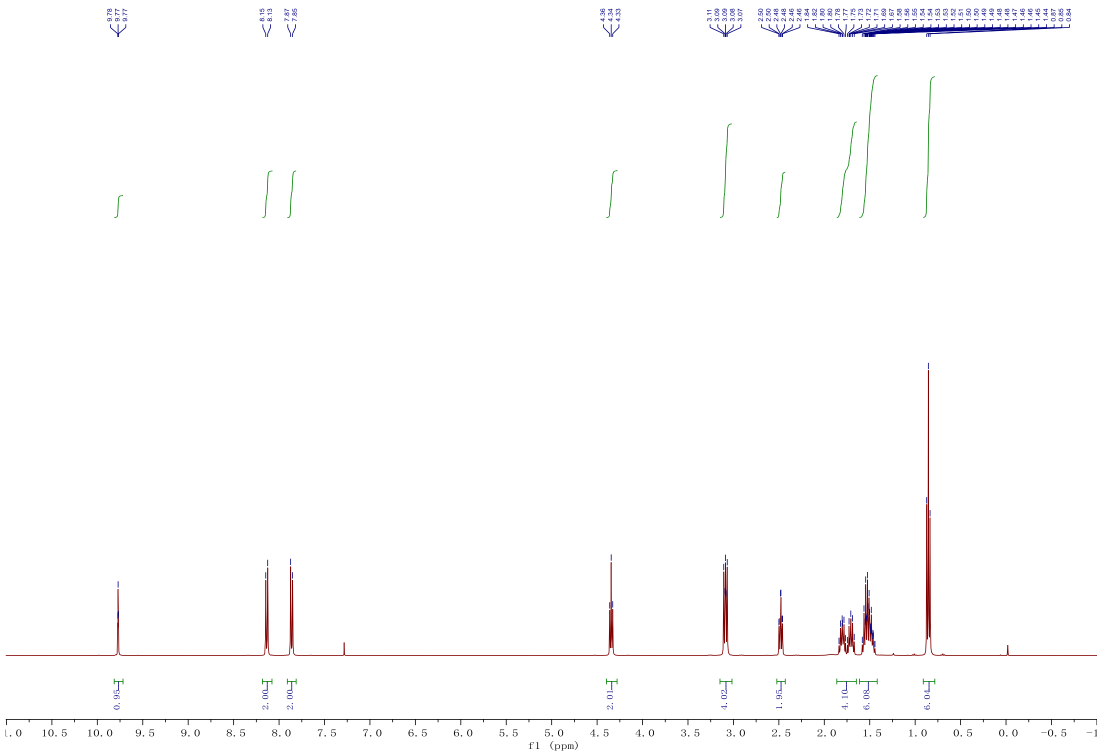

Supplementary Figure 188. ^{1}H NMR (400 MHz, Chloroform-*d*) of **3r**

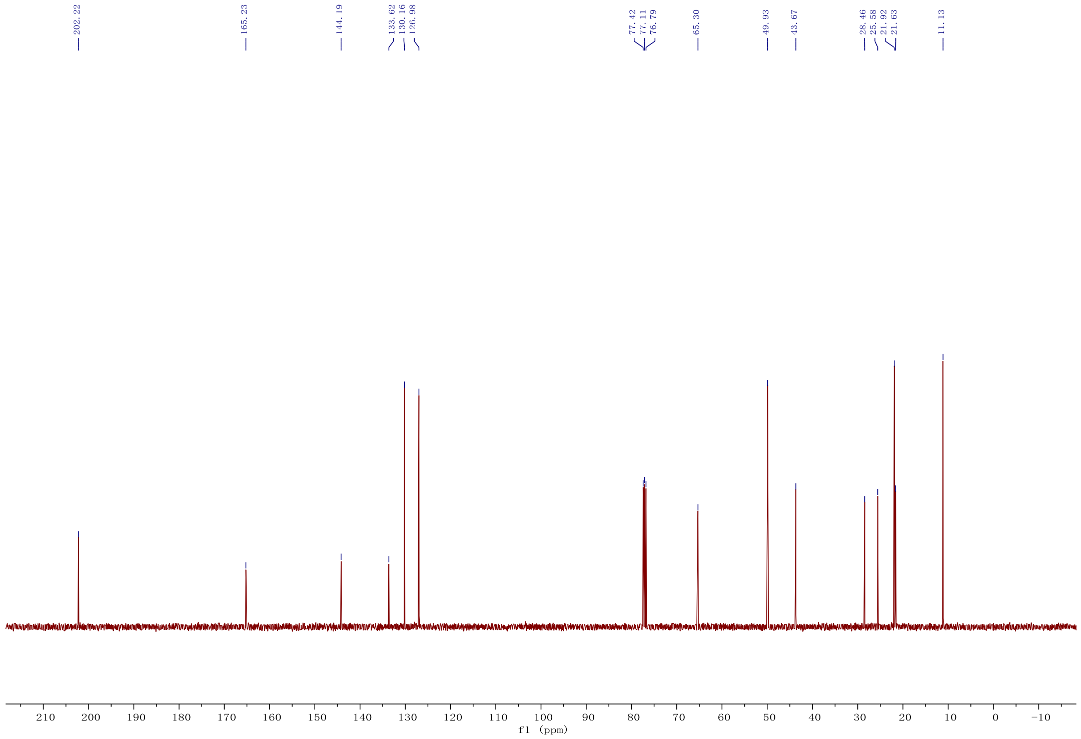

Supplementary Figure 189. ^{13}C NMR (101 MHz, Chloroform-*d*) of **3r**

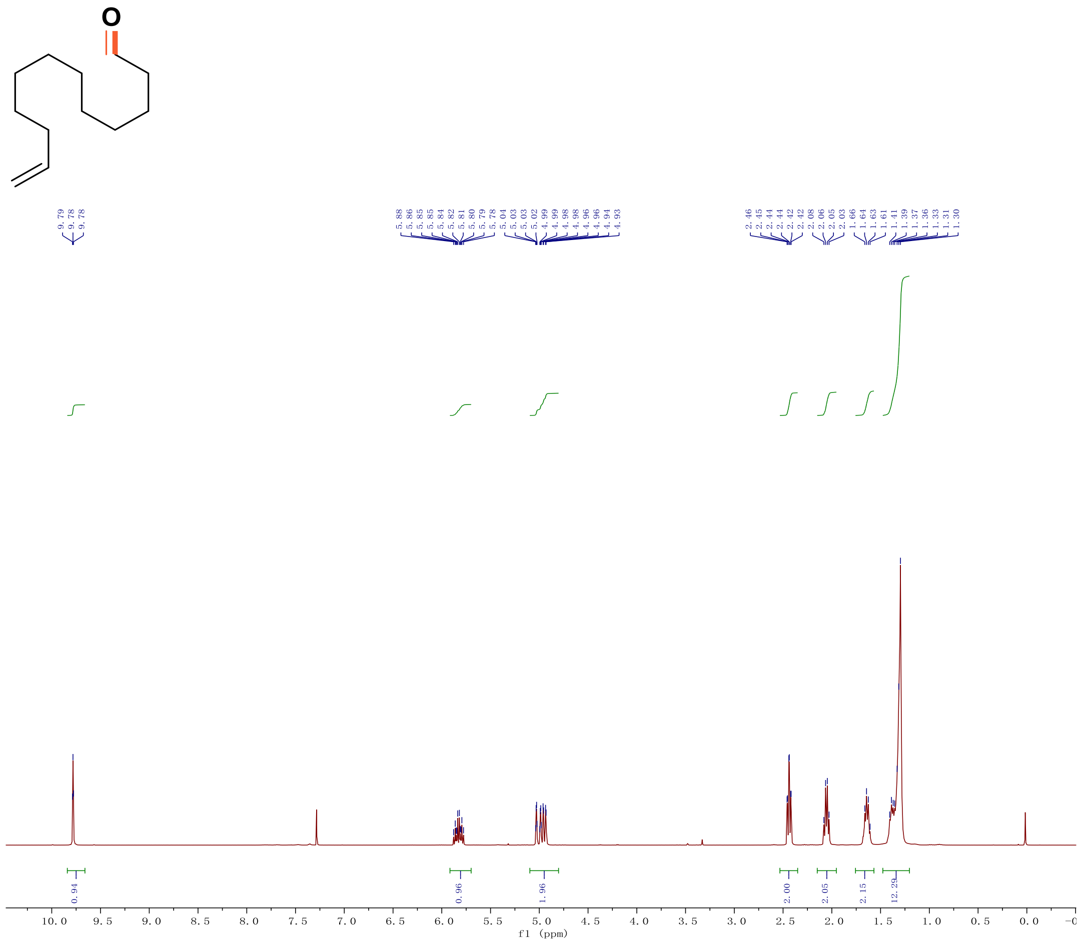

Supplementary Figure 190. ^{1}**H NMR** (400 MHz, Chloroform-*d*) of **3s**

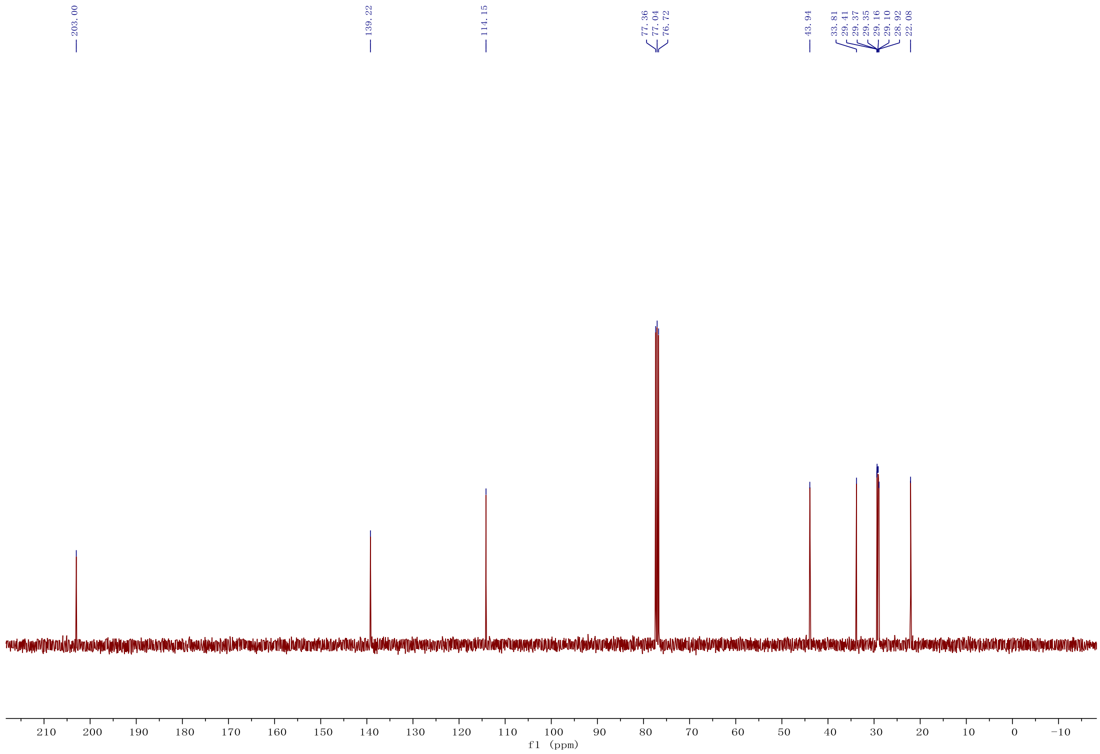

Supplementary Figure 191. ^{13}**C NMR** (101 MHz, Chloroform-*d*) of **3s**

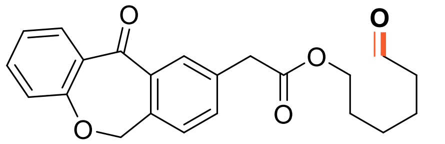

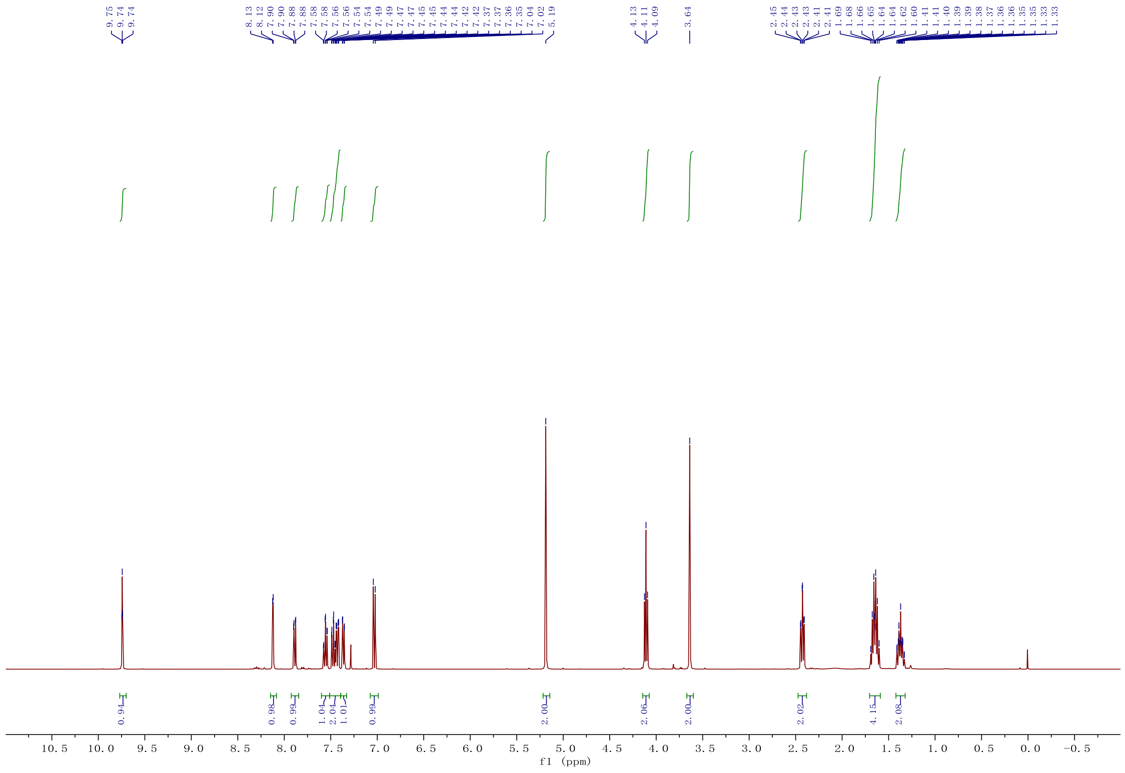

Supplementary Figure 192. ^{1}H NMR (400 MHz, Chloroform-*d*) of **3t**

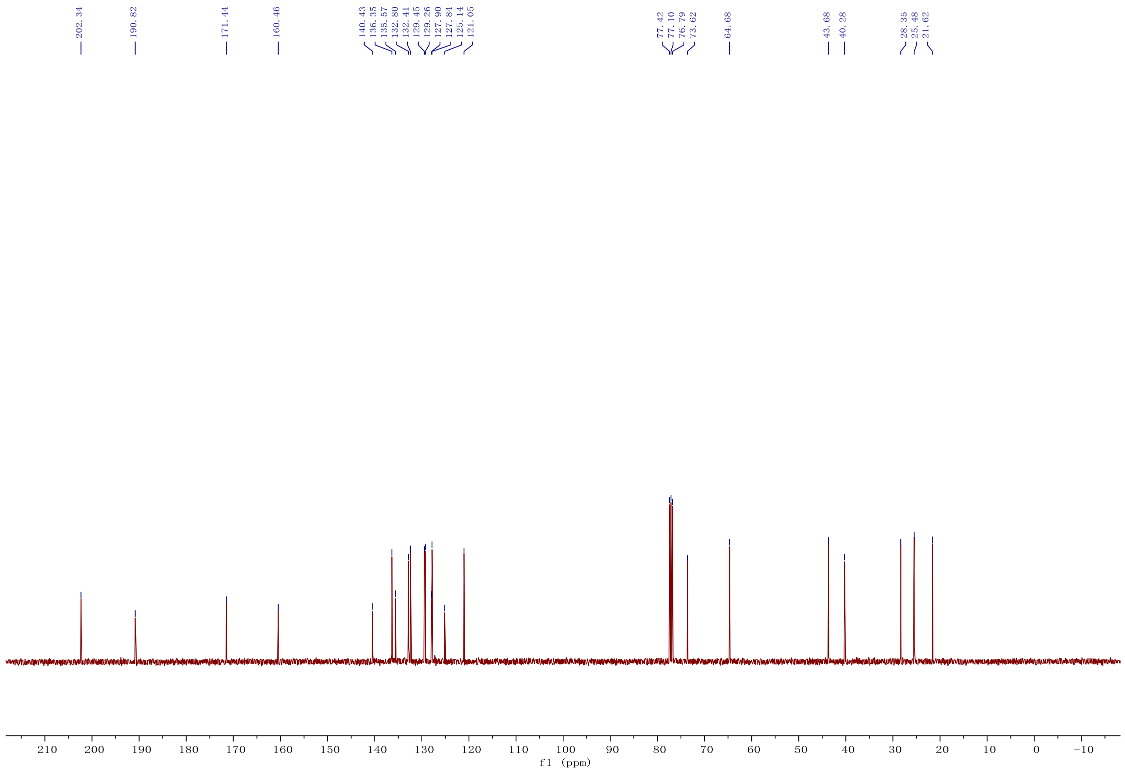

Supplementary Figure 193. ^{13}C NMR (101 MHz, Chloroform-*d*) of **3t**

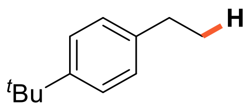

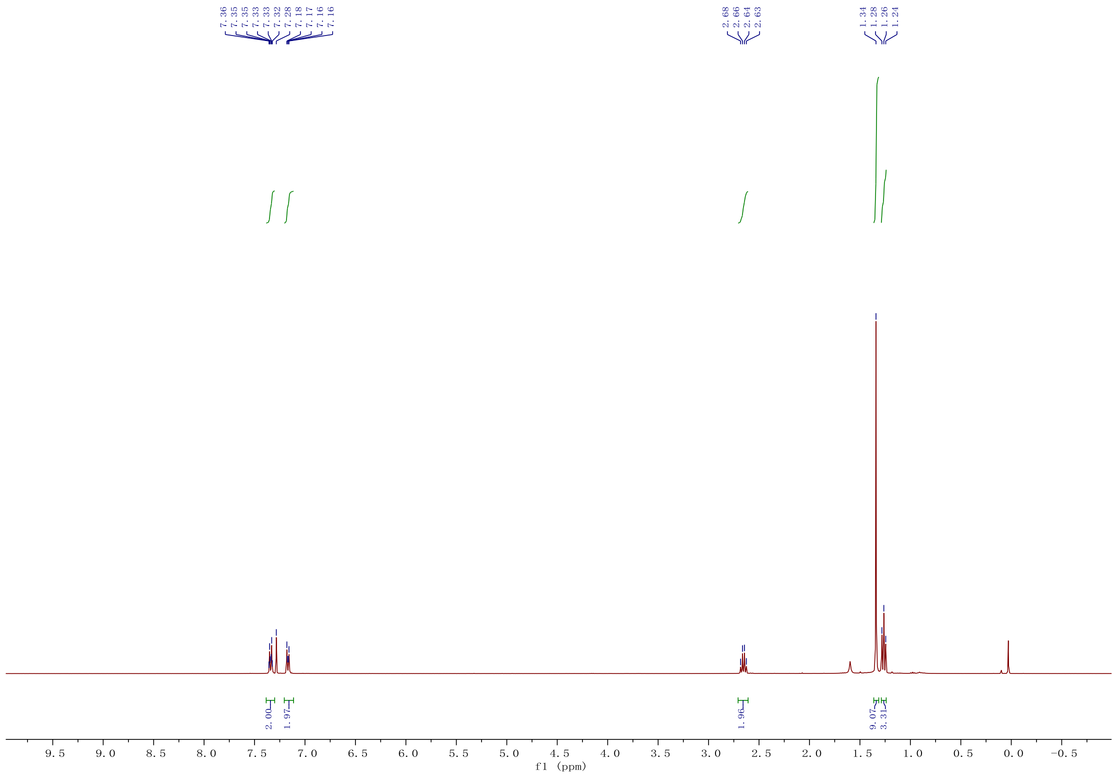

Supplementary Figure 194. ^{1}H NMR (400 MHz, Chloroform-*d*) of **4f**

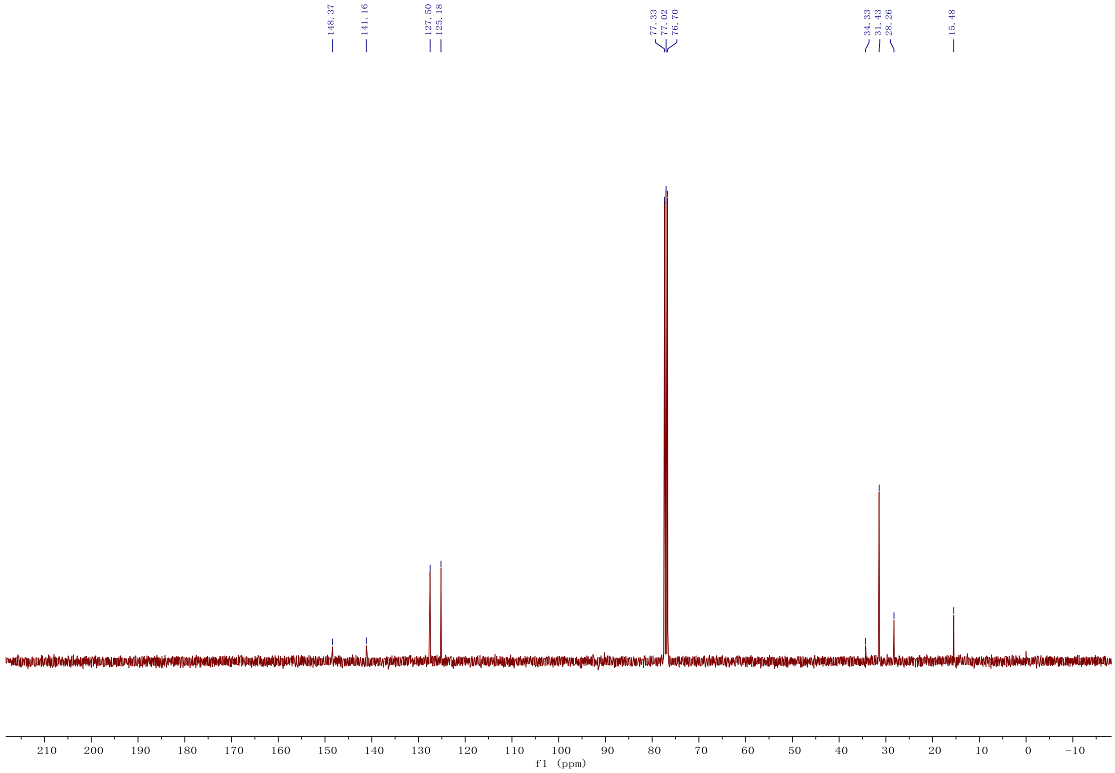

Supplementary Figure 195. ^{13}C NMR (101 MHz, Chloroform-*d*) of **4f**

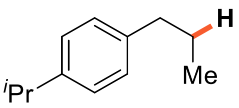

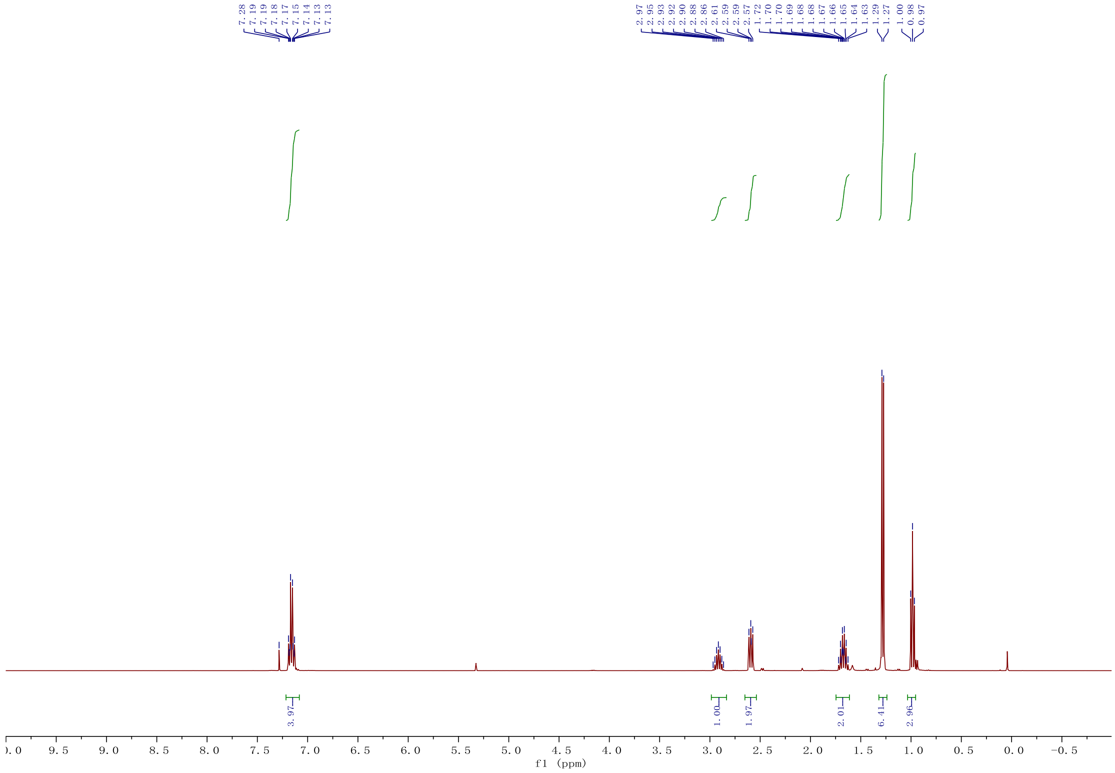

Supplementary Figure 196. **^{1}H NMR** (400 MHz, Chloroform-*d*) of **4g**

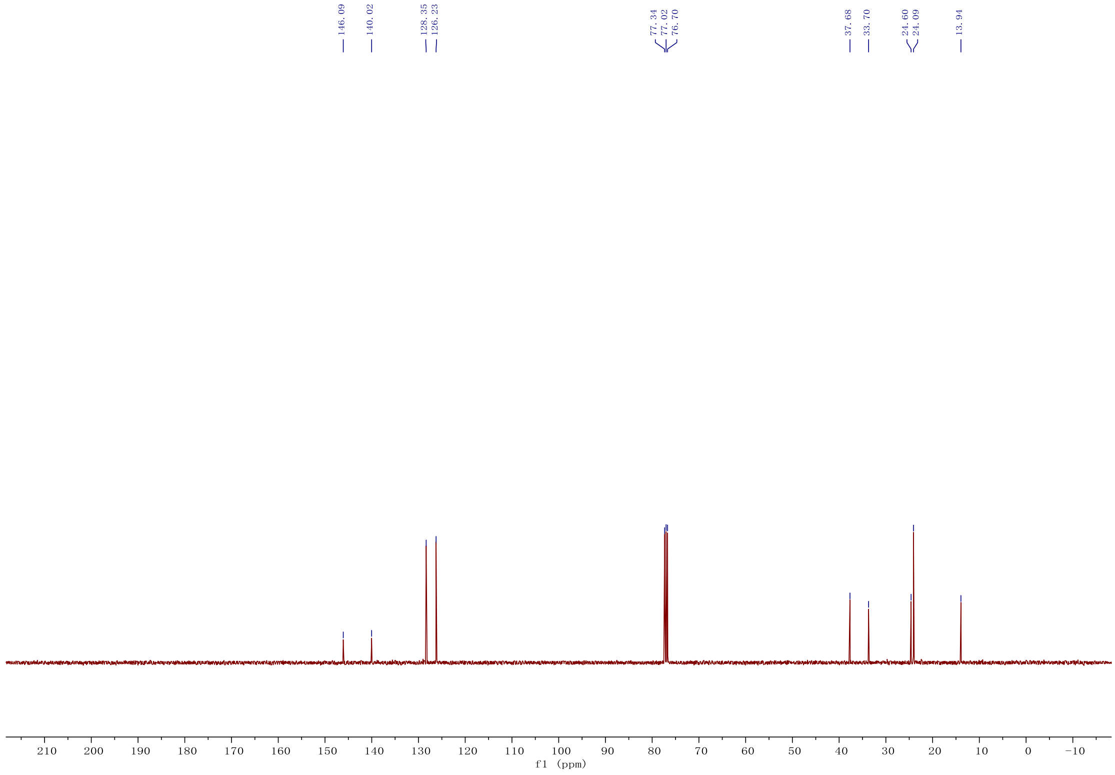

Supplementary Figure 197. **^{13}C NMR** (101 MHz, Chloroform-*d*) of **4g**

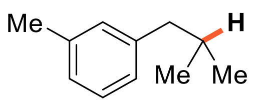

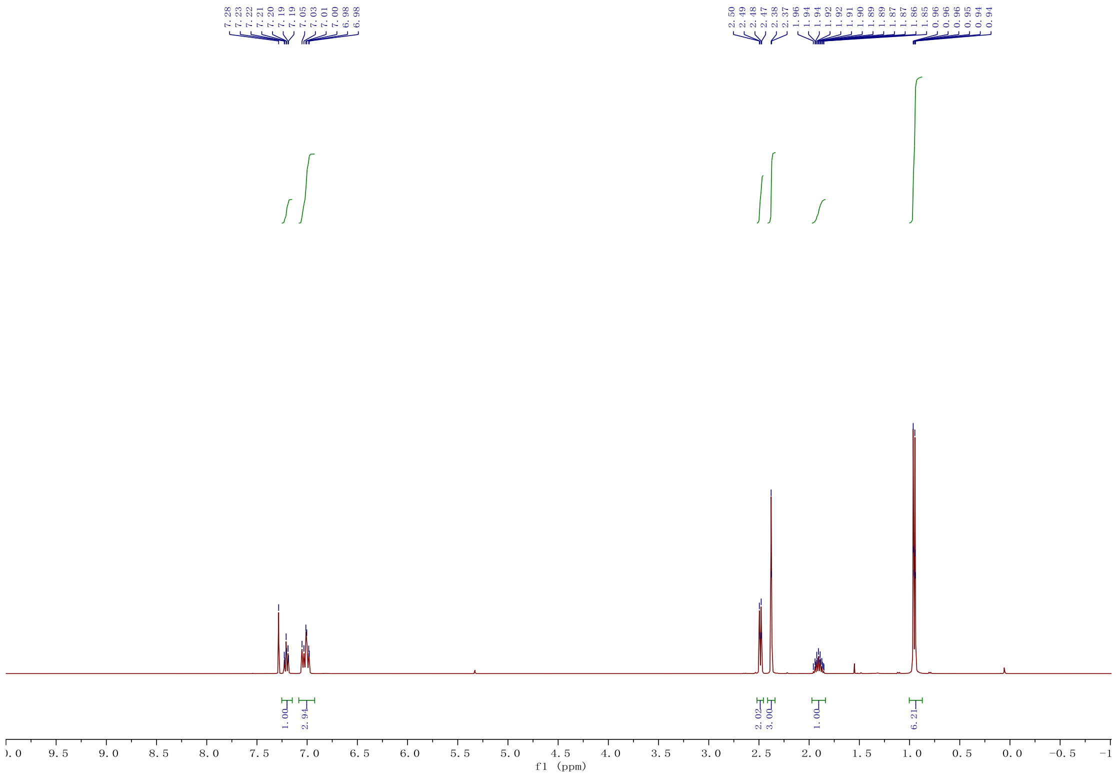

Supplementary Figure 198. ¹H NMR (400 MHz, Chloroform-*d*) of **4h**

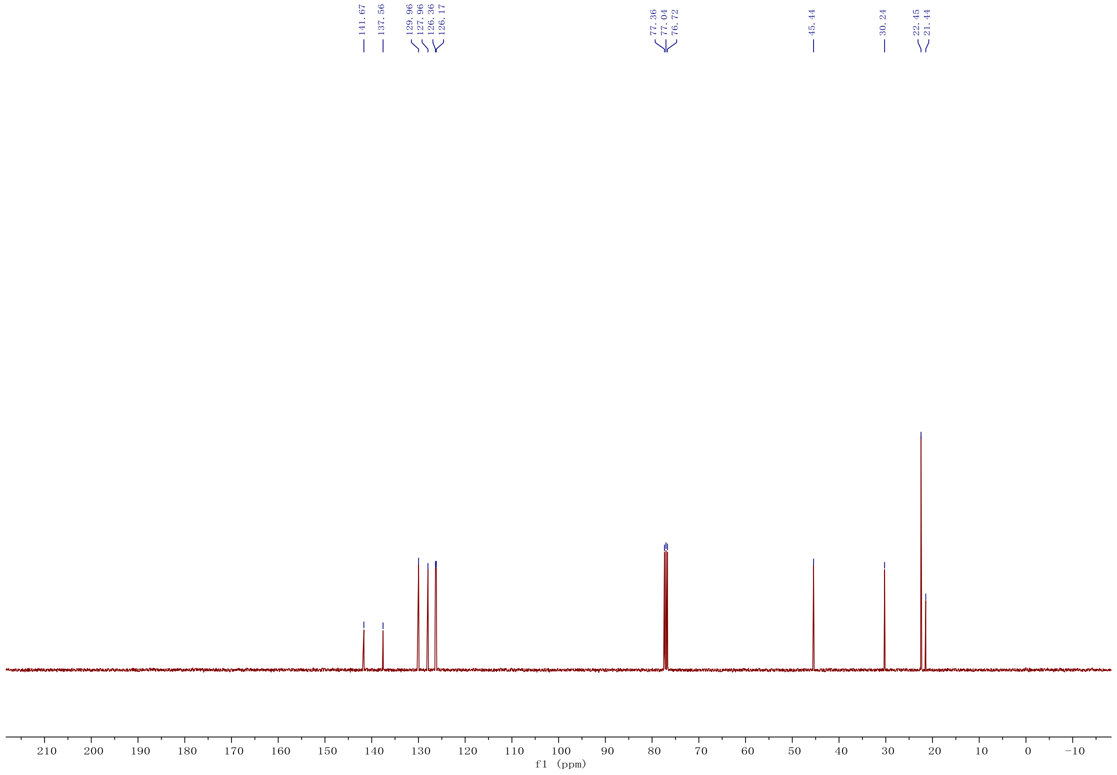

Supplementary Figure 199. ¹³C NMR (101 MHz, Chloroform-*d*) of **4h**

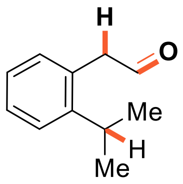

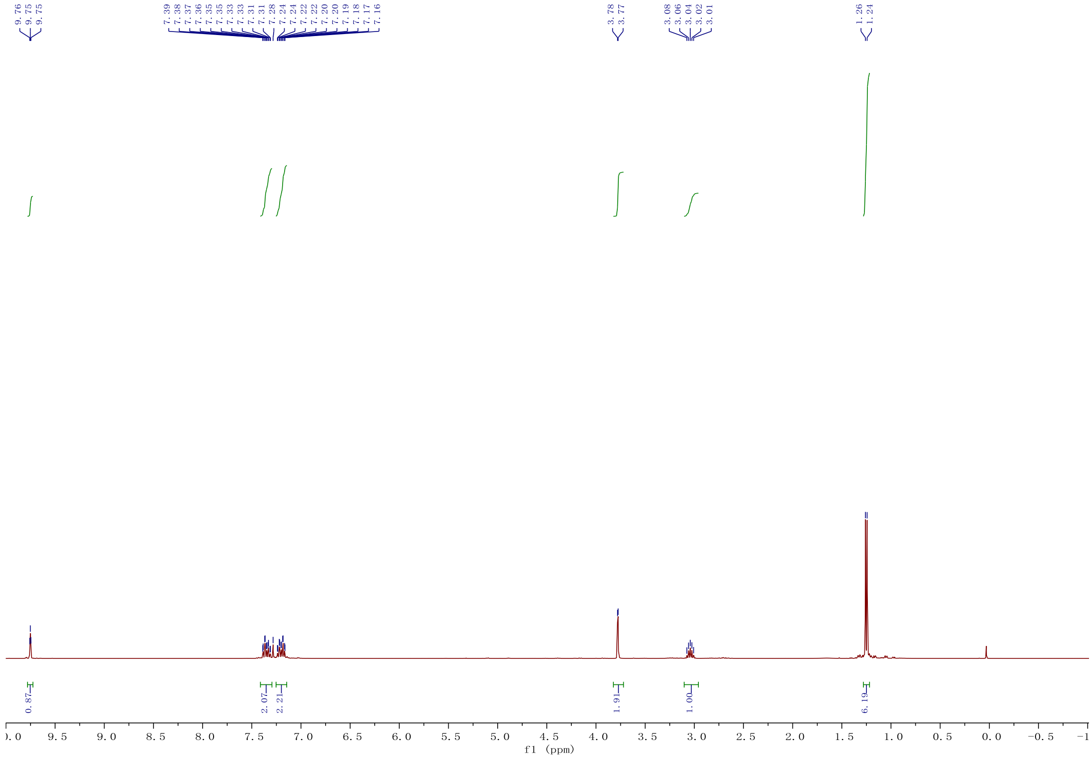

Supplementary Figure 200. **^{1}H NMR** (400 MHz, Chloroform-*d*) of **5b**

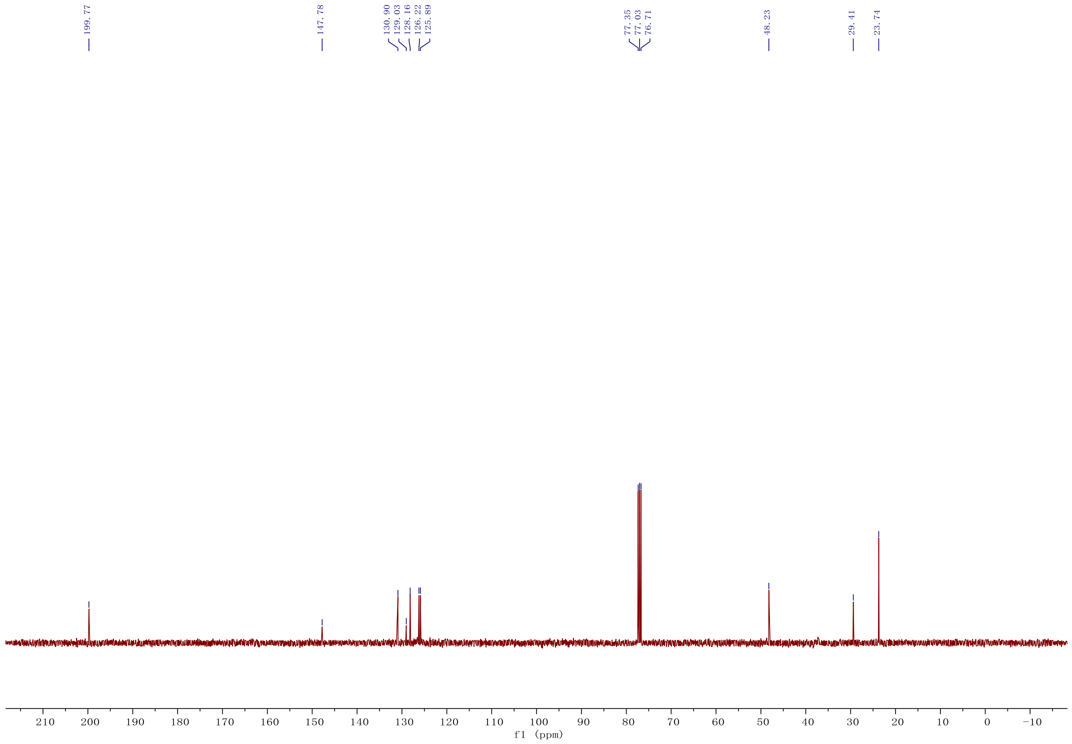

Supplementary Figure 201. **^{13}C NMR** (101 MHz, Chloroform-*d*) of **5b**

6. **References**

1. Kustiana, B. A.; Elsherbeni, S. A.; Linford-Wood, T. G.; Melen, R. L.; Grayson, M. N.; Morrill, L. C. B(C_6F_5)$_3$-catalyzed E-selective isomerization of alkenes. *Chem. Eur. J.* **28**, e202202454 (2022).
2. Qrareya, H.; Raviola, C.; Protti, S.; Fagnoni, M.; Albini, A. Transition-metal-free arylations via photogenerated triplet 4-alkyl- and 4-trimethylsilylphenyl cations. *J. Org. Chem.* **78**, 6016-6024 (2013).
3. Gerbino, D. C.; Mandolesi, S. D.; Schmalz, H. G.; Podestá, J. C. Introduction of allyl and prenyl side-chains into aromatic systems by Suzuki cross-coupling reactions. *Eur. J. Org. Chem.* **2009**, 3964-3972 (2009).
4. Lin, S.; Song, C. X.; Cai, G. X.; Wang, W. H.; Shi, Z. J. Intra/intermolecular direct allylic alkylation via Pd(II)-catalyzed allylic C-H activation. *J. Am. Chem. Soc.* **130**, 12901-12903 (2008).
5. Wu, F. P.; Li, D.; Peng, J. B.; Wu, X. F. Carbonylative transformation of allylarenes with CO surrogates: tunable synthesis of 4-arylbutanoic acids, 2-arylbutanoic acids, and 4-arylbutanals. *Org. Lett.* **21**, 5699-5703 (**2019**).
6. Zhang, S.; Ullah, A.; Yamamoto, Y.; Bao, M. Palladium-catalyzed regioselective allylation of chloromethyl(hetero)arenes with allyl pinacolborate. *Adv. Syn. Cat.* **359**, 2723-2728 (2017).
7. Glunz, P. W.; Mueller, L.; Cheney, D. L.; Ladziata, V.; Zou, Y.; Wurtz, N. R.; Wei, A.; Wong, P. C.; Wexler, R. R.; Priestley, E. S. Atropisomer control in macrocyclic factor VIIa inhibitors. *J. Med. Chem.* **59**, 4007-4018 (2016).
8. Scrivanti, A.; Beghetto, V.; Bertoldini, M.; Matteoli, U. Catalyst-free Suzuki-type coupling of allylic bromides with arylboronic acids. *Eur. J. Org. Chem.* **2012**, 264-268 (2011).
9. Goodman, J. L.; Berson, J. A. m-Quinodimethane, parent hydrocarbon of the m-quinonoid non-Kekule series. Low-temperature isolation and solution-phase chemical reactivity. *J. Am. Chem. Soc.* **107**, 5409-5424 (1985).
10. Zhang, Z. Y.; Liu, Z. Y.; Guo, R. T.; Zhao, Y. Q.; Li, X.; Wang, X. C. B(C_6F_5)$_3$-catalyzed ring opening and isomerization of unactivated cyclopropanes. *Angew. Chem. Int. Ed.* **56**, 4028-4032 (2017).
11. Yang, S.; Li, Z.; Jian, X.; He, C. Platinum(II)-catalyzed intramolecular cyclization of o-substituted aryl alkynes through sp3 C-H activation. *Angew. Chem. Int. Ed.* **48**, 3999-4001 (2009).
12. Tsukamoto, H.; Uchiyama, T.; Suzuki, T.; Kondo, Y. Palladium(0)-catalyzed direct cross-coupling reaction of allylic alcohols with aryl- and alkenylboronic acids. *Org. Biomol. Chem.* **6**, 3005-3013 (2008).
